# HIV and adolescents: focus on young key populations

**DOI:** 10.7448/IAS.18.2.20076

**Published:** 2015-02-26

**Authors:** Linda-Gail Bekker, Sybil Hosek

## Building our youth for the future

We cannot always build the future for our youth, but we can build our youth for the future.

—Franklin D. Roosevelt

The inaugural summary on the Global Youth Wellbeing Index reports that a sobering 85% of youth (age 10 to 24) in the 30 countries included report low levels of overall well-being [1]. The overall well-being score, as defined by the index, is composed of six domains shown in Table 1.

**Table 1:** Domains considered in the Global Youth Wellbeing Index

Domain
Citizen participation
Economic opportunity
Education
Health
Information and communication technology
Safety and security

**Figure 1:**
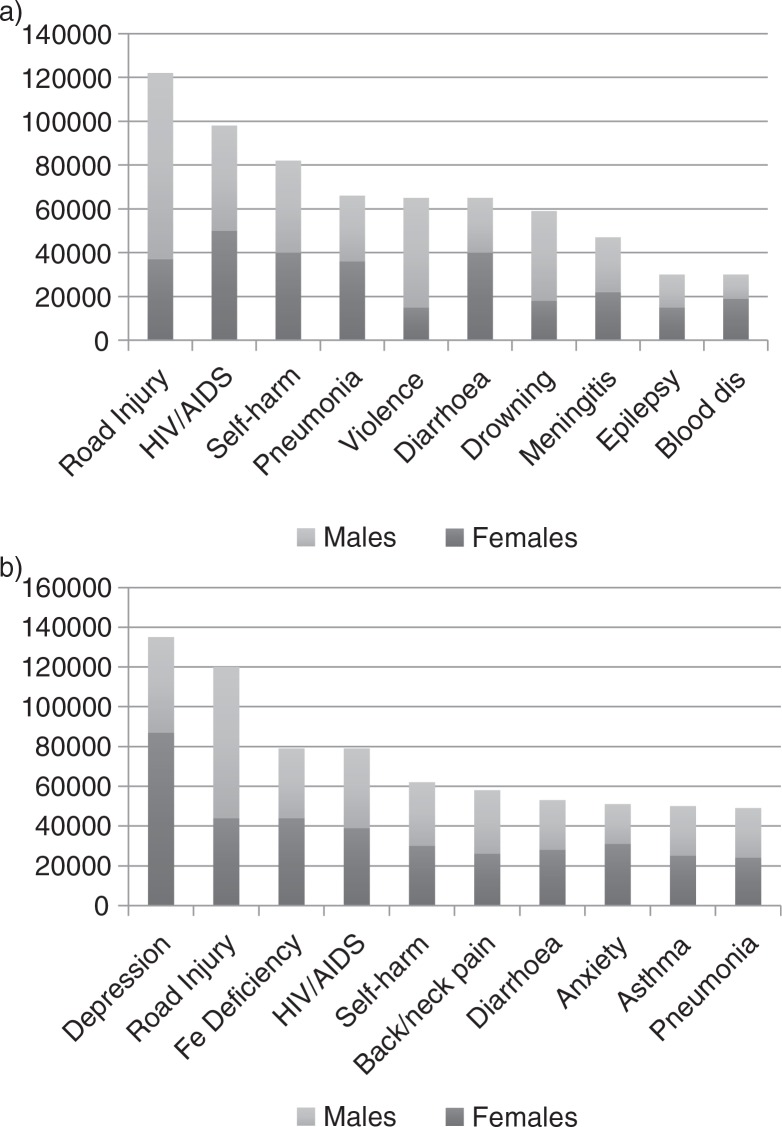
a) Top 10 causes of mortality in adolescents worldwide. b) Top 10 causes of disability-adjusted life years in adolescents worldwide. Adapted from World Health Organization (WHO) [2].

If these youth are representative of the one billion youth alive in the world today, then we must ask ourselves where have we failed and what more we can do [2]. Young people are our future as well as the world's greatest resource. Overall, there were an estimated 1.3 million adolescent deaths in 2012, most of them from causes that could have been prevented or treated. Mortality is higher in boys than in girls and in older adolescents (15 to 19 years) than in the younger group (10 to 14 years). Whereas there are many causes of mortality common to boys and girls, violence is a particular problem in boys and maternal causes in girls [2]. Figure 1 shows the top 10 causes of death and disability-adjusted life-years lost in adolescents worldwide.

In contrast to reductions in other population groups, estimates suggest that numbers of HIV deaths are rising in the adolescent age group. This increase has occurred predominantly in the African region, resulting in AIDS being the leading cause of death among adolescents in Africa and the second leading cause for adolescents worldwide [3]. There are approximately four million young people aged 15 to 24 living with HIV globally, and 29% of those are adolescents aged 15 to 19 [4]. Between 2005 and 2012, the number of AIDS-related deaths decreased by 30% for all ages except among adolescents, who experienced a 50% increase in that same period. Similarly, two-thirds of new HIV infections in 2012 occurred among youth aged 15 to 24 [5]. HIV prevention and decreasing HIV-related deaths depend critically on reaching adolescents.

Young people, adolescents and young adults, are at increased risk for HIV due in part to the multiple transitions (i.e., biological, psychological) and developmental tasks (e.g., establishing identity) in this period of the lifespan. Among youth, there are also key populations that bear disproportionate burdens of HIV and are the most vulnerable. These young key populations (YKPs) include men who have sex with men (MSM), transgender people, those who inject drugs and sex workers, as well as youth who belong in multiple groups (e.g., transgender youth who inject drugs) [6].

### Young key population vulnerabilities

#### Young MSM

Among young MSM, HIV incidence has been shown to be very high across multiple countries, and global reports estimate an HIV prevalence of 4.2% for young gay men under the age of 25 [4, 7]. In the United States, MSM account for most (72%) new HIV infections among youth aged 13 to 24, making them the only group that has shown a significant increase in estimated new infections. Among young MSM in the United States, African-American/black youth bear the greatest burden of HIV [8]. Young MSM who engage in sex work are even more vulnerable to HIV. A recent study in Kenya found a baseline HIV prevalence of 40% among young MSM who sell sex in Nairobi [9].

#### Transgender youth

While there remains a paucity of studies that focus on transgender men (female-to-male), data from studies on transgender women (male-to-female) demonstrate they are up to 49 times more likely to acquire HIV than other adults, with an estimated 19% of transgender women infected with HIV [10]. There is also significant overlap for young transgender women with other key population categories, including drug use and sex work [11, 12]. For example, a study in Larkana, Pakistan, among transgender sex workers found an astonishing 27.6% HIV prevalence [13]. Secondary analysis of that data revealed that younger age (20 to 24 years) was strongly associated with higher HIV prevalence [14]. Community-based samples of young, transgender females in the United States have found self-reported rates of HIV infection ranging from 19 to 22% [12, 15].

#### Youth who inject drugs

Surveys have found very high HIV prevalence among young people who inject drugs. Globally, a recent report from the Joint United Nations Programme on HIV/AIDS [7] found that the HIV prevalence among young people under 25 who inject drugs was 5.2% [7]. In Russia, for example, the prevalence of HIV among injecting drug users under 25 was estimated at 12% [16]. Despite a decreasing trend, the HIV prevalence in Ukraine among youth under 25 that inject drugs remains at 7.2% [17]. When youth from other key populations also inject drugs, such as sex workers and transgender youth, the HIV prevalence climbs even higher [11, 18].

#### Female and male sex workers

An estimated 20 to 40% of female sex workers began selling sex before the age of 18 [19]. Among young women in Cambodia who engage in sex work, an HIV prevalence of 23% and incidence of 3.6 per 100 person-years was reported, along with high rates of amphetamine-type substance use [20]. The prevalence of sex work is a concern for female, male and transgender youth. In a recent population-based survey in Kenya, 30.9% of females and 20.9% of males aged 18 to 24 reported a history of sex work [21]. In an HIV-prevention intervention study among young, male sex workers in Mexico City, the investigators found a baseline HIV prevalence rate of 38% [22].

#### Young women in Eastern and Southern Africa

Regions with the highest numbers of HIV-positive adolescents are sub-Saharan Africa and South Asia. Of the 2.1 million adolescents (11 to 19 years) infected with HIV, about 1.3 million (62%) live in Eastern and Southern Africa. Girls and young women between 15 and 30 years old have an extraordinarily high incidence, particularly in countries such as South Africa [23–25]. The most recent household survey confirms the feminization of the epidemic nationally, with adolescent girls 15 to 19 years of age four times more likely to be infected than their male counterparts [25]. In this supplement Karim and colleagues make a compelling case for considering young women in sub-Saharan Africa a key population that urgently requires attention and intervention [26].

The Collaborative Initiative for Paediatric Education and Research (CIPHER) has sponsored this supplement of the journal to highlight where we continue to fall short in our response to adolescent and YKPs, to identify gaps in our understanding and to call the world to action on this urgent public health need [27].

## Young key populations: opportunities

**Figure 2:**
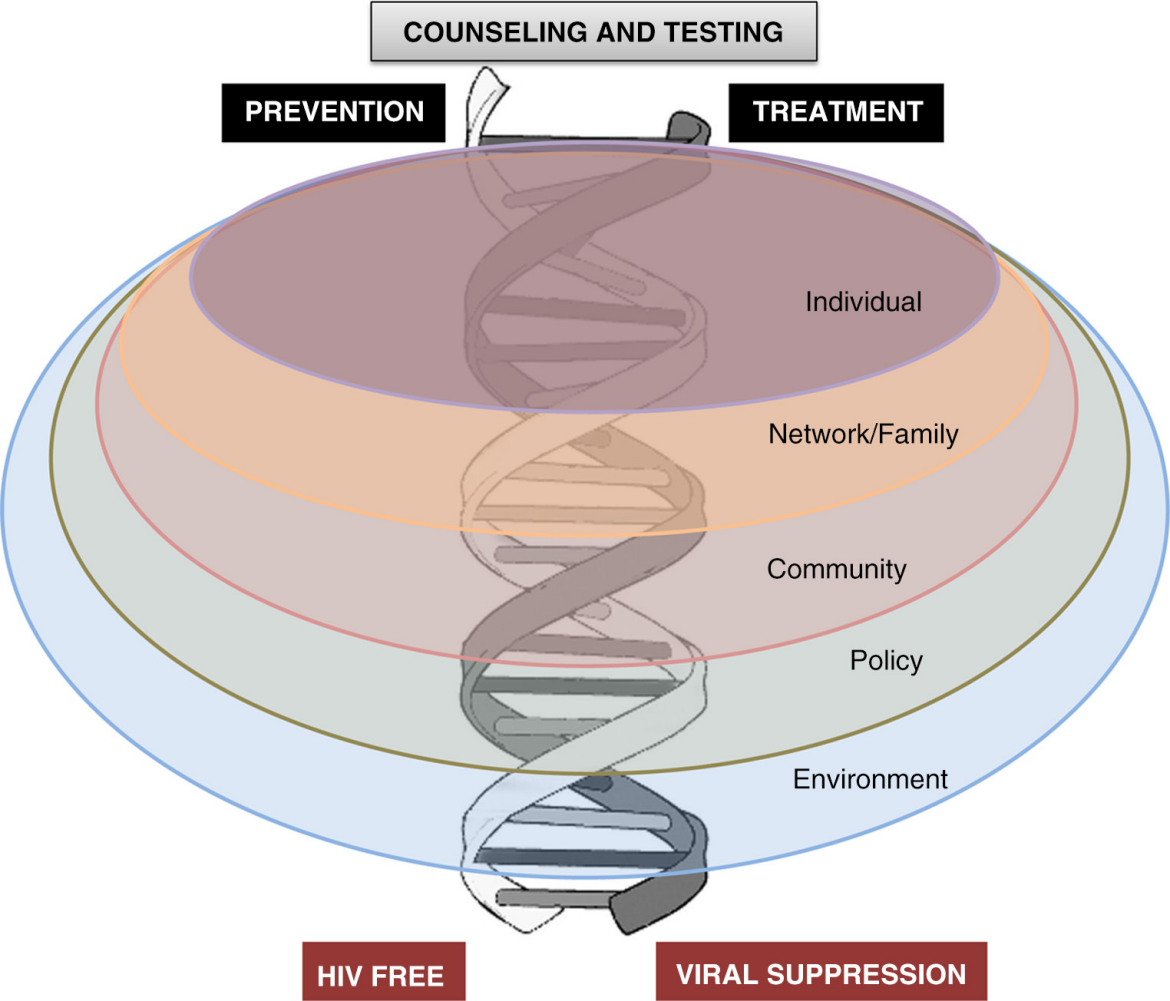
A framework for adolescent service provision. Adapted from DiClemente et al. [29].

The starting point for all HIV programming commences with counselling and HIV testing (HCT) [28]. Thereafter, a number of interventions should occur, either in an HIV-positive adolescent with an ultimate goal of viral suppression (positive/treatment cascade) or in an HIV-negative adolescent to enhance virus-free living (negative/prevention cascade). Adolescents live and interact within families, sexual and social networks and communities, and are affected by society, policies and broader environments and epidemic settings [29]. The positive and negative cascades should occur on this socio-ecological backdrop. The assumption in this model is that interventions will be built around “adolescence” as a common factor and that adolescence gives shared opportunities for interventions at individual, network and community levels (Figure 2).

**Figure 3:**
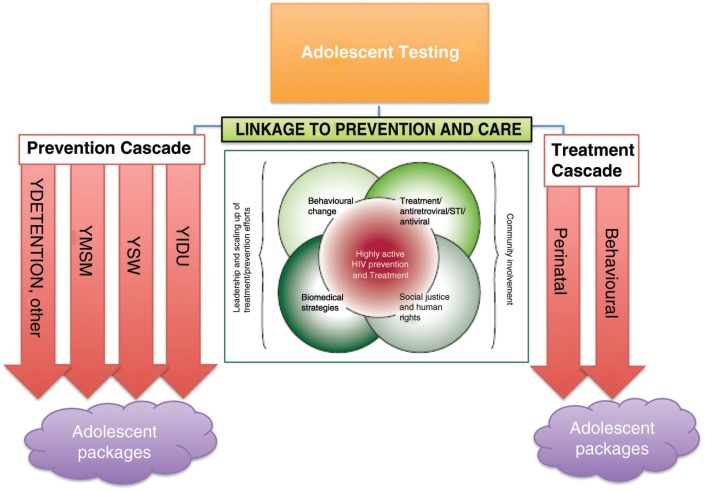
A framework for tailored adolescent key population service provision. Adapted from Coates et al. [31].

Following the recent “prevention revolution,” there has been a call for greater focus on tailored combination HIV prevention (primary and secondary) for adolescents, incorporating structural, biomedical and behavioural interventions within a rights and privacy framework [30, 31]. Pettifor and colleagues have set out a comprehensive review of some of the potential prevention interventions available to YKPs [32]. The double helix cascade can be further developed to embrace a tailored approach (Figure 3).

### HIV testing and linkage

Among youth aged 15 to 19 in Eastern and Southern Africa, only 29% of girls and 20% of boys had ever tested for HIV and received their results [33]. Gaps exist in our understanding of the behavioural and structural barriers to HIV testing and subsequent linkages into either HIV prevention or treatment [30]. Innovative ways to encourage testing have shown promise [34], including incentivization, but more evaluation to show efficacy in this age group is required. Increased awareness among care providers and policy makers is critical, and provider-initiated testing as well as the provision of adolescent-friendly testing services outside of health facilities where youth naturally gather (e.g. home testing, community based, youth centre, club, needle exchange and drop-in site testing) is recommended, as well as the potential for self-testing. A number of countries require parental consent for HIV testing, which can be a significant barrier to testing [35]. Kurth and colleagues in this supplement outline what some of the difficulties are in testing and offer some approaches to adolescent HIV testing and linkage [36].

Following testing, encouraging youth to remain engaged with sexual and reproductive health and other adolescent health services is key. This is to ensure uptake of risk reduction interventions as well as utilization of contraception, primary or secondary HIV, tuberculosis and sexually transmitted infection (STI) prevention interventions, needle and syringe exchange and treatments as required. Youth have repeatedly articulated that engagement with health services should be local, integrated, quick, confidential, non-prejudicial, “hassle-free” and free (or inexpensive) [37]. Utilizing venues and activities where adolescents gather offers opportunities for youth-friendly interventions, including male and female condom provision, STI screening and treatment, human papillomavirus vaccination, contraception and risk reduction counselling. Opportunities should be explored to bring these services into places where youth already congregate: schools, institutions of higher learning, after-school clubs, centres and community venues. Youth-related venues including virtual “meeting places” could be used to deliver programmes, messages and health-related services. By exploiting the commonality of adolescence and group norms, comprehensive services that meet the needs of the adolescent regardless of positive or negative status can be offered with subsequent reduction in stigma, a sense of shared experiences, peer support and health system efficiencies.

### Positive cascade

There are an estimated 2.1 million adolescents aged 10 to 19 living with HIV in the world today [38]. Failure to fully anticipate this situation has resulted in inadequate care and support for this group, requiring rapid redress. Yet little is understood regarding adolescents’ specific healthcare requirements within this context. Healthcare provision specific to adolescents is largely unprecedented worldwide [39]. Whereas the most pressing issue is the requirement for access to antiretroviral therapy, appropriate and effective intervention requires a biopsychosocial approach in order to attend to both the physical and psychological needs of the adolescent, with consideration for the socio-economic context in which treatment is occurring [29]. In addition, specific needs exist among different YKPs. Khairuddin and colleagues in their paper in this supplement have reviewed the evidence for adherence and retention in programmes of young drug users. Krug and colleagues, on the other hand, bring the voice of young people to the supplement in their paper [40, 41].

Research suggests the adolescent developmental phase poses particular challenges: perinatally infected adolescents may experience puberty later; neurocognitive delays with associated behavioural issues may occur; and HIV or its treatment may predispose adolescents to mental health problems [42, 43]. Social context is also significant. Many HIV-positive adolescents will have experienced the death of one or both parents and are likely to have been subjected to stigmatization as a result of household illness [44]. Additional poverty-related challenges exist as well [45]. For those who are behaviourally infected, adolescence may be a particularly difficult time to cope with an HIV diagnosis and, without appropriate support; adolescents may not link into care effectively [39, 46]. In terms of care provision, numerous unmet needs exist including adherence support, mental health assessment and intervention and sexual health, family planning and secondary prevention [47–49]. The need to more effectively link YKPs to service is addressed comprehensively by Delany and colleagues [50]. Matumba and Harper explore more extensively the mental health needs of adolescents in care [51].

### Negative cascade

Historically, adolescent HIV-prevention interventions have targeted individual behaviour change, but impacting biological endpoints, such as HIV incidence through such approaches, has remained elusive. Many would argue unsurprisingly with powerful external socio-economic drivers at play. Consequently, interventions aiming to address some of these structural drivers have shown somewhat more promise. Stepping Stones, a programme targeting gender inequality in relationships through participatory learning approaches, was able to show a significantly reduced incidence of HSV-2 and self-reported intimate partner violence in participants aged 15 to 26 years, although HIV incidence did not change significantly [52].

There has also been a growing focus on the efficacy of structural interventions involving cash transfers and incentives. Cash transfer may operate on at least two levels: conditional on safer sex practices as “contingency management,” or as a way to reduce economic vulnerability, thereby encouraging behaviours with social benefits [53]. In a prospective observational study, Cluver *et al*. conducted interviews with over 3000 adolescents (10 to 18 years) and found an association between household receipt of government cash transfers and reduced incidence of transactional and transgenerational sex (but not other risk behaviours) at one year follow-up in adolescent girls (but not boys), suggesting that this intervention works through removing or reducing those risks taken out of economic need [54]. In the Zomba cash transfer trial in Malawi, adolescent girls who received transfer money were less likely to have older sexual partners and had less frequent sex, resulting in lower rates of HIV infection [55]. In the RESPECT study, beneficiaries were given rewards every four months for remaining free of curable STIs [56]. After one year, the study recorded a 25% drop in STI incidence. Currently, two randomized controlled trials are underway in South Africa to determine the impact of incentivizing school attendance on HIV incidence and sexual behaviour and school attendance, improved academic performance and HIV testing on HIV incidence and sexual behaviour in adolescent girls [57, 58].

Whereas structural interventions are necessary in addressing the distal drivers of the adolescent HIV epidemic, their impact tends to be long-term and difficult to ascertain accurately. In contrast, biomedical interventions are able to target biological endpoints directly. Recently, biomedical HIV prevention has shown a number of successes, including evidence of the effectiveness of male circumcision and oral and topical pre-exposure prophylaxis (PrEP) [59]. This provides ample opportunity for utilization with adolescents, although with the caveat that research is needed to address issues of safety, acceptability, preference and adherence specific to this age group, an area that has thus far largely been neglected [59]. Pettifor and colleagues raise some of the logistical and psychosocial considerations in the application of combination prevention, including biomedical intervention [32]. Conner addresses the legal aspects of service provision to a young drug-using community and outline the responsibilities and complexities that health care providers face in doing so [60].

## Discussion

There are vulnerabilities to communicable diseases that youth in adolescent transition share as a result of the onset of increasing socialization. However, there is also an array of opportunities that present as a result of this increasing tendency to socially congregate. In addition, the evolving capacities of adolescents afford opportunities for uptake of innovation and demand creation. At the end of 2013, there were 1.2 billion Facebook users in the world and 82% of them were between the ages of 18 and 35 [61]. Half of these utilized this technology daily, most before getting out of bed in the morning! Given that adolescents are the one population worldwide that are not seeing a decrease in new HIV cases nor HIV-related mortality, new approaches to both prevention and treatment programming are urgently called for.

Throughout this supplement, we have suggested that our approach should be adolescent-centred rather than issue-centred and should take into account lifestyles, common venues of adolescents and a community-based model rather than conforming to the rigid medicalized, health facility-based model. In addition, we suggest that HIV testing becomes an entry point to an intertwined treatment and prevention cascade with numerous opportunities to provide comprehensive, peer-guided, youth-friendly, “one-stop shop” services in a diverse array of community based settings (Figure 2). Social media and other innovations may inform, create demand and help monitor uptake and use of services [62]. These approaches are endorsed in guidelines for services for YKP emanating from the World Health Organization and are described by Armstrong and colleagues in their contribution to the supplement [63].

In East Africa, 49% of the almost 350,000 medical male circumcisions performed between 2008 and 2011 were in young men aged 15 to 19 [64]. Although the idea is yet to be tested, youth may be just the population who need, understand and take up novel biomedical interventions such as topical and systemic PrEP, harm reduction and other interventions. These discreet, user-controlled methods may be an excellent intervention to tide youth over the difficult transition of sexual debut, experimentation and unbalanced sexual relationships that occur during this time. Mathematical models have suggested that, over the long term, it is more efficient to promote HIV prevention programmes in adolescents than in other age groups [65, 66]. There are a number of reasons for this conclusion: Firstly, adolescents have high HIV prevalence relative to other age groups [67, 68]. Secondly, in the absence of adolescent-focused interventions, adolescents tend to be relatively disadvantaged in their access to prevention services, because adolescent sexual activity is often covert and adolescents prefer not to attend health facilities [37]. Adolescent-focused interventions are needed to remedy the existing inequalities in access to prevention services. A third reason why it is more efficient to focus prevention efforts on adolescents is that individuals who acquire HIV at younger ages have greater future potential to transmit HIV than individuals who acquire HIV at older ages. Because HIV risk behaviour is generally highest at young ages and decreases as individuals enter into long-term relationships and age, individuals who become infected at young ages have more high-risk transmission potential ahead of them [69, 70]. In addition, individuals who have high propensity for sexual and other risk behaviours tend to become infected at younger ages than individuals who have lower propensity for risk behaviours [71].

It is therefore important to focus HIV primary and secondary prevention efforts in adolescents, not only because they are at high risk of acquiring HIV, but also because they have a high risk of transmitting HIV to others. The latter point has often been overlooked in the HIV-prevention literature. Adolescents need to be considered as a “core group” in the same way as other high risk groups such as sex workers and their clients, in the development of HIV, tuberculosis and STI prevention strategies. For this reason, testing, linkage to care and earlier treatment with viral suppression both for personal health and to reduce onward transmission are urgent goals for every Adolescents Living with HIV (ALWH) [72].

The goal of zero infections, zero discrimination and zero deaths in the adolescent population for HIV is a goal within our reach and a very important one to attain, not only because efficiencies and impact on the broader epidemics require this, but also because the youth of today represent our collective hope for the future.

Young people should be at the forefront of global change and innovation. Empowered, they can be key agents for development and peace. If, however, they are left on society's margins, all of us will be impoverished. Let us ensure that all young people have every opportunity to participate fully in the lives of their societies.

—Kofi Annan

## References

[1] Center for Strategic & International Studies (2014). The global youth wellbeing index [Internet].

[2] World Health Organization (WHO) (2014). Health for the world's adolescents: a second chance in the second decade.

[3] Fact sheet on adolescent health (2014). http://www.unaids.org/en/media/unaids/contentassets/documents/factsheet/2012/20120417_FS_adolescentsyoungpeoplehiv_en.pdf.

[4] Joint United Nations Programme on HIV/AIDS (UNAIDS) (2014). The gap report – July 2014.

[5] United Nations Children's Fund (2013). Towards an AIDS-free generation – children and aids: sixth stocktaking report, 2013.

[6] YKPs guidelines (2014). Consolidated guidelines on HIV prevention, diagnosis, treatment and care for key populations.

[7] UNAIDS (2014). Global AIDS response progress reporting.

[8] Centers for Disease Control and Prevention (CDC) (2014). HIV among youth fact sheet.

[9] McKinnon LR, Gakii G, Juno JA, Izulla P, Munyao J, Ireri N (2014). High HIV risk in a cohort of male sex workers from Nairobi, Kenya. Sex Transm Infect.

[10] Baral SD, Poteat T, Strömdahl S, Wirtz AL, Guadamuz TE, Beyrer C (2013). Worldwide burden of HIV in transgender women: a systematic review and meta analysis. Lancet Infect Dis.

[11] Baral S, Todd CS, Aumakhan B, Lloyd J, Delegchoimbol A, Sabin K (2013). HIV among female sex workers in the Central Asian Republics, Afghanistan, and Mongolia: contexts and convergence with drug use. Drug Alcohol Depend.

[12] Wilson EC, Garofalo R, Harris RD, Herrick A, Martinez M, Martinez J (2009). Transgender female youth and sex work: HIV risk and a comparison of life factors related to engagement in sex work. AIDS Behav.

[13] United Nations General Assembly: country report on the follow up to the declaration of commitment on HIV/AIDS Reporting period 2006–2007.

[14] Altaf A, Zahidie A, Agha A (2012). Comparing risk factors of HIV among *hijra* sex workers in Larkana and other cities of Pakistan: an analytical cross sectional study. BMC Public Health.

[15] Garofalo R, Deleon J, Osmer E, Doll M, Harper GW (2006). Overlooked, misunderstood and at-risk: exploring the lives and HIV risk of ethnic minority male-to-female transgender youth. J Adolesc Health.

[16] UNAIDS (2013). Global report: UNAIDS report on the global AIDS epidemic 2013.

[17] Vitek CR, Čakalo JI, Kruglov YV, Dumchev KV, Salyuk TO, Božičević I (2014). Slowing of the HIV epidemic in Ukraine: evidence from case reporting and key population surveys, 2005–2012. PLoS One.

[18] Hoffman BR (2014). The interaction of drug use, sex work, and HIV among transgender women. Subst Use Misuse.

[19] Silverman JG (2011). Adolescent female sex workers: invisibility, violence and HIV. Arch Dis Child.

[20] Couture MC, Sansothy N, Sapphon V, Phal S, Sichan K, Stein E (2011). Young women engaged in sex work in Phnom Penh, Cambodia, have high incidence of HIV and sexually transmitted infections, and amphetamine-type stimulant use: new challenges to HIV prevention and risk. Sex Transm Dis.

[21] Githuka G, Hladik W, Mwalili S, Cherutich P, Muthui M, Gitonga J (2014). Populations at increased risk for HIV infection in Kenya: results from a national population-based household survey, 2012. J Acquir Immune Defic Syndr.

[22] Galárraga O, Sosa-Rubí SG, González A, Badial-Hernández F, Conde-Glez CJ, Juárez-Figueroa L (2014). The disproportionate burden of HIV and STIs among male sex workers in Mexico City and the rationale for economic incentives to reduce risks. J Int AIDS Soc.

[23] Jaspan HB, Berwick JR, Myer L, Mathews C, Flisher AJ, Wood R (2006). Adolescent HIV prevalence, sexual risk, and willingness to participate in HIV vaccine trials. J Adolesc Health.

[24] Dunkle KL, Jewkes R, Nduna M, Jama N, Levin J, Sikweyiya Y (2007). Transactional sex with casual and main partners among young South African men in the rural Eastern Cape: prevalence, predictors, and associations with gender-based violence. Soc Sci Med.

[25] Pettifor AE, Measham DM, Rees HV, Padian NS (2004). Sexual power and HIV risk, South Africa. Emerg Infect Dis.

[26] Dellar RC, Dlamini S, Karim QA (2015). Adolescent girls and young women: key populations for HIV epidemic control. J Int AIDS Soc.

[27] International AIDS Society Collaborative Initiative for Paediatric HIV Education and Research (CIPHER) [Internet].

[28] WHO HIV and adolescents: guidance for HIV testing and counselling and care for adolescents living with HIV.

[29] DiClemente RJ, Salazar LF, Crosby RA, Rosenthal SL (2005). Prevention and control of sexually transmitted infections among adolescents: the importance of a socio-ecological perspective – a commentary. Public Health.

[30] Pettifor A, Bekker L-G, Hosek S, DiClemente R, Rosenberg M, Bull S (2013). for the HIV Prevention Trials Network (HPTN) Adolescent Scientific Committee (2013). Preventing HIV among young people: research priorities for the Future. J Acquir Immune Defic Syndr.

[31] Coates T, Richter L, Caceres C (2008). Behavioural strategies to reduce HIV transmission: how to make them work better. Lancet.

[32] Pettifor A, Nguyen NL, Celum C, Cowan FM, Go V, Hightow-Weidman L (2015). Tailored combination prevention packages and PrEP for young key populations. J Int AIDS Soc.

[33] UNAIDS/Programme Coordinating Board (2013). HIV, adolescents and youth.

[34] Black S, Wallace M, Middelkoop K, Robbertze D, Bennie T, Wood R (2014). Improving HIV testing amongst adolescents through an integrated Youth Centre rewards program: insights from South Africa. Child Youth Serv Rev.

[35] American Academy of Pediatrics Policy statement: adolescents and HIV infection: the pediatrician's role in promoting routine testing [Internet].

[36] Kurth AE, Lally MA, Choko AT, Inwani IW, Fortenberry JD (2015). HIV testing and linkage to services for youth. J Int AIDS Soc.

[37] Klein J, Wilson KM (2002). Delivering quality care: adolescents’ discussion of health risks with their providers. J Adolesc Health.

[38] Idele P, Gillespie A, Porth T, Suzuki C, Mahy M, Kasedde S (2014). Epidemiology of HIV and AIDS among adolescents: current status, inequities, and data gaps. J Acquir Immune Defic Syndr.

[39] Jaspan HB, Li R, Johnson L, Bekker LG (2009). The emerging need for adolescent-focused HIV care in South Africa: opinion. South Afr J HIV Med.

[40] Lall P, How LS, Khairuddin N, Khairuddin A (2015). Review: an urgent need for research on factors impacting adherence to and retention in care among HIV-positive youth and adolescents from key populations. J Int AIDS Soc.

[41] Krug A, Hildebrand M, Sun N (2015). ‘We don't need services. We have no problems’: exploring the experiences of young people who inject drugs in accessing harm reduction services. J Int AIDS Soc.

[42] Mellins CA, Brackis-Cott E, Dolezal C, Abrams EJ (2006). Psychiatric disorders in youth with perinatally acquired human immunodeficiency virus infection. Pediatr Infect Dis J.

[43] Brackis-Cott E, Kang E, Dolezal C, Abrams EJ, Mellins CA (2009). The impact of perinatal HIV infection on older school-aged children's and adolescents' receptive language and word recognition skills. AIDS Patient Care STDS.

[44] Cluver L, Gardner F, Operario D (2009). Poverty and psychological health among AIDS-orphaned children in Cape Town, South Africa. AIDS Care.

[45] Gillespie S, Kadiyala S, Greener R (2007). Is poverty or wealth driving HIV transmission?. AIDS.

[46] Kranzer K, Zeinecker J, Ginsberg P, Orrell C, Kalawe NN, Lawn SD (2010). Linkage to HIV care and antiretroviral therapy in Cape Town, South Africa. PLoS One.

[47] Nglazi MD, Kranzer K, Holele P, Kaplan R, Mark D, Jaspan H (2012). Treatment outcomes in HIV-infected adolescents attending a community-based antiretroviral therapy clinic in South Africa. BMC Infect Dis.

[48] Nachega JB, Hislop M, Nguyen H, Dowdy DW, Chaisson RE, Regensberg L (2009). Antiretroviral therapy adherence, virologic and immunologic outcomes in adolescents compared with adults in southern Africa. J Acquir Immune Defic Syndr.

[49] Kancheva Landolt N (2011). Sexual life and contraception in people living with HIV. Asian Biomed.

[50] Delany-Moretlwe S, Cowan FM, Busza J, Bolton-Moore C, Kelley K, Fairlie L (2015). Providing comprehensive health services for young key populations: needs, barriers and gaps. J Int AIDS Soc.

[51] Mathumba M, Harper GW (2015). Mental health and support among young key populations: an ecological approach to understanding and intervention. J Int AIDS Soc.

[52] Jewkes R, Nduna M, Levin J, Jama N, Dunkle K, Puren A (2008). Impact of stepping stones on incidence of HIV and HSV-2 and sexual behaviour in rural South Africa: cluster randomised controlled trial. BMJ.

[53] Heise L, Lutz B, Ranganthan M, Watts C (2013). Cash transfers for HIV prevention: considering their potential. J Int AIDS Soc.

[54] Cluver L, Boyes M, Orkin M, Pantelic M, Molwena T, Sherr L (2013). Child-focused state cash transfers and adolescent risk of HIV infection in South Africa: a propensity-score-matched case-control study. Lancet Glob Health.

[55] Baird S, Garfein R, McIntosh C, Ozler B (2012). Effect of cash transfer program for schooling on prevalence of HIV and herpes simplex type 2 in Malawi: a cluster randomised trial. Lancet.

[56] de Walque D, Dow WH, Nathan R, Abdul R, Abilahi F, Gong E (2012). Incentivising safe sex: a randomised trial of conditional cash transfers for HIV and sexually transmitted infection prevention in rural Tanzania. BMJ Open.

[57] Pettifor A, MacPhail C, Nguyen N, Rosenberg M (2012). Can money prevent the spread of HIV? A review of cash payments for HIV prevention. AIDS Behav.

[58] Karim QA (2012). A proof of concept cluster randomised controlled trial to evaluate the impact of a cash incentivised prevention intervention to reduce HIV infection in high school learners in rural KwaZulu-Natal, South Africa [Internet].

[59] Padian NS, McCoy SI, Abdool Karim SS, Hasen N, Kim J, Bartos M (2011). HIV prevention transformed: the new prevention research agenda. Lancet.

[60] Conner B (2015). “First, do no harm”: legal guidelines for health programmes affecting adolescents aged 10–17 who sell sex or inject drugs. J Int AIDS Soc.

[61] Bekker LG Teen to grown-up: falling between the cracks or lost in the crowd.

[62] The Associated Press How Facebook has grown: number of active users at Facebook over the years [Internet].

[63] Baggaley R, Armstrong A, Dodd Z, Ngoksin E, Krug A (2015). Young key populations and HIV: a special emphasis and consideration in the new WHO Consolidated guidelines on HIV prevention, diagnosis. treatment and care for key populations.

[64] Galbraith JS, Ochieng A, Mwalili S, Emusu D, Mwandi Z, Kim AA (2014). Status of voluntary medical male circumcision in Kenya: findings from 2 nationally representative surveys in Kenya, 2007 and 2012. J Acquir Immune Defic Syndr.

[65] White RG, Glynn JR, Orroth KK, Freeman EE, Bakker R, Weiss HA (2008). Male circumcision for HIV prevention in sub-Saharan Africa: who, what and when?. AIDS.

[66] Johnson LF, Bekker LG, Dorrington RE (2007). HIV/AIDS vaccination in adolescents would be efficient and practical when vaccine supplies are limited. Vaccine.

[67] Hallett TB, Stover J, Mishra V, Ghys PD, Gregson S, Boerma T (2010). Estimates of HIV incidence from household-based prevalence surveys. AIDS.

[68] Tanser F, Bärnighausen T, Grapsa E, Zaidi J, Newell M-L (2013). High coverage of ART associated with decline in risk of HIV acquisition in rural KwaZulu-Natal, South Africa. Science.

[69] Bongaarts J (2007). Late marriage and the HIV epidemic in sub-Saharan Africa. Popul Stud (Camb).

[70] Caraël M, Cleland J, Ferry B (1995). Sexual behaviour. Sexual behaviour and AIDS in the developing world.

[71] Bershteyn A, Klein DJ, Eckhoff PA (2013). Age-dependent partnering and the HIV transmission chain: a microsimulation analysis. J R Soc Interface.

[72] Bekker LG, Beyrer C, Quinn TC (2012). Behavioral and biomedical combination strategies for HIV prevention. Cold Spring Harb Perspect Med.

