# Supplementary material for: HIV and adolescents: focus on young key populations
Source: J Int AIDS Soc. 2015 Feb 26;18(2Suppl 1):20076. doi: 10.7448/IAS.18.2.20076 (PMC4581085; doi:10.7448/IAS.18.2.20076)
Supplement: Related texts

# Sub article: Building our youth for the future

**DOI:** 10.7448/IAS.18.2.20027

**Published:** 2015-02-26

**Authors:** Linda-Gail Bekker, Leigh Johnson, Melissa Wallace, Sybil Hosek

**Affiliations:** 1 The Desmond Tutu HIV Centre, Institute of Infectious Disease and Molecular Medicine, University of Cape Town, Cape Town, South Africa; 2 Department of Medicine, University of Cape Town, Cape Town, South Africa; 3 Centre for Infectious Disease Epidemiology and Research, University of Cape Town, Cape Town, South Africa; 4 Department of Psychiatry, Stroger Hospital of Cook County, Chicago, IL, USA

**Keywords:** young key populations, HIV, Prevention and treatment

## Abstract

Adolescents and young adults are at increased risk for HIV due to the many developmental, psychological, social, and structural transitions that converge in this period of the lifespan. In addition, adolescent deaths resulting from HIV continue to rise despite declines in other age groups. There are also young key populations (YKPs) that bear disproportionate burdens of HIV and are the most vulnerable, including young men who have sex with men (MSM), transgender youth, young people who inject drugs, and adolescent and young adult sex workers. As a society, we must do more to stop new HIV infections and untimely HIV-related deaths through both primary and secondary prevention and better management approaches. Using an interwoven prevention and treatment cascade approach, the starting point for all interventions must be HIV counselling and testing. Subsequent interventions for both HIV-negative and HIV-positive youth must be “adolescent-centred,” occur within the socio-ecological context of young people and take advantage of the innovations and technologies that youth have easily incorporated into their daily lives. In order to achieve the global goals of zero infections, zero discrimination and zero deaths, a sustained focus on HIV research, policy and advocacy for YKPs must occur.

## References

[1] Center for Strategic & International Studies (2014). The global youth wellbeing index [Internet].

[2] World Health Organization (WHO) (2014). Health for the world's adolescents: a second chance in the second decade.

[3] Fact sheet on adolescent health (2014). http://www.unaids.org/en/media/unaids/contentassets/documents/factsheet/2012/20120417_FS_adolescentsyoungpeoplehiv_en.pdf.

[4] Joint United Nations Programme on HIV/AIDS (UNAIDS) (2014). The gap report – July 2014.

[5] United Nations Children's Fund (2013). Towards an AIDS-free generation – children and aids: sixth stocktaking report, 2013.

[6] YKPs guidelines (2014). Consolidated guidelines on HIV prevention, diagnosis, treatment and care for key populations.

[7] UNAIDS (2014). Global AIDS response progress reporting.

[8] Centers for Disease Control and Prevention (CDC) (2014). HIV among youth fact sheet.

[9] McKinnon LR, Gakii G, Juno JA, Izulla P, Munyao J, Ireri N (2014). High HIV risk in a cohort of male sex workers from Nairobi, Kenya. Sex Transm Infect.

[10] Baral SD, Poteat T, Strömdahl S, Wirtz AL, Guadamuz TE, Beyrer C (2013). Worldwide burden of HIV in transgender women: a systematic review and meta analysis. Lancet Infect Dis.

[11] Baral S, Todd CS, Aumakhan B, Lloyd J, Delegchoimbol A, Sabin K (2013). HIV among female sex workers in the Central Asian Republics, Afghanistan, and Mongolia: contexts and convergence with drug use. Drug Alcohol Depend.

[12] Wilson EC, Garofalo R, Harris RD, Herrick A, Martinez M, Martinez J (2009). Transgender female youth and sex work: HIV risk and a comparison of life factors related to engagement in sex work. AIDS Behav.

[13] United Nations General Assembly: country report on the follow up to the declaration of commitment on HIV/AIDS Reporting period 2006–2007.

[14] Altaf A, Zahidie A, Agha A (2012). Comparing risk factors of HIV among *hijra* sex workers in Larkana and other cities of Pakistan: an analytical cross sectional study. BMC Public Health.

[15] Garofalo R, Deleon J, Osmer E, Doll M, Harper GW (2006). Overlooked, misunderstood and at-risk: exploring the lives and HIV risk of ethnic minority male-to-female transgender youth. J Adolesc Health.

[16] UNAIDS (2013). Global report: UNAIDS report on the global AIDS epidemic 2013.

[17] Vitek CR, Čakalo JI, Kruglov YV, Dumchev KV, Salyuk TO, Božičević I (2014). Slowing of the HIV epidemic in Ukraine: evidence from case reporting and key population surveys, 2005–2012. PLoS One.

[18] Hoffman BR (2014). The interaction of drug use, sex work, and HIV among transgender women. Subst Use Misuse.

[19] Silverman JG (2011). Adolescent female sex workers: invisibility, violence and HIV. Arch Dis Child.

[20] Couture MC, Sansothy N, Sapphon V, Phal S, Sichan K, Stein E (2011). Young women engaged in sex work in Phnom Penh, Cambodia, have high incidence of HIV and sexually transmitted infections, and amphetamine-type stimulant use: new challenges to HIV prevention and risk. Sex Transm Dis.

[21] Githuka G, Hladik W, Mwalili S, Cherutich P, Muthui M, Gitonga J (2014). Populations at increased risk for HIV infection in Kenya: results from a national population-based household survey, 2012. J Acquir Immune Defic Syndr.

[22] Galárraga O, Sosa-Rubí SG, González A, Badial-Hernández F, Conde-Glez CJ, Juárez-Figueroa L (2014). The disproportionate burden of HIV and STIs among male sex workers in Mexico City and the rationale for economic incentives to reduce risks. J Int AIDS Soc.

[23] Jaspan HB, Berwick JR, Myer L, Mathews C, Flisher AJ, Wood R (2006). Adolescent HIV prevalence, sexual risk, and willingness to participate in HIV vaccine trials. J Adolesc Health.

[24] Dunkle KL, Jewkes R, Nduna M, Jama N, Levin J, Sikweyiya Y (2007). Transactional sex with casual and main partners among young South African men in the rural Eastern Cape: prevalence, predictors, and associations with gender-based violence. Soc Sci Med.

[25] Pettifor AE, Measham DM, Rees HV, Padian NS (2004). Sexual power and HIV risk, South Africa. Emerg Infect Dis.

[26] Dellar RC, Dlamini S, Karim QA (2015). Adolescent girls and young women: key populations for HIV epidemic control. J Int AIDS Soc.

[27] International AIDS Society Collaborative Initiative for Paediatric HIV Education and Research (CIPHER) [Internet].

[28] WHO HIV and adolescents: guidance for HIV testing and counselling and care for adolescents living with HIV.

[29] DiClemente RJ, Salazar LF, Crosby RA, Rosenthal SL (2005). Prevention and control of sexually transmitted infections among adolescents: the importance of a socio-ecological perspective – a commentary. Public Health.

[30] Pettifor A, Bekker L-G, Hosek S, DiClemente R, Rosenberg M, Bull S (2013). for the HIV Prevention Trials Network (HPTN) Adolescent Scientific Committee (2013). Preventing HIV among young people: research priorities for the Future. J Acquir Immune Defic Syndr.

[31] Coates T, Richter L, Caceres C (2008). Behavioural strategies to reduce HIV transmission: how to make them work better. Lancet.

[32] Pettifor A, Nguyen NL, Celum C, Cowan FM, Go V, Hightow-Weidman L (2015). Tailored combination prevention packages and PrEP for young key populations. J Int AIDS Soc.

[33] UNAIDS/Programme Coordinating Board (2013). HIV, adolescents and youth.

[34] Black S, Wallace M, Middelkoop K, Robbertze D, Bennie T, Wood R (2014). Improving HIV testing amongst adolescents through an integrated Youth Centre rewards program: insights from South Africa. Child Youth Serv Rev.

[35] American Academy of Pediatrics Policy statement: adolescents and HIV infection: the pediatrician's role in promoting routine testing [Internet].

[36] Kurth AE, Lally MA, Choko AT, Inwani IW, Fortenberry JD (2015). HIV testing and linkage to services for youth. J Int AIDS Soc.

[37] Klein J, Wilson KM (2002). Delivering quality care: adolescents’ discussion of health risks with their providers. J Adolesc Health.

[38] Idele P, Gillespie A, Porth T, Suzuki C, Mahy M, Kasedde S (2014). Epidemiology of HIV and AIDS among adolescents: current status, inequities, and data gaps. J Acquir Immune Defic Syndr.

[39] Jaspan HB, Li R, Johnson L, Bekker LG (2009). The emerging need for adolescent-focused HIV care in South Africa: opinion. South Afr J HIV Med.

[40] Lall P, How LS, Khairuddin N, Khairuddin A (2015). Review: an urgent need for research on factors impacting adherence to and retention in care among HIV-positive youth and adolescents from key populations. J Int AIDS Soc.

[41] Krug A, Hildebrand M, Sun N (2015). ‘We don't need services. We have no problems’: exploring the experiences of young people who inject drugs in accessing harm reduction services. J Int AIDS Soc.

[42] Mellins CA, Brackis-Cott E, Dolezal C, Abrams EJ (2006). Psychiatric disorders in youth with perinatally acquired human immunodeficiency virus infection. Pediatr Infect Dis J.

[43] Brackis-Cott E, Kang E, Dolezal C, Abrams EJ, Mellins CA (2009). The impact of perinatal HIV infection on older school-aged children's and adolescents' receptive language and word recognition skills. AIDS Patient Care STDS.

[44] Cluver L, Gardner F, Operario D (2009). Poverty and psychological health among AIDS-orphaned children in Cape Town, South Africa. AIDS Care.

[45] Gillespie S, Kadiyala S, Greener R (2007). Is poverty or wealth driving HIV transmission?. AIDS.

[46] Kranzer K, Zeinecker J, Ginsberg P, Orrell C, Kalawe NN, Lawn SD (2010). Linkage to HIV care and antiretroviral therapy in Cape Town, South Africa. PLoS One.

[47] Nglazi MD, Kranzer K, Holele P, Kaplan R, Mark D, Jaspan H (2012). Treatment outcomes in HIV-infected adolescents attending a community-based antiretroviral therapy clinic in South Africa. BMC Infect Dis.

[48] Nachega JB, Hislop M, Nguyen H, Dowdy DW, Chaisson RE, Regensberg L (2009). Antiretroviral therapy adherence, virologic and immunologic outcomes in adolescents compared with adults in southern Africa. J Acquir Immune Defic Syndr.

[49] Kancheva Landolt N (2011). Sexual life and contraception in people living with HIV. Asian Biomed.

[50] Delany-Moretlwe S, Cowan FM, Busza J, Bolton-Moore C, Kelley K, Fairlie L (2015). Providing comprehensive health services for young key populations: needs, barriers and gaps. J Int AIDS Soc.

[51] Mathumba M, Harper GW (2015). Mental health and support among young key populations: an ecological approach to understanding and intervention. J Int AIDS Soc.

[52] Jewkes R, Nduna M, Levin J, Jama N, Dunkle K, Puren A (2008). Impact of stepping stones on incidence of HIV and HSV-2 and sexual behaviour in rural South Africa: cluster randomised controlled trial. BMJ.

[53] Heise L, Lutz B, Ranganthan M, Watts C (2013). Cash transfers for HIV prevention: considering their potential. J Int AIDS Soc.

[54] Cluver L, Boyes M, Orkin M, Pantelic M, Molwena T, Sherr L (2013). Child-focused state cash transfers and adolescent risk of HIV infection in South Africa: a propensity-score-matched case-control study. Lancet Glob Health.

[55] Baird S, Garfein R, McIntosh C, Ozler B (2012). Effect of cash transfer program for schooling on prevalence of HIV and herpes simplex type 2 in Malawi: a cluster randomised trial. Lancet.

[56] de Walque D, Dow WH, Nathan R, Abdul R, Abilahi F, Gong E (2012). Incentivising safe sex: a randomised trial of conditional cash transfers for HIV and sexually transmitted infection prevention in rural Tanzania. BMJ Open.

[57] Pettifor A, MacPhail C, Nguyen N, Rosenberg M (2012). Can money prevent the spread of HIV? A review of cash payments for HIV prevention. AIDS Behav.

[58] Karim QA (2012). A proof of concept cluster randomised controlled trial to evaluate the impact of a cash incentivised prevention intervention to reduce HIV infection in high school learners in rural KwaZulu-Natal, South Africa [Internet].

[59] Padian NS, McCoy SI, Abdool Karim SS, Hasen N, Kim J, Bartos M (2011). HIV prevention transformed: the new prevention research agenda. Lancet.

[60] Conner B (2015). “First, do no harm”: legal guidelines for health programmes affecting adolescents aged 10–17 who sell sex or inject drugs. J Int AIDS Soc.

[61] Bekker LG Teen to grown-up: falling between the cracks or lost in the crowd.

[62] The Associated Press How Facebook has grown: number of active users at Facebook over the years [Internet].

[63] Baggaley R, Armstrong A, Dodd Z, Ngoksin E, Krug A (2015). Young key populations and HIV: a special emphasis and consideration in the new WHO Consolidated guidelines on HIV prevention, diagnosis. treatment and care for key populations.

[64] Galbraith JS, Ochieng A, Mwalili S, Emusu D, Mwandi Z, Kim AA (2014). Status of voluntary medical male circumcision in Kenya: findings from 2 nationally representative surveys in Kenya, 2007 and 2012. J Acquir Immune Defic Syndr.

[65] White RG, Glynn JR, Orroth KK, Freeman EE, Bakker R, Weiss HA (2008). Male circumcision for HIV prevention in sub-Saharan Africa: who, what and when?. AIDS.

[66] Johnson LF, Bekker LG, Dorrington RE (2007). HIV/AIDS vaccination in adolescents would be efficient and practical when vaccine supplies are limited. Vaccine.

[67] Hallett TB, Stover J, Mishra V, Ghys PD, Gregson S, Boerma T (2010). Estimates of HIV incidence from household-based prevalence surveys. AIDS.

[68] Tanser F, Bärnighausen T, Grapsa E, Zaidi J, Newell M-L (2013). High coverage of ART associated with decline in risk of HIV acquisition in rural KwaZulu-Natal, South Africa. Science.

[69] Bongaarts J (2007). Late marriage and the HIV epidemic in sub-Saharan Africa. Popul Stud (Camb).

[70] Caraël M, Cleland J, Ferry B (1995). Sexual behaviour. Sexual behaviour and AIDS in the developing world.

[71] Bershteyn A, Klein DJ, Eckhoff PA (2013). Age-dependent partnering and the HIV transmission chain: a microsimulation analysis. J R Soc Interface.

[72] Bekker LG, Beyrer C, Quinn TC (2012). Behavioral and biomedical combination strategies for HIV prevention. Cold Spring Harb Perspect Med.

# Sub article: Tailored combination prevention packages and PrEP for young key populations

**DOI:** 10.7448/IAS.18.2.19434

**Published:** 2015-02-26

**Authors:** Audrey Pettifor, Nadia L Nguyen, Connie Celum, Frances M Cowan, Vivian Go, Lisa Hightow-Weidman

**Affiliations:** 1 Department of Epidemiology, University of North Carolina at Chapel Hill, Chapel Hill, NC, USA; 2 School of Public Health, University of the Witwatersrand, Johannesburg, South Africa; 3 International Clinical Research Center, University of Washington, Seattle, WA, USA; 4 Centre for Sexual Health and HIV/AIDS Research (CeSHHAR) Zimbabwe, Harare, Zimbabwe; 5 Department of Infection and Population Health, University College London, London, United Kingdom; 6 Department of Health Behavior, University of North Carolina at Chapel Hill, Chapel Hill, NC, USA; 7 Department of Infectious Diseases, University of North Carolina at Chapel Hill, Chapel Hill, NC, USA

**Keywords:** HIV, key populations, combination prevention, pre exposure prophylaxis

## Abstract

**Introduction:**

Young key populations, defined in this article as men who have sex with men, transgender persons, people who sell sex and people who inject drugs, are at particularly high risk for HIV. Due to the often marginalized and sometimes criminalized status of young people who identify as members of key populations, there is a need for HIV prevention packages that account for the unique and challenging circumstances they face. Pre-exposure prophylaxis (PrEP) is likely to become an important element of combination prevention for many young key populations.

**Objective:**

In this paper, we discuss important challenges to HIV prevention among young key populations, identify key components of a tailored combination prevention package for this population and examine the role of PrEP in these prevention packages.

**Methods:**

We conducted a comprehensive review of the evidence to date on prevention strategies, challenges to prevention and combination prevention packages for young key populations. We focused specifically on the role of PrEP in these prevention packages and on young people under the age of 24, and 18 in particular.

**Results and discussion:**

Combination prevention packages that include effective, acceptable and scalable behavioural, structural and biologic interventions are needed for all key populations to prevent new HIV infections. Interventions in these packages should meaningfully involve beneficiaries in the design and implementation of the intervention, and take into account the context in which the intervention is being delivered to thoughtfully address issues of stigma and discrimination. These interventions will likely be most effective if implemented in conjunction with strategies to facilitate an enabling environment, including increasing access to HIV testing and health services for PrEP and other prevention strategies, decriminalizing key populations’ practices, increasing access to prevention and care, reducing stigma and discrimination, and fostering community empowerment. PrEP could offer a highly effective, time-limited primary prevention for young key populations if it is implemented in combination with other programs to increase access to health services and encourage the reliable use of PrEP while at risk of HIV exposure.

**Conclusions:**

Reductions in HIV incidence will only be achieved through the implementation of combinations of interventions that include biomedical and behavioural interventions, as well as components that address social, economic and other structural factors that influence HIV prevention and transmission.

## Introduction

Globally young people face a high burden of HIV infection. It is estimated that 39% of new infections occur among adolescents annually, and despite global declines in HIV mortality among adults [1], HIV-related deaths among young people increased by 50% between 2005 and 2012 [2]. Key populations, defined here as men who have sex with men (MSM), transgender persons, sex workers and people who inject drugs (PWID), experience a high burden of HIV infection and incidence rates in both concentrated and generalized epidemic settings. It is estimated that up to 50% of new infections occur among key populations annually [2].

Young people (which we define as persons between 10 and 24) who fall under the umbrella term “key population” are at particularly high risk for HIV and may engage in overlapping risk behaviours, such as injecting drugs and selling sex. While data are scarce on the size of adolescent key populations (defined as ages 10–19 years), in areas of the world where the epidemic is concentrated among key populations, adolescents clearly face an increased burden. It is estimated that 95% of new infections among adolescents in Asia are among key populations (PWID, MSM and sex workers) [3] and that 70% of all individuals who inject drugs are under the age of 25 [4]. A number of studies have documented that many individuals who engage in sex work or injection drug use began before the age of 18 [5, 6]. Among MSM globally, infection rates continue to increase in many settings [7]. HIV incidence data from the United States highlight the crisis of HIV among young MSM (YMSM); from 2008 to 2011, HIV incidence for YMSM aged 13–24 years increased 26% [8]. Due to this increased risk, multiple programmatic calls have been issued to refocus prevention efforts on adolescent and youth key populations. Reductions in HIV incidence will only be achieved through the implementation of combinations of interventions that include biomedical and behavioural interventions, as well as components that address social, economic and other structural factors that influence HIV prevention and transmission [9–15]. Antiretroviral-based prevention, specifically pre-exposure prophylaxis (PrEP), is one biomedical prevention approach that has recently shown great promise in reducing risk of HIV acquisition [16–20]. However, its effectiveness in some adolescent key populations remains unclear.

In this article, we review the current evidence on prevention strategies for young key populations and specific challenges to HIV prevention unique to young key populations, describe what an effective and tailored combination prevention package would look like for young key populations and discuss the role of PrEP as a potential component of that prevention package.

## Methods

We conducted a comprehensive review of the evidence to date on prevention strategies, challenges to prevention and combination prevention packages for young key populations. We focused specifically on the role of PrEP in these prevention packages for young key populations under the age of 24, and under the age of 18 in particular. We examined the published literature by searching PubMED using the following search terms: PrEP, MSM, IDU, PWID, Sex work and HIV prevention. We also examined the works cited of published articles. We identified ongoing studies of PrEP by examining the AVAC database of ongoing and planned PrEP evaluation studies, conference abstracts and the NIH Research Portfolio Online Reporting Tools (RePORT). We did not utilize any exclusion criteria; however, we focused our search on studies or evaluations of PrEP among young [18–24], key populations (MSM, PWID and people who sell sex).

## Results and discussion

### Challenges to HIV prevention among young key populations

Young key populations are at increased risk of HIV infection compared to adults due to cognitive, contextual and structural factors that increase their vulnerability to peer pressure, manipulation and exploitation or abuse by older people [21]. At the same time, young key populations are a heterogeneous group and the risk factors for HIV differ across young key populations and vary by age and setting.

### Young PWID

Young PWID face a number of challenges to HIV prevention. PWID aged 18–29 are more likely to inject daily than other age groups [22], more likely to share syringes than other age groups [22], less likely to use harm reduction and treatment services, more likely to be reliant on older people for access to drugs and injecting equipment, more likely to obtain needles from unofficial sources, and less informed about risks and their rights [23]. Female PWIDs frequently experience violence from intimate partners, police and sex trade clients [24], as well as homelessness [25] and psychiatric co-morbidities [26], which may act synergistically, increasing their risk for HIV infection [23]. Young female PWID in particular may face unique risks for HIV, including mental health disorders [27], and high suicide risk [28]. In addition, young female injectors have higher injecting risk behaviours compared to young male injectors, including multiple sex partners [29] and co-infection with HIV and HCV [30].

Despite existing evidence-based prevention tools for PWID populations, including opioid substitution therapy (OST) [31–34], needle and syringe exchange programs (NSP) [31, 35, 36] and HIV testing and counselling (HTC) [31, 37], the epidemic among PWID continues to accelerate in many settings [38] while the proportion of youth who are PWID continues to increase [39].

### Young MSM and transgender persons

Young MSM experience multiple life stressors and high levels of victimization based on sexual identity that can lead to engagement in higher sexual and drug use activities, and also make practicing HIV prevention strategies challenging [40–42]. Compared to their heterosexual peers, YMSM have been found to have an increased risk of depressive symptoms, anxiety disorders, suicidal ideation and attempts, and PTSD [43–45]. Some YMSM may experience homelessness or unstable housing as a result of being driven out of their family homes. Further, YMSM face additional social challenges in developing a positive self-identity due to stigmatization, discrimination and homophobia. The challenges that place YMSM, and in particular YMSM of colour, at risk for HIV infection also impact their awareness, access to, and adherence to prevention services, including PrEP [46–50]. For instance, despite routine testing recommendations, MSM who are younger (<25 years), black, and/or have low income are less likely to test or be aware that they are HIV-infected [51–55]. These challenges are magnified in areas where homosexuality is criminalized.

Young transgender women are also at extremely high risk of HIV infection due to multiple concurrent risk factors, including substance use, sex work, depression, unstable housing, discrimination, violence and victimization [56–59]. Limited access to gender-sensitive health services can also interfere with HIV prevention efforts.

### Young people who sell sex

Young people who sell sex also face challenges that put them at greater risk of HIV when compared to adult sex workers. These include a heightened risk of physical and sexual violence by clients and law enforcement agents [60–63]. As a result of exploitation by adults, young people who sell sex may lack control over the frequency and location of where they sell sex, and may be more likely to work on the streets than adults [64–67]. Young people who have been orphaned or abandoned by their family face social and economic marginalization; consequently, in many parts of the world, children living on the street sell sex as a survival strategy [68–70]. In addition, young people who sell sex use condoms less consistently than adult sex workers due to lack of access to condoms, poor negotiating skills and limited knowledge of issues related to sexual and reproductive health. Young people who sell sex also face stigma and discrimination, which not only affects their ability to access services but may also lead to low self-worth and self-stigmatization [71]. Young people who sell sex may also be more difficult to reach with services because initiation into sex work may be gradual and thus they may not recognize themselves to be at risk.

### Legal and structural barriers to HIV prevention

Across all young key populations, parental permission laws in many settings poses an additional challenge for delivering effective prevention packages to this age group because they prevent minors from accessing prevention and care services without the involvement of a parent. A recent survey by UNAIDS found that over 33 countries in Africa have age based criteria for HTC [72]. In addition, young people often do not seek health services due to stigma associated with youth attending HIV prevention services, and lack of youth friendliness and confidentiality in many health settings [73]. These structural barriers are even greater for young key populations because their behaviours are stigmatized and illegal in many settings, resulting in discrimination, marginalization, possible legal consequences (such as imprisonment) and fear of punishment [3]. In countries where homosexuality is illegal, YMSM who fear being outed by health workers may delay care. Laws that classify sex work among people who are under 18 as sexual exploitation (designed to protect minors involved in the sex industry), may have the unintended consequence of encouraging young women who sell sex to deny involvement or avoid health services because of fear of being sent to state institutions or suffering abuse and harassment by law enforcement [74–80] . Laws requiring parental permission for prevention services also fail to recognize that many adolescents engaged in injecting drug use or selling sex do not live with family or may be orphans.

### Combination prevention packages for young key populations

Combination prevention packages that include effective, acceptable and scalable behavioural, structural and biologic interventions are needed for all key populations in order to have the greatest impact on preventing new infections. This is supported by mathematical modelling which has found that existing structural and behavioural prevention approaches for key populations could be further strengthened by combining them with newer biomedical prevention interventions, such as PrEP [9–15] . Combination prevention packages should aim to achieve high coverage of HIV testing and knowledge of HIV serostatus, parsimony in selecting evidence-based interventions, synergy such that the effect of a combination of interventions is at least the sum of the parts, if not greater, and intervention coverage, which is a function of access to, utilization of, and high retention (see Table 1) [81]. Based on recent guidelines from the WHO for HIV prevention, diagnosis, treatment and care for key populations, combination prevention packages should also include the key health care sector interventions as summarized in Table 2 and strive to create an enabling environment. Among key populations, interventions that meaningfully involve beneficiaries in the design and implementation of the intervention, and take into account the context in which the intervention is being delivered to thoughtfully address issues of stigma and discrimination are most likely to be most effective.

**Figure 1:** Representation of key populations and young people included in completed PrEP studies. Bangkok and TDF2 participants in “participants <25 category” includes participants under 30 years of age. Number of participants in “participants <25” unknown for US Safety study and VOICE study.

**Table 1:** Principles of combination prevention

Principle	Details
1. High coverage of HIV testing and knowledge of HIV serostatus	HIV testing is the “gateway” to both the HIV treatment and prevention cascades; HIV testing programs need to facilitate linkages to care and prevention
2. Parsimony in selecting evidence-based interventions	Scale, coverage, affordability and impact could be compromised with more complex combination packages
3. Pilot work to determine the acceptability and feasibility of scaling these interventions	Achieve high coverage by prioritizing the subset of the population most at risk of HIV transmission or acquisition
4. Synergy such that the effect of a combination of interventions is at least the sum of the parts, if not greater	Delivering non-overlapping and complimentary interventions to reduce HIV infectiousness and susceptibility
5. Intervention coverage	A function of access to the interventions, willingness of persons prioritized based on risk to utilize the interventions, high retention in the prevention/treatment cascade

**Table 2:** Key components of a comprehensive prevention package

The WHO comprehensive package for prevention			
*Essential health sector interventions*	*IDU*	*Sex workers*	*MSM*
1. Comprehensive condom and lubrication programming	✓	✓	✓
2. Harm reduction interventions for substance use	Needle and syringe programs and opioid substitution therapy		
3. Behavioural interventions	✓	✓	✓
4. HIV testing and counselling	✓	✓	✓
5. HIV treatment and care	✓	✓	✓
6. Sexual and reproductive health interventions	✓	✓	✓
7. Prevention and management of co-infections and other co-morbidities	Viral hepatitis, tuberculosis and mental health conditions	Mental health; substance use	Mental health; substance use
*Essential strategies for an enabling environment*	*Examples*		
1. Supportive legislation, policy and financial commitment	Decriminalization of NSP and OST programs		Social Protection; Decriminalization
2. Addressing stigma and discrimination	✓	✓	✓
3. Community empowerment		✓	
4. Addressing violence against people from key populations	✓	✓	✓
*PrEP plus adherence support*	✓^a^	✓^a^	✓

a Note that the WHO has currently only issued a strong recommendation for PrEP use among MSM. The WHO has made no recommendations regarding PrEP among PWIDs and sex workers but has called for PrEP demonstration projects to assess how to implement PrEP as part of comprehensive risk reduction services in these populations. OST, opioid substitution therapy; NSP, needle and syringe exchange programs.

### PrEP as a potential component of combination prevention packages

PrEP has recently emerged as a promising biomedical intervention to prevent HIV infection [16–20] (see Table 3). For adolescent and young key populations, PrEP could offer a highly effective, time-limited primary prevention if they can access health services and are motivated to use PrEP while at risk of HIV exposure. Although no PrEP efficacy trials completed to date exclusively recruited adolescents and young persons, all the trials included persons between ages 18 and 24 (see Table 4 and Figure 1). Nonetheless, young key population face unique challenges that may influence their willingness to use and adhere to PrEP. Addressing these challenges will be key to the success of PrEP as an intervention strategy in this vulnerable population.

**Table 3:** Completed PrEP studies among key populations and young people

Trial name and location	Number enrolled	Median age (Range)	Study population	Young people/key populations, N (%)	Design and intervention	Percent relative reduction in HIV incidence (95% CI; *p*-value)	Adherence
The Bangkok Tenofovir Study [18] Thailand	2413	31 (20–60)	PWID	Under 30 years old: 1033 (43%) PWID: 2413 (100%) MSM: 91 (5%)	Randomized controlled trial – TDF – Placebo	48.9% (95% CI: 9.6, 72.2%; *p*=0.01)	Drug diaries: 83.8% DOT: 86.9% Blood plasma: TDF detected in 66% in TDF group (overall); TDF detected in 39% among participants who seroconverted; TDF detected in 67% among participants who did not seroconvert
CAPRISA 004 [20] KwaZulu Natal, South Africa	889	23.9 (mean) (18–40)	Women	Under 25 years old: 579 (65.1%) SW: 17 (1.9%)	Randomized controlled trial – TDF vaginal gel (BAT24) – Placebo	39% (95% CI: 6, 60%; *p*=0.017)	Monthly (applicator) count divided by number of sex acts that month: 72.20% (all participants); 61.3% among women who did not seroconvert; 59.2% among women who did seroconvert Blood plasma: 50.5%
FEM-PrEP [82] Kenya, South Africa, Tanzania	2120	23 (18–35)	Women	Under 25: 1213 (57.2%) SW: 268 (12.6%)	Randomized controlled trial – TVD – Placebo	Stopped for futility	Self-report: 95% Pill count: 88% Blood plasma: TVD detected in 26% at beginning of infection window, 21% at end of window and 15% at both visits among women who seroconverted; TVD detected in 35% at beginning of the infection window, 37% of women at end of the window and 24% at both visits among women who did not seroconvert
iPrEx [16] US, Brazil, Peru, Ecuador, Thailand, South Africa	2499	27 (mean) (18–67)	MSM TGW	Under 25: 1153 (46%) TGW: 29 (1%) SW: 1027 (41%)	Randomized controlled trial – TVD – Placebo	44% (95% CI: 15, 63%; *p*=0.005) No significant difference across age	Self-reported pill use: 95% Pill count of unused study product: 89–95% Blood plasma: TVD detected in 9% among participants who seroconverted; TVD was detected in 51% among participants who did not seroconvert
iPrEx OLE [83] Peru, Ecuador, US, Brazil, Thailand, South Africa	1603	28 (mean) (18–40 +)	MSM TGW	Under 25 years old: 317 (20%) MSM: 1603 (100%) TGW: 175 (11%)	Open-label extension – 1225 (76%) received TDF	49% (95% CI: −1, 74%)	Blood plasma: 71% (week 4, 8, or 12)
Partners PrEP [17] Kenya, Uganda	4758	Women: 33 Men: 34 (18–65)	Sero-discordent couples	Under 25: 533 (11%)	Phase 3 study extension/ rollover Randomized controlled trial – TDF – TVD – Placebo	TDF: 67% (95% CI: 44, 81%; *p*<0.001) TVD: 75% (95% CI: 55, 87%; *p*<0.001) No significant difference between women <25 and ≥25	Bottle count: 98% Pill count: 97% Blood plasma: TDF/TVD detected in 31% among participants who seroconverted; TDF/TVD detected in 82% among participants who did not seroconvert
Project PrEPare (ATN 082) [84] US (Baltimore, Boston, Chicago, Denver, Detroit, Houston, Los Angeles, Memphis, Miami, New Orleans, Philadelphia, Tampa)	68	20 (18–22)	Young MSM (focus on MSM of colour)	Under 25: 58 (100%) MSM: 58 (100%) SW: 10 (17.24%)	Feasibility and acceptability study – 3MV (Many Men, Many Voices behavioural HIV intervention) alone – 3MV with TVD (*N*=20) – 3MV with placebo	n/a - Feasibility and acceptability study	Self-reported: 62% (range 43–83%) Blood plasma: 63.2% (week 4), 20% (week 24)
TDF2 (CDC 494) [19] Botswana	1219	25 (18–39)	Men and women (mostly young)	Under 21: 25 (2%) Age 21–29: 1082 (89%)	Randomized controlled trial – TVD – Placebo	62.2% (95% CI: 21.5, 83.4%; *p*=0.03)	Pill count: 84.2% (TVG group) Self-reported 3 days prior: 94.4% (TVD group) Blood plasma: TVD detected in 50% among participants who seroconverted; TVD detected in 81% among participants who did not seroconvert
US Safety study (CDC 4323) [85] US (San Francisco, Atlanta, Boston)	400	39 (18–60)	MSM	Under 25: Unknown MSM: 400 (100%)	Phase II safety study – TDF upon enrolment – Placebo upon enrolment – TDF 9 months after enrolment – Placebo 9 months after enrolment	n/a - Safety study	
VOICE (MTN 003) [86] Uganda, Zimbabwe, South Africa	5029	25.3 (mean) (18–45)	Women	Under 25: unknown	Phase IIb (proof of concept) trial – TVD – TDF – TDF vaginal gel – Placebo (pill) – Placebo (gel)	Stopped for futility	Self-report and pill/applicator count: ~90% Blood plasma: TVD detected in 29% in TVD group (overall); TVD detected in 21% in TVD group (≤25, single); TVD detected in 54% in TVD group (>25, married); TDF detected in 28% in oral TDF group; TDF detected in 23% in TDF gel group
Willingness of PWID to use PrEP in Ukraine [87] Ukraine	128	(16–40+)	PWID	Under 25/PWID: 22 (17% of PWIDs)	Willingness to accept and use PrEP	n/a 53% stated they would “definitely” be willing to use PrEP (based on a 4-point Likert scale) 32.6% stated they would “probably” be willing to use PrEP	n/a

MSM, men who have sex with men; TWG, transgender women; SW, sex workers; PWID, people who inject drugs; TDF, tenofovir; TVD, emtricitabine/tenofovir (FTC/TDF).

**Table 4:** Overview of completed and ongoing PrEP studies targeting young people and key populations, by population and PrEP type/mode of delivery

Target population	Oral PrEP and combination prevention	Dosing/alternative formulations of oral PrEP	Topical PrEP^c^
Under 18 years old	CHAMPS-SA Plus Pills^a^ FACTS 002^a^ Project PrEPare (ATN 113)^a^		
MSM/TGW	California Collaborative Treatment Group Consortium/ALERT (CCTG 593) DemoPrEP The Demo Project (NIAID) East Bay Consortium/CRUSH^b^ HPTN 073 Los Angeles County PATH PrEP Demo Project LVCT and SWOP Project PrEPare (ATN 110)^b^ Project PrEPare (ATN 113)^a^ PROUD Sibanye Health Project SPARK Project NYC Sustainable Health Center Implementation PrEP Pilot Study (SHIPP) (CDC Foundation) VicPrEP Demonstration Project	ADAPT (HPTN 067) IPERGAY NEXT-PREP (HPTN 069/ACTG 5305)	MTN 017
SW	Durbar (DMSC) and Ashodaya Samithi LVCT and SWOP SAPPH-IRe TAPS: Expanded use of ART for treatment and prevention for female sex workers in South Africa Wits Reproductive Health and HIV Institute		
PWID	Bangkok Tenofovir Study Open-Label Extension Sustainable Health Center Implementation PrEP Pilot Study (SHIPP) (CDC Foundation)		

a Participants 18 and younger.

b participants 24 and younger.

c note that there are other efficacy trials of topical PrEP (e.g., FACTS 001, ASPIRE, Ring Study) but they do not exclusively target young people or key populations.

Adherence to medications is known to be a significant challenge for young people, [88–91] and thus adherence to PrEP must be an important focus of any intervention providing PrEP to this population [92]. Across all the PrEP trials, there is robust evidence that PrEP has high effectiveness, but this effectiveness is highly dependent on adherence [11, 93]. Sub-analyses of existing trial data suggest that younger and unmarried participants as well as those with highest behavioural risk were the least likely to adhere to PrEP [17, 20, 94]. These results are in line with evidence from other medical conditions, which have found that between 10 and 90% of adolescents demonstrate inadequate adherence to therapy, and those least likely to adhere are the most vulnerable psychosocially [89, 95, 96]. Notably, all the PrEP trials had a subset of persons who had consistent and sustained use of PrEP, which ranged from 30% in the VOICE [94] and FEM-PrEP [82] trials to 80% in the Partners PrEP Study [17].

Concerns about adherence to PrEP and subsequent drug-resistance are particularly strong for PWID [97], whose barriers to antiretroviral therapy (ART) adherence include interruptions in care due to low social support, incarceration, and compulsory detoxification and detention [98]. At the same time, a recent meta-analysis revealed that PWID had comparable rates of ART adherence to non-drug using populations [98] suggesting that these concerns may be unfounded.

There are limited data on adherence to ART among persons who sell sex [99, 100]. Some reports suggest that persons who sell sex may be poorly adherent due to their social instability, increased mobility and police harassment, but there are also data suggesting that persons who sell sex can adhere if properly supported. However, while we can learn from studies on ART adherence, the barriers to adherence may be quite different among HIV-negative PrEP users [101]. There is a critical need to understand the reasons for poor PrEP adherence among young women, including sex workers [102]. Several upcoming studies and demonstration projects are examining the impact of different adherence counselling programs and delivery mechanisms to improve PrEP adherence among participants (see Table 5, Supplementary files).

The differential uptake and sustained use among populations enrolled in placebo-controlled PrEP efficacy trials in part reflects population differences in terms of levels of uncertainty and ambivalence about using antiretrovirals for prevention, risk perception, concerns about side effects, stigma, reactions of others, partner support, participation in a placebo-controlled trial to obtain access to health care and other services, and concerns about randomization to placebo or a product of uncertain efficacy [103–105] . Uptake and adherence among participants in clinical trials who are randomized to placebo or active product and counselled about unknown efficacy may not predict uptake and adherence among at risk participants who are offered open-label product and counselled about known efficacy and the importance of adherence. Encouragingly, two studies of daily and intermittent oral PrEP among MSM were recently stopped early due to high effectiveness: 1) the immediate daily oral PrEP arm in the United Kingdom compared to the delayed PrEP arm in the PROUD study [106], and 2) the intermittent, event-driven dosing of Truvada arm compared to the placebo arm in France and Quebec in the IPERGAY study [107]. The high effectiveness demonstrated early in these studies indicate that adherence to oral PrEP among MSM is high in the context of known efficacy even when delivered with less intensive adherence counselling.

In addition, new studies and ongoing demonstration projects are examining new PrEP formulations and coitally-dependent pill/gel-schedules, which may simplify and improve adherence (see Table 5, Supplementary files). Long acting injectable and slow release delivery mechanisms (for example, using a vaginal ring) are currently being evaluated for efficacy and may be available for more real world evaluation within the next 1–3 years. Antiretrovirals (including dapivirine and tenofovir) are being formulated in sustained release vaginal rings combined with levonorgestrol for contraception (multi-purpose technologies), which may further enhance uptake and adherence for young women [108, 109]. These new PrEP delivery mechanisms are likely to be highly applicable to adolescent key populations as they do not require daily pill taking which may prove difficult for some adolescents, particularly those with unpredictable lives, unstable living situations, and/or mental health or substance use issues.

In sum, the efficacy of oral TDF and FTC/TDF has been demonstrated across multiple studies, and demonstration projects are currently evaluating strategies to improve access to, uptake of and adherence to PrEP in key populations (see Table 4 and Table 5 in Supplementary files). PrEP has great promise if integrated into a combination prevention package that provides support for the structural and behavioural barriers to this innovative biomedical prevention strategy, including accessing health care, assessing one's risk and motivation for prevention, and developing adherence habits. Below we will highlight what an ideal combination package for young key populations might look like and the potential role of PrEP within such a package.

### Combination prevention for MSM and transgender persons

An ideal combination prevention package for YMSM and young transgendered persons would include effective interventions to address behavioural risk factors, PrEP uptake and adherence support as well as addressing structural barriers to prevention (including criminalization, stigma, discrimination and homophobia). High rates of mobile phone ownership and technology use among youth provide a unique platform to deliver tailored, engaging HIV health promotion interventions to YMSM and young transgendered persons [110–112]. For example, a combination prevention app could include features to 1) increase HIV testing (e.g. provide youth with access to nearby HIV testing locations or facilitate ordering of home HIV tests); 2) help YMSM and young transgendered persons successfully access and adhere to PrEP (e.g. tracking of pill taking, side effects, pharmacy refill information); and 3) enhance patient provider interactions to ensure timely and comprehensive follow-up (e.g. symptom tracker to document any symptoms of acute HIV infection, reminders for HIV and other testing). However, to date behavioural and structural HIV prevention interventions designed specifically for YMSM and young transgendered persons are severely lacking. A recent review of primary HIV prevention interventions for adolescents/young adults found that of the 92 articles reviewed, only three unique interventions were specifically tailored to the needs of gay/bisexual male adolescents and young adults [113].

Young transgender women may require a fairly different package of combination HIV prevention interventions than young MSM. Although they may share some similar structural and social barriers, they face unique challenges, including those related to transitioning, gender discrimination, transphobia and violence [114]. A recent review has highlighted the lack of evidence-based interventions for transgender populations and the need to understand differences between MSM and transgender populations and the heterogeneity within the group so that prevention and care can be implemented more effectively [115].

Currently two studies have been conducted that have offered PrEP to younger MSM (Project PrEPare and iPrEx OLE), while only one study has included transgender persons (iPrEx OLE) [83, 84] (see Table 3). Transgender persons have been largely underrepresented in biomedical and behavioural prevention trials and more work is needed to determine the ideal set of interventions in a combination prevention package for this population [114]. In contrast, in the two years since the FDA approved Truvada for PrEP, there is growing momentum in policy related to PrEP for MSM. CDC guidance in 2014 made PrEP a central part of US prevention efforts [116], and it has been featured as one of the three key components of the New York state response to reduce new HIV infections [117]. In 2014 WHO issued guidelines for PrEP implementation which focused on MSM [118].

Project PrEPare was a pilot study conducted in the US that used a randomized 3-arm design to compare an efficacious behavioural HIV prevention intervention (Many Men, Many Voices—3 MV) alone with 3 MV combined with PrEP (tenofovir/emtricitabine), and 3 MV combined with placebo [84]. For the purposes of this trial, the 3 MV intervention was adapted for use with youth groups of mixed racial and ethnic identities. Sixty-eight youth (mean age=19.97 years; 53% African American, 40% Latino) were enrolled, 58 were randomized, 20 received PrEP and no one under the age of 18 was included [84]. Although acceptability (size of the FTC/TDF pill) was an issue for some men, the study found that 62% had tenofovir detected in plasma samples, which is an encouraging finding in this age group, and likely could be improved with an adherence support intervention during PrEP use. Future PrEP demonstration projects among YMSM should focus on acceptability, motivation and adherence support for men who are motivated to take PrEP.

To date some of the structural barriers to uptake of PrEP among YMSM have included cost of the medication and the comprehensive services required for those on PrEP, and limited access to primary care. Providers may also be not offering PrEP to those most in need. To improve uptake of PrEP, we recommend more fully integrating the provision of PrEP into sexually transmitted infection (STI) services and educating health care providers about the efficacy of PrEP and strategies for providing culturally competent and non-judgmental care for young key populations. We anticipate that the provider reluctance to prescribe PrEP will decrease in the wake of the PROUD and IPERGAY results, which indicate that MSM were able to make informed decisions about their risks and need for PrEP and adhere sufficiently to obtain substantial prevention benefits.

### Combination prevention for young people who sell sex

Combination prevention for HIV in young people who sell sex should include behavioural, structural and biomedical interventions. Community empowerment, condom promotion, HTC with linkage to treatment and care services, STI treatment and health education have been shown to be effective interventions for sex workers, but they have not been taken to scale or adequately resourced in most parts of the world [9].

To be effective, interventions targeting young people who sell sex must address their specific needs and the unique barriers they face to accessing programs for adult sex workers. For example, young sex workers may not perceive HIV prevention programs to be relevant to them, and may face competition from adult sex workers, who act as gatekeepers to sex worker HIV prevention programs. Tailored programs for younger women also need to encompass interventions that address issues of social protection which can be implemented as required on a case by case basis. Given that the majority of sex workers who acquire HIV are infected early in their career, programs need to have a strategy for identifying young people shortly after they start selling sex, and to facilitate their timely engagement with prevention services [119].

Access to prevention services is also often hampered by the legal and policy environment. UNAIDS defines sex workers as “people who receive money or goods in exchange for sexual services, either regularly or occasionally”[120], while the Convention on the Rights of the Child considers anyone selling sex under age 18 years to be sexually exploited [71]. Governments have a legal obligation to protect those under 18 from sexual exploitation and this obligation frequently results in a “raid and rescue” response to HIV prevention which perversely results in increased vulnerability and decreased access to HIV prevention services [121]. Criminalization of sex work in many settings results in young people who sell sex being afraid to seek services because of fear of arrest or imprisonment. Some countries have mandatory reporting laws for people under 18 selling sex which put health care providers in direct conflict with their responsibility to provide confidential care [75].

Although there are examples of small scale HIV prevention programs targeting young people who sell sex, these existing approaches need to be scaled up more widely and evaluated to realize improvements in HIV prevention and sexual and reproductive health among this group. For example the SHARPER project in Accra, Ghana effectively uses young peer educators who are paired with older women in the community “peer protectors”. The program focuses on health education, skills building, assisting with linkage to services and violence prevention [122]. In the Philippines, the River of Life Initiative works with young MSM who sell sex and uses peer to peer outreach to contact these hard to reach young men [123].

To date, there have been no completed trials of PrEP conducted specifically among sex workers (although two of the six trials demonstrating efficacy included sex worker participants, see Table 3). However, when the number needed to treat (NNT) to avert one HIV infection was estimated among sub-sets of women in the Partners PrEP trial, the NNT was lowest among women under 30 years and women who reported multiple high-risk behaviours. These findings suggest that the number of young women who sell sex that would need to access PrEP to prevent one infection is likely to be favourable PrEP can be safely and effectively implemented [124].

We know already that PrEP for young people who sell sex should not be considered as a stand-alone intervention, but will need to be implemented within a comprehensive package of interventions that strengthen community cohesion (such as those described in the examples above) alongside behavioural/technological approaches to build individual agency, self-efficacy and skills. The intervention components will need to be relevant to, and address the specific concerns of, young people who sell sex and be implemented in conjunction with them. It is likely that the exact form and delivery of comprehensive prevention will be context and culture specific. The next step will be to use formative research to develop and test comprehensive prevention packages for young people who sell sex, which can then be rigorously evaluated as they are scaled up using impact evaluation.

### People who inject drugs

UNAIDS has identified nine interventions considered essential to prevent HIV among IDUs. These interventions consist of needle syringe programs, opioid substitution treatment, HTC, ART, STI treatment, condom distribution, information and education campaigns, vaccination and treatment of viral hepatitis, and prevention and treatment of tuberculosis [29]. In this context, PrEP is a promising addition to the existing cadre of evidence-based interventions especially given that tenofovir does not alter the pharmacokinetics or pharmacodynamics of methadone or benprenorphine [125].

While evidence on PrEP among key populations is growing, studies with empirical data collection among PWID are limited to one PrEP efficacy trial among PWID (see Table 3). The Bangkok Tenofovir Study which was a phase III randomized double-blind placebo-controlled trial to evaluate the efficacy of PrEP with daily oral tenofovir on HIV infections in PWID [18].

Despite the promising results of the Bangkok Tenofovir Study, some have questioned whether PrEP provided protection against parenteral HIV exposure, given the low and declining incidence of reported injection and needle sharing behaviours during the trial. Although it is not possible to distinguish between the proportion of infections in the Bangkok Tenofovir Study that were attributable to parenteral versus sexual transmission [126], the key finding was the halving of HIV incidence in the PrEP arm. This is a generalizable result for HIV protection for PWIDs given that many PWID populations are at risk of HIV through both parenteral and sexual exposure. Notably, the majority of study participants were on methadone maintenance and in both arms, and injecting risk behaviours, including injecting and needle sharing decreased dramatically over three years of follow-up, suggesting that parenteral transmission may have only contributed a small proportion of the incidence. Thus, the Bangkok Tenofovir Study demonstrates that daily oral tenofovir significantly reduces HIV transmission among PWID in the context of opiate substitution therapy, and thus is a demonstration of effective combination prevention for PWID.

Several challenges remain for implementing PrEP among PWID outside a research setting. In many settings, injecting drug use is highly stigmatized, and PWID-specific HIV prevention interventions do not have adequate governmental or public support [127, 128] leading to suboptimal implementation of known highly effective prevention methods [23, 129]. Until these evidence-based intervention components including NSP, OST and HTC are successfully implemented, the role of PrEP may be limited. A recent systematic review of barriers to treatment among PWID [93] found that structural barriers, including incarceration, inadequate housing, and lack of a legal income [130, 131], were more common than individual-level barriers to accessing HIV treatment and care. In order for PrEP to be successfully implemented, a supportive political, social and environmental platform is imperative.

PrEP is not a replacement for other evidence-based programs. Rather, PrEP should be considered as part of a combination prevention package that includes other proven prevention strategies such as OST, NSP and HTC [23, 31, 34, 129, 132]. A package that integrates and provides PrEP into drug treatment programs and pharmacies and HTC clinics where there is the ability to frequently perform HIV testing and create linkages to providers to monitor patients would be ideal. In addition, it will be important to package PrEP with interventions that have been shown to increase adherence among PWID, particularly when targeting adolescent PWID, such as directly observed therapy and methadone maintenance therapy. Sub-populations of adolescent PWID such as young injecting initiates are more likely to be homeless [133] and engage in a range of risk behaviours including hazardous alcohol use, cocaine use, crystal methamphetamine use [133], unprotected sex [134, 135] and survival sex [133]. The concurrent high-risk behaviour and lack of effective treatments for cocaine or methamphetamine dependency underscore the importance of PrEP in this population [129] while at the same time highlighting their specific adherence challenges related to alcohol use [136, 137] and homelessness [138, 139]. Behavioural strategies that are part of a comprehensive approach for young people should encourage the delay of sexual debut, emphasize a reduction in the number of sexual partners and encourage the use of voluntary HTC services without concern for penalization. Further research is still needed to identify the most effective combination of interventions for PWID with an understanding that packages will need to be tailored for specific settings and sub-populations of drug users, such as adolescent and young PWID.

## Conclusions

Effective yet scalable combination packages are needed for young key populations. To date, adolescents generally, and adolescent key populations specifically, have not been included in studies of biomedical and combination prevention due to regulatory and parental permission related issues [140]. In an era of constrained resources, we need to identify intervention components that are most effective at addressing the key issues for the target population. In many settings, young key populations are at highest risk of infection. While the key populations highlighted in this paper face unique risks for HIV, they also share many important challenges to prevention, including stigma, marginalization, discrimination and, in some cases, criminalization. It is critical that we address these structural risk factors when developing prevention packages for these populations.

With regard to PrEP as part of any combination prevention package, the World Health Organization strongly recommends the use of oral PrEP among MSM based on the evidence that PrEP works in this population and is safe if taken as prescribed [21]. Improving knowledge about PrEP, and uptake of and adherence to this intervention among YMSM who have an incredibly high incidence of infection is a priority. For young PWID, expansion of harm reduction, specifically needle and syringe programs, and OST is a critical first step to creating an environment conducive to PrEP. Among sex workers, although no PrEP trials to date have specifically targeted sex workers, in particular young sex workers, PrEP has shown to be efficacious in trials that included individuals who report trading sex for money or housing. Structural impediments, including policy/law, stigma and access to health service will not be addressed by efficacy or behavioural trials, thus major policy, educational and advocacy work will be needed along with the prevention components discussed here. For all of these populations, there is a need to address critical enablers to access to HIV testing and health services for PrEP and other prevention strategies, including decriminalization of key populations’ practices, improved access to prevention and care, a reduction in stigma and discrimination, and community empowerment.

## Supplementary Material

Tailored combination prevention packages and PrEP for young key populations Click here for additional data file.

## References

[1] UNAIDS (2012). UNAIDS report on the global AIDS epidemic.

[2] UNAIDS (2013). UNAIDS report on the global AIDS epidemic.

[3] Schunter BT, Cheng WS, Kendall M, Marais H (2014). Lessons learned from a review of interventions for adolescent and young key populations in Asia Pacific and opportunities for programming. J Acquir Immune Defic Syndr.

[4] UNAIDS (2008). Redefining AIDS in Asia: crafting an effective response.

[5] Silverman JG (2011). Adolescent female sex workers: invisibility, violence and HIV. Arch Dis Child.

[6] Barrett D, Hunt N, Stoicescu C (2013). Injecting drug use among under 18s a snapshot of available data.

[7] Beyrer C, Baral SD, van Griensven F, Goodreau SM, Chariyalertsak S, Wirtz AL (2012). Global epidemiology of HIV infection in men who have sex with men. Lancet.

[8] CDC (2011). HIV surveillance report.

[9] Bekker LG, Johnson L, Cowan F, Overs C, Besada D, Hillier S (2014). Combination HIV prevention for female sex workers: what is the evidence?. Lancet.

[10] Alistar SS, Owens DK, Brandeau ML (2014). Effectiveness and cost effectiveness of oral pre-exposure prophylaxis in a portfolio of prevention programs for injection drug users in mixed HIV epidemics. PloS One.

[11] Baeten JM, Haberer JE, Liu AY, Sista N (2013). Preexposure prophylaxis for HIV prevention: where have we been and where are we going?. J Acquir Immune Defic Syndr.

[12] Cremin I, Alsallaq R, Dybul M, Piot P, Garnett G, Hallett TB (2013). The new role of antiretrovirals in combination HIV prevention: a mathematical modelling analysis. AIDS.

[13] Gomez GB, Borquez A, Caceres CF, Segura ER, Grant RM, Garnett GP (2012). The potential impact of pre-exposure prophylaxis for HIV prevention among men who have sex with men and transwomen in Lima, Peru: a mathematical modelling study. PLoS Med.

[14] Kim SB, Yoon M, Ku NS, Kim MH, Song JE, Ahn JY (2014). Mathematical modeling of HIV prevention measures including pre-exposure prophylaxis on HIV incidence in South Korea. PloS One.

[15] Lasry A, Sansom SL, Wolitski RJ, Green TA, Borkowf CB, Patel P (2014). HIV sexual transmission risk among serodiscordant couples: assessing the effects of combining prevention strategies. AIDS.

[16] Grant RM, Lama JR, Anderson PL, McMahan V, Liu AY, Vargas L (2010). Preexposure chemoprophylaxis for HIV prevention in men who have sex with men. New Engl J Med.

[17] Baeten JM, Donnell D, Ndase P, Mugo NR, Campbell JD, Wangisi J (2012). Antiretroviral prophylaxis for HIV prevention in heterosexual men and women. New Engl J Med.

[18] Choopanya K, Martin M, Suntharasamai P, Sangkum U, Mock PA, Leethochawalit M (2013). Antiretroviral prophylaxis for HIV infection in injecting drug users in Bangkok, Thailand (the Bangkok Tenofovir Study): a randomised, double-blind, placebo-controlled phase 3 trial. Lancet.

[19] Thigpen MC, Kebaabetswe PM, Paxton LA, Smith DK, Rose CE, Segolodi TM (2012). Antiretroviral preexposure prophylaxis for heterosexual HIV transmission in Botswana. New Engl J Med.

[20] Abdool Karim Q, Abdool Karim SS, Frohlich JA, Grobler AC, Baxter C, Mansoor LE (2010). Effectiveness and safety of tenofovir gel, an antiretroviral microbicide, for the prevention of HIV infection in women. Science.

[21] World Health Organization (2014). HIV prevention, diagnosis, treatment and care for key populations consolidated guidelines.

[22] CDC (2014). HIV infection and risk, prevention, and testing behaviors among injecting drug users – National HIV Behavioral Surveillance System, 20 U.S. Cities, 2009. Contract No. 6.

[23] Strathdee SA, Shoptaw S, Dyer TP, Quan VM, Aramrattana A (2012). Towards combination HIV prevention for injection drug users: addressing addictophobia, apathy and inattention. Curr Opin HIV AIDS.

[24] Cavanaugh CE, Latimer WW (2010). Recent sex trade and injection drug use among pregnant opiate and cocaine dependent women in treatment: the significance of psychiatric comorbidity. Addict Disord Their Treat.

[25] Brown QL, Cavanaugh CE, Penniman TV, Latimer WW (2012). The impact of homelessness on recent sex trade among pregnant women in drug treatment. J Subst Use.

[26] Strathdee SA, Lozada R, Martinez G, Vera A, Rusch M, Nguyen L (2011). Social and structural factors associated with HIV infection among female sex workers who inject drugs in the Mexico-US border region. PloS One.

[27] Mackesy-Amiti ME, Donenberg GR, Ouellet LJ (2012). Prevalence of psychiatric disorders among young injection drug users. Drug Alcohol Depend.

[28] Lowry R, Crosby AE, Brener ND, Kann L (2014). Suicidal thoughts and attempts among U.S. high school students: trends and associated health-risk behaviors, 1991–2011. J Adolesc Health.

[29] Gore–Felton C, Somlai AM, Benotsch EG, Kelly JA, Ostrovski D, Kozlov A (2003). The influence of gender on factors associated with HIV transmission risk among young Russian injection drug users. Am J Drug Alcohol Abuse.

[30] Miller CL, Wood E, Spittal PM, Li K, Frankish JC, Braitstein P (2004). The future face of coinfection: prevalence and incidence of HIV and hepatitis C virus coinfection among young injection drug users. J Acquir Immune Defic Syndr.

[31] Dutta A, Wirtz AL, Baral S, Beyrer C, Cleghorn FR (2012). Key harm reduction interventions and their impact on the reduction of risky behavior and HIV incidence among people who inject drugs in low-income and middle-income countries. Curr Opin HIV AIDS.

[32] Gowing L, Farrell M, Bornemann R, Sullivan L, Ali R (2008). Substitution treatment of injecting opioid users for prevention of HIV infection. Cochrane Database Syst Rev.

[33] MacArthur GJ, Minozzi S, Martin N, Vickerman P, Deren S, Bruneau J (2012). Opiate substitution treatment and HIV transmission in people who inject drugs: systematic review and meta-analysis. BMJ.

[34] MacArthur GJ, van Velzen E, Palmateer N, Kimber J, Pharris A, Hope V (2014). Interventions to prevent HIV and hepatitis C in people who inject drugs: a review of reviews to assess evidence of effectiveness. Int J Drug Policy.

[35] Abdul-Quader AS, Feelemyer J, Modi S, Stein ES, Briceno A, Semaan S (2013). Effectiveness of structural–level needle/syringe programs to reduce HCV and HIV infection among people who inject drugs: a systematic review. AIDS Behav.

[36] Wodak A, Cooney A (2006). Do needle syringe programs reduce HIV infection among injecting drug users: a comprehensive review of the international evidence. Subst Use Misuse.

[37] Booth RE, Kwiatkowski CF, Mikulich-Gilbertson SK, Brewster JT, Salomonsen-Sautel S, Corsi KF (2006). Predictors of risky needle use following interventions with injection drug users in Ukraine. Drug Alcohol Depend.

[38] UNAIDS HIV prevention among injecting drug users.

[39] Tempalski B, Pouget ER, Cleland CM, Brady JE, Cooper HL, Hall HI (2013). Trends in the population prevalence of people who inject drugs in US metropolitan areas 1992–2007. PloS One.

[40] Marshal MP, Friedman MS, Stall R, Thompson AL (2009). Individual trajectories of substance use in lesbian, gay and bisexual youth and heterosexual youth. Addiction.

[41] Marshal MP, Friedman MS, Stall R, King KM, Miles J, Gold MA (2008). Sexual orientation and adolescent substance use: a meta-analysis and methodological review. Addiction.

[42] Balaji AB, Bowles KE, Le BC, Paz-Bailey G, Oster AM, Group NS (2013). High HIV incidence and prevalence and associated factors among young MSM, 2008. AIDS.

[43] Hightow-Weidman LB, Phillips G, Jones KC, Outlaw AY, Fields SD, Smith JC (2011). Racial and sexual identity-related maltreatment among minority YMSM: prevalence, perceptions, and the association with emotional distress. AIDS Patient Care STDS.

[44] Garofalo R, Wolf RC, Wissow LS, Woods ER, Goodman E (1999). Sexual orientation and risk of suicide attempts among a representative sample of youth. Arch Pediatr Adolesc Med.

[45] D'Augelli AR, Grossman AH, Starks MT (2006). Childhood gender atypicality, victimization, and PTSD among lesbian, gay, and bisexual youth. J Interpers Violence.

[46] Brewer RA, Magnus M, Kuo I, Wang L, Liu TY, Mayer KH (2014). The high prevalence of incarceration history among Black men who have sex with men in the United States: associations and implications. Am J Public Health.

[47] Dyer TP, Regan R, Wilton L, Harawa NT, Ou SS, Wang L (2013). Differences in substance use, psychosocial characteristics and HIV-related sexual risk behavior between Black men who have sex with men only (BMSMO) and Black men who have sex with men and women (BMSMW) in six US cities. J Urban Health.

[48] Irvin R, Wilton L, Scott H, Beauchamp G, Wang L, Betancourt J (2014). A study of perceived racial discrimination in Black men who have sex with men (MSM) and its association with healthcare utilization and HIV testing. AIDS Behav.

[49] Koblin BA, Mayer KH, Eshleman SH, Wang L, Mannheimer S, del Rio C (2013). Correlates of HIV acquisition in a cohort of Black men who have sex with men in the United States: HIV prevention trials network (HPTN) 061. PloS One.

[50] Mayer KH, Wang L, Koblin B, Mannheimer S, Magnus M, del Rio C (2014). Concomitant socioeconomic, behavioral, and biological factors associated with the disproportionate HIV infection burden among Black men who have sex with men in 6 U.S. cities. PloS One.

[51] Mimiaga MJ, Goldhammer H, Belanoff C, Tetu AM, Mayer KH (2007). Men who have sex with men: perceptions about sexual risk, HIV and sexually transmitted disease testing, and provider communication. Sex Transm Dis.

[52] CDC (2005). HIV prevalence, unrecognized infection and HIV testing among men who have sex with men – five US cities, June 2004–April 2005. MMWR.

[53] Kellerman SE, Lehman JS, Lansky A, Stevens MR, Hecht FM, Bindman AB (2002). HIV testing within at-risk populations in the United States and the reasons for seeking or avoiding HIV testing. J Acquir Immune Defic Syndr.

[54] Campsmith ML, Goldbaum GM, Brackbill RM, Tollestrup K, Wood RW, Weybright JE (1997). HIV testing among men who have sex with men – results of a telephone survey. Prev Med.

[55] Oster AM, Wiegand RE, Sionean C, Miles IJ, Thomas PE, Melendez-Morales L (2011). Understanding disparities in HIV infection between black and white MSM in the United States. AIDS.

[56] Duncan DT, Hatzenbuehler ML, Johnson RM (2014). Neighborhood-level LGBT hate crimes and current illicit drug use among sexual minority youth. Drug Alcohol Depend.

[57] Herbst JH, Jacobs ED, Finlayson TJ, McKleroy VS, Neumann MS, Crepaz N (2008). Estimating HIV prevalence and risk behaviors of transgender persons in the United States: a systematic review. AIDS Behav.

[58] Hoffman B (2014). An overview of depression among transgender women. Depress Res Treat.

[59] Santos GM, Wilson EC, Rapues J, Macias O, Packer T, Raymond HF (2014). HIV treatment cascade among transgender women in a San Francisco respondent driven sampling study. Sex Transm Infect.

[60] Beattie TS, Bhattacharjee P, Ramesh BM, Gurnani V, Anthony J, Isac S (2010). Violence against female sex workers in Karnataka state, south India: impact on health, and reductions in violence following an intervention program. BMC Public Health.

[61] Blanchard JF, O'Neil J, Ramesh BM, Bhattacharjee P, Orchard T, Moses S (2005). Understanding the social and cultural contexts of female sex workers in Karnataka, India: implications for prevention of HIV infection. J Infect Dis.

[62] Sarkar K, Bal B, Mukherjee R, Chakraborty S, Saha S, Ghosh A (2008). Sex-trafficking, violence, negotiating skill, and HIV infection in brothel-based sex workers of eastern India, adjoining Nepal, Bhutan, and Bangladesh. J Health Popul Nutr.

[63] Shannon K, Kerr T, Strathdee SA, Shoveller J, Montaner JS, Tyndall MW (2009). Prevalence and structural correlates of gender based violence among a prospective cohort of female sex workers. BMJ.

[64] Grossman AH, D'Augelli AR (2006). Transgender youth: invisible and vulnerable. J Homosexual.

[65] Swart-Kruger J, Richter LM (1997). AIDS-related knowledge, attitudes and behaviour among South African street youth: reflections on power, sexuality and the autonomous self. Soc Sci Med.

[66] World Health Organization (1998). Coming of age: from facts to action for adolescent sexual and reproductive health.

[67] Towe VL, ul Hasan S, Zafar ST, Sherman SG (2009). Street life and drug risk behaviors associated with exchanging sex among male street children in Lahore, Pakistan. J Adolescent Health.

[68] Busza JR, Balakireva OM, Teltschik A, Bondar TV, Sereda YV, Meynell C (2011). Street-based adolescents at high risk of HIV in Ukraine. J Epidemiol Community Health.

[69] Haley N, Roy E, Leclerc P, Boudreau JF, Boivin JF (2004). HIV risk profile of male street youth involved in survival sex. Sex Transm Infect.

[70] Kombarakaran FA (2004). Street children of Bombay: their stresses and strategies of coping. Child Youth Serv Rev.

[71] World Health Organization (2014). HIV and young people who sell sex: a technical brief.

[72] McCartney D, Yadav G At what age? Autonomy and the role of parents and the state in young people's access to HIV and sexual and reproductive health Services.

[73] Napierala Mavedzenge SM, Doyle AM, Ross DA (2011). HIV prevention in young people in sub-Saharan Africa: a systematic review. J Adolescent Health.

[74] McClure C, Chandler C, Bissell S (2014). Responses to HIV in sexually exploited children or adolescents who sell sex. Lancet.

[75] Conner B, Middleton-Lee S, Mago A Sexual and reproductive health needs and access to health services for adolescents 10–18 engaged in selling sex in Asia Pacific.

[76] HIV Young Leaders Fund “First, do no harm:” an advocacy brief on sexual and reproductive health needs and access to health services for adolescents 10–17 engaged in selling sex in the Asia Pacific.

[77] World Health Organization (2013). UNPF, Joint United Nations Programme on HIV/AIDS, Global Network of Sex Work Projects, The World Bank. Implementing comprehensive HIV/STI programmes with sex workers: practical approaches from collaborative interventions. http://apps.who.int/iris/bitstream/10665/90000/1/9789241506182_eng.pdf?ua=1.

[78] ECPAT International (2011). http://resources.ecpat.net/EI/Pdf/A4A_II/A4A_V2_SA_PAKISTAN.pdf.

[79] ECPAT International (2006). The boys and the bullies: a situation analysis research on prostitution of boys in Bangladesh. http://www.humantrafficking.org/uploads/publications/Bangladesh_Part1.pdf.

[80] ECPAT International (2006). Situational analysis report on prostitution of boys in India (Hyderabad). http://www.humantrafficking.org/uploads/publications/India_Hyderabad.pdf.

[81] Celum C, Baeten JM, Hughes JP, Barnabas R, Liu A, Van Rooyen H (2013). Integrated strategies for combination HIV prevention: principles and examples for men who have sex with men in the Americas and heterosexual African populations. J Acquir Immune Defic Syndr.

[82] Van Damme L, Corneli A, Ahmed K, Agot K, Lombaard J, Kapiga S (2012). Preexposure prophylaxis for HIV infection among African women. New Engl J Med.

[83] Grant RM, Anderson PL, McMahan V, Liu A, Amico KR, Mehrotra M (2014). Uptake of pre-exposure prophylaxis, sexual practices, and HIV incidence in men and transgender women who have sex with men: a cohort study. Lancet Infect Dis.

[84] Hosek SG, Siberry G, Bell M, Lally M, Kapogiannis B, Green K (2013). The acceptability and feasibility of an HIV preexposure prophylaxis (PrEP) trial with young men who have sex with men. J Acquir Immune Defic Syndr.

[85] Grohskopf L, Gvetadze R, Pathak S, O'Hara B, Mayer K, Liu A Preliminary analysis of biomedical data from the phase II clinical safety trial of tenofovir disoproxil fumarate (TDF) for HIV-1 pre-exposure prophylaxis (PrEP) among U.S. men who have sex with men (MSM).

[86] Marrazzo J, Ramjee G, Nair G, Palanee T, Mkhize B, Nakabiito Taljaard M Pre-exposure prophylaxis for HIV in Women: daily oral tenofovir, oral tenofovir/emtricitabine, or vaginal tenofovir gel in the VOICE Study (MTN 003).

[87] Eisingerich AB, Wheelock A, Gomez GB, Garnett GP, Dybul MR, Piot PK (2012). Attitudes and acceptance of oral and parenteral HIV preexposure prophylaxis among potential user groups: a multinational study. PloS One.

[88] Thannhauser JE, Mah JK, Metz LM (2009). Adherence of adolescents to multiple sclerosis disease-modifying therapy. Pediatr Neurol.

[89] Taddeo D, Egedy M, Frappier JY (2008). Adherence to treatment in adolescents. Paediatr Child Health.

[90] Salema NE, Elliott RA, Glazebrook C (2011). A systematic review of adherence-enhancing interventions in adolescents taking long-term medicines. J Adolesc Health.

[91] Rudy BJ, Murphy DA, Harris DR, Muenz L, Ellen J (2009). Adolescent Trials Network for HIVAI. Patient–related risks for nonadherence to antiretroviral therapy among HIV-infected youth in the United States: a study of prevalence and interactions. AIDS Patient Care STDS.

[92] Marcus J, Buisker T, Horvath T, Amico K, Fuchs J, Buchbinder S (2014). Helping our patients take HIV pre-exposure prophylaxis (PrEP): a systematic review of adherence interventions. HIV Med.

[93] Okwundu CI UO, Okoromah CAN (2012). Antiretroviral pre-exposure prophylaxis (PrEP) for preventing HIV in high-risk individuals. Cochrane Database Syst Rev.

[94] Marrazzo J, Ramjee G, Nair G, Palanee T, Mkhize B, Nakabiito C Pre-exposure prophylaxis for HIV in women: daily oral tenofovir, oral tenofovir/emtricitabine, or vaginal tenofovir gel in the VOICE study (MTN 003).

[95] Thurston IB, Bogart LM, Wachman M, Closson EF, Skeer MR, Mimiaga MJ (2014). Adaptation of an HIV medication adherence intervention for adolescents and young adults. Cogn Behav Pract.

[96] Reisner SL, Mimiaga MJ, Skeer M, Perkovich B, Johnson CV, Safren SA (2009). A review of HIV antiretroviral adherence and intervention studies among HIV-infected youth. Top HIV Med.

[97] Crawford ND, Vlahov D (2010). Progress in HIV reduction and prevention among injection and noninjection drug users. J Acquir Immune Defic Syndr.

[98] Milloy MJ, Montaner J, Wood E (2012). Barriers to HIV treatment among people who use injection drugs: implications for “treatment as prevention.”. Curr Opin HIV AIDS.

[99] Diabaté S, Zannou D, Geraldo N, Chamberland A, Akakpo J, Ahouada C (2011). Antiretroviral therapy among HIV-1 infected female sex workers in Benin: a comparative study with patients from the general population. World J AIDS.

[100] Huet C, Ouedraogo A, Konate I, Traore I, Rouet F, Kabore A (2011). Long-term virological, immunological and mortality outcomes in a cohort of HIV-infected female sex workers treated with highly active antiretroviral therapy in Africa. BMC Public Health.

[101] Escudero DJ, Lurie MN, Kerr T, Howe CJ, Marshall BD (2014). HIV pre-exposure prophylaxis for people who inject drugs: a review of current results and an agenda for future research. J Int AIDS Soc.

[102] Campbell JD, Herbst JH, Koppenhaver RT, Smith DK (2013). Antiretroviral prophylaxis for sexual and injection drug use acquisition of HIV. Am J Prev Med.

[103] Ware NC, Wyatt MA, Haberer JE, Baeten JM, Kintu A, Psaros C (2012). What's love got to do with it? Explaining adherence to oral antiretroviral pre-exposure prophylaxis for HIV-serodiscordant couples. J Acquir Immune Defic Syndr.

[104] Amico KR, Mansoor LE, Corneli A, Torjesen K, van der Straten A (2013). Adherence support approaches in biomedical HIV prevention trials: experiences, insights and future directions from four multisite prevention trials. AIDS Behav.

[105] van der Straten A, Stadler J, Montgomery E, Hartmann M, Magazi B, Mathebula F (2014). Women's experiences with oral and vaginal pre-exposure prophylaxis: the VOICE-C qualitative study in Johannesburg, South Africa. PloS One.

[106] AVAC (2014). PROUD day for PrEP in gay men and other MSM in the UK. http://www.avac.org/blog/proud-day-prep-gay-men-and-other-msm-uk.

[107] Mascolini M (2014). French trial finds “On-Demand” PrEP protection from HIV in MSM. http://www.iasociety.org/Default.aspx?pageId=5&elementId=16083.

[108] Brady M, Manning J (2013). Lessons from reproductive health to inform multipurpose prevention technologies: don't reinvent the wheel. Antiviral Res.

[109] Tolley EE, Morrow KM, Owen DH (2013). Designing a multipurpose technology for acceptability and adherence. Antiviral Res.

[110] Hightow-Weidman LB, Pike E, Fowler B, Matthews DM, Kibe J, McCoy R (2012). HealthMpowerment.org: feasibility and acceptability of delivering an internet intervention to young Black men who have sex with men. AIDS Care.

[111] LeGrand S, Muessig KE, Pike EC, Baltierra N, Hightow-Weidman LB (2014). If you build it will they come? Addressing social isolation within a technology-based HIV intervention for young black men who have sex with men. AIDS Care.

[112] Muessig KE, Pike EC, Fowler B, LeGrand S, Parsons JT, Bull SS (2013). Putting prevention in their pockets: developing mobile phone-based HIV interventions for black men who have sex with men. AIDS Patient Care STDs.

[113] Harper GW, Riplinger AJ (2013). HIV prevention interventions for adolescents and young adults: what about the needs of gay and bisexual males?. AIDS Behav.

[114] Escudero DJ, Kerr T, Operario D, Socias ME, Sued O, Marshall BD (2014). Inclusion of trans women in pre-exposure prophylaxis trials: a review. AIDS Care.

[115] Allison SM, Adams D, Klindera KC, Poteat T, Wolf RC (2014). Innovative uses of communication technology for HIV programming for men who have sex with men and transgender persons. J Int AIDS Soc.

[116] CDC (2014). Preexposure prophylaxis for the prevention of HIV infection in the United States – 2014: a clinical practice guideline.

[117] New York Governor's Press Office (2014). Governor Cuomo announces plan to end the AIDS epidemic in New York State. https://www.governor.ny.gov/press/06292014-end-aids-epidemic.

[118] World Health Organization (2014). Consolidated guidelines on HIV prevention, diagnosis, treatment and care for key populations.

[119] Asamoah-Adu C, Khonde N, Avorkliah M, Bekoe V, Alary M, Mondor M (2001). HIV infection among sex workers in Accra: need to target new recruits entering the trade. J Acquir Immune Defic Syndr.

[120] UNAIDS (2009). Guidance note on HIV and sex work.

[121] Steen R, Jana S, Reza-Paul S, Richter M (2014). Trafficking, sex work, and HIV: efforts to resolve conflicts. Lancet.

[122] FHI 360 Strengthening HIV/AIDS response partnership with evidenced-based results (SHARPER). http://www.fhi360.org/projects/strengthening-hivaids-response-partnership-evidenced-based-results-sharper.

[123] The River of Life Initiatives (ROLi) Programme. http://www.projectpage.info/my-river-of-life/about-us.

[124] Murnane PM, Celum C, Kahle EM, Donnell D, Bukusi E, Mugo N Daily oral pre-exposure prophylaxis in highly effective among subsets of highest-risk participants: Partners PrEP Study.

[125] Baker J, Rainey PM, Moody DE, Morse GD, Ma Q, McCance-Katz EF (2010). Interactions between buprenorphine and antiretrovirals: nucleos(t)ide reverse transcriptase inhibitors (NRTI) didanosine, lamivudine, and tenofovir. Am J Addict.

[126] Karim SS (2013). HIV pre-exposure prophylaxis in injecting drug users. Lancet.

[127] Mathers BM, Degenhardt L, Ali H, Wiessing L, Hickman M, Mattick RP (2010). HIV prevention, treatment, and care services for people who inject drugs: a systematic review of global, regional, and national coverage. Lancet.

[128] Wolfe D, Carrieri MP, Shepard D (2010). Treatment and care for injecting drug users with HIV infection: a review of barriers and ways forward. Lancet.

[129] Baral SD, Stromdahl S, Beyrer C (2012). The potential uses of preexposure prophylaxis for HIV prevention among people who inject drugs. Curr Opin HIV AIDS.

[130] Milloy MJ, Kerr T, Buxton J, Rhodes T, Krusi A, Guillemi S (2012). Social and environmental predictors of plasma HIV RNA rebound among injection drug users treated with antiretroviral therapy. J Acquir Immune Defic Syndr.

[131] Palepu A, Milloy MJ, Kerr T, Zhang R, Wood E (2011). Homelessness and adherence to antiretroviral therapy among a cohort of HIV-infected injection drug users. J Urban Health.

[132] Degenhardt L, Mathers B, Vickerman P, Rhodes T, Latkin C, Hickman M (2010). Prevention of HIV infection for people who inject drugs: why individual, structural, and combination approaches are needed. Lancet.

[133] Roy E, Godin G, Boudreau JF, Cote PB, Denis V, Haley N (2011). Modeling initiation into drug injection among street youth. J Drug Educ.

[134] Mackesy-Amiti ME, Boodram B, Williams C, Ouellet LJ, Broz D (2013). Sexual risk behavior associated with transition to injection among young non-injecting heroin users. AIDS Behav.

[135] Neaigus A, Reilly KH, Jenness SM, Hagan H, Wendel T, Gelpi-Acosta C (2013). Dual HIV risk: receptive syringe sharing and unprotected sex among HIV-negative injection drug users in New York City. AIDS Behav.

[136] Chander G, Lau B, Moore RD (2006). Hazardous alcohol use: a risk factor for non-adherence and lack of suppression in HIV infection. J Acquir Immune Defic Syndr.

[137] Tran BX, Nguyen LT, Do CD, Nguyen QL, Maher RM (2014). Associations between alcohol use disorders and adherence to antiretroviral treatment and quality of life amongst people living with HIV/AIDS. BMC Public Health.

[138] Milloy MJ, Kerr T, Bangsberg DR, Buxton J, Parashar S, Guillemi S (2012). Homelessness as a structural barrier to effective antiretroviral therapy among HIV-seropositive illicit drug users in a Canadian setting. AIDS Patient Care STDs.

[139] Milloy MJ, Marshall BD, Montaner J, Wood E (2012). Housing status and the health of people living with HIV/AIDS. Current HIV/AIDS reports.

[140] Bekker LG, Slack C, Lee S, Shah S, Kapogiannis B (2014). Ethical issues in adolescent HIV research in resource-limited countries. J Acquir Immune Defic Syndr.

# Sub article: HIV testing and linkage to services for youth

**DOI:** 10.7448/IAS.18.2.19433

**Published:** 2015-02-26

**Authors:** Ann E Kurth, Michelle A Lally, Augustine T Choko, Irene W Inwani, J Dennis Fortenberry

**Affiliations:** 1 College of Nursing and Global Institute of Public Health, New York University, New York, NY, USA; 2 Lifespan Hospital System, Providence, VA, USA; 3 Alpert Medical School, Brown University, Providence, RI, USA; 4 Malawi-Liverpool Wellcome Trust Clinical Research Programme, Blantyre, Malawi; 5 Kenyatta National Hospital, Nairobi, Kenya; 6 Department of Pediatrics, Indiana University, Indianapolis, IN, USA

**Keywords:** HIV testing, HIV continuum of care, youth, adolescents, key populations, development

## Abstract

**Introduction:**

HIV testing is the portal to serostatus knowledge that can empower linkage to care for HIV treatment and HIV prevention. However, young people's access to HIV testing is uneven worldwide. The objective of this paper is to review the context and concerns faced by youth around HIV testing in low- as well as high-income country settings.

**Discussion:**

HIV testing is a critical entry point for primary and secondary prevention as well as care and treatment for young people including key populations of vulnerable youth. We provide a framework for thinking about the role of testing in the continuum of prevention and care for young people. Brief case study examples from Kenya and the US illustrate some of the common barriers and issues involved for young people.

**Conclusions:**

Young people worldwide need more routine access to HIV testing services that effectively address the developmental, socio-political and other issues faced by young women and men.

## Introduction

Youth aged 15–24 represent 39% of new HIV infections in people aged 15 years and older (2012) [1]. Among young people with HIV, most (4 million) live in sub-Saharan Africa [2]. Access to HIV testing and to antiretroviral therapy (ART) for youth remains a concern globally. Young people's HIV testing levels in low- and middle-income countries (LMICs), that contain most global HIV disease burden, is uneven [3]. Fewer than one in five boys and one in three girls aged 15–19 years in Africa report ever HIV testing [2]. The United States has poor sexual health statistics including in HIV testing access [4]; the proportion of US youth who HIV test has remained low at 22% and stagnant since 2005 [5]. For key populations (KPs), including males who have sex with males (MSM), people who inject drugs (PWID), transgender people (TG) and sex workers (SW), access to HIV testing is even more challenging due to marginalization and stigma.

The seek, test, treat, retain and suppress continuum has been promulgated as an approach with potential to bend the curve of the HIV epidemic [6]. Knowledge of serostatus is a starting point for lifesaving ART and to reduce sexual, parenteral, or vertical transmission. The particular HIV testing barriers and facilitators for youth in the HIV continuum of care have had less focus, however. Attention to developmental milestones is critical, e.g., yet most of what is known regarding linkage and retention in care has been based on adult, not youth populations. The sense of invulnerability that many adolescents feel – despite epidemiologic risks – also contributes [7,8]. Young people who are part of KP subgroups face overt discrimination and have lower testing rates than general population youth, facing additional barriers including fear, concerns about confidentiality and cost [4], low self-efficacy [9] and lack of KP-youth-friendly services. In this paper, we highlight critical issues involved for youth, including KPs, along the HIV testing–prevention–treatment continuum.

### Framework: testing as entry to prevention and treatment

Testing for HIV is offered via provider-initiated testing and counselling (PITC) in health facilities, on a self-initiated basis through voluntary counselling and testing sites (VCT), delivered by staff in homes (home-based or HBCT), through community campaigns and through self-testing. Each modality has benefits and drawbacks specific to youth, yet HIV testing overall serves as a critical core component of the HIV continuum for vulnerable youth. In Figure 1, we graphically summarize a model where testing for HIV functions as a triage portal for needed youth-friendly services around an HIV Continuum of Prevention and Care, including housing, mental health treatment, substance abuse treatment and sexually transmitted infection (STI) services.

**Figure 1:** Framework for HIV services for youth in the HIV continuum.

At-risk and KP youth [10] need safe opportunities to test, and re-test, for HIV. Acute HIV infection is important to identify [11] and US guidelines call for 4th generation screening tests [12], though these may not be available in many settings globally. Linkage to care for positive youth is especially challenging. Even with dedicated outreach and youth-friendly clinics, only 70% of HIV-infected youth in the US Adolescent Trials Network were successfully linked [13].

The primary goal of HIV care is viral suppression, with strong evidence for immunological advantage from early suppression [14]. Individuals also gain emotional assurance that viral suppression minimizes risk for transmission. This benefit can be realized on a population level [15]. Lifelong ART is challenging, with high attrition post-ART initiation among youth noted in Africa [16] and elsewhere. Technologies that are highly acceptable to young people, such as text messaging, to support adherence are promising [17]. Technology tools also have been used successfully to support HIV testing uptake among adolescents including in US emergency departments [18]. Partner services are critical for HIV-infected youth. Keeping HIV-negative youth healthy and uninfected remains a key goal.

Since the global burden of HIV disease is in sub-Saharan Africa, with two-thirds of all HIV cases and four-fifths of all young persons living with HIV, making new modalities such as self-tests available may help KP youth gain access to serostatus knowledge. In Malawi, in work done by Choko and colleagues, uptake of HIV self-testing among young people aged 16–24 years was consistently higher than among adults aged ≥25 years: 93.7% among those under age 25 versus 65.5% among adults 25 years and older (*p<*0.001). However, only 42.4% of the youth in that study had ever HIV tested, compared with 57.6% of those 25 and older (*p*<0.001) [19], indicating an unmet need as the younger age group is sexually active and exposed to HIV including through sex with older partners more likely to be HIV-infected [20].

In Kenya, where an estimated 100,000 new HIV infections occurred in 2013, girls and KPs are disproportionately affected by HIV [21]. School-based HIV education does not equip youth to seek testing, and there are few youth-friendly facilities available. Policy guidance says minors require parental/guardians’ consent for HIV testing, though Kenya has eliminated age limit as the only criteria. Many healthcare providers are ignorant of this provision, however, and deny unaccompanied adolescents an HIV test. In the country's new HIV roadmap [21], there is commitment to reviewing parental consent for HIV testing for adolescents. Youth who discuss testing with their parents are more likely to HIV test [22]. However, youth often rightfully fear negative reactions from parents and providers, including in schools where they fear isolation and missed opportunities and employment prospects if known to be HIV-positive. In some communities, women cannot give consent without consent by family members (case example, Box 1).

*Box 1*. Benta [a pseudonym], 17 years old, was admitted with her 2 year-old child into the paediatric ward. She comes from a pastoral community, got married at 15 and never attended antenatal clinic. She does not know her HIV status. She and her child are offered provider-initiated HTC. She has to get permission from her mother-in-law who says Benta and her child can be tested but only if the father of the child consents. He cannot be reached by phone, does not visit the family in the hospital for 10 days, and Benta and her child leave hospital without knowing their HIV status.

KPs including MSM and transgender youth in sub-Saharan Africa are often hidden and it is not safe to self-identify to providers (Box 2). Issues faced by youth for HIV testing cut across country contexts or resource boundaries, especially when in persecuted groups like MSM (Box 3 from high-income US setting).

*Box 2*. Paul [a pseudonym] is a transgender youth in Kenya. After high school, he was not able to get a job. One day he dressed like a female and got employed as house help looking after two girls. One day the girls and their mother saw Paul in a mall, dressed as a young man. The mother confronted him claiming Paul was masquerading as a woman with intent to abuse her children. A crowd gathered and physically assaulted Paul. Police officers forcibly took him to a nearby clinic where he was tested for STIs including HIV without consent. Results were disclosed without consent. He lost his job and had to re-locate.

*Box 3*. Michael [a pseudonym] is an 18-year-old who has been homeless for 12 months. He exchanges sex with other men for money in order to survive, and most do not use condoms. He was recently tested for HIV, and he was told it was positive. He is sure it was a mistake, and avoids going to any health facility.

## Discussion

### Developmental issues relevant to HIV testing

The dividing line of adolescence and adulthood is often seen as a sharp transition (e.g. at 18 or 21 years). These age transitions mark relevant thresholds for age of consent for HIV testing, for HIV medical services, and for partnered sex (Figure 1). Although adolescence is developmentally continuous and subject to substantial individual, cultural and national variation, it is useful to think of the HIV service continuum in the context of early (10–14 years), middle (15–17 years) and late adolescence (18 years and older).

#### Early adolescence (10–14 years)

Early adolescence is marked by puberty, achievement of adult size and gender-typical body contours with new assumptions about responsibility for sexual behaviour. Family and economic situations may require contributions to household income and sibling care that affect schooling and vocational opportunities [23]. Puberty in many cultures is associated with initiation rites that may not include HIV prevention messages [24–26] and that carry potential risks including non-medical circumcision [27,28]. (Voluntary medical male circumcision plays a critical role in HIV prevention and is well-accepted by many young men and parents [29].)

Development of sexual orientation is a key task. Often heterosexual identity is assumed as the “normal” outcome while other identities may be considered deviant [30]. During early and middle adolescence, sexual orientation has substantial variation and fluidity and often, lack of congruence between identity and behaviour [31].

Much emphasis is given to timing of coitus [32,33]. Over-emphasis on adolescent coitus complicates appropriate matching of services because many adolescents do not have coitus yet engage in other partnered sexual behaviours associated with HIV risk. Same-sex partnered behaviours often are omitted from sexuality education or relegated to being entirely risky without contribution to sexual or relationship satisfaction.

As pointed out in Figure 1, there may be a mismatch and delay between when young people begin having sex and when they can legally obtain HIV testing independently. HIV tests before first partnered sexual event have unproven benefits (e.g. normalization of testing) and harms (e.g. false security). After first partnered sex, it is unclear when young people begin to seek HIV testing on their own, or when clinicians recommend testing [33], despite guidelines that paediatricians and youth providers offer on HIV testing around age 13 onward [7].

Health providers should ask about sexual activity among younger patients. Many youth are not consensually sexually active and may acquire HIV via sexual abuse, or may have acquired HIV perinatally. In both these cases the young person may not be willing to share their sexual activity history at their first encounter with a new provider. Adolescents’ non-consensual sexual experiences and intimate partner violence (IPV) may increase risky behaviours [34,35]. Few IPV victims report discussions with a provider, demonstrating the importance of routine assessment for partner violence [36].

Sexuality education may occur in secondary schools, although content varies greatly [37–39] and this misses out-of-school youth. Primary emphasis on abstinence-until-marriage is less effective for HIV prevention than age-appropriate, comprehensive programs [40–42]. Informal sources of information including social media are ubiquitous in adolescents’ daily lives worldwide [32,43–45].

#### Middle adolescence (15–17 years)

By this stage some functional competencies needed to manage one's health may be in place. However, many adolescents lack skills or status to negotiate complex systems [46].

Adolescents’ participation in the HIV continuum of care as consumers of health products (e.g. condoms, pregnancy tests) is infrequently explored. Sale of HIV self-testing kits is not age-restricted although costs, test implementation fidelity and point-of-sale confidentiality have not been fully explored with young, high-risk persons [47–49] . Early data on self-testing acceptability, as seen in the Malawi example, are encouraging.

Many youth in this age group routinely have sex, especially in subgroups where survival depends on sexual exchange, which is often unprotected given power differentials in these encounters. Many KP youth live on their own, though are not yet an age of legal majority.

#### Late adolescence – youth (18–24 years)

Age 18 often is considered adulthood; however, it is now known if significant brain development including in the prefrontal cortex responsible for decision-making does not actually mature fully until age 25, which may influence vulnerability and resilience of young people [50] in terms of HIV risk and testing decisions.

### Legal issues and the HIV continuum of care for adolescents

Three highly variable (from jurisdiction to jurisdiction) milestones dictate legal thresholds for adolescents’ engagement in the HIV continuum of care: age of consent for partnered sex; for HIV testing; and, for HIV medical services (Figure 1). Adolescents’ differential legal access to HIV-related testing and other services is based on traditional assumptions of parental rights as well as restricted autonomy of children [51].

Identification of sexual activity of minors less than the age of consent threshold may mandate reporting to child protection authorities [52]. Given concerns about widespread victimization and HIV (especially of girls and younger adolescents) [53], some countries have enacted “defilement” laws that can be enforced without regard for consensuality of the partnered sex [54].

The ethical concept of “the mature minor,” while infrequently given legal sanction for adolescents’ self-consent for general medical treatment [55], informs legal exceptions to consent requirements for HIV [56,57]. Age thresholds for consent of diagnostic HIV testing are widely variable, often as young as 12 years of age. These laws recognize that parental permission is a critical barrier to HIV testing, and could invoke physical danger if non-marital or same-sex activity is suspected or disclosed. Age thresholds for minor self-consent for HIV is sometimes addressed within the context of laws that allow for STI assessment [58,59]. However, medical HIV treatments are lifelong and expensive, requiring ongoing relationships with providers [60].

#### Cross-cutting issues for youth that affect HIV testing uptake

Across all age groups, stigma adversely affects each phase of adolescents’ engagement with the HIV continuum [61]. Internalized stigma may particularly effect HIV testing behaviours while anticipated stigma may have especially strong effects on care-seeking and adherence [58,59].

Physical, sexual and emotional aggression is experienced by many youth, especially in KP groups (e.g. sexual minorities), where microaggressions also have a damaging impact [62]. Legal protection, and campaigns to reduce bullying and other forms of aggression, are needed. Finally, many young people are economically disadvantaged relative to adults and cost barriers to HIV testing must be effectively addressed.

### Solutions to increase HIV testing uptake among youth including KPs

HIV testing services must be available to all young people, particularly those from KPs. Health literacy is an issue for many adolescents [63] which “youth-friendly” programs may address [64]. Demand creation strategies have been used effectively for HIV testing via social marketing campaigns [65] and should be further employed. Testing availability where youth gather, and user-friendly free or subsidized test kits, may increase uptake. Once confirmed positive, linkage to care is critical and youth should access treatment services in whatever clinical venue is preferred, whether paediatric or adult (to reduce loss to follow-up when forced into adult services at arbitrary age cutoffs like 18 years). Testing and care are enhanced by respectful health care teams, and reduction of resource barriers such as transport fees and homelessness, that disrupt treatment continuity. Counselling can address the utility of ART taking into account developmental stages in which many young people may feel invulnerable and find navigating complex health systems overwhelming, especially in the commonly-occurring context of depression, substance use and other co-morbidities. Failure to link/retain adequately has dire consequence; Zanoni and Mayer estimate that only 6% of HIV-positive US youth are virally suppressed. They recommend that HIV testing be integrated wherever youth interact with health systems, as well as in youth venues, to normalize and promote testing and recurrent testing among high-risk and KP youth [66].

## Conclusions

### Recommendations and research gaps

Adolescents and young adults worldwide deserve better access to HIV testing and re-testing. We recommend that testing venues be made more youth-friendly, and promising new approaches like self-testing be monitored regarding how well they work for youth. Implementation science can identify optimal ways to improve HIV testing access and delivery for youth [67]. HIV testing in prevention of maternal to child transmission (PMTCT), antepartum care and voluntary medical male circumcision (VMMC) campaigns alone is insufficient. For youth, HIV testing is a key portal for linkage to necessary HIV care and prevention services.

Despite international and national guidelines, HIV testing for adolescents is still not consistently done in high- [68] or lower-income countries. Providers worldwide [69] must consistently assess sexual behaviours or partnership risks, so that appropriate counselling based on the young person's actual needs is not pre-empted [10]. HIV testing can be made more youth-friendly [70] even under the constraints of ART scale-up [71], but truly supportive services ultimately must rely on empathetic, self-aware [72] and professional health provider behaviours [73] including assurance of confidentiality around test results [74], reinforcement for those testing HIV-negative, and social and clinical support for those testing HIV-positive. Ensuring youth rights cannot occur only within clinic walls but must extend to the community and to social as well as legal norms [75].

There are social justice and public health imperatives to focus on structural factors that keep young people from freely HIV testing – including laws that harm KPs and program structures and costs that restrict access. The HIV Investment Framework points out that contraceptive services are a cost-effective portal for youth HIV testing [76], of even more importance in LMIC settings where a higher proportion of the population are of reproductive ages [77]. Achieving universal access to youth-friendly services worldwide would cost around US$ 1 per adolescent [78]. Program quality monitoring of HIV testing access [79], and implementation of best HIV testing practices, for young people must a part of the HIV agenda if we are to achieve generations with fewer HIV infections and provide better care of those living with HIV.

## References

[1] UNAIDS (2012). Fact Sheet: UNAIDS's vision: zero new HIV infections. Zero discrimination. http://www.unaids.org/en/media/unaids/contentassets/documents/factsheet/2012/20120417_FS_adolescentsyoungpeoplehiv_en.pdf.

[2] Idele P, Gillespie A, Porth T, Suzuki C, Mahy M, Kasedde S (2014). Epidemiology of HIV and AIDS among adolescents: current status, inequities, and data gaps. J Acquir Immune Defic Syndr.

[3] UNICEF (2011). Opportunity in Crisis: preventing HIV from early adolescence to young adulthood [Internet]. http://www.unicef.org/publications/files/Opportunity_in_Crisis-Report_EN_052711.pdf.

[4] Peralta L, Deeds BG, Hipszer S, Ghalib K (2007). Barriers and facilitators to adolescent HIV testing. AIDS Patient Care STDS.

[5] Kann L, Kinchen S, Shanklin SL, Flint KH, Kawkins J, Harris WA (2014). Youth risk behavior surveillance – United States, 2013. MMWR Surveill Summ.

[6] Hull MW, Wu Z, Montaner JS (2012). Optimizing the engagement of care cascade: a critical step to maximize the impact of HIV treatment as prevention. Curr Opin HIV AIDS.

[7] Emmanuel PJ, Martinez J, Committee on Pediatric AIDS (2011). Adolescents and HIV infection: the pediatrician's role in promoting routine testing. Pediatrics.

[8] Mill JE, Jackson RC, Worthington CA, Archibald CP, Wong T, Myers T (2008). HIV testing and care in Canadian Aboriginal youth: a community based mixed methods study. BMC Infect Dis.

[9] Berendes S, Rimal RN (2011). Addressing the slow uptake of HIV testing in Malawi: the role of stigma, self-efficacy, and knowledge in the Malawi BRIDGE Project. J Assoc Nurses AIDS Care.

[10] Leonard NR, Rajan S, Gwadz MV, Aregbesola T (2014). HIV testing patterns among urban YMSM of color. Health Educ Behav.

[11] Cohen MS, Shaw GM, McMichael AJ, Haynes BF (2011). Acute HIV-1 infection. N Engl J Med.

[12] Branson BM, Owen SM, Wesolowski LG, Bennett B, Werner BG, Wroblewski KE (2014). Laboratory testing for the diagnosis of HIV infection: updated recommendations. http://stacks.cdc.gov/view/cdc/23447.

[13] Philbin MM, Tanner AE, DuVal A, Ellen JM, Xu J, Kapogiannis B (2014). Factors affecting linkage to care and engagement in care for newly diagnosed HIV-positive adolescents within fifteen adolescent medicine clinics in the United States. AIDS Behav.

[14] U.S. Department of Health and Human Services (2014). Guidelines for the use of antiretroviral agents in HIV-1-infected adults and adolescents. Panel on Antiretroviral Guidelines for Adults and Adolescents – A Working Group of the Office of AIDS Research Advisory Council.

[15] Cohen MS, Chen YQ, McCauley M, Gamble T, Hosseinipour MC, Kumarasamy N (2011). Prevention of HIV-1 infection with early antiretroviral therapy. N Engl J Med.

[16] Lamb MR, Fayorsey R, Nuwagaba-Biribonwoha H, Viola V, Mutabazi V, Alwar T (2014). High attrition before and after ART initiation among youth (15–24 years of age) enrolled in HIV care. AIDS.

[17] Belzer ME, Naar-King S, Olson J, Sarr M, Thornton S, Kahana SY (2014). The use of cell phone support for non-adherent HIV-infected youth and young adults: an initial randomized and controlled intervention trial. AIDS Behav.

[18] Calderon Y, Cowan E, Nickerson J, Mathew S, Fettig J, Rosenberg M (2011). Educational effectiveness of an HIV pretest video for adolescents: a randomized controlled trial. Pediatrics.

[19] Choko AT (2011). PLOS Med.

[20] Poulin M (2007). Sex, money, and premarital partnerships in southern Malawi. Soc Sci Med.

[21] Kenya MoH (2014). Kenya HIV prevention revolution road map [Internet]. http://www.nacc.or.ke/attachments/article/418/Kenya_HIV_Prevention_Revolution_Road_Map.pdf.

[22] Peltzer K, Matseke G (2013). Determinants of HIV testing among young people aged 18–24 years in South Africa. Afr Health Sci.

[23] Sommer M (2011). An overlooked priority: puberty in sub-Saharan Africa. Am J Public Health.

[24] Malisha L, Maharaj P, Rogan M (2008). Rites of passage to adulthood: traditional initiation schools in the context of HIV/AIDS in the Limpopo Province, South Africa. Health Risk Soc.

[25] Munthali AC, Zulu EM (2007). The timing and role of initiation rites in preparing young people for adolescence and responsible sexual and reproductive behaviour in Malawi. Afr J Reprod Health.

[26] Skinner J, Underwood C, Schwandt H, Magombo A (2013). Transitions to adulthood: examining the influence of initiation rites on the HIV risk of adolescent girls in Mangochi and Thyolo districts of Malawi. AIDS Care.

[27] Mudege NN, Egondi T, Beguy D, Zulu EM (2012). The determinants of female circumcision among adolescents from communities that practice female circumcision in two Nairobi informal settlements. Health Soc Rev.

[28] Martinez Perez G, Namulondo H, Tomas Aznar C (2013). Labia minora elongation as understood by Baganda male and female adolescents in Uganda. Cult Health Sex.

[29] Corduk N, Unlu G, Sarioglu-Buke A, Buber A, Savran B, Zencir M (2013). Knowledge, attitude and behaviour of boys and parents about circumcision. Acta Paediatr.

[30] Morgan EM (2013). Contemporary issues in sexual orientation and identity development in emerging adulthood. Emerg Adulthood.

[31] Mustanski B, Birkett M, Greene GJ, Rosario M, Bostwick W, Everett BG (2014). The association between sexual orientation identity and behavior across race/ethnicity, sex, and age in a probability sample of high school students. Am J Public Health.

[32] Secor-Turner M, Sieving R, Eisenberg ME, Skay C (2011). Associations between sexually experienced adolescents’ sources of information about sex and sexual risk outcomes. Sex Educ.

[33] Tu W, Batteiger BE, Wiehe S, Ofner S, Van Der Pol B, Katz BP (2009). Time from first intercourse to first sexually transmitted infection diagnosis among adolescent women. Arch Pediatr Adolesc Med.

[34] Young BJ, Furman W, Jones MC (2012). Changes in adolescents’ risk factors following peer sexual coercion: evidence for a feedback loop. Dev Psychopathol.

[35] Decker MR, Miller E, McCauley HL, Tancredi DJ, Anderson H, Levenson RR (2014). Recent partner violence and sexual and drug-related STI/HIV risk among adolescent and young adult women attending family planning clinics. Sex Transm Infect.

[36] Miller E, Decker MR, Raj A, Reed E, Marable D, Silverman JG (2010). Intimate partner violence and health care-seeking patterns among female users of urban adolescent clinics. Matern Child Health J.

[37] Lindberg LD, Santelli JS, Singh S (2006). Changes in formal sex education: 1995–2002. Perspect Sex Reprod Health.

[38] Ott MA, Santelli JS (2007). Abstinence and abstinence-only education. Curr Opin Obstet Gynecol.

[39] Santelli JS (2008). Medical accuracy in sexuality education: ideology and the scientific process. Am J Public Health.

[40] Lindberg LD, Maddow-Zimet I (2012). Consequences of sex education on teen and young adult sexual behaviors and outcomes. J Adolesc Health.

[41] Vivancos R, Abubakar I, Phillips-Howard P, Hunter PR (2013). School-based sex education is associated with reduced risky sexual behaviour and sexually transmitted infections in young adults. Public Health.

[42] Hall KS, Moreau C, Trussell J (2012). Associations between sexual and reproductive health communication and health service use among U.S. adolescent women. Perspect Sex Reprod Health.

[43] Bleakley A, Hennessy M, Fishbein M, Jordan A (2009). How sources of sexual information relate to adolescents’ beliefs about sex. Am J Health Behav.

[44] Jones RK, Biddlecom AE (2011). Is the internet filling the sexual health information gap for teens? An exploratory study. J Health Commun.

[45] Lagus KA, Bernat DH, Bearinger LH, Resnick MD, Eisenberg ME (2011). Parental perspectives on sources of sex information for young people. J Adolesc Health.

[46] Parker R, Ratzan SC (2010). Health literacy: a second decade of distinction for Americans. J Health Commun.

[47] Mavedzenge SN, Luecke E, Ross DA (2014). Effective approaches for programming to reduce adolescent vulnerability to HIV infection, HIV risk, and HIV-related morbidity and mortality: a systematic review of systematic reviews. J Acquir Immune Defic Syndr.

[48] Meyerson B, Barnes P, Emetu R, Bailey M, Ohmit A, Gillespie A (2014). Institutional and structural barriers to HIV testing: elements for a theoretical framework. AIDS Patient Care STDS.

[49] Pant Pai N, Sharma J, Shivkumar S, Pillay S, Vadnais C, Joseph L (2013). Supervised and unsupervised self-testing for HIV in high- and low-risk populations: a systematic review. PLoS Med.

[50] Johnson SB, Blum RW, Giedd JN (2009). Adolescent maturity and the brain: the promise and pitfalls of neuroscience research in adolescent health policy. J Adolesc Health.

[51] Iltis AS (2009). Introduction: vulnerability in biomedical research. J Law Med Ethics.

[52] Miller BB, Cox DN, Saewyc EM (2010). Age of sexual consent law in Canada: population-based evidence for law and policy. Can J Human Sexuality.

[53] Oudekerk BA, Guarnera LA, Reppucci ND (2014). Older opposite-sex romantic partners, sexual risk, and victimization in adolescence. Child Abuse Negl.

[54] Parikh SA (2012). “They arrested me for loving a schoolgirl”: ethnography, HIV, and a feminist assessment of the age of consent law as a gender-based structural intervention in Uganda. Soc Sci Med.

[55] Coleman DL, Rosoff PM (2013). The legal authority of mature minors to consent to general medical treatment. Pediatrics.

[56] English A (2010). State minor consent laws: a summary.

[57] WHO (2013). HIV and adolescents: guidance for HIV testing and counselling and care for adolescents living with HIV.

[58] Earnshaw VA, Smith LR, Chaudoir SR, Amico KR, Copenhaver MM (2013). HIV stigma mechanisms and well-being among PLWH: a test of the HIV stigma framework. AIDS Behav.

[59] Nelson RM, Lewis LL, Struble K, Wood SF (2010). Ethical and regulatory considerations for the inclusion of adolescents in HIV biomedical prevention research. J Acquir Immune Defic Syndr.

[60] Fortenberry JD, Martinez J, Rudy BJ, Monte D (2012). Adolescent Trials Network for HIVAI. Linkage to care for HIV-positive adolescents: a multisite study of the adolescent medicine trials units of the adolescent trials network. J Adolesc Health.

[61] Fortenberry JD (1997). Health care seeking behaviors related to sexually transmitted diseases among adolescents. Am J Public Health.

[62] Nadal KL (2013). That's so Gay! Microaggressions and the Lesbian, Gay, Bisexual, and Transgender Community.

[63] Massey P, Prelip M, Calimlim B, Afifi A, Quiter E, Nessim S (2013). Findings toward a multidimensional measure of adolescent health literacy. Am J Health Behav.

[64] Tylee A, Haller DM, Graham T, Churchill R, Sanci LA (2007). Youth-friendly primary-care services: how are we doing and what more needs to be done?. Lancet.

[65] Futterman DC, Peralta L, Rudy BJ, Wolfson S, Guttmacher S, Rogers AS (2001). The ACCESS (Adolescents Connected to Care, Evaluation, and Special Services) project: social marketing to promote HIV testing to adolescents, methods and first year results from a six city campaign. J Adolesc Health.

[66] Zanoni BC, Mayer KH (2014). The adolescent and young adult HIV cascade of care in the United States: exaggerated health disparities. AIDS Patient Care STDS.

[67] Kapogiannis BG, Legins KE, Chandan U, Lee S (2014). Evidence-based programming for adolescent HIV prevention and care: operational research to inform best practices. J Acquir Immune Defic Syndr.

[68] Coeytaux K, Kramer MR, Sullivan PS (2014). HIV testing among United States high school students at the state and national level, Youth Risk Behavior Survey 2005–2011. Springerplus.

[69] Godia PM, Olenja JM, Lavussa JA, Quinney D, Hofman JJ, van den Broek N (2013). Sexual reproductive health service provision to young people in Kenya; health service providers’ experiences. BMC Health Serv Res.

[70] MacPhail CL, Pettifor A, Coates T, Rees H (2008). “You must do the test to know your status”: attitudes to HIV voluntary counseling and testing for adolescents among South African youth and parents. Health Educ Behav.

[71] Renju J, Andrew B, Nyalali K, Kishamawe C, Kato C, Changalucha J (2010). A process evaluation of the scale up of a youth-friendly health services initiative in northern Tanzania. J Int AIDS Soc.

[72] Parker L, Maman S, Pettifor A, Chalachala JL, Edmonds A, Golin CE (2013). Barriers to provider-delivered sexual behavior counseling for youth living with HIV/AIDS in the Democratic Republic of the Congo. J HIV AIDS Soc Serv.

[73] Mathews C, Guttmacher SJ, Flisher AJ, Mtshizana YY, Nelson T, McCarthy J (2009). The quality of HIV testing services for adolescents in Cape Town, South Africa: do adolescent-friendly services make a difference?. J Adolesc Health.

[74] Ntsepe Y, Simbayi LC, Shisana O, Rehle T, Mabaso M, Ncitakalo N (2014). Perceptions about the acceptability and prevalence of HIV testing and factors influencing them in different communities in South Africa. SAHARA J.

[75] Shaw D (2009). Access to sexual and reproductive health for young people: bridging the disconnect between rights and reality. Int J Gynaecol Obstet.

[76] Hainsworth G, Engel DM, Simon C, Rahimtoola M, Ghiron LJ (2014). Scale-up of adolescent contraceptive services: lessons from a 5-country comparative analysis. J Acquir Immune Defic Syndr.

[77] Laski L, Wong S (2010). Addressing diversity in adolescent sexual and reproductive health services. Int J Gynaecol Obstet.

[78] Deogan C, Ferguson J, Stenberg K (2012). Resource needs for adolescent friendly health services: estimates for 74 low- and middle-income countries. PLoS One.

[79] Naidoo P, Chirinda W, McHunu G, Swartz S, Anderson J (2014). Social and structural factors associated with vulnerability to HIV infection among young adults in South Africa. Psychol Health Med.

# Sub article: Providing comprehensive health services for young key populations: needs, barriers and gaps

**DOI:** 10.7448/IAS.18.2.19833

**Published:** 2015-02-26

**Authors:** Sinead Delany-Moretlwe, Frances M Cowan, Joanna Busza, Carolyn Bolton-Moore, Karen Kelley, Lee Fairlie

**Affiliations:** 1 Wits RHI, Faculty of Health Sciences, University of the Witwatersrand, Johannesburg, South Africa; 2 CeSHHAR Zimbabwe, Harare, Zimbabwe;; 3 Department of Infection and Population Health, University College London, London, United Kingdom; 4 Department of Population Health, London School of Hygiene and Tropical Medicine, London, United Kingdom; 5 Centre for Infectious Disease Research in Zambia (CIDRZ), Lusaka, Zambia

**Keywords:** adolescent, youth, injecting drug use, MSM, sex workers, risk, integrated services

## Abstract

**Introduction:**

Adolescence is a time of physical, emotional and social transitions that have implications for health. In addition to being at high risk for HIV, young key populations (YKP) may experience other health problems attributable to high-risk behaviour or their developmental stage, or a combination of both.

**Discussion:**

We reviewed the needs, barriers and gaps for other non-HIV health services for YKP. We searched PubMed and Google Scholar for articles that provided specific age-related data on sexual and reproductive health; mental health; violence; and substance use problems for adolescent, youth or young sex workers, men who have sex with men, transgender people, and people who inject drugs.

**Results:**

YKP experience more unprotected sex, sexually transmitted infections including HIV, unintended pregnancy, violence, mental health disorders and substance use compared to older members of key populations and youth among the general population. YKP experience significant barriers to accessing care; coverage of services is low, largely because of stigma and discrimination experienced at both the health system and policy levels.

**Discussion:**

YKP require comprehensive, integrated services that respond to their specific developmental needs, including health, educational and social services within the context of a human rights-based approach. The recent WHO *Consolidated Guidelines on HIV Prevention, Diagnosis, Treatment and Care for Key Populations* are an important first step for a more comprehensive approach to HIV programming for YKP, but there are limited data on the effective delivery of combined interventions for YKP. Significant investments in research and implementation will be required to ensure adequate provision and coverage of services for YKP. In addition, greater commitments to harm reduction and rights-based approaches are needed to address structural barriers to access to care.

## Introduction

Young people aged 10–24 represent at least a quarter of the world's population, but are disproportionately affected by HIV [1,2]. Globally, the health of young people is important as it is an indicator of future population health, and also social and economic development. Although the rate of new HIV infections has declined or stabilized in many populations, over a third of new HIV infections continue to occur among the 15- to 24-year age group [3]. HIV risk and prevalence are not uniform; people who sell sex (SW), inject drugs (PWID), men who have sex with men (MSM) and transgender people (TG) have been shown to have higher risks for HIV infection than the general population [4–6]. Many practice more than one risk behaviour. For example, young MSM may also use drugs and sell sex for drugs, emphasizing the need for comprehensive, integrated health services. Key populations (KP) contribute disproportionately to HIV transmission dynamics within countries, with recent estimates suggesting that 50% of new HIV infection occur among these populations [7]. In addition to their high-risk behaviour, KP frequently experience significant stigma, discrimination and violence, which further limits their ability to adopt preventive behaviours and access health services [8]. In YKP, the effects of stigma, discrimination and violence are exacerbated by policy and legal barriers related to the age of consent for sex as well as selected medical interventions, further limiting access to a range of health services [7]. As a consequence, YKP are frequently a hidden population, and reliable and representative epidemiological data on their health are scarce [7]. This paucity of data often leads to neglect of their specific needs by programmes designed either for young people more generally, or for adult KP. Failure to identify the comprehensive health needs of YKP, and their specific barriers to care, has the potential to undermine the success of HIV prevention programmes targeted at these populations [9].

### Conceptual framework for adolescent health

While YKP require specific interventions for the prevention, treatment and care of HIV, YKP also require non-HIV-related health services that respond to the health needs of their particular developmental life stage. The complex physical, psychological, emotional and social changes that take place during adolescence have immediate and long-term implications for individuals [9]. For example, the onset of puberty is linked to the initiation of sexual activity, and subsequent exposure to the risk of pregnancy and STIs, including HIV. Awareness of sexual orientation emerges during this period. Mental health disorders also emerge during the second decade of life. High rates of self-harm are observed in young people, and suicide is a leading cause of death [10]. Increased risk-taking and a heightened sensitivity to peers may influence adolescent experimentation with substance use. Although risk-taking is considered a normal part of adolescent development, risk-taking by YKP can have serious adverse consequences. More than any other life stage, adolescent health is strongly determined by social context. Both structural determinants of health (e.g. national wealth, income inequality, access to education and health services, employment opportunities and gender inequality) and more proximate determinants of health (e.g. connectedness of adolescents to family and school) affect health-related behaviour and outcomes during adolescence [11]. It is not surprising therefore that poor sexual and reproductive health (SRH), mental health disorders, violence and injury, and substance use account for the majority of disability and disease experienced by people aged 10–24 globally [12]. Sawyer and colleagues have proposed a conceptual framework to enhance our understanding of adolescent health and development (Figure 1) [9]. The horizontal axis describes a life-course perspective from the pre-conceptual and prenatal period through to adulthood. The vertical axis describes the social determinants of health and the pathways by which these influence health outcomes. The nexus of these two axes is the period of adolescence, a time of enormous physical, emotional, mental and social transition. Policy and programmatic responses to adolescent health operate along the vertical axis, but should not be developed without an appreciation for the importance of adolescence within a life-course perspective [9]. Guided by this conceptual approach, we review the needs, barriers and gaps for non-HIV-related services for YKP as part of a special series of papers on YKP.

**Figure 1:** Conceptual framework of adolescent health, adapted from Sawyer et al. [9].

## Methods

We focused our review on those non-HIV-related services that are developmentally relevant to this population based on the conceptual framework outlined above, and/or included as recommended interventions in the recently published WHO *Consolidated Guidelines on HIV Prevention, Diagnosis, Treatment and Care for Key Populations* (Table 1). We undertook a targeted, web-based search to identify age-specific data on the health needs and barriers to care for YKP aged 10–24. Using PubMed and Google Scholar, we focused on articles published in English since 1990, with a particular emphasis on systematic reviews and more recent publications. In order to identify age-specific data or references we used the key words “young,” “youth,” “adolescent,” and “age” in combination with search terms for each KP (e.g. “MSM,” “men who have sex with men,” “gay,” “bisexual”) and for each health topic (e.g. “condoms,” “unprotected sex,” “STI,” etc.). Abstracts were retrieved and read, and if relevant age-related data were provided, full-text articles were retrieved. Over a 2000 abstracts were identified in the initial searches, but far fewer contained age-specific information relevant to YKP and 110 articles with age-specific information were included. Due to the paucity of age-specific data on YKP, articles that referred to KP or adolescent populations were used to supplement searches.

**Table 1:** Summary of relevant WHO recommendations for linked services key populations [7]

**HIV Prevention**
**Post-exposure prophylaxis (PEP)** should be available to all eligible people from key populations on a voluntary basis after possible exposure to HIV.
All pregnant women from key populations should have the same access to services for **prevention of mother-to-child transmission (PMTCT)** and follow the same recommendations as women in other populations.
**Harm reduction**
All people from key populations with **harmful alcohol or other substance use** should have access to evidence-based interventions, including brief psychosocial interventions involving assessment, specific feedback and advice.
**Prevention and management of co-infections and co-morbidities**
Key populations should have the same access to **hepatitis B and C** prevention, screening and treatment services as other populations at risk of or living with HIV.
Routine screening and management of **mental health disorders** (depression and psychosocial stress) should be provided for people from key populations living with HIV to optimize health outcomes and improve their adherence to ART. Management can range from counselling for HIV and depression to appropriate medical therapies.
**Sexual and reproductive health**
Screening, diagnosis and treatment of **sexually transmitted infections** should be offered routinely as part of comprehensive HIV prevention and care for key populations.
People from key populations, including those living with HIV, should be able to experience full, pleasurable sex lives and have **access to a range of reproductive options**.
**Abortion laws and services** should protect the health and human rights of all women, including those from key populations.
It is important to offer **cervical cancer screening** to all women from key populations.
(Note: for adolescent populations, HPV vaccination is an additional recommendation for prevention of HPV-associated disease including anogenital cancers).
It is important that all women from key populations have the same support and access to services related to **conception and pregnancy care**, as women from other groups.
**Critical enablers**
**Laws, policies and practices** should be reviewed and, where necessary, revised by policymakers and government leaders, with meaningful engagement of stakeholders from key population groups, to allow and support the implementation and scale-up of health care services for key populations.
Countries should work towards implementing and enforcing **anti-discrimination** and protective laws, derived from human rights standards, to eliminate stigma, discrimination and violence against people from key populations.
Health services should be made **available, accessible and acceptable** to key populations, based on the principles of medical ethics, avoidance of stigma, non-discrimination and the right to health.
Programmes should work towards implementing a package of interventions to enhance **community empowerment** among key populations.
**Violence** against people from key populations should be prevented and addressed in partnership with key population-led organizations. All violence against people from key populations should be monitored and reported, and redressal mechanisms should be established to provide justice.

## Results

Poor SRH outcomes, mental health disorders, violence and injury, and substance use account for the majority of disability and disease experienced by young people aged 10–24 globally [12]. We summarized the findings of our review which demonstrate overall that YKP experience an even higher burden of disease than older KP, as well as a higher burden of disease than their age peers in the general population.

### Unprotected sex

Unprotected sex is an important risk factor for negative health outcomes among young people, accounting for 4% of disability-adjusted life years (DALYs) in those aged 10–24 [12]. Several studies have shown younger age is associated with more frequent unprotected sex among KP [13], often due to lower levels of education, knowledge, and risk perception [13–15]. Young key populations (YKP) may also be unaware of where to access condoms and other contraception. The combination of low condom self-efficacy and more frequent sex or change in sexual partners also puts YKP at higher risk of sexually transmitted infection (STI) [16–18]. For many, the ability to negotiate safer sex with partners is limited by imbalances in relationship power, compounded by adolescent aspirations for love and intimacy. For example, although condom use rates may be higher among sex workers than the general population, several studies have shown how younger sex workers may be less experienced than older sex workers with condom negotiation, and more vulnerable to being forced to have sex without a condom either by clients or managers [19,20]. Unprotected sex may also be associated with expressions of intimacy or trust, with higher rates for sex with primary partners, who are often older and thus have had a longer period of potential exposure to HIV and other STI [21–23]. In one study of young MSM aged 16–20, considering a relationship to be serious was associated with an eight-fold increase in the rate of unprotected sex [24]. For TG women, condom negotiation may be more difficult given their female gender identity and socially constructed role, and young TG people may be more likely to have unprotected sex to validate their gender identity [13]. Among young PWID, low rates of condom use are often associated with other high-risk practices such as needle sharing or smoking drugs together [23]. Among young PWID, there are gender differences in risk with higher rates of unprotected sex and sexual risk observed in young women [25]. Given the lower levels of condom use among YKP, the potential for negative health outcomes in this population is high.

### Sexually transmitted infections

The prevalence of STIs is also higher in YKP than among older KP peers. Studies show elevated rates of syphilis [26], gonorrhoea [27], chlamydia [28,29] and herpes simplex [30] among YKP compared with adult KP. Young MSM and TG may be more likely to have anal or rectal infections that are asymptomatic and/or remain undiagnosed [31]. STIs are also more common in those populations with more than one risk behaviour. For example, a prospective study among PWID in British Columbia, Canada, found that incident STIs were more frequent among those involved in the sex trade compared with those who did not sell sex, over a three-year period [32]. In addition to causing significant morbidity and mortality, STIs also increase the risk of HIV transmission.

Two of the viral STIs associated with cancer outcomes are now vaccine preventable. HPV is a common STI that causes cervical and other anogenital cancers. A high prevalence of HPV infection has been observed in adult KP. In a study of anal HPV prevalence and risk factors among men in Brazil, Mexico and the United States, among MSM, younger age was associated with increased prevalence of any anal canal HPV [33]. YKP may be exposed to HPV earlier and may be more at risk of developing of pre-neoplastic and neoplastic lesions in later life, especially if they are co-infected with HIV. Although access to HPV vaccination has expanded significantly in the past five years, young MSM or TG women may not benefit from vaccination programmes targeted at young girls, and may not receive the benefit afforded to heterosexual men through herd immunity [34].

YKP may be at increased risk for viral hepatitis. Hepatitis C virus (HCV) incidence in young PWID is high, raising concerns about the prevention and control of an expanding epidemic in young people [35]. A study on young PWID in Afghanistan showed that risk of HCV infection increased with each additional year of injecting among young PWID [36]. Young female PWID were reported to have a higher incidence of HCV, associated with higher risk injection practices, when compared to young men [37]. Young MSM are also at increased risk of viral hepatitis A and B [38]. YKP would benefit from vaccination against hepatitis A and B in settings where universal childhood vaccination is not routine. Where vaccination is offered to MSM, coverage is still relatively low, although promising data from several studies show that vaccine uptake is associated with younger age [39].

### Reproductive health

In addition to STIs, a frequent outcome of unprotected sex in female YKP is unintended pregnancy, which during adolescence can pose particular risks to both mother and infant [40]. Data on pregnancy intentions, outcomes, and use of contraception or prevention of mother-to-child-transmission (PMTCT) in YKP are limited. Studies in adult female sex workers show that pregnancy is frequently unwanted, termination of pregnancy is common and contraceptive use, including of emergency contraception, is low, suggesting significant unmet needs [41–43]. A recent study among Chinese adolescent sex workers showed that a quarter had never used a modern contraceptive method [44]. The main method of pregnancy prevention in this population was condoms, although condom use was often inconsistent. Half of those interviewed reported a previous induced abortion, although only a third of those had sought care from public sector services. These data are supported by another study in Chinese sex workers showing that younger sex workers were less likely to terminate their pregnancy, but those that did were more likely to seek termination from informal providers [45]. For HIV-positive YKP, PMTCT is a priority. While few data are available on access to PMTCT in YKP, given high rates of HIV infection in this population, there is likely to be a significant need. In a study of PWID in Ukraine, PWID were more likely than non-PWID to be diagnosed during labour and to have more advanced HIV disease, but less likely to receive prophylaxis or HAART to prevent vertical transmission. As a consequence, vertical transmission rates in this population were higher than in the general population [46].

Young TG have specific health needs related to their gender identity. Hormone therapy may have significant benefits for TG people, but access is frequently limited by cost or provider attitudes. As a consequence, some TG may seek hormone therapy from the non-medical sources [47], despite potential side effects from unmonitored treatment including overdose [48], or the risk associated with injecting hormones or silicone [49]. In some cases, young TG may engage in sex work to fund treatments [50]. Services for YKP need to be able to provide reliable, evidence-informed information regarding TG-specific medical and surgical procedures.

### Sexual assault

YKP are more likely to require sexual assault services including access to post-exposure prophylaxis (PEP). While no age-specific data were provided, a recent systematic review of the prevalence and correlates of violence against sex workers estimated that the lifetime prevalence of any violence ranged from 45% to 75% [51]. Young trafficked sex workers may have experienced violent rape in order to coerce them to sell sex [18,52]. Young male sex workers are not immune, and studies show that they also experience verbal, physical and sexual abuse [53]. In a study of MSM in Thailand, 18% had experienced forced sex, the majority by someone they knew, and the forced sex occurred more than once, with the first experience occurring during adolescence [54]. While estimates of the prevalence of sexual assault among PWID are limited, a recent study among women aged 16–29 attending family planning services in Pennsylvania, USA, showed 11% of women had experienced sexual violence in the previous three months, and that sexual assault was associated with injection drug use, their own or their partners [55]. Experiences of violence are strongly associated with increased risk behaviour and risk for HIV [18,56,57], as well as other negative SRH outcomes [58,59]. Interventions that address sexual assault and that provide access to PEP are a critical component of a package of services for YKP [7].

### Mental health

While emergence of mental health problems in the second decade of life is common, data suggest that YKP experience higher rates of mental health problems when compared with their same age counterparts in the general population, or older key population peers. For example, a study of young SW in China showed that those younger than age 20 experienced the highest rates of depression, suicide and substance use compared to those older than 20 [60]. A study in lesbian, gay, bisexual, and transgender (LGBT) youth in the US observed higher rates of mental disorders in this population compared to the general population [61]. Major depression, personality and substance use disorders are more common in young PWID [62], with higher rates of mental health disorders observed in young women [63].

Among the factors that influence poor mental health in young KP, stigma, discrimination, social exclusion and victimization are substantial contributors. For example, a study among young PWID in Russia showed that a large proportion had experienced discrimination resulting in loss of jobs, lack of access to health care and being forced from their family homes. Over one-third had clinical depression [64]. A study in Chinese sex workers showed that the majority had high levels of self-stigma, and that this was significantly associated with poor mental health [65]. In a US study of young MSM, experiences of victimization were strongly associated with a syndemic of depression, substance use, risky sex and intimate partner violence; these factors were also strongly associated with an increase in suicide attempts [66]. These findings were echoed in a global study of MSM in 151 countries [67], and a study in young TG women [68]. Violence experienced at the hands of family, partners, clients or police is associated with increased reporting of poor mental health outcomes [69–72].

### Substance use

Substance use and experimentation are common in adolescence, but evidence suggests that YKP are more likely to initiate substance use at an earlier age, to engage in polysubstance use and to experience more rapid increases in substance use over time [73,74]. This is of particular concern given the findings that substance use in adolescence may be more harmful to brain function and behaviour while the brain is still developing [75]. YKP may frequent social spaces where alcohol and drug use are tolerated and consumption normalized. YKP may initiate substance use to self-medicate against the anxiety associated with group behaviours, or associated negative experiences [76,77]. A study in Australian LGBT youth highlighted a higher prevalence of alcohol and drug use in this population compared to the general population. In this study, alcohol use was significantly higher in those younger than 18, and those who believed that homophobia influenced alcohol and drug use were significantly more likely to use alcohol or drugs [78]. Substance use may increase sexual desire, lower inhibitions and impair decision-making. A study of young TG women in the US documented that this group was significantly less likely to use condoms with main partners while under the influence of drugs or alcohol. Substance use may reduce an individual's concerns about safe sex in the face of other, more important desires and immediate priorities [22]. Several other studies among YKP have shown that drinking beforehand can be associated with unprotected sex and injecting behaviours [76,79,80].

The risks associated with injecting drug use are disproportionately high. Problem drug use often starts with recreational drug use, and studies show that the transition from non-injecting to injecting drug use in young people is rapid and high [77]. Early initiation of injecting drug use is associated with younger age and being female [25,81]. Young PWID are frequently initiated into drug use by peers [82,83]; young women more often are initiated by a sex partner [77,84]. A study in Ukraine reported that in 56% of boys and 72% of girls, the first injection was unplanned and often occurred after exposure to injecting among friends, with around 32% of girls initiated by their sexual partners [85]. Young PWID are more likely to inject in groups, and develop rituals associated with injecting that expose them to sharing of non-sterile equipment. Studies in young PWID show more frequent sharing of equipment, more frequent injecting and injecting in public spaces [86,87]. Young PWID are more likely to practice unprotected sex, have increased numbers of partners, or trade sex for drugs than their older peers [88,89]. Young PWID may also be less likely to engage in care for their addiction, and are also more likely to report relapse after treatment [72,90]. Young PWID have much higher mortality rates than their peers in the general population, associated with overdose or injury [91]. They have a substantial need for harm reduction and addiction treatment services, as well as links to services to address their integrated health and social needs.

### Educational, vocational and social support

In addition to health care, YKP frequently require social support because of their life stage as well as socio-structural factors that influence their behaviour. YKP may be orphaned or rejected by their families, experiencing homelessness, food insecurity and economic instability [56,92,93], and may often prioritize food, shelter and money over health [94]. Female YKP may have concerns about the welfare of their own children [95]. Access to social support and benefits is therefore essential to reducing their risk. Completion of education, initiating employment and transitioning out of the childhood home may be considered a normal part of adolescent development, yet YKP may not experience these positively. For example, school bullying and victimization may severely impair educational attainment and future employment opportunities of young MSM and TG [96]. For some, low educational attainment may become a reason to sell sex [97]. Young PWID who drop out of school may be at higher risk for HIV [15]. Linkage to educational and vocational support interventions are an important part of a developmentally appropriate response to YKP, and while not directly linked to HIV programme activities educational and vocational interventions may be critical enablers for HIV programme success in this age group.

### Barriers to care

Adolescence is marked by high rates of attrition along the continuum of HIV prevention, diagnosis and treatment services. YKP are less likely to be engaged in care [98], and coverage of services is low. While data are limited for YKP, evidence from young adult and adult populations shows that KP experience poor access to condoms and HIV testing [99], may present later for HIV treatment [100] and have lower rates of adherence [101], viral suppression [102] and retention in care [103]. PMTCT outcomes and access to linked mental health, substance use and SRH services are poor [46,104]. PWID may experience difficulty in accessing safe injecting equipment or treatment for dependence. Using the conceptual framework (Figure 1), reasons for poor access to care can be categorized as individual-level, health-system-level or structural-level barriers, and are common to all YKP.

### Individual-level barriers to care

Low levels of education and HIV knowledge or risk perception are associated with low uptake of HIV services [13]. YKP with less formal education and/or less sex education may be less familiar with what constitute safe sex or safe injection practices. YKP with internalized stigma experience more social isolation and are less able to ask trusted adults for support in decision-making [105]. They may also experience bullying by older KP [106]. YKP who have experienced poor mental health, violence or low levels of social support may have lower levels of self-efficacy for health-seeking [107].

### Health-system-level barriers to care

Perhaps the most significant barrier to health-seeking among YKP is the experience of stigma, discrimination or victimization at the hands of health care providers (HCP). In a study of young migrant sex workers in North Vietnam, despite health care being available, the young women perceived the stigma attached to sex work as a barrier to receiving health care, and preferred to receive health education and care from peers [52]. In another study involving male, female and TG sex workers in Africa, denial of treatment for injuries following physical assault or rape and general hostility from public sector providers were common [108]. Similar experiences were reported by PWID in India [70]. Younger PWID expressed a preference for syringe-dispensing machines over staffed needle exchange programmes because of their desire to hide their identity or because they did not like the way they were treated at staffed services [109]. Concerns about privacy and confidentiality are an important barrier to care. In a US-based study, LGBT youth expressed greater concerns about confidentiality and were less likely to seek care from school-based services compared to heterosexual peers [110]. In addition to concerns about poor attitudes, HCP may not have sufficient skill, competence or training to deal with the specific health and social needs of YKP. A US survey of HCP reported that the majority of respondents would not regularly discuss sexual orientation, sexual attraction or gender identity while taking a sexual history from a sexually active adolescent. The majority of physicians did not believe that they had all the necessary skills to address issues of sexual orientation with adolescents [111]. Studies reveal that provider willingness to answer questions, their respect for and understanding of adolescents and the responsiveness of the social and physical environment towards youth are all associated with young people's intention to seek and engage in care [112]. Negative experiences with providers may prompt YKP to seek care from non-conventional services [47]. For YKP, cost and waiting time are also barriers to care [103]. Young people are less likely to have access to ready cash and may have competing demands or less control over their time. In several studies, YKP highlight the importance of integrated services that address their multiple health needs [113]. Lack of service integration adds time and cost to clinic visits and may be a further barrier to care. In many cases, services for KP may not be sufficiently “youth-friendly.” Providers may not have an appreciation for the specific health and communication needs of YKP. YKP may experience discomfort when seeking care with adults. Location and transport may also be a barrier to care. Larger, more formal venues can enhance prevention initiatives, including on-site services. Geographical targeting of services for YKP can be complicated by the social and sexual networking patterns of YKP who may find partners through the Internet, meet them in informal venues, and generally be more mobile. This can increase the risk involved and makes it harder for services to identify and reach them [114].

### Structural-level barriers

Criminalization reduces YKP's control over their behaviour, impedes their access to health services and obstructs health-service provision and legal protection. In many settings, YKP are criminalized for their behaviour/s and risk incarceration [115,116]. Even in settings where activities are not criminalized, they may experience significant stigma, discrimination or police harassment, as a result of both their group identity and their age [117–119]. Studies of MSM in African countries where homosexuality is criminalized demonstrate how criminalization makes MSM more vulnerable to violence and less able to access health care or preventive services [99,119,120]. YKP are vulnerable to harassment and exploitation by the police, and may go to substantial lengths to avoid police. A mapping study in Canada showed a significant geographic relationship between a heavily concentrated core area of health and syringe availability and avoidance of these settings by substance using street-based sex workers due to policing; this correlation was strongest among younger women [121]. Several studies have highlighted how police arrest YKP for carrying drug paraphernalia or confiscate it without arrest [70,122]. Arrest and detention are frequently associated with police beatings; a Thai study among young PWID showed that younger age was associated with more frequent reports of police beating [118]. In addition to physical violence, YKP are also vulnerable to sexual coercion and violence at the hands of authorities [123].

Access to care is further confounded by the legal status of YKP as minors. In many countries, adolescents require parental permission to access testing, treatments or procedures. This is a particular problem for minors who do not live with their parents or do not wish to disclose their behaviour to them. For example, young women seeking contraception or safe abortion services are likely to seek care outside of conventional health services [45]. In many countries where opioid substitution services are available, age restrictions are placed on them [124]. In some settings, HCP are legally obliged to report underage sex or other illegal activities. This may compromise disclosure and provision of adequate care for risky behaviours. Age restrictions are also placed on access to housing and other social services which may be needed.

An absence of clear legal status may also be a barrier for access to health services. TG people may experience significant barriers to obtaining services, legal entitlements and legal protection because the gender assignment on their administrative documents may be in conflict with their gender identity [113]. Migrant YKP may also not have adequate legal documentation of citizenship, and may not be able to access services in their host country. As adolescents, they are often dependent on parents for the provision of information necessary for citizenship or travel documentation. The absence of appropriate official documentation may make them vulnerable by limiting their access to health and social services and benefits that they might otherwise be entitled to.

Many YKP experience additional stigma and discrimination associated with their racial or ethnic identity, in addition to their group identity. There is overwhelming evidence from the United States that young Black MSM have the highest concentration of HIV of any sub-population despite little evidence of higher risk behaviour. Instead, social and structural factors act as barriers to health care access [125]. In other settings, YKP from either indigenous or migrant populations are marginalized and have limited access to health services [87].

## Discussion

Despite the paucity of age-specific data for YKP, this review confirms that in addition to interventions for the prevention, treatment and care of HIV, YKP also require other, non-HIV-related health services that respond to their significant health and development needs as adolescents. The WHO recommendations for a comprehensive package of services that includes SRH services and care for mental health disorders is an important first step in recognizing the impact of these other health concerns on the success of HIV prevention, treatment and care interventions [7]. These guidelines specifically recognize the health and developmental needs of YKP, and provide commentary on specific considerations for the delivery of health sector interventions to YKP.

The next priority is to ensure that these recommendations are implemented at scale and with sufficient intensity to ensure an impact on the HIV epidemic, and the health of YKP. While there is substantial evidence for effective interventions to prevent and treat HIV infection in adults, less is known about the delivery of these interventions to adolescents [126]. Current coverage of services for YKP is generally low [127,128], and consideration of optimal service delivery models that respond to current barriers to care are now a priority. The requirement to make services accessible, acceptable and available to YKP provides an opportunity to evaluate interventions aimed at addressing health system barriers to care. Beyer and colleagues have proposed three models of service provision for KP: integrated models of care, stand-alone models of care or hybrid models of service provision [8]. Integrating HIV and related service provision for YKP into primary health care (PHC) offers significant potential for expanding coverage and access to care for YKP, and may be the only option for service delivery in some settings. Integrated models have the potential to address several of the health systems barriers. Sensitization of services and training of all staff in facilities, not just HCP, is a potentially powerful structural intervention to enhance the effectiveness of HIV programmes for YKP and reduce stigma more generally. There are a number of positive approaches to stigma reduction, with growing experience on how to work with HCP and communities to reduce anticipated and enacted stigma [129]. There is accumulating evidence to suggest that interventions using a combination of sensitization and participatory activities can reduce HIV stigma in health care [130,131] and community settings [132,133]. A recent systematic review identified 48 evaluations in which HIV-related stigma was assessed as an outcome [134]. While the studies found that information, skills building, counselling and PLHIV testimonials were associated with less stigmatizing attitudes among participants, the evidence base had many gaps. Training and sensitization of HCP to the needs of MSM has also been shown in a study in Kenya to reduce homophobic attitudes up to three months after training [135]. In addition to stigma reduction interventions, initiatives to make PHC more “adolescent and youth-friendly” are likely to benefit the subset of YKP. Evidence from several systematic reviews confirms that implementation of a combination of interventions, including training of HCP, outreach activities and out-of-facility services tailored to context and target population, demonstrated some impact on uptake of health services by young people [126], although training of service providers in adolescent-friendly service provision alone appears to be less beneficial [128].

Peer approaches are potentially a critical component of services for YKP, given the particular developmental susceptibility of adolescents to peer influence. Peers are in a unique position to identify and reach out to YKP who may be experiencing barriers to health care through lack of knowledge, risk perception or self-efficacy. Evidence from a systematic review of interventions to improve linkage and retention in HIV care in low- and middle-income countries supports integrating formalized care with peer support to increase the uptake of HIV services, although data on adolescents are limited [136]. While peer interventions for YKP have been found to be positively associated with increased knowledge and condom use in some programmes [13], they are optimal when included as part of a comprehensive empowerment approach [137]. There is growing evidence that empowerment approaches for SW in particular improve HIV programme outcomes [138]. In addition, empowerment approaches are likely to have benefits for other health challenges, particularly violence. Evidence from programme assessments show that it is possible to prevent violence using empowerment approaches, with some interventions achieving significant effects within programme timeframes [139]. Integrated services delivered at scale offer a significant platform for the delivery of community mobilization and empowerment interventions. Despite the growing evidence that empowerment approaches produce health benefits, effective implementation of empowerment processes within many settings, particularly in Africa, has been limited [140]. Challenges to the sustainability of empowerment interventions include lack of social cohesion within transient communities, limited capacity and resources, and variable commitment of programmers to empowerment interventions. Given these challenges, and the fact that integrated models may not sufficiently address the barriers presented by lack of privacy and accessibility, cost or waiting times, or the need for access to a range of non-health services, alternative models warrant further exploration.

While there is a precedent for stand-alone models of service delivery for KP [141], they may not be suitable in many settings. While these services may be able to provide KP-sensitive services, they may also increase stigma and marginalization, and provide targets for attack. An alternative to stand-alone facilities are out-of-facility-based delivery strategies. Currently data are limited on the benefits of out-of-facility-based approaches to health care delivery among adolescents, although two reviews suggest that services delivered through mixed-use youth centres are not well-used or particularly effective for adolescents in the general population [128]. The authors do note, however, the absence of studies or evaluations examining outcomes among vulnerable or marginalized adolescents. Programmatic experience suggests that drop-in centres provide a valuable opportunity to offer a range of services specific to the needs of YKP. For example, as an alternative to integrating adolescent PWID into programming targeted at adult injectors, who can appear threatening and model harmful behaviours, Moldovan NGOs established drop-in centres welcoming adolescents with overlapping risks, including those living on the street, injecting drugs or involved in sex work. A case management approach then linked individuals to a network of health and social services [142]. Linkage to non-health services is generally valued by YKP [103,113], and may even encourage retention in care. Medical and food incentives have been found to increase retention in care prior to antiretroviral treatment initiation [136], emphasizing the importance of non-HIV-related service delivery provision. Other out-of-facility options include the training of pharmacist to counsel and provide adolescents with appropriate needle exchange and drug substitution services [143]. Work place policies have been used with particularly good effect in sex work establishments in Asia; however, there is less evidence for how these policies may benefit YKP [13]. In these cases, strong linkages with child protection services ensure enforcement of anti-trafficking laws. While schools are an important venue for the delivery of health education, they may not be an optimal for the delivery of services to YKP and should be used to complement, not replace, health care services for adolescents located outside schools [126].

Internet-based interventions represent a different type of out-of-facility service. The rapid expansion of access to the Internet and social media in the past two decades, even in low- and middle-income countries, through mobile phone technology, represents a significant opportunity to engage with previously hidden populations, or those that are socially or geographically isolated [144,145]. The Internet provides a novel way to expand access to standardized information, to build virtual communities of supportive peers and to link YKP to services. There is accumulating evidence of the acceptability of delivering digital-based media interventions to adolescents, KP or YKP in settings in North America [146–148], South America [149], Asia [150,151] and Africa [152], although there is less evidence on the impact of these services on longer term health outcomes [153]. A recent review evaluated the impact of digital-media-based interventions on sexual health knowledge, attitudes and/or behaviours of adolescents in the general population aged 13–24 [154]. Of the ten studies reviewed, six studies increased knowledge of HIV, STI or pregnancy. A recent study among young MSM showed that an Internet-based, peer-led social media HIV prevention intervention can increase community cohesion and uptake of HIV services [155]. These and other evaluations of Internet interventions for YKP show positive short-term outcomes for health. Given initial findings from this and other similar programmes, further evaluation is needed to gauge the potential benefit of these programmes on health outcomes over a longer period [156].

Hybrid models that combine the reach of services integrated at PHC level with the peer-based, outreach and empowerment approaches offered by more flexible community-based NGOs are probably the optimal model of service delivery. Ultimately, decisions about service delivery models need to be informed by user preferences, and they need to take into account considerations that are context-specific and address the age-based needs of YKP, as well as respond to their specific risk behaviours, the epidemic setting and the social, legal and political complexities associated with service delivery for this group. To this end, efforts should be focused on making YKP more visible through research and monitoring, so that their needs are recognized and prioritized by public health systems. While the provision of linked non-HIV-specific services requires significant investment and innovation, significant gains in coverage can be achieved with modest increases in resources [157]. Finally, given the strong influence of socio-structural factors on adolescent health, coupled with the fact that many factors that influence YKP's risk are outside of their immediate control, interventions that address the structural barriers to care are a critical part of an effective HIV response for YKP. In addition to changes in laws and policies that promote stigma and discrimination, specific interventions that address the age of consent are more essential for this age group.

## Conclusions

Despite the dearth of age-specific data, YKP have significant non-HIV-related health needs, and face significant obstacles to accessing care as a result of their age and membership of KP. While YKP face significant hardship and risk, they also represent the greatest hope for reducing the harms associated with their behaviours, and preventing new HIV infections. Now that normative guidance exists for the optimal set of interventions for KP, priority needs to be placed on evaluating optimal approaches for the delivery of a comprehensive package of care of YKP. Investments in providing linked, non-HIV but related services that also address critical enablers of programmes are likely to have significant benefits for HIV prevention across all populations.

## References

[1] WHO (2009). Global health risks: mortality and burden of disease attributable to selected major risks.

[2] Idele P, Gillespie A, Porth T, Suzuki C, Mahy M, Kasedde S (2014). Epidemiology of HIV and AIDS among adolescents: current status, inequities, and data gaps. J Acquir Immune Defic Syndr.

[3] Kasedde S, Kapogiannis BG, McClure C, Luo C (2014). Executive summary: opportunities for action and impact to address HIV and AIDS in adolescents. J Acquir Immune Defic Syndr.

[4] Baral S, Sifakis F, Cleghorn F, Beyrer C (2007). Elevated risk for HIV infection among men who have sex with men in low- and middle-income countries 2000–2006: a systematic review. PLoS Med.

[5] Herbst JH, Jacobs ED, Finlayson TJ, McKleroy VS, Neumann MS, Crepaz N (2008). Estimating HIV prevalence and risk behaviors of transgender persons in the United States: a systematic review. AIDS Behav.

[6] Baral S, Beyrer C, Muessig K, Poteat T, Wirtz AL, Decker MR (2012). Burden of HIV among female sex workers in low-income and middle-income countries: a systematic review and meta-analysis. Lancet Infect Dis.

[7] WHO (2014). Consolidated guidelines on HIV prevention, diagnosis, treatment and care for key populations.

[8] Beyrer C, Baral S, Kerrigan D, El-Bassel N, Bekker LG, Celentano DD (2011). Expanding the space: inclusion of most-at-risk populations in HIV prevention, treatment, and care services. J Acquir Immune Defic Syndr.

[9] Sawyer SM, Afifi RA, Bearinger LH, Blakemore SJ, Dick B, Ezeh AC (2012). Adolescence: a foundation for future health. Lancet.

[10] Patel V, Flisher AJ, Hetrick S, McGorry P (2007). Mental health of young people: a global public-health challenge. Lancet.

[11] Viner RM, Ozer EM, Denny S, Marmot M, Resnick M, Fatusi A (2012). Adolescence and the social determinants of health. Lancet.

[12] Gore FM, Bloem PJ, Patton GC, Ferguson J, Joseph V, Coffey C (2011). Global burden of disease in young people aged 10–24 years: a systematic analysis. Lancet.

[13] Schunter BT, Cheng WS, Kendall M, Marais H (2014). Lessons learned from a review of interventions for adolescent and young key populations in Asia Pacific and opportunities for programming. J Acquir Immune Defic Syndr.

[14] Newman PA, Chakrapani V, Cook C, Shunmugam M, Kakinami L (2008). Determinants of sexual risk behavior among men who have sex with men accessing public sex environments in Chennai, India. J LGBT Health Res.

[15] Czerwinski M, McNutt LA, DeHovitz JA, Zielinski A, Rosinska M (2013). Refining HIV risk: the modifying effects of youth, gender and education among people who inject drugs in Poland. PLoS One.

[16] Klein H (2012). A comparison of HIV risk practices among unprotected sex-seeking older and younger men who have sex with other men. Aging Male.

[17] Wall KM, Stephenson R, Sullivan PS (2013). Frequency of sexual activity with most recent male partner among young, Internet-using men who have sex with men in the United States. J Homosex.

[18] George A, Sabarwal S (2013). Sex trafficking, physical and sexual violence, and HIV risk among young female sex workers in Andhra Pradesh, India. Int J Gynaecol Obstet.

[19] Urada LA, Silverman JG, Cordisco Tsai L, Morisky DE (2014). Underage youth trading sex in the Philippines: trafficking and HIV risk. AIDS Care.

[20] Januraga PP, Mooney-Somers J, Ward PR (2014). Newcomers in a hazardous environment: a qualitative inquiry into sex worker vulnerability to HIV in Bali, Indonesia. BMC Public Health.

[21] Arrington-Sanders R, Leonard L, Brooks D, Celentano D, Ellen J (2013). Older partner selection in young African-American men who have sex with men. J Adolesc Health.

[22] Wilson EC, Garofalo R, Harris DR, Belzer M (2010). Sexual risk taking among transgender male-to-female youths with different partner types. Am J Public Health.

[23] Kapadia F, Latka MH, Hudson SM, Golub ET, Campbell JV, Bailey S (2007). Correlates of consistent condom use with main partners by partnership patterns among young adult male injection drug users from five US cities. Drug Alcohol Depend.

[24] Mustanski B, Newcomb ME, Clerkin EM (2011). Relationship characteristics and sexual risk-taking in young men who have sex with men. Health Psychol.

[25] Mullen L, Barry J (2001). An analysis of 15–19-year-old first attenders at the Dublin Needle Exchange, 1990–97. Addiction.

[26] Thurnheer MC, Weber R, Toutous-Trellu L, Cavassini M, Elzi L, Schmid P (2010). Occurrence, risk factors, diagnosis and treatment of syphilis in the prospective observational Swiss HIV Cohort Study. AIDS.

[27] Koedijk FD, van Benthem BH, Vrolings EM, Zuilhof W, van der Sande MA (2014). Increasing sexually transmitted infection rates in young men having sex with men in the Netherlands, 2006–2012. Emerg Themes Epidemiol.

[28] Wilkinson A, El-Hayek C, Fairley CK, Leslie D, Roth N, Tee BK (2012). Incidence and risk factors associated with chlamydia in men who have sex with men: a cohort analysis of Victorian Primary Care Network for Sentinel Surveillance data. Sex Transm Infect.

[29] Masese L, Baeten JM, Richardson BA, Deya R, Kabare E, Bukusi E (2013). Incidence and correlates of Chlamydia trachomatis infection in a high-risk cohort of Kenyan women. Sex Transm Dis.

[30] Ryder N, Jin F, McNulty AM, Grulich AE, Donovan B (2009). Increasing role of herpes simplex virus type 1 in first-episode anogenital herpes in heterosexual women and younger men who have sex with men, 1992–2006. Sex Transm Infect.

[31] Rieg G, Lewis RJ, Miller LG, Witt MD, Guerrero M, Daar ES (2008). Asymptomatic sexually transmitted infections in HIV-infected men who have sex with men: prevalence, incidence, predictors, and screening strategies. AIDS Patient Care STDS.

[32] Kuyper LM, Collins CL, Kerr T, Hogg RS, Li K, Tyndall MW (2005). The prevalence and incidence of sexually transmitted infections in a prospective cohort of injection drug users in Vancouver, British Columbia. Can J Infect Dis Med Microbiol.

[33] Nyitray AG, Carvalho da Silva RJ, Baggio ML, Lu B, Smith D, Abrahamsen M (2011). Age-specific prevalence of and risk factors for anal human papillomavirus (HPV) among men who have sex with women and men who have sex with men: the HPV in men (HIM) study. J Infect Dis.

[34] Ali H, Donovan B, Wand H, Read TR, Regan DG, Grulich AE (2013). Genital warts in young Australians five years into national human papillomavirus vaccination programme: national surveillance data. BMJ.

[35] Centers for Disease Control and Prevention (CDC) (2011). Hepatitis C virus infection among adolescents and young adults:Massachusetts, 2002–2009. MMWR Morb Mortal Wkly Rep.

[36] Bautista CT, Todd CS, Abed AM, Botros BA, Strathdee SA, Earhart KC (2010). Effects of duration of injection drug use and age at first injection on HCV among IDU in Kabul, Afghanistan. J Public Health (Oxf).

[37] Tracy D, Hahn JA, Fuller Lewis C, Evans J, Briceno A, Morris MD (2014). Higher risk of incident hepatitis C virus among young women who inject drugs compared with young men in association with sexual relationships: a prospective analysis from the UFO Study cohort. BMJ Open.

[38] Diamond C, Thiede H, Perdue T, Secura GM, Valleroy L, Mackellar D (2003). Viral hepatitis among young men who have sex with men: prevalence of infection, risk behaviors, and vaccination. Sex Transm Dis.

[39] Matthews JE, Stephenson R, Sullivan PS (2012). Factors associated with self-reported HBV vaccination among HIV-negative MSM participating in an online sexual health survey: a cross-sectional study. PLoS One.

[40] WHO (2012). Adolescent pregnancy. Fact Sheet 364.

[41] Duff P, Shoveller J, Zhang R, Alexson D, Montaner JS, Shannon K (2011). High lifetime pregnancy and low contraceptive usage among sex workers who use drugs- an unmet reproductive health need. BMC Pregnancy Childbirth.

[42] Yam EA, Mnisi Z, Maziya S, Kennedy C, Baral S (2014). Use of emergency contraceptive pills among female sex workers in Swaziland. J Fam Plann Reprod Health Care.

[43] Decker MR, Yam EA, Wirtz AL, Baral SD, Peryshkina A, Mogilnyi V (2013). Induced abortion, contraceptive use, and dual protection among female sex workers in Moscow, Russia. Int J Gynaecol Obstet.

[44] Zhang XD, Kennedy E, Temmerman M, Li Y, Zhang WH, Luchters S (2014). High rates of abortion and low levels of contraceptive use among adolescent female sex workers in Kunming, China: a cross-sectional analysis. Eur J Contracept Reprod Health Care.

[45] Lau JT, Mui LW, Tsui HY, Wong E, Ho SP (2007). Prevalence of induced abortion and associated factors among Chinese female sex workers in Hong Kong. J Sex Marital Ther.

[46] Thorne C, Semenenko I, Malyuta R (2012). Prevention of mother-to-child transmission of human immunodeficiency virus among pregnant women using injecting drugs in Ukraine, 2000–10. Addiction.

[47] Rotondi NK, Bauer GR, Scanlon K, Kaay M, Travers R, Travers A (2013). Nonprescribed hormone use and self-performed surgeries: “do-it-yourself” transitions in transgender communities in Ontario, Canada. Am J Public Health.

[48] Gooren LJ, Sungkaew T, Giltay EJ (2013). Exploration of functional health, mental well-being and cross-sex hormone use in a sample of Thai male-to-female transgendered persons (kathoeys). Asian J Androl.

[49] Guadamuz TE, Wimonsate W, Varangrat A, Phanuphak P, Jommaroeng R, McNicholl JM (2011). HIV prevalence, risk behavior, hormone use and surgical history among transgender persons in Thailand. AIDS Behav.

[50] Sevelius JM, Reznick OG, Hart SL, Schwarcz S (2009). Informing interventions: the importance of contextual factors in the prediction of sexual risk behaviors among transgender women. AIDS Educ Prev.

[51] Deering KN, Amin A, Shoveller J, Nesbitt A, Garcia-Moreno C, Duff P (2014). A systematic review of the correlates of violence against sex workers. Am J Public Health.

[52] Rushing R, Watts C, Rushing S (2005). Living the reality of forced sex work: perspectives from young migrant women sex workers in northern Vietnam. J Midwifery Womens Health.

[53] Aho J, Hakim A, Vuylsteke B, Semde G, Gbais HG, Diarrassouba M (2014). Exploring risk behaviors and vulnerability for HIV among men who have sex with men in Abidjan, Cote d'Ivoire: poor knowledge, homophobia and sexual violence. PLoS One.

[54] Guadamuz TE, Wimonsate W, Varangrat A, Phanuphak P, Jommaroeng R, Mock PA (2011). Correlates of forced sex among populations of men who have sex with men in Thailand. Arch Sex Behav.

[55] Decker MR, Miller E, McCauley HL, Tancredi DJ, Anderson H, Levenson RR (2014). Recent partner violence and sexual and drug-related STI/HIV risk among adolescent and young adult women attending family planning clinics. Sex Transm Infect.

[56] Koblin BA, Torian L, Xu G, Guilin V, Makki H, Mackellar D (2006). Violence and HIV-related risk among young men who have sex with men. AIDS Care.

[57] Deuba K, Ekstrom AM, Shrestha R, Ionita G, Bhatta L, Karki DK (2013). Psychosocial health problems associated with increased HIV risk behavior among men who have sex with men in Nepal: a cross-sectional survey. PLoS One.

[58] McDougal L, Strathdee SA, Rangel G, Martinez G, Vera A, Sirotin N (2013). Adverse pregnancy outcomes and sexual violence among female sex workers who inject drugs on the United States-Mexico border. Violence Vict.

[59] Decker MR, McCauley HL, Phuengsamran D, Janyam S, Silverman JG (2011). Sex trafficking, sexual risk, sexually transmitted infection and reproductive health among female sex workers in Thailand. J Epidemiol Community Health.

[60] Su S, Li X, Zhang L, Lin D, Zhang C, Zhou Y (2014). Age group differences in HIV risk and mental health problems among female sex workers in Southwest China. AIDS Care.

[61] Mustanski BS, Garofalo R, Emerson EM (2010). Mental health disorders, psychological distress, and suicidality in a diverse sample of lesbian, gay, bisexual, and transgender youths. Am J Public Health.

[62] Mackesy-Amiti ME, Donenberg GR, Ouellet LJ (2015). Prescription opioid misuse and mental health among young injection drug users. Am J Drug Alcohol Abuse.

[63] Yorick R, Skipalska H, Suvorova S, Sukovatova O, Zakharov K, Hodgdon S (2012). HIV prevention and rehabilitation models for women who inject drugs in Russia and Ukraine. Adv Prev Med.

[64] Amirkhanian YA, Kelly JA, McAuliffe TL (2003). Psychosocial needs, mental health, and HIV transmission risk behavior among people living with HIV/AIDS in St Petersburg, Russia. AIDS.

[65] Hong Y, Fang X, Li X, Liu Y, Li M, Tai-Seale T (2010). Self-perceived stigma, depressive symptoms, and suicidal behaviors among female sex workers in China. J Transcult Nurs.

[66] Mustanski B, Andrews R, Herrick A, Stall R, Schnarrs PW (2014). A syndemic of psychosocial health disparities and associations with risk for attempting suicide among young sexual minority men. Am J Public Health.

[67] Santos GM, Do T, Beck J, Makofane K, Arreola S, Pyun T (2014). Syndemic conditions associated with increased HIV risk in a global sample of men who have sex with men. Sex Transm Infect.

[68] Brennan J, Kuhns LM, Johnson AK, Belzer M, Wilson EC, Garofalo R (2012). Syndemic theory and HIV-related risk among young transgender women: the role of multiple, co-occurring health problems and social marginalization. Am J Public Health.

[69] Phillips G, Hightow-Weidman LB, Fields SD, Giordano TP, Outlaw AY, Halpern-Felsher B, 2nd (2014). Experiences of community and parental violence among HIV-positive young racial/ethnic minority men who have sex with men. AIDS Care.

[70] Sarin E, Samson L, Sweat M, Beyrer C (2011). Human rights abuses and suicidal ideation among male injecting drug users in Delhi, India. Int J Drug Policy.

[71] Grossman AH, D'Augelli AR (2006). Transgender youth: invisible and vulnerable. J Homosex.

[72] Rio Navarro J, Cohen J, Rocillo Arechaga E, Zuniga E (2012). Physical and sexual violence, mental health indicators, and treatment seeking among street-based population groups in Tegucigalpa, Honduras. Rev Panam Salud Publica.

[73] Mustanski BS, Newcomb ME, Du Bois SN, Garcia SC, Grov C (2011). HIV in young men who have sex with men: a review of epidemiology, risk and protective factors, and interventions. J Sex Res.

[74] Francis JM, Grosskurth H, Changalucha J, Kapiga SH, Weiss HA (2014). Systematic review and meta-analysis: prevalence of alcohol use among young people in eastern Africa. Trop Med Int Health.

[75] Spear LP (2002). Alcohol's effects on adolescents. Alcohol Res Health.

[76] Zhang XD, Temmerman M, Li Y, Luo W, Luchters S (2013). Vulnerabilities, health needs and predictors of high-risk sexual behaviour among female adolescent sex workers in Kunming, China. Sex Transm Infect.

[77] Debeck K, Kerr T, Marshall BD, Simo A, Montaner J, Wood E (2013). Risk factors for progression to regular injection drug use among street-involved youth in a Canadian setting. Drug Alcohol Depend.

[78] Kelly J, Davis C, Schlesinger C (2014). Substance use by same sex attracted young people: prevalence, perceptions and homophobia. Drug Alcohol Rev.

[79] Newcomb ME (2013). Moderating effect of age on the association between alcohol use and sexual risk in MSM: evidence for elevated risk among younger MSM. AIDS Behav.

[80] Le Marchand C, Evans J, Page K, Davidson PJ, Hahn JA (2013). Hazardous alcohol consumption among young adult IDU and its association with high risk behaviors. Drug Alcohol Depend.

[81] Vorobjov S, Des Jarlais DC, Abel-Ollo K, Talu A, Ruutel K, Uuskula A (2013). Socio-demographic factors, health risks and harms associated with early initiation of injection among people who inject drugs in Tallinn, Estonia: evidence from cross-sectional surveys. Int J Drug Policy.

[82] Fuller CM, Vlahov D, Latkin CA, Ompad DC, Celentano DD, Strathdee SA (2003). Social circumstances of initiation of injection drug use and early shooting gallery attendance: implications for HIV intervention among adolescent and young adult injection drug users. J Acquir Immune Defic Syndr.

[83] Kermode M, Longleng V, Singh BC, Bowen K, Rintoul A (2009). Killing time with enjoyment: a qualitative study of initiation into injecting drug use in north-east India. Subst Use Misuse.

[84] Hadland SE, Kerr T, Marshall BD, Small W, Lai C, Montaner JS (2010). Non-injection drug use patterns and history of injection among street youth. Eur Addict Res.

[85] Balakireva OM, Grund JPC, Barendregt C, Rubanets YV, Ryabova MV, Volyk AM (2006). Risk and protective factors in the initiation of injecting drug use: report of a respondent driven sampling study & strategy paper preventing the initiation of injecting drug use among vulnerable adolescents and young people: final report.

[86] Horyniak D, Dietze P, Degenhardt L, Higgs P, McIlwraith F, Alati R (2013). The relationship between age and risky injecting behaviours among a sample of Australian people who inject drugs. Drug Alcohol Depend.

[87] Degenhardt L, Kinner SA, Roxburgh A, Black E, Bruno R, Fetherston J (2008). Drug use and risk among regular injecting drug users in Australia: does age make a difference?. Drug Alcohol Rev.

[88] Buxton JA, Rothon D, Durigon M, Lem M, Tu AW, Remple VP (2009). Hepatitis C and HIV prevalence using oral mucosal transudate, and reported drug use and sexual behaviours of youth in custody in British Columbia. Can J Public Health.

[89] Li J, Liu H, Li J, Luo J, Jarlais DD, Koram N (2011). Role of sexual transmission of HIV among young noninjection and injection opiate users: a respondent-driven sampling study. Sex Transm Dis.

[90] Evans JL, Hahn JA, Lum PJ, Stein ES, Page K (2009). Predictors of injection drug use cessation and relapse in a prospective cohort of young injection drug users in San Francisco, CA (UFO Study). Drug Alcohol Depend.

[91] Zabransky T, Csemy L, Grohmannova K, Janikova B, Brenza J (2011). Mortality of cohort of very young injecting drug users in Prague, 1996–2010. Cent Eur J Public Health.

[92] Cheng T, Wood E, Feng C, Mathias S, Montaner J, Kerr T (2013). Transitions into and out of homelessness among street-involved youth in a Canadian setting. Health Place.

[93] Cluver L, Orkin M, Boyes M, Gardner F, Meinck F (2011). Transactional sex amongst AIDS-orphaned and AIDS-affected adolescents predicted by abuse and extreme poverty. J Acquir Immune Defic Syndr.

[94] Tarasuk V, Dachner N, Poland B, Gaetz S (2009). Food deprivation is integral to the ‘hand to mouth’ existence of homeless youths in Toronto. Public Health Nutr.

[95] Kermode M, Songput CH, Sono CZ, Jamir TN, Devine A (2012). Meeting the needs of women who use drugs and alcohol in North-east India – a challenge for HIV prevention services. BMC Public Health.

[96] Russell ST, Ryan C, Toomey RB, Diaz RM, Sanchez J (2011). Lesbian, gay, bisexual, and transgender adolescent school victimization: implications for young adult health and adjustment. J Sch Health.

[97] Reid JA, Piquero AR (2014). Age-graded risks for commercial sexual exploitation of male and female youth. J Interpers Violence.

[98] Koblin BA, Mayer KH, Eshleman SH, Wang L, Mannheimer S, del Rio C (2013). Correlates of HIV acquisition in a cohort of black men who have sex with men in the United States: HIV prevention trials network (HPTN) 061. PLoS One.

[99] Vu L, Andrinopoulos K, Tun W, Adebajo S (2013). High levels of unprotected anal intercourse and never testing for HIV among men who have sex with men in Nigeria: evidence from a cross-sectional survey for the need for innovative approaches to HIV prevention. Sex Transm Infect.

[100] Kiriazova TK, Postnov OV, Perehinets IB, Neduzhko OO (2013). Association of injecting drug use and late enrolment in HIV medical care in Odessa Region, Ukraine. HIV Med.

[101] Graham SM, Mugo P, Gichuru E, Thiong'o A, Macharia M, Okuku HS (2013). Adherence to antiretroviral therapy and clinical outcomes among young adults reporting high-risk sexual behavior, including men who have sex with men, in coastal Kenya. AIDS Behav.

[102] Hadland SE, Milloy MJ, Kerr T, Zhang R, Guillemi S, Hogg RS (2012). Young age predicts poor antiretroviral adherence and viral load suppression among injection drug users. AIDS Patient Care STDS.

[103] Mtetwa S, Busza J, Chidiya S, Mungofa S, Cowan F (2013). “You are wasting our drugs”: health service barriers to HIV treatment for sex workers in Zimbabwe. BMC Public Health.

[104] Salomon EA, Mimiaga MJ, Husnik MJ, Welles SL, Manseau MW, Montenegro AB (2009). Depressive symptoms, utilization of mental health care, substance use and sexual risk among young men who have sex with men in EXPLORE: implications for age-specific interventions. AIDS Behav.

[105] Fields EL, Bogart LM, Smith KC, Malebranche DJ, Ellen J, Schuster MA (2015). “I always felt I had to prove my manhood”: homosexuality, masculinity, gender role strain, and HIV risk among young black men who have sex with men. Am J Public Health.

[106] Busza J, Mtetwa S, Chirawu P, Cowan F (2014). Triple jeopardy: adolescent experiences of sex work and migration in Zimbabwe. Health Place.

[107] Miller CL, Fielden SJ, Tyndall MW, Zhang R, Gibson K, Shannon K (2011). Individual and structural vulnerability among female youth who exchange sex for survival. J Adolesc Health.

[108] Scorgie F, Nakato D, Harper E, Richter M, Maseko S, Nare P (2013). We are despised in the hospitals’: sex workers’ experiences of accessing health care in four African countries. Cult Health Sex.

[109] Islam M, Stern T, Conigrave KM, Wodak A (2008). Client satisfaction and risk behaviours of the users of syringe dispensing machines: a pilot study. Drug Alcohol Rev.

[110] Williams KA, Chapman MV (2011). Comparing health and mental health needs, service use, and barriers to services among sexual minority youths and their peers. Health Soc Work.

[111] Kitts RL (2010). Barriers to optimal care between physicians and lesbian, gay, bisexual, transgender, and questioning adolescent patients. J Homosex.

[112] Lee L, Rand CS, Ellen JM, Agwu AL (2014). Factors informing HIV providers’ decisions to start antiretroviral therapy for young people living with behaviorally acquired HIV. J Adolesc Health.

[113] Corliss HL, Belzer M, Forbes C, Wilson EC (2007). An evaluation of service utilization among male to female transgender youth: qualitative study of a clinic-based sample. J LGBT Health Res.

[114] Goldenberg SM, Chettiar J, Nguyen P, Dobrer S, Montaner J, Shannon K (2014). Complexities of short-term mobility for sex work and migration among sex workers: violence and sexual risks, barriers to care, and enhanced social and economic opportunities. J Urban Health.

[115] Omura JD, Wood E, Nguyen P, Kerr T, DeBeck K (2014). Incarceration among street-involved youth in a Canadian study: implications for health and policy interventions. Int J Drug Policy.

[116] Thomson N, Sutcliffe CG, Sirirojn B, Keawvichit R, Wongworapat K, Sintupat K (2009). Correlates of incarceration among young methamphetamine users in Chiang Mai, Thailand. Am J Public Health.

[117] Busza JR, Balakireva OM, Teltschik A, Bondar TV, Sereda YV, Meynell C (2011). Street-based adolescents at high risk of HIV in Ukraine. J Epidemiol Community Health.

[118] Hayashi K, Ti L, Csete J, Kaplan K, Suwannawong P, Wood E (2013). Reports of police beating and associated harms among people who inject drugs in Bangkok, Thailand: a serial cross-sectional study. BMC Public Health.

[119] Scorgie F, Vasey K, Harper E, Richter M, Nare P, Maseko S (2013). Human rights abuses and collective resilience among sex workers in four African countries: a qualitative study. Global Health.

[120] Wirtz AL, Jumbe V, Trapence G, Kamba D, Umar E, Ketende S (2013). HIV among men who have sex with men in Malawi: elucidating HIV prevalence and correlates of infection to inform HIV prevention. J Int AIDS Soc.

[121] Shannon K, Rusch M, Shoveller J, Alexson D, Gibson K, Tyndall MW (2008). Mapping violence and policing as an environmental-structural barrier to health service and syringe availability among substance-using women in street-level sex work. Int J Drug Policy.

[122] Ti L, Wood E, Shannon K, Feng C, Kerr T (2013). Police confrontations among street-involved youth in a Canadian setting. Int J Drug Policy.

[123] Decker MR, Pearson E, Illangasekare SL, Clark E, Sherman SG (2013). Violence against women in sex work and HIV risk implications differ qualitatively by perpetrator. BMC Public Health.

[124] Merkinaite S, Grund JP, Frimpong A (2010). Young people and drugs: next generation of harm reduction. Int J Drug Policy.

[125] Millett GA, Peterson JL, Flores SA, Hart TA, Jeffries WL, Wilson PA (2012). Comparisons of disparities and risks of HIV infection in black and other men who have sex with men in Canada, UK, and USA: a meta-analysis. Lancet.

[126] Mavedzenge SN, Luecke E, Ross DA (2014). Effective approaches for programming to reduce adolescent vulnerability to HIV infection, HIV risk, and HIV-related morbidity and mortality: a systematic review of systematic reviews. J Acquir Immune Defic Syndr.

[127] Dhana A, Luchters S, Moore L, Lafort Y, Roy A, Scorgie F (2014). Systematic review of facility-based sexual and reproductive health services for female sex workers in Africa. Global Health.

[128] Denno DM, Hoopes AJ, Chandra-Mouli V (2015). Effective strategies to provide adolescent sexual and reproductive health services and to increase demand and community support. J Adolesc Health.

[129] Heise LL (2011). What works to prevent partner violence? An evidence overview.

[130] Pulerwitz J, Oanh KT, Akinwolemiwa D, Ashburn K, Nyblade L (2014). Improving hospital-based quality of care by reducing HIV-related stigma: evaluation results from vietnam. AIDS Behav.

[131] Nyblade L, Stangl A, Weiss E, Ashburn K (2009). Combating HIV stigma in health care settings: what works?. J Int AIDS Soc.

[132] Apinundecha C, Laohasiriwong W, Cameron MP, Lim S (2007). A community participation intervention to reduce HIV/AIDS stigma, Nakhon Ratchasima province, northeast Thailand. AIDS Care.

[133] Nyblade L, Hong K, Anh N, Ogden J, Jain A, Stangl A (2008). Communities confront HIV stigma in Viet Nam: participatory interventions reduce HIV-related stigma in two provinces.

[134] Stangl A, Lloyd J, Brady L, Holland C, Baral S (2013). A systematic review of interventions to reduce HIV-related stigma and discrimination from 2002 to 2013: how far have we come?. J Int AIDS Soc.

[135] van der Elst EM, Smith AD, Gichuru E, Wahome E, Musyoki H, Muraguri N (2013). Men who have sex with men sensitivity training reduces homoprejudice and increases knowledge among Kenyan healthcare providers in coastal Kenya. J Int AIDS Soc.

[136] Govindasamy D, Meghij J, Kebede Negussi E, Clare Baggaley R, Ford N, Kranzer K (2014). Interventions to improve or facilitate linkage to or retention in pre-ART (HIV) care and initiation of ART in low- and middle-income settings – a systematic review. J Int AIDS Soc.

[137] Kerrigan DL, Fonner VA, Stromdahl S, Kennedy CE (2013). Community empowerment among female sex workers is an effective HIV prevention intervention: a systematic review of the peer-reviewed evidence from low- and middle-income countries. AIDS Behav.

[138] Wirtz AL, Pretorius C, Beyrer C, Baral S, Decker MR, Sherman SG (2014). Epidemic impacts of a community empowerment intervention for HIV prevention among female sex workers in generalized and concentrated epidemics. PLoS One.

[139] Ellsberg M, Arango DJ, Morton M, Gennari F, Kiplesund S, Contreras M (2014). Prevention of violence against women and girls: what does the evidence say?. Lancet.

[140] Moore L, Chersich MF, Steen R, Reza-Paul S, Dhana A, Vuylsteke B (2014). Community empowerment and involvement of female sex workers in targeted sexual and reproductive health interventions in Africa: a systematic review. Global Health.

[141] Swendeman D, Basu I, Das S, Jana S, Rotheram-Borus MJ (2009). Empowering sex workers in India to reduce vulnerability to HIV and sexually transmitted diseases. Soc Sci Med.

[142] Busza J, Douthwaite M, Bani R, Scutelniciuc O, Preda M, Simic D (2013). Injecting behaviour and service use among young injectors in Albania, Moldova, Romania and Serbia. Int J Drug Policy.

[143] Zaller N, Jeronimo A, Bratberg J, Case P, Rich JD (2010). Pharmacist and pharmacy staff experiences with non-prescription (NP) sale of syringes and attitudes toward providing HIV prevention services for injection drug users (IDUs) in providence, RI. J Urban Health.

[144] Grov C, Breslow AS, Newcomb ME, Rosenberger JG, Bauermeister JA (2014). Gay and bisexual men's use of the internet: research from the 1990s through 2013. J Sex Res.

[145] LeGrand S, Muessig KE, Pike EC, Baltierra N, Hightow-Weidman LB (2014). If you build it will they come? Addressing social isolation within a technology-based HIV intervention for young black men who have sex with men. AIDS Care.

[146] Holloway IW, Rice E, Gibbs J, Winetrobe H, Dunlap S, Rhoades H (2014). Acceptability of smartphone application-based HIV prevention among young men who have sex with men. AIDS Behav.

[147] Muessig KE, Pike EC, Fowler B, LeGrand S, Parsons JT, Bull SS (2013). Putting prevention in their pockets: developing mobile phone-based HIV interventions for black men who have sex with men. AIDS Patient Care STDS.

[148] Shoveller J, Knight R, Davis W, Gilbert M, Ogilvie G (2012). Online sexual health services: examining youth's perspectives. Can J Public Health.

[149] Blas MM, Menacho LA, Alva IE, Cabello R, Orellana ER (2013). Motivating men who have sex with men to get tested for HIV through the internet and mobile phones: a qualitative study. PLoS One.

[150] Justumus P, Colby D, Mai Doan Anh T, Balestre E, Becquet R, Orne-Gliemann J (2013). Willingness to use the internet to seek information on HIV prevention and care among men who have sex with men in Ho Chi Minh City, Vietnam. PLoS One.

[151] Kasatpibal N, Viseskul N, Srikantha W, Fongkaew W, Surapagdee N, Grimes RM (2012). Developing a web site for human immunodeficiency virus prevention in a middle income country: a pilot study from Thailand. Cyberpsychol Behav Soc Netw.

[152] Ybarra ML, Bull SS, Prescott TL, Birungi R (2014). Acceptability and feasibility of CyberSenga: an internet-based HIV-prevention program for adolescents in Mbarara, Uganda. AIDS Care.

[153] Yonker LM, Zan S, Scirica C, Jethwani K, Kinane TB (2015). “Friending” teens: systematic review of social media in adolescent and young adult health care. J Med Internet Res.

[154] Guse K, Levine D, Martins S, Lira A, Gaarde J, Westmorland W (2012). Interventions using new digital media to improve adolescent sexual health: a systematic review. J Adolesc Health.

[155] Young SD, Holloway I, Jaganath D, Rice E, Westmoreland D, Coates T (2014). Project HOPE: online social network changes in an HIV prevention randomized controlled trial for African American and Latino men who have sex with men. Am J Public Health.

[156] Allison S, Bauermeister JA, Bull S, Lightfoot M, Mustanski B, Shegog R (2012). The intersection of youth, technology, and new media with sexual health: moving the research agenda forward. J Adolesc Health.

[157] Stover J, Rosen J, Kasedde S, Idele P, McClure C (2014). The impact and cost of the HIV/AIDS investment framework for adolescents. J Acquir Immune Defic Syndr.

# Sub article: Review: An urgent need for research on factors impacting adherence to and retention in care among HIV-positive youth and adolescents from key populations

**DOI:** 10.7448/IAS.18.2.19393

**Published:** 2015-02-26

**Authors:** Priya Lall, Sin How Lim, Norliana Khairuddin, Adeeba Kamarulzaman

**Affiliations:** Centre of Excellence for Research in AIDS (CERiA), Department of Medicine, Faculty of Medicine, University of Malaya, Kuala Lumpur, Malaysia

**Keywords:** human immunodeficiency virus, young key populations, adherence, retention in HIV care, antiretroviral therapy

## Abstract

**Introduction:**

The 50% increase in HIV-related deaths in youth and adolescents (aged 10–24) from 2005 to 2012 highlights the need to improve HIV treatment and care in this population, including treatment adherence and retention. Youth and adolescents from key populations or young key populations (YKP) in particular are highly stigmatized and may face additional barrier(s) in adhering to HIV treatment and services. We reviewed the current knowledge on treatment adherence and retention in HIV care among YKP to identify gaps in the literature and suggest future directions to improve HIV care for YKP.

**Methods:**

We conducted a comprehensive literature search for YKP and their adherence to antiretroviral therapy (ART) and retention in HIV care on PsycInfo (Ovid), PubMed and Google Scholar using combinations of the keywords HIV/AIDS, ART, adolescents, young adults, adherence (or compliance), retention, men who have sex with men, transgender, injection drug users, people who inject drugs and prisoners. We included empirical studies on key populations defined by WHO; included the terms youth and adolescents and/or aged between 10 and 24; examined adherence to or retention in HIV care; and published in English-language journals. All articles were coded using NVivo.

**Results and discussion:**

The systematic search yielded 10 articles on YKP and 16 articles on behaviourally infected youth and adolescents from 1999 to 2014. We found no studies reporting on youth and adolescents identified as sex workers, transgender people and prisoners. From existing literature, adherence to ART was reported to be influenced by age, access to healthcare, the burden of multiple vulnerabilities, policy involving risk behaviours and mental health. A combination of two or more of these factors negatively impacted adherence to ART among YKP. Collectively, these studies demonstrated that future programmes need to be tailored specifically to YKP to ensure adherence.

**Conclusions:**

There is an urgent need for more systematic research in YKP. Current limited evidence suggests that healthcare delivery should be tailored to the unique needs of YKP. Thus, research on YKP could be used to inform future interventions to improve access to treatment and management of co-morbidities related to HIV, to ease the transition from paediatric to adult care and to increase uptake of secondary prevention methods.

## Introduction

According to WHO, youth and adolescents have become increasingly vulnerable to HIV infection [1]. In 2012, it was estimated that of the 35.3 million people living with HIV (PLHIV) globally, 5 million were aged 10–24. In the same year, over a third of new HIV-positive cases occurred among these age groups. During 2005–2012, while the global number of HIV-related deaths fell by 30%, the corresponding number among youth and adolescents increased by 50% [1]. The factors contributing to the higher mortality among HIV-positive youth and adolescents were the lack of awareness of sero-status [2–4], poor linkages between testing and treatment services [5], difficulty in retention in care [6] and lack of adherence to antiretroviral therapy (ART) regimes [7,8].

Youth and adolescents living with HIV can be broadly categorized into two groups according to routes of transmission: (1) infection at birth, that is, perinatally infected youth and adolescents (PIY), and (2) acquired by high-risk behaviours through injecting drug use and/or having condomless sex, that is, behaviourally infected youth and adolescents (BIY). It is important to note that adolescence can act as a transitional phase towards adulthood in which drug use and sexual experimentation are initiated, thus increasing the risk of contracting HIV [9,10]. For example, a national survey of high school students in the United States highlighted that 40.9% of adolescents had not used a condom in their last sexual encounter [11].

The WHO guidelines on testing and treatment of youth and adolescents aged 10–24 has identified men who have sex with men (MSM), transgender persons, people who inject drugs (PWID), sex workers and prisoners as young key populations (YKP). The term YKP recognizes that people belonging to these groups are at heightened risk of contracting HIV due to specific behaviours and social and legal environments which curtail their ability to protect themselves [7,12].

Currently little is known about how YKP engage with healthcare services while managing dual stigmas related to their HIV status as well as belonging to a marginalized section of the population. In order to understand these issues, it is useful to learn from the broader literature on youth and adolescents living with HIV in general. Research on PIY found a unique set of individual and environmental-level barriers to adherence and retention in HIV-related healthcare services. On the individual level, psychosocial barriers, such as depression and anxiety, have consistently been found to have an adverse effect on adherence of PIY to ART [13–15]. A cohort study of perinatally infected children and adolescents on ART in the United States found that depression or anxiety was predictive of non-adherence [16]. Meanwhile, on the environmental level, stigma [14], social or familial support [17] and socioeconomic status [15] can impact usage of healthcare services by youth and adolescents. People belonging to YKP can be stigmatized for their engagement in risk behaviours. Some HIV-positive YKP may, as a consequence, experience heightened socioeconomic and cultural barriers to accessing services owing to the fact that their already stigmatized status leaves them with few social or financial resources [9].

Previous reviews on adherence and retention in care in youth and adolescents have mostly focused on those who contracted HIV perinatally [13,18]. Although these reviews provide useful guidance on what types of individual and environmental-level barriers may affect HIV-positive YKP, individuals belonging to this group may have a slightly different set of needs to those who contracted it perinatally. For example, YKP who contracted HIV through injecting drug use could be in need of methadone treatment in addition to ART [19]. This literature review thus identified previous research on adherence to ART and retention in HIV-related care in YKP, discussed the current knowledge of individual and environmental-level barriers and facilitators to usage of healthcare services among these individuals, and suggested future directions in research to fill the gaps of knowledge and services in order to improve adherence to and retention in HIV care in these vulnerable populations.

## Methods

A comprehensive search for adherence to ART and retention in HIV care in YKP was conducted on PsycInfo (Ovid), PubMed and Google Scholar using combinations of the keywords HIV/AIDS, antiretroviral therapy, adolescents, young adults, adherence (or compliance), retention, MSM, transgender, injection drug users, PWID and prisoners. In addition, bibliographies of relevant articles were reviewed for supplementary studies. The inclusion criteria of the current review are empirical studies that (1) included key populations defined by WHO; (2) included the terms youth and adolescents or aged between 10 and 24; (3) examined adherence to or retention in HIV care; and (4) published in English-language journals. There were studies on adherence to and retention in HIV care among participants with wide age range but data were not disaggregated by age and therefore findings could not be inferred on the younger age group (10–24 years). These studies were excluded from the review. All articles were coded using NVivo. The systematic search yielded 26 articles dating from 1999 to 2014.

## Results and discussions

Our literature search yielded 26 studies overall, 20 of which were conducted in the United States (Table 1). Sixteen of these studies examined the adherence behaviours of BIY, where HIV was acquired through sexual risk behaviour or injecting drug use [20–31,33,34,39,42,43]. Seven other studies focused specifically on the treatment needs of HIV-positive young MSM (YMSM) belonging to ethnic minorities [32,36–38,40,41,44]. Finally, only two studies specifically assessed the adherence of young HIV-positive PWID [19,35]. Belzer *et al*. [42] focused on both BIY in general and YMSM.

**Table 1:** Studies conducted between the years 1999 and 2014 on adherence to ART and retention in care among BIY and YKP

Publication (first author, year)	Location (cities, country)	Study populations	Age (mean, range)	Sample size (HIV-positive)	Measurement of adherence to and/or retention in HIV care	Method	Intervention	Main findings
Belzer *et al*. 1999 [20]	Los Angeles, USA	BIY	15–24	31	Self-reported adherence	Quantitative (survey)	No	Medication adherence most significantly correlates with stability of living conditions in BIY.
Martinez *et al*. 2000 [21]	Cook county, Illinois, USA	BIY	13–21	25	Self-reported adherence	Quantitative (retrospective analysis of medical charts)	No	61% of subjects reported >90% compliance with their medications in the previous 90 days. 5 of 10 substance abusing subjects reported adherence to ART.
Murphy *et al*. 2001 [22]	13 cities in USA	BIY	12–19	161	Self-reported adherence, psychological theory to measure anxiety, social support and depression. Viral load (VL).	Quantitative (cohort study)	No	Higher levels of adherence associated with decreased depression, a strong association between adherence and reduced VL.
Rogers *et al*. 2001 [23]	USA	BIY	N/A^a^	288	Viral load and CD4 count	Quantitative (evaluation)	Yes	Only 18 of 288 participants received full TREAT programme, which led to adherence with ART.
Murphy *et al*. 2003 [24]	13 cities in USA	BIY	12–19	114	Self-reported adherence and viral load (HIV-1 RNA level in plasma)	Quantitative (survey)	No	Viral load was significantly associated with self-report of adherence to ART. Only 28.3% of adolescents reported taking all of their prescribed antiretroviral medications in the previous month.
Flynn *et al*. 2004 [25]	28 sites in the US and Puerto Rico in USA	BIY	8–22	120	Self-reported adherence and viral load	Quantitative (cohort study)	No	Adherence to ART was the only predictor of achieving undetectable virus loads.
Murphy *et al*. 2005 [26]	13 cities in USA	BIY	18.4, 12–18	231	Self-reported adherence, behavioural factors associated with adherence and viral load	Quantitative (survey)	No	Adolescents in the later HIV disease stage were less likely to be adherent compared with those in the earlier disease stage. Less alcohol use and being in school were associated with adherence by adolescents on weekends and over the preceding month.
Puccio *et al*. 2006 [27]	Los Angeles, USA	BIY	16–24	81	Self-reported adherence	Quantitative (pilot intervention study)	Yes	Most participants found the calls to be helpful and the level of intrusion into their daily lives acceptable. Using cell phone reminders to assist patients does not require an extensive amount of daily staff time.
Naar-King *et al*. 2006 [28]	USA	BIY	16–24	24	Self-reported adherence, self-efficacy, social support, psychological distress	Quantitative (survey)	No	Self-efficacy and psychological distress were significantly correlated with adherence but social support was not. Social support specific to taking medications was correlated with self-efficacy.
Rao *et al*. 2007 [29]	Chicago, USA	BIY	17–25	25	Self-reported adherence	Qualitative	No	Half of respondents indicated that they skipped doses because they feared family or friends would discover their status, suggesting that HIV stigma impacts treatment for youth on several levels, from the accuracy of communication with medical providers to medication adherence, subsequent health outcomes and the emergence of treatment-resistant strains.
Rudy *et al*. 2009 [30]	USA	BIY and blood products. Separate sexual abuse category	12–24	396	Survey instrument to measure adherence and outcome expectancy of adherence	Quantitative (observational study)	No	Non-adherence influenced by not having healthcare insurance, dropped out of school, homelessness and/or spent time in detention facility.
Garvie *et al*. 2010 [31]	Mid-southern USA	BIY, blood transfusion and unknown	16–24	60	Routine pharmacy pill count and self-reported. CD4 and VL.	Quantitative (survey)	No	The first study to measure adherence measurement based on both CD4 and VL. Non-adherence was related to off-schedule dosing.
Magnus *et al*. 2010 [32]	Bronx, Chapel Hill, Chicago, Detroit, Houston, Los Angeles, Oakland, Rochester, USA	AA, Latino YMSM	16–24	224	Retention defined as programme visits every three months	Quantitative (cohort study)	No	Retention associated with <21 years old, history of depression, receipt of programme services, feeling respected at clinic.
Comulada *et al*. 2003 [33]	Los Angeles, USA	BIY	14–29	253	Self-reported adherence, health status, sexual behaviour, substance use and psychological measures	Quantitative (survey)	No	Almost all youth had been offered ART (84%); 77% had ever used it, 54% were currently using and 63% of users adhered to 90% of their medications. Compared to non-users, users were more likely to be female, Latino or AA.
Agwu *et al*. 2011 [34]	17 US Clinic sites	BIY	18–24	3127	Self-reported adherence and clinic visits	Quantitative (retrospective study)	No	Youth PLHIV less likely to report injecting drug use behaviour. They were less likely to initiate ART.
Tapp *et al*. 2011^b^ [19]	Vancouver, Canada	YPWID	<24	PWID <24 (n=24), N=545	Adherence measured by compliance to prescription refill	Quantitative (cohort study)	No	Younger age (<24), being female, daily heroin injection and daily cocaine injection were negatively associated with 95% adherence while methadone treatment was positively associated with adherence.
Hadland *et al*. 2012^b^ [35]	Vancouver, Canada	YPWID	Median=37.2, age was dichotomized at 29	545	Self-reported adherence, VL	Quantitative (cohort study)	No	Young adults (age <29) were less adherent and were less likely to achieve VL suppression.
Wohl *et al*. 2011 [36]	Los Angeles, USA	AA and Latino YMSM	18–24	61	Retention associated with number of intervention visits, prescription of ART	Quantitative (pilot intervention study)	Yes	Highlights the critical needs of HIV-positive AA and Latino YMSM and demonstrate that a clinic-based YCM can be effective in stabilizing hard-to-reach clients and retaining them in consistent HIV care.
Hightow-Weidman *et al*. 2011 [37]	North Carolina, USA	AA and Latino MSM	Mean age 21	81	Retention defined as 1 medical visit every four months	Quantitative (cohort study)	Yes	Interventions on adherence need to actively reach out to youth populations.
Bouris *et al*. 2013 [38]	Chicago, USA	AA YMSM and TG	16–29	94	Self-reported adherence, VL	Quantitative (RCT)	Yes	Supportive relationships promote retention in care.
Barnes *et al*. 2013 [39]	Baltimore, New York City, Washington, USA	BIY, PIY	13–21	166	Assessed HIV knowledge	Quantitative (survey)	Yes	BIY outperformed PIY on questions related to disease awareness.
Gillman *et al*. 2013 [40]	Houston, USA	AA YMSM	Mean 19.9	47	Retention in care defined as completion of physician visits 90 days after linkage to care	Quantitative (survey)	No	Greater conspiracy beliefs were associated with negative medication attitudes while trust in physicians was correlated with positive medication attitudes; conspiracy beliefs were not associated with poor linkage to care and retention.
Harper *et al*. 2013 [41]	14 cities in USA	YMSM (66% AA, 19% Latino)	Mean 21.5, range 16–24	200	Self-reported adherence to medical appointment in the past three months	Quantitative (survey)	No	Ethnic identity affirmation and HIV-positive identity were associated with significantly higher risk for missed appointments in the past three months.
Belzer *et al*. 2013 [42]	Los Angeles, Washington, New Orleans, Fort Lauderdale, San Francisco, USA	BIY, YMSM	15–24	37	Self-reported adherence (dichotomized at 90%), viral load data abstracted from medical record	Quantitative	Yes	Intervention of daily cell phone conversation with health care providers. Self-reported adherence was significantly higher in intervention group than in the control group.
Saberi *et al*. 2014 [43]	USA	BIY, PIY	12–24	1317	Self-reported adherence in the past seven days (dichotomized at 100%); plasma HIV RNA	Quantitative	No	Pillbox was the most endorsed adherence device. Using adherence devices was inversely associated with having undetectable viral load. BIY more likely to be gay, adherent to ART and never been to jail.
Hussen *et al*. 2014 [44]	Atlanta, USA	YMSM	13–24	20	Self-reported adherence	Qualitative	No	Successful transition to adulthood and optimal ART adherence were inextricably linked. Detrimental impact of HIV on development was moderated by the degree of physical illness at diagnosis.

a Only specify participants as from REACH project

b these two studies were conducted on the same cohort.

AA=African American; ART=antiretroviral therapy; BIY=behaviourally infected youth and adolescents including sexual behaviour and injecting drug use; HAART=highly active antiretroviral therapy; HIV=human immunodeficiency virus; PIY=perinatally infected youth and adolescents; RCT=randomized control trial; REACH=Reaching for Excellence in Adolescents Care and Health; TG=transgender; TREAT=Therapeutic Regimens Enhancing Adherence in Teens; YCM=youth-focused case management; YMSM=young men having sex with men; YPWID=young people who inject drugs.

Research that specifically focused on adherence to ART regimes in HIV-positive youth and adolescents was relatively sparse because many clinical studies on treatment classified children and young adults into the age groups of around 0–14 and 15–24 [45,46], which overlaps the WHO definition of youth and adolescent of 10–24 years. These studies may fail to uncover factors affecting adherence which would be unique to HIV-positive adolescents (age 10–18) and young adults (age 19–24), simply due to different age categorization. There was even more of a dearth of literature on YKP, partly because it is more challenging to recruit HIV-positive young sexual minorities, sex workers, PWID and prisoners into research studies.

The literature search, thus, revealed that current knowledge on adherence to ART and retention in care in YKP was limited as research was heavily concentrated in the United States and focused on key populations which are of concern to that particular setting, including YMSM of ethnic minorities. To the best of our knowledge, there were no peer-reviewed articles that focused specifically on the treatment needs of young female sex workers, transgender youth and adolescents, and young offenders. There were, consequently, glaring gaps in the literature, as there appeared to be little to no research on adherence in YKP in developing countries, where most PLHIV live [46]. Furthermore, we were unable to find any studies that explored possible gender determinants of ART adherence, although 16 studies from the 26 studies reviewed here did involve female subjects.

The following section categorizes studies according to BIY in general and the different categories of YKP as per WHO classifications. We focus on individual and environmental factors related to adherence to ART and retention in care which were deemed as unique to each group. The final section of the results explores studies of interventions addressing the treatment needs of YKP.

### Research on BIY

Research on ART adherence among BIY did not focus on one particular key population, which resulted in some studies not differentiating participants according to their route of transmission and risk groups. Nonetheless, these studies do provide a useful overview of types of factors which may affect retention in care in YKP and how their adherence behaviours may differ from those of PIY.

Nine studies on BIY which included young people who inject drugs (YPWID) recruited participants from the Reaching for Excellence in Adolescent Care and Health (REACH) project [22–26,30,34,42,43]. REACH was originally designed as an observational study of HIV-positive patients attending “adolescent-specific medical care centres” in the United States [22]. Participants between the ages of 12 and 18 who contracted HIV through high-risk behaviours, such as condomless sexual contact or injecting drug use, were intentionally sampled in order to compare their behaviours to that of HIV-negative adolescents who engaged in similar behaviours and PIY. These studies found that risky sexual behaviours or injecting drug use, self-efficacy and positive mental health outcomes were associated with greater ART adherence.

Studies sampling from REACH suggested that adherence to ART in YKP were affected by a combination of individual and environmental-level barriers. At the individual level, younger age and history of depression were significantly associated with failure to adhere to complex medical regimes [26]. At the environmental level, unstable housing conditions [30] and lack of attendance at school acted as barriers to adherence [34]. Although there was no association between the ownership of medical insurance and initiation of ART, it was found that the type of medical insurance could influence their usage of healthcare services [34]. Those who were in receipt of publicly funded insurance were more likely to discontinue their treatment than those who had no insurance or private medical coverage. There was a possibility that “the small prescriptions co-payment that may be associated with publicly funded insurance programmes … may potentially serve as an impediment to … (art) continuation”, suggesting that participants with few financial resources may face many more difficulties in adhering to treatment [34], p. 6].

These studies also indicated that YKP who experienced a combination of both individual and environmental level barriers simultaneously may experience heightened difficulties in adhering to treatment. Rudy *et al*. [30] statistically tested the impact that the amount and type of barriers including not having healthcare insurance, having dropped out of school, homelessness and/or having spent time in detention facilities), as well as the existence of a mental disorder, had on adherence. It was found that 73% of participants with no barriers were adherent in comparison to 62% of those experiencing one barrier and 40% of those who reported two or more barriers. Moreover, 69% of respondents who had a low level of self-efficacy, a mental disorder and experienced at least one structural barrier were non-adherent.

Some HIV-positive youth and adolescents may attempt to mitigate the impact of individual and environmental level barriers on to adherence through actions which are not always beneficial towards their overall treatment. A focus group study of HIV-positive BIY on how they managed their HIV diagnosis found that many participants would conceal their sero-status from family and friends in order to avoid being stigmatized [29]. This resulted in half of the respondents skipping doses due to the fear that others would learn of their condition.

A few studies did, however, demonstrate that BIY were able to overcome environmental and individual level barriers in order to be more adherent to ART than their perinatally infected counterparts [31]. In a multi-clinic survey of HIV-positive youth and adolescents who were prescribed ART, Saberi *et al*. [43] found that full adherence was correlated with the behavioural route of infection, MSM behaviour, never being jailed and not using alcohol or illegal drugs]. It is possible that BIY may have more knowledge and awareness of their condition [39] than PIY due to the fact that they are expected to take charge of their treatment in the absence of family and social networks [17].

Although research on BIY provided a useful overview on individual and environmental level barriers which could potentially impact adherence to ART and retention in healthcare in YKP, very few studies distinguished between behavioural routes of transmission among their participants [26,31,33,34]. This resulted in different groups of YKP with varying treatment needs, such as YPWID and YMSM, being included into one category.

The other methodological issue was that many studies tested respondents’ socio-demographic characteristics against outcome variables without having *a priori* theory on how these factors may impact respondent's adherence to treatment [21,27,31,34]. Moreover, these studies often failed to include measurements of factors which could impact adherence and retention in care for youth and adolescents in general, let alone those belonging to key populations, such as their transition from child to adult healthcare services [44].

### Research on YMSM

Our literature search revealed only seven peer-reviewed publications yielded from research studies specifically on YMSM, most of which were conducted in the United States [32,36–38,40,41,44]. Six of these studies employ quantitative methods [36–38,40,41,44] , such as randomized control trials and surveys, to examine factors which could impact adherence to treatment regimes in YMSM, including age, ethnicity and sexual identity, while one study uses qualitative research methods [44].

In contrast to studies on treatment behaviours of BIY, research on African American YMSM was strongly informed by theories that took into account factors which were particular to their age, ethnicity and sexual identity. As a consequence, these studies were able to explore at length factors which could be unique to this particular YKP. One study examined respondents’ “conspiracy beliefs” in relation to HIV, namely the beliefs that the government was involved in the spread of HIV, had highlighted that many African Americans held these attitudes [40]. It was found that participants with greater “conspiracy beliefs” also had negative attitudes towards medication. None of these conspiracy beliefs, however, were correlated with CD4 counts at diagnosis, nor linkage and retention in care in the study, which suggested that participants were still willing to use treatment even if they did not fully trust their doctors.

As these studies incorporated theories and measurements which were relevant to YMSM, they were able to demonstrate that this population suffered from intersecting vulnerabilities due to increased stigma from belonging to three marginalized populations: ethnic minority, MSM, and HIV-positive. In a survey on HIV-positive YMSM of African American and Latino origins from Chicago, participants who held negative attitudes towards being gay and HIV-positive and strongly identified as belonging to an ethnic minority were more likely to miss clinical appointments [41]. These findings demonstrated the importance of understanding the development of multiple identities when treating YMSM from ethnic minorities.

In addition, YMSM belonging to ethnic minorities may experience environmental level barriers to adherence, such as poverty. For instance, in an assessment of a youth-focused case management intervention targeting HIV-positive YMSM from ethnic minorities, over three quarters of participants were in urgent neeed of stable housing, nutritional support, drug rehabilitation and mental health services at baseline [36]. These barriers to adherence were mitigated through increased number of intervention visits, more hours in the intervention and prescription of ART.

Finally, these studies were also able to identitify possible facilitators to adherence to ART and retention in care. A qualitative study of experiences of living with HIV and adherence to ART among African American YMSM found that their treatment behaviours were influenced by the developmental goals that they created as part of transitioning towards adulthood [44]. Participants who were able to attain self sufficiency through the development of a positive gay and HIV-positive identity were better able to adhere to medication than those who viewed their condition and sexual orientation in negative terms. These findings indicated that future interventions may need to tailor care of HIV-positive YMSM to engage with their developmental needs as well as their negotiation of multiple identities.

### Research on YPWID

The literature search identified only two studies, both came from a cohort study that investigated adherence behaviours of YPWID in Canada between 1996 and 2008 [19,35]. These studies did not focus on the youth and adolescent population as defined by the WHO; however, they did investigate factors which could impact ART adherence and retention in care in this particular group. One of these studies used age categorization of young adult (18–29 years) which did not fit into the WHO definition (18–24 years) [35]. Furthermore, the study did not disaggregate the data by age group, thus the results could not be inferred to the 18–24 age group. In the other study, participants who were younger than 25 years, female, and not receiving methadone treatment displayed a lower likelihood of being adherent to 95% of medication [19]. These findings suggest that female YPWID may experience greater barriers to adherence than their male counterparts, suggesting a gender bias. Further, female adult PWID were revealed to be harder to identify and procure healthcare services as they secure drugs from their abusive male partners [47].

### Research on interventions targeted towards YKP

From the 26 articles covered here, only seven intervention studies either described the latest programmatic developments or assessed the efficacy of a particular intervention focusing on adherence to ART and retention in healthcare services in the United States in YKP [23,27,32,36–39,42]. Three of these targeted BIY [27,39,42] with two of these studies using mobile phone technology to aid and monitor adherence. The other four intervention studies were conducted on African American YMSM. A randomized control trial of a cell phone adherence support intervention was conducted among HIV-positive BIY in comparison to PIY, where participants were reminded through daily telephone contact to take their ART medication [42]. There appeared to be no differences in adherence behaviours between BIY and PIY. Self-reported adherence was found to be significantly higher among participants belonging to the intervention group than that of the control. There were also medium to large effect sizes on self-reported adherence and viral load during the course of the study. These results indicated that mobile phone technology could be harnessed to encourage youth and adolescents to adhere to medications.

The other four studies covered interventions that are designed to improve ART adherence among HIV-positive African American YMSM [32,36–38]. These programmes provided care which was directed to the unique and complex psychosocial and physical health needs of this particular YKP. The Los Angeles County Department of Public Health actively targeted African American YMSM through community-based outreach services which encouraged participants to visit clinics for counselling and testing [32]. HIV-positive African American YMSM were referred to a youth-focused case management intervention. A study team from the Special Projects of National Significance then assessed these beneficiaries and found that over the first two years of study, only 11% of beneficiaries missed appointments for unknown reasons. Other factors that were associated with retention were feeling respected by medical staff and being in receipt of programme services. These findings suggested that the “youth centred” nature of these programmes may increase retention in treatment as YMSM feel respected and are given access to other services which catered to their psychosocial needs.

A few interventions that focused on treatment of HIV-positive YMSM tried to increase retention in healthcare services through developing and nurturing social and medical networks which could assist YMSM in overcoming individual and environmental level barriers to attending facilities [37,38]. For instance, Project nGage attempted to harness existing social support networks of HIV-positive YMSM to identify “support confidants” who provided beneficiaries with psychosocial assistance [38]. In addition, the Strength Through Youth Livin’ Empowered intervention used social marketing campaigns to target the social and sexual networks of potential peer leaders, which were later cultivated to provide the basis of a medical support network for those who had been newly diagnosed as HIV-positive [37]. Over a three-year period, 81 men were diagnosed or re-engaged in healthcare services and the odds of patients attending clinic visits increased two fold.

In summary, there have been programmes which have attempted to cater to the unique needs of this population using innovative methods, such as mobile phone technology. Magnus *et al*. [32] collected data from eight clinics across the United States to assess such programmes, and demonstrated that it was possible to refine existing youth-centred programmes to increase retention in care in YKP through the development of networks of providers, peer support groups and community-based services. Unfortunately, many of these studies reported the findings of small-scale pilot interventions which often had small sample sizes and were underpowered, hence limiting the generalizability of the results to the wider population of YKP [36,42]. For example, in Puccio *et al*. [27], the evaluation of an intervention using mobile phone technology had a sample of only eight participants.

## Conclusions

As the paradigm of HIV prevention has shifted to treatment-as-prevention strategies, it is necessary to identify factors that promote adherence to and retention in care to antiretroviral regimens among HIV-positive youth and adolescents [13]. Our literature search identified only a handful of studies on adherence to and retention in ART among YKP; seven of them on YMSM and two on YPWID. We expanded the review to include 16 studies on BIY; some of these studies did not specify risk behaviours thus we cannot be confident as to whether these studies have included YKP. Nonetheless, studies on BIY do provide a useful overview of types of factors which may affect retention in care in YKP and how their adherence behaviours may differ from those of PIY.

Most studies focused on YKP that are relevant to the HIV epidemic in the United States and Canada, which were BIY, YMSM belonging to ethnic minorities and YPWID. We were unable to find any research on certain groups of YKP identified by the WHO, including female sex workers and transgendered youth; although, it has been reported that these populations are more susceptible to HIV infection due to sexual exploitation, poverty, violence and stigma [9,48], all of which have the potential to impede their access to HIV services should they contract HIV.

Research on BIY and YKP highlighted that they suffered from a combination of individual- and environmental-level barriers to adherence, as a result of intersecting vulnerabilities owing to the fact that they experience from multiple forms of oppression. In many studies, the bulk of respondents belonged to ethnic minorities who have been historically marginalized in a Western context, were sometimes isolated from their social and familial networks and experienced bouts of imprisonment and housing instability. It was noted by researchers that respondents’ adherence to medication was found not necessarily to be affected by the existence of barriers but rather by the intensity and nature of these barriers. It was found in a few studies that respondents who took recreational drugs, experienced depression and were unable to afford private medical insurance were less adherent than those who did not report these barriers [19,26,34,35].

A recent study demonstrated that YKP were able to overcome these barriers and in some cases display higher rates of adherence to ART than their perinatally infected counterparts [18]. The results of these studies broke down common stereotypes associated with YKP by demonstrating that many adopted a responsible stance towards their treatment as they had few social or familial networks to rely on. This finding also suggested some YKP demonstrated strength and resilience in coping with challenges in engaging in HIV medical care.

In contrast, there exist a worrying paucity of research on adherence in YKP in developing countries as their health behaviours can vary by area. A systematic meta-analysis of studies mostly assessing adherence to ART in PIY illustrated that respondents living in Asian (84%, 95% CI 77–91) and African (84%, 95% CI 79–89) countries displayed higher rates of adherence than those located in North America (53%, 95% CI 46–59) [18]. As none of the studies conducted in Africa or Asia recorded the route of transmission or specifically targeted YKP, it was difficult to know if there was a unique set of factors influencing their adherence to treatment.

Taking all these findings together, we conclude that among YKP, individual and environmental factors including access to psychosocial support, experience of stigma, access to social and behavioural support, and socioeconomic status are important determinants to adherence behaviours. Existing intervention studies suggest that mobile phone technology, social marketing and support for social network may improve adherence among YKP, particularly YMSM. More research on young female sex workers, young transgenders and young offenders is urgently needed. While these populations are hidden and difficult to access, research studies in the United States and Canada demonstrate that accessing these populations is possible through developing research networks between academic institutions and clinics that provide services to these populations. The contexts in which other YKP seeking treatment and engaging in HIV care continuum are likely to be different and each YKP will require culturally tailored interventions to promote retention in and adherence to ART.

Structural factors such as added stigma and discrimination, marginalization, lack of social and family support, and poverty would most likely be barriers that impede YKP from continuing HIV care and treatment. Policy guidelines thus must undergo a paradigm shift to focus specifically on YKP and their unique needs as opposed to their adult counterparts.

## References

[1] World Health Organization (2013). HIV and adolescents.

[2] Arrive E, Dicko F, Amghar H, Aka AE, Dior H, Bouah B (2012). HIV status disclosure and retention in care in HIV-infected adolescents on antiretroviral therapy (ART) in West Africa. PLoS One.

[3] Bhattacharya M, Dubey AP, Sharma M (2011). Patterns of diagnosis disclosure and its correlates in HIV-infected North Indian children. J Trop Pediatr.

[4] Kallem S, Renner L, Ghebremichael M, Paintsil E (2011). Prevalence and pattern of disclosure of HIV status in HIV-infected children in Ghana. AIDS Behav.

[5] Philbin MM, Tanner AE, DuVal A, Ellen JM, Xu J, Kapogiannis B (2014). Factors affecting linkage to care and engagement in care for newly diagnosed HIV-positive adolescents within fifteen Adolescent Medicine Clinics in the United States. AIDS Behav.

[6] Auld AF, Agolory SG, Shiraishi RW, Wabwire-Mangen F, Kwesigabo G, Mulenga M (2014). Antiretroviral therapy enrollment characteristics and outcomes among HIV-infected adolescents and young adults compared with older adults-seven African countries, 2004–2013. MMWR Morb Mortal Wkly Rep.

[7] Mellins CA, Tassiopoulos K, Malee K, Moscicki AB, Patton D, Smith R (2011). Behavioural health risks in perinatally HIV-exposed youth: co-occurrence of sexual and drug use behaviour, mental health problems, and nonadherence to antiretroviral treatment. AIDS Patient Care STDS.

[8] Nachega JB, Hislop M, Nguyen H, Dowdy DW, Chaisson RE, Regensberg L (2009). Antiretroviral therapy adherence, virologic and immunologic outcomes in adolescents compared with adults in southern Africa. J Acquir Immune Defic Syndr.

[9] Rivers K, Aggleton P (2001). Adolescent sexuality, gender, and the HIV epidemic. BETA.

[10] Naswa S, Marfatia YS (2010). Adolescent HIV/AIDS: issues and challenges. Indian J Sex Transm Dis.

[11] Eaton DK, Kann L, Kinchen S, Shanklin S, Flint KH, Hawkins J (2012). Youth risk behaviour surveillance-United States, 2011. MMWR Surveill Summ.

[12] Wood EB, Hutchinson MK, Kahwa E, Hewitt H, Waldron N (2011). Jamaican adolescent girls with older male sexual partners. J Nurs Scholarsh.

[13] Reisner SL, Mimiaga MJ, Skeer M, Perkovich B, Johnson CV, Safren SA (2009). A review of HIV antiretroviral adherence and intervention studies among HIV-infected youth. Top HIV Med.

[14] Giannattasio A, Officioso A, Continisio GI, Griso G, Storace C, Coppini S (2011). Psychosocial issues in children and adolescents with HIV infection evaluated with a World Health Organization age-specific descriptor system. J Dev Behav Pediatr.

[15] Petersen I, Bhana A, Myeza N, Alicea S, John S, Holst H (2010). Psychosocial challenges and protective influences for socio-emotional coping of HIV+ adolescents in South Africa: a qualitative investigation. AIDS Care.

[16] Williams PL, Storm D, Montepiedra G, Nichols S, Kammerer B, Sirois PA (2006). Predictors of adherence to antiretroviral medications in children and adolescents with HIV infection. Pediatrics.

[17] Abramowitz S, Koenig LJ, Chandwani S, Orban L, Stein R, Lagrange R (2009). Characterizing social support: global and specific social support experiences of HIV-infected youth. AIDS Patient Care STDS.

[18] Kim S, Gerver SM, Fidler S, Ward H (2014). Adherence to antiretroviral therapy in adolescents living with HIV: systematic review and meta-analysis. AIDS.

[19] Tapp C, Milloy M-J, Kerr T, Zhang R, Guillemi S, Hogg RS (2011). Female gender predicts lower access and adherence to antiretroviral therapy in a setting of free healthcare. BMC Infect Dis.

[20] Belzer ME, Fuchs DN, Luftman GS, Tucker DJ (1999). Antiretroviral adherence issues among HIV-positive adolescents and young adults. J Adolesc Health.

[21] Martinez J, Bell D, Camacho R, Henry-Reid LM, Bell M, Watson C (2000). Adherence to antiviral drug regimens in HIV-infected adolescent patients engaged in care in a comprehensive adolescent and young adult clinic. J Natl Med Assoc.

[22] Murphy DA, Wilson CM, Durako SJ, Muenz LR, Belzer M, Adolescent Medicine HIV/AIDS Research Network (2001). Antiretroviral medication adherence among the REACH HIV-infected adolescent cohort in the USA. AIDS Care.

[23] Rogers AS, Miller S, Murphy DA, Tanney M, Fortune T (2001). The TREAT (Therapeutic Regimens Enhancing Adherence in Teens) program: theory and preliminary results. J Adolesc Health.

[24] Murphy DA, Sarr M, Durako SJ, Moscicki AB, Wilson CM, Muenz LR (2003). Barriers to HAART adherence among human immunodeficiency virus-infected adolescents. Arch Pediatr Adolesc Med.

[25] Flynn PM, Rudy BJ, Douglas SD, Lathey J, Spector SA, Martinez J (2004). Virologic and immunologic outcomes after 24 weeks in HIV type 1-infected adolescents receiving highly active antiretroviral therapy. J Infect Dis.

[26] Murphy DA, Belzer M, Durako SJ, Sarr M, Wilson CM, Muenz LR (2005). Longitudinal antiretroviral adherence among adolescents infected with human immunodeficiency virus. Arch Pediatr Adolesc Med.

[27] Puccio JA, Belzer M, Olson J, Martinez M, Salata C, Tucker D (2006). The use of cell phone reminder calls for assisting HIV-infected adolescents and young adults to adhere to highly active antiretroviral therapy: a pilot study. AIDS Patient Care STDS.

[28] Naar-King S, Templin T, Wright K, Frey M, Parsons JT, Lam P (2006). Psychosocial factors and medication adherence in HIV-positive youth. AIDS Patient Care STDS.

[29] Rao D, Kekwaletswe TC, Hosek S, Martinez J, Rodriguez F (2007). Stigma and social barriers to medication adherence with urban youth living with HIV. AIDS Care.

[30] Rudy BJ, Murphy DA, Harris DR, Muenz L, Ellen J, Adolescent Trials Network for HIV/AIDS Interventions (2009). Patient-related risks for nonadherence to antiretroviral therapy among HIV-infected youth in the United States: a study of prevalence and interactions. AIDS Patient Care STDS.

[31] Garvie PA, Wilkins ML, Young JC (2010). Young, medication adherence in adolescents with behaviourally-acquired HIV: evidence for using a multimethod assessment protocol. J Adolesc Health.

[32] Magnus M, Jones K, Phillips G, Binson D, Hightow-Weidman LB, Richards-Clarke C (2010). Characteristics associated with retention among African American and Latino adolescent HIV-positive men: results from the outreach, care, and prevention to engage HIV-seropositive young MSM of color special project of national significance initiative. J Acquir Immune Defic Syndr.

[33] Comulada WS, Swendeman DT, Rotheram-Borus MJ, Mattes KM, Weiss RE (2003). Use of HAART among young people living with HIV. Am J Health Behav.

[34] Agwu AL, Fleishman JA, Korthuis PT, Siberry GK, Ellen JM, Gaur AH (2011). Disparities in antiretroviral treatment: a comparison of behaviourally HIV-infected youth and adults in the HIV Research Network. J Acquir Immune Defic Syndr.

[35] Hadland SE, Milloy MJ, Kerr T, Zhang R, Guillemi S, Hogg RS (2012). Young age predicts poor antiretroviral adherence and viral load suppression among injection drug users. AIDS Patient Care STDS.

[36] Wohl AR, Garland WH, Wu J, Au CW, Boger A, Dierst-Davies R (2011). A youth-focused case management intervention to engage and retain young gay men of color in HIV care. AIDS Care.

[37] Hightow-Weidman LB, Smith JC, Valera E, Matthews DD, Lyons P (2011). Keeping them in “STYLE”: finding, linking, and retaining young HIV-positive black and Latino men who have sex with men in care. AIDS Patient Care STDS.

[38] Bouris A, Voisin D, Pilloton M, Flatt N, Eavou R, Hampton K (2013). Project nGage: network supported HIV care engagement for younger black men who have sex with men and transgender persons. J AIDS Clin Res.

[39] Barnes WM, Abramowitz S, Lagrange R, Chandwani S, Moschel DBA, Koenig LJ (2013). Disease-specific knowledge among HIV-infected adolescents: what do they know and how do they learn it?. J HIV AIDS Soc Serv.

[40] Gillman J, Davila J, Sansgiry S, Parkinson-Windross D, Miertschin N, Mitts B (2013). The effect of conspiracy beliefs and trust on HIV diagnosis, linkage, and retention in young MSM with HIV. J Health Care Poor Underserved.

[41] Harper GW, Fernandez IM, Bruce D, Hosek SG, Jacobs RJ, Adolescent Medicine Trials Network for HIV/AIDS Interventions (2013). The role of multiple identities in adherence to medical appointments among gay/bisexual male adolescents living with HIV. AIDS Behav.

[42] Belzer ME, Naar-King S, Olson J, Sarr M, Thornton S, Kahana SY (2013). The use of cell phone support for non-adherent HIV-infected youth and young adults: an initial randomized and controlled intervention trial. AIDS Behav.

[43] Saberi P, Mayer K, Vittinghoff E, Naar-King S, The Adolescent Medicine Trials Network for HIV/AIDS Interventions (2014). Correlation between use of antiretroviral adherence devices by HIV-infected youth and plasma HIV RNA and self-reported adherence. AIDS Behav.

[44] Hussen SA, Andes K, Gilliard D, Chakraborty R, Del Rio C, Malebranche DJ (2014). Transition to adulthood and antiretroviral adherence among HIV-positive young black men who have sex with men. Am J Public Health.

[45] Gray GE (2010). Adolescent HIV-causes for concern in southern Africa. PLoS Med.

[46] UNAIDS, WHO (2009). AIDS epidemic update.

[47] El-Bassel N, Shaw SA, Dasgupta A, Strathdee SA (2014). People who inject drugs in intimate relationships: it takes two to combat HIV. Curr HIV/AIDS Rep.

[48] Tolou-Shams M, Stewart A, Fasciano J, Brown LK (2010). A review of HIV prevention interventions for juvenile offenders. J Pediatr Psychol.

# Sub article: Mental health and support among young key populations: an ecological approach to understanding and intervention

**DOI:** 10.7448/IAS.18.2.19429

**Published:** 2015-02-26

**Authors:** Massy Mutumba, Gary W Harper

**Affiliations:** 1 Center for Sexuality and Health Disparities, Department of Health Behavior and Health Education, University of Michigan, Ann Arbor, MI, USA; 2 Joint Clinical Research Center, Kampala, Uganda; 3 Department of Health Behavior and Health Education, University of Michigan, Ann Arbor, MI, USA

**Keywords:** youth, adolescents, psychiatric disorders, psychosocial, HIV/AIDS, treatment, programmes

## Abstract

**Introduction:**

The patterning of the HIV epidemic within young key populations (YKPs) highlights disproportionate burden by mental disorders in these populations. The mental wellbeing of YKPs is closely associated with biological predispositions and psychosocial factors related to YKPs’ sexual and gender identities and socio-economic status. The purpose of this paper is to highlight sources of risk and resilience, as well as identify treatment and supports for mental health disorders (MHDs) among YKPs.

**Discussion:**

This paper utilizes Bronfenbrenner's Bioecological Systems Theory and the Social Stress Model to explore the risk and protective factors for MHDs across YKPs’ ecological systems, and identify current gaps in treatment and support for MHDs among these youth. We emphasize the fluidity and intersections across these categorizations which reinforce the vulnerability of these populations, the lack of concrete data to inform mental health interventions among YKPs, and the need to ground YKP interventions and programmes with human rights principles stipulated in the convention on the rights of a child.

**Conclusions:**

We put forth recommendations for future research and strategies to address the mental wellbeing of YKPs, including the need for integrated interventions that address the multiplicity of risk factors inherent in the multiple group membership, rather than single-focus interventions whilst addressing the unique needs or challenges of YKPs.

## Introduction

HIV infection disproportionately affects youth [1–3] , and the patterning of the HIV epidemic within young key populations (YKPs) underscores the role of mental health disorders (MHDs) in structuring vulnerability of these populations to HIV. For the purposes of this article, YKPs will be defined as sexual minority youth (including gay, bisexual, and lesbian youth and young men who have sex with men regardless of their sexual orientation identity); gender minority youth (specifically transgender and gender non-conforming youth); youth who inject drugs; youth involved in sex work; runaway and homeless youth; and detained or incarcerated youth.

MHDs may increase YKPs’ vulnerability to HIV, and/or alter the course of infection among those already living with HIV [4–6]. Among YKPs, MHDs have been linked to HIV risk behaviours such as early sexual debut, high numbers of sexual partners, low condom use, transactional sex, needle sharing, and drug/alcohol use [4–13]; lower uptake, adherence to, and retention in HIV care [14,15]; and increased risk of AIDS mortality [16]. Moreover, HIV infection also increases the risk of MHDs among YKPs [17,18].

## Epidemiology of MHDs among YKPs

Although adolescence and emerging adulthood is a time of relative positive physical health as measured by traditional indicators such as rates of mortality, chronic disease burden and hospitalizations, it is also a peak time for developing MHDs [19–21] and health-related challenges stemming from participation in high risk behaviours [22,23]. Studies have consistently reported higher rates of MHDs such as major depression, anxiety, conduct disorder, attention-deficit/hyperactivity disorder (ADHD), substance use disorder, alcohol dependence and abuse, suicide, and post-traumatic stress disorder (PTSD) among sexual minority youth [24–28], gender minority youth [25,27,29,30], youth who inject drugs [20,31], detained or incarcerated youth [32–36], runaway and homeless youth [37–39] and youth involved in sex work [40–42], relative to comparable youth populations. It is important to note that the higher rates of MHDs among YKPs are not due to any inherent dysfunction within these youth, but are closely associated with their membership in socially stigmatized minority groups that experience excessive stress in the form of prejudice-related stressful life events, discrimination, rejection and violence [43–45] .

Sexual minority adult populations have a two-fold excess in suicide attempts, and rates of depression, anxiety and substance use disorders are almost twice as high among sexual minorities compared to heterosexual populations [28]. Among sexual minority youth, a review of MHDs found that one third of participants met the criteria for any MHD including 17% for conduct disorder, 15% for major depression and 9% for PTSD [25]. Studies have reported even higher rates of MHDs among gender minority youth relative to comparable youth populations. A cross-sectional study of 515 gender minority persons found that 60% of participants were depressed; the prevalence of attempted suicide in this sample was 32% [29]. A cross-sectional study of 55 transgender youth found that 45% of participants had seriously considered suicide and 26% had attempted suicide [46], while another study of 571 male-to-female transgender persons in New York found that the lifetime prevalence of major depression among youth in this study was 54.7% [47].

More than two thirds of runaway and homeless youth meet the criteria for two or more MHDs including depression, conduct disorders, ADHD and PTSD [48–52]. Rates of attempted suicide among runaway and homeless youth who self-identify as sexual minorities range between 2 and 42% [26]. Detained or incarcerated youth are 10 times more likely to suffer from psychosis and depression compared to youth in the general population [32]. A nationwide review of 57 juvenile justice agencies (*N*=9, 819) found that 51.9% of youth met the criteria for a MHD; one third met the criteria for more than one disorder and about a quarter met the criteria for multiple clusters disorders. In this study, 20.4% reported anxiety, 27% reported disruptive behaviour disorder, 14% reported lifetime suicide attempts and 7.9% reported affective disorders [53]. Data on the mental health of youth involved in sex work are rare, but a study in Goa, India, found that 41.5% of female sex workers under 20 years of age had attempted suicide in the past three months [40]. Rates of physical and sexual violence among youth involved in sex work are high, ranging between 18 and 67% [54,55].

Current data point to sex/gender differences in prevalence of MHDs among YKPs. Gay/bisexual male youth have higher rates of panic and depression disorders, while lesbian/bisexual female youth have higher rates of substance abuse [56]. A study of sexual and gender minority youth found that transgender youth had a lower prevalence of all MHDs compared to gay/bisexual youth [25]. Among runaway and homeless youth, rates of drug abuse among were 10 times higher among male youth and 17 times higher among female youth as compared to youth in a nationally representative sample, and alcohol abuse was significantly higher among male youth [52]. Almost twice as many female runaway and homeless youth (25%) had attempted suicide at least once compared to male runaway and homeless youth (14%) [26]. Among detained or incarcerated youth, rates of major depression were twice as high among female youth compared to male youth (29% vs. 10.6%), while young men reported higher rates of psychotic illness (3.3% vs. 2.7%) [32]. These sex/gender differences underscore the diversity in experiences and needs within specific YKPs, which may have significant implications for intervention development. However, more studies are needed to elaborate on these differences.

### Cross-cutting issues

The needs and challenges of YKPs vary with their age, sex, race/ethnicity, gender identity, sexual identity, socio-economic status and geographic region. However, YKPs also share a host of socio-ecological experiences, broadly engendered by their sexual orientation and gender identities, which confer selective risks and vulnerabilities for MHDs and HIV. The categorizations of YKPs are not mutually exclusive (see Figure 1): there is high fluidity and intersections across these categories [57,58]. For example, approximately 30–45% of clients served in homeless youth services are sexual minority youth [59]; compared to heterosexual female youth, lesbian and bisexual youth are over-represented among detained or incarcerated youth [60,61]; homelessness is associated with greater risks for substance abuse [62]; and runaway and homeless youth who also self-identify as gay are more likely to report being tested or treated for HIV compared to bisexual or heterosexual youth [58,63] and are also more likely to engage in substance and alcohol abuse [64].

**Figure 1:** Intersection of group membership and identities among young key populations.

This intersectionality of oppressed identities and multiple memberships among YKP categories that experience social marginalization may increase the presence of MHDs. This is supported by the syndemic production theory, which posits that for some marginalized groups (e.g. sexual minority youth), there is a syndemic process of interacting physical and psychosocial challenges (e.g. HIV, substance use, depression, violence) that cause poor health outcomes within these populations [65,66]. For this reason, we emphasize the importance of developing interventions that address the intersectionality of social and cultural identities possessed by YKPs and the multiplicity of risk and resilience factors that may accompany membership in these various groups, rather than single-focus interventions.

## Discussion

### Theoretical framework

This paper utilizes two theoretical frameworks based on diathesis-stress models, to situate the epidemiology of MHDs among YKPs: Bronfenbrenner's Bioecological Systems Theory (BST) [67,68], and the Social Stress Model (SST) [69–71]. Generally, diathesis-stress models assert that all people have some level of pre-disposing risk factors (biological diathesis) for any given MHD, and that stress activates a diathesis, transforming the potential pre-disposition into an MHD [72–74]. BST is useful in understanding the linkages between biological factors and psychosocial factors in the development of MHDs among YKPs [69,70]. It proposes that an individual is continually impacted by four successive and interconnected levels of influence (i.e. microsystem, mesosystem, exosystem and macrosystem) over their life course; the biological diatheses and ecological stressors may act directly or synergistically to increase an individual's risk for MHDs. SST posits that one's disadvantaged position in the social hierarchy leads to more stressful conditions and fewer resources to counteract these stressors, resulting in greater rates of MHDs.

The *microsystem* comprises the complex relations between the developing person and the environments in the immediate settings containing the person. The *mesosystem* is a set of microsystems constituting an individual's developmental niche within a given period of development; mesosystems are more challenging to quantify and represent the assumption that microsystems do not function independently. The *exosystem* is composed of contexts that do not directly involve the developing person but have an influence on the person's behaviour and development. The *macrosystem* is the super ordinate ecological level of human development, involving culture, macro-institutions and public policy [75].

BST also provides a useful theoretical framework for understanding nested ecological system factors that influence the mental health and HIV risk/protective behaviours of YKPs, and the reciprocal relationships between youth and their environments. Both the BST and SST enable examination of youth within their social contexts, thereby allowing identification of contextually relevant cultural and developmental risk and protective factors.

Figure 2 illustrates the adaptation of the SST to MHDs among YKPs. We argue that MHDs among YKPs result from their disadvantaged positions in the social hierarchy within their ecological systems, and this positioning is closely linked to their sexual orientation identity, gender identity and/or socio-economic status [76,77]. Consistent with our diathesis-stress framework, we argue that biological predispositions and ecological stressors may act independently, additively or synergistically to create MHDs and maladaptive HIV risk behaviours, and these pathways may be moderated by resilience factors [78]. This model highlights the reciprocal relationship between maladaptive behaviours and MHDs, which is also moderated by resilience factors.

**Figure 2:** A framework using the Social Stress Theory to depict evolution of mental disorders among young key populations.

### Risk factors

Microsystem factors could be sub-divided into intrapersonal and interpersonal factors. *Intrapersonal factors* include biological or cognitive factors that contribute toward certain abnormal states or conditions including genetic factors, inherited traits, neurological anomalies and patterns of psycho-physiological stress responses [73,74,79]. HIV may affect central nervous system structures involved in the regulation of emotion and behaviour, thereby increasing youth's risk of MHDs [80,81]. Additionally, normative developmental processes such as identity development and increased propensity for risk-taking, psychosocial distress manifested by self-esteem, poor self-image, hopelessness, helplessness and internalized homophobia may increase MHDs and HIV risk behaviours among YKPs [45,82–86]. Adolescence is a peak time for traumatic injuries, which, in turn, increase the risk of MHDs and HIV risk behaviours among youth [87,88]. MHDs such as depression, conduct disorder and PTSD and related dimensions of behaviour including coping strategies have been linked to diatheses such as genetic factors, depressogenic cognitive structures, traumatic brain injury and ecological stressors [72,73,87–89].

Interpersonal factors include experiences of victimization, family conflict, family/peer rejection, social isolation, poverty and housing instability [24,42,45,90–98]. Studies have found high rates of childhood maltreatment (physical and sexual abuse) among youth involved in sex work and runaway and homeless youth [99–102]. Childhood maltreatment, especially childhood sexual abuse, has been associated with alcohol use, delinquency and sexual risk behaviour [103–106] and MHDs such as depression and PTSD [107–109]. The pathways through which childhood physical and sexual abuse result in MHDs and HIV risk behaviours are not clearly elucidated but several authors hypothesize that these traumas could influence the development of maladaptive coping skills, maladaptive social information processing, and feelings of hopelessness, vulnerability and loneliness [110,111], leading to MHDs and HIV risk behaviours.

Exosystem factors include school and neighbourhood safety, neighbourhood poverty, stereotypes and representation of YKPs in communities, absence of caring adults, negative experiences with service providers, dearth of trained mental health providers, and geographical and financial barriers to accessing comprehensive and sensitive mental health services [85,112–116]. Exposure to violence, including war and civil strife, contributes significantly to MHDs, especially among youth in low-income countries [117,118]. The macrosystem factors include stigma, discrimination, social and economic marginalization, criminalizing or disenfranchising public policies, and cost of health care services.

All of these systems of influence act individually or synergistically to heighten YKPs’ risk for MHDs and HIV risk behaviours [42,45,112,119–122]. Different types of stressors (acute or chronic) may play different roles in the aetiology of MHDs and HIV risk behaviours. However, within the current research, there is a lack of theoretical attention to the nature and quality of the stressors, and the complex interactions through which biological diatheses and ecological factors influence the development of MHDs among YKPs.

### Resilience factors

Despite the host of powerful negative forces, not all YKPs have MHDs or engage in HIV risk behaviours. Many have multiple resilience factors – personal traits or characteristics of their social environment that protect young people from harm and reduce the likelihood of MHDs among vulnerable youth [123–125]. Intrapersonal resilience factors include high self-esteem, positive self-image, positive coping strategies, spirituality/religiosity, hopefulness, positive future expectations and participation in support or advocacy networks [85,90,98,123,126,127]. Interpersonal factors such as family and peers play an important role in youth development. Social support is generally hypothesized to be a protective factor that buffers individuals against the potential negative consequences of stressful events [128]. However, social support from family and peers may have differential effects for YKPs, including positive effects [129–133] or no effects at all [91,96,134]. For example, parental support may be more predictive of future MHDs than peer support [135,136]. Family connectedness and positive family acceptance have also been associated with positive mental health outcomes, particularly for sexual and gender minority youth who are grappling with issues of sexual orientation and gender identity [64,85,93,137–140]. Strong peer group affiliations may enhance risky health behaviours such as substance abuse and survival sex among runaway and homeless youth [141–143].

Exosystem factors include the availability of support from caring adults including teachers, case managers, programme facilitators and health providers; access to comprehensive youth-friendly social services with trained providers; non-discriminatory and anti-bullying policies in schools, homeless shelters and detention facilities; and child protection policies. These factors prevent MHDs, increase access to and utilization of health services, and promote YKPs’ ability to desist HIV risk behaviours [63,85,112,122,127,129,144]. Within the exosystem, organizations such as schools, churches, youth centres and health facilities are well positioned to provide safe environments and prevent MHDs among YKPs. For example, health providers could prepare youth and their families for changes related to pubertal development, understand and accept the gender and sexual identity of their children, provide parents/guardians with the skills needed to fully support YKPs and facilitate family re-integration (when appropriate). Schools, detentions centres, homeless shelters and foster homes can institute policies to prevent victimization of YKPs in these environments and advocate for the rights of YKPs. However, the success of these preventive actions requires providers who are knowledgeable and sensitive to the specific needs of YKPs.

### Programmes and interventions

Interventions to prevent or improve MHDs and HIV risk behaviours among YKPs are critical to addressing the HIV epidemic among youth. MHDs among youth are addressed through treatment with pharmacological agents or psychosocial interventions [145]. Current treatment guidelines discourage use of pharmacotherapy among children and adolescents [146]; rather pharmacological agents should only be prescribed if psychosocial interventions prove ineffective. However, compared to adults, the evidence base for management of MHDs and HIV risk behaviours among youth is less established. For example, depression and PTSD are some of the most common MHDs among YKPs [147], but evidence for the effectiveness of medications for treatment of these MHDs among adolescents remains elusive [146,148,149].

Globally, there is a paucity of programmes addressing MHDs and HIV risk among YKPs, and even fewer of these programmes exist in low- and middle-income countries [150]. Psychosocial interventions for management of MHDs among YKPs include interpersonal psychotherapy, cognitive behavioural therapy, behavioural therapy, psychodynamic therapy, structured physical activity programmes, relaxation training, problem-solving therapy and motivational interviewing. Within the adult literature, there is an extensive body of knowledge on the effectiveness of these psychosocial interventions [151] but even so, understanding the exact mechanisms by which these interventions achieve their effects and consensus over the relative effectiveness of different psychosocial therapies is lacking [145,152,153].

There is a paucity of interventions to prevent MHDs among YKPs. Universal and targeted prevention programmes have been developed to address alcohol and substance use and HIV risk behaviours among YKPs [154–156] , but results from systematic reviews of these interventions indicate that the majority do not obtain significantly better mental health outcomes compared to controls [150], and reductions in HIV risk behaviours, if realized, are often short-lived [150,154,157–160] . These findings suggest a need to re-consider strategies for engaging and promoting sustainability of behavioural gains among YKPs.

The persistent fragmentation of services, often with single-focus programmes targeting specific YKPs or MHDs, disregards the co-occurrence of MHDs among YKPs [161] and multiplicity of needs across these intersecting populations, thus limiting the efficacy of these interventions. Commonly cited components of integrated MHD services include comprehensive screening for all MHDs, development of a common treatment plan addressing all conditions, a multi-disciplinary team that includes a specialist in co-occurring disorders and psychosocial and pharmacological interventions, and services such as assertive outreach, coordinated care and supported employment [162].

The World Health Organization advocates for treatment of mental and psychosocial problems within primary care settings, but several concerns abound with this strategy including lack of organizational resources and expertise, gaps in provider knowledge regarding the developmental and mental health needs of YKPs, and lack of cultural competency in addressing needs of YKPs [163,164] . Additionally, YKPs may have significant challenges in accessing services within primary care settings, due to lack of health insurance coverage and concerns about confidentiality and privacy.

## Conclusions

Promoting the wellbeing of YKPs requires culturally and developmentally appropriate primary prevention interventions to eliminate or reduce risk factors for MHDs and HIV risk behaviours, and foster resilience factors throughout YKPs’ ecological environment. In addition, culturally and developmentally appropriate HIV care and mental health services are needed for youth living with HIV and/or MHDs, as well as secondary prevention interventions that promote healthy functioning and life course development for affected YKPs. All youth programmes and services need to address the intersectionality of marginalized identities and group membership often found among YKPs. They should be grounded in the latest theoretical and empirical data related to risk reduction and health promotion, and attend to the cultural and developmental needs of these youth.

While there is a growing body of knowledge regarding MHDs in some YKPs (e.g. sexual minority youth, runaway and homeless youth, detained or incarcerated youth), the literature on other populations such as gender minority youth and youth involved in sex work as well as YKPs in low-income countries continues to lag behind. The majority of studies on YKPs have been conducted in the United States; less is known about the psychosocial challenges or burden of MHDs among YKPs outside of the United States. This challenge is exacerbated by the lack of consistency in how MHDs are conceptualized and measured across countries and cultures [165–168], and differences in how adolescence is defined as a developmental period across settings [169,170]. Future research should focus on developing and validating mental health measures for non-US based populations and assessing the efficacy of these interventions in both US and non-US populations, keeping in mind the importance of tailoring interventions to local contexts.

Additional research is needed to increase understanding of key issues that influence the MHDs and HIV-related behaviours of YKPs, and to inform the development of effective interventions to address the unique needs of these young people. Of particular importance are studies elaborating on the complex pathways through which biological and ecological diatheses influence the development of MHDs and HIV risk behaviours among YKPs, the relative effects of the different types of stressors and appropriate strategies for management of MHDs in young people.

Interventions and research among YKPs would benefit from utilizing a BST framework for understanding the range of ecological factors that impact the MHDs and HIV-related risk and resilience of YKPs and in developing culturally and developmentally appropriate MHD and HIV-focused primary and secondary prevention interventions for YKPs. More research is needed to better understand the burden of MHDs in YKPs (especially outside of the US) and the interaction of MHDs and HIV-related risk and resilience. Such research should be sensitive to the multiple group membership of YKPs in often marginalized populations, addressing the multiplicity of risk and resilience factors across YKPs.

Given the high levels of stigma and discrimination experienced by YKPs globally, we argue that youth interventions should be rooted in the key human rights principles advanced in the convention on the rights of a child [171] including: (1) protection from physical and mental harm and exploitation; (2) utilization of evidence-based practices in establishing programmes and services for children; (3) provision of secure conditions that ensure dignity and promote self-reliance and (4) participation in decision-making processes taken in their regard. These human rights principles should supersede any social, cultural, political and other hegemonic ideologies, which may serve to oppress YKPs.

Below, we provide recommendations for practitioners and researchers on the best practices to promote the mental health and reduce HIV risk behaviours among YKPs:

1. *Consolidate youth services* to address the multiplicity of risk factors, and resulting MHDs and HIV risk behaviours. Develop partnerships across public, private and civil organizations to address the multiplicity of risk factors and special needs within YKPs, whilst attending to the individual needs of each youth. Such services should promote YKPs’ access to and sustained engagement in mental health services, HIV continuum of care and youth development programmes.
2. *Tailor programmes and interventions* to biological, cognitive, social and identity development stage of YKPs because there are wide variations in developmental differences between adolescents and young adults within YKPs.
3. *Expand training for providers in mental health*, particularly in low- and middle-income countries with a dearth of trained clinicians. This training should equip providers with the knowledge and skills to promote positive adolescent development and address the needs of YKPs through affirmative and respectful approaches.
4. *Create supportive environments* within programmes and services, and foster positive youth development by strengthening family, peers, school and community support systems. Family-centred interventions that enhance parent's/guardian's ability to connect with and support youth grappling with various psychosocial issues especially gender and sexual identity, and prevent risk factors such as family abuse, rejection and poor parent-youth communication and support are critical to preventing MHDs and HIV risk behaviours among YKPs.
5. *Develop youth capabilities* and *critical consciousness* by equipping youth with knowledge, skills and resources to counter their varied challenges [172] and provide opportunities for YKPs to participate in their socio-political environments.
6. *Develop and enforce formal child protection systems*, *policies and guidelines* in institutions such as schools and juvenile justice systems to prevent re-victimization of YKPs, and ensure access to mental health and HIV care services as well as positive youth development programmes within these contexts.

## References

[1] Hall HI, Song R, Rhodes P, Prejean J, An Q, Lee LM (2008). Estimation of HIV incidence in the United States. JAMA.

[2] Centers for Disease Control and Prevention (CDC) (2011). HIV surveillance – United States, 1981–2008. MMWR.

[3] UNICEF (2014). Monitoring the situation of children and women. http://data.unicef.org/hiv-aids/adolescents-young-people.

[4] Donenberg GR (2005). Youths and HIV/AIDS: psychiatry's role in a changing epidemic. J Am Acad Child Adolesc Psychiatry.

[5] Brown LK, Danovsky MB, Lourie KJ, DiClemente RJ, Ponton LE (1997). Adolescents with psychiatric disorders and the risk of HIV. J Am Acad Child Adolesc Psychiatry.

[6] DiClemente RJ, Ponton LE (1993). HIV-related risk behaviors among psychiatrically hospitalized adolescents and school-based adolescents. Am J Psychiatry.

[7] Rotheram-Borus MJ, Koopman C (1991). Sexual risk behaviors, AIDS knowledge, and beliefs about AIDS among runaways. Am J Public Health.

[8] Tubman JG, Gil AG, Wagner EF, Artigues H (2003). Patterns of sexual risk behaviors and psychiatric disorders in a community sample of young adults. J Behav Med.

[9] Stewart AJ, Theodore-Oklota C, Hadley W, Brown LK, Donenberg G, DiClemente R (2012). Mania symptoms and HIV-risk behavior among adolescents in mental health treatment. J Clin Child Adolesc Psychol.

[10] Elkington KS, Bauermeister JA, Zimmerman MA (2010). Psychological distress, substance use, and HIV/STI risk behaviors among youth. J Youth Adolesc.

[11] Lehrer JA, Shrier LA, Gortmaker S, Buka S (2006). Depressive symptoms as a longitudinal predictor of sexual risk behaviors among US middle and high school students. Pediatrics.

[12] Tolou-Shams M, Brown LK, Houck C, Lescano CM (2007). The association between depressive symptoms, substance use, and HIV risk among youth with an arrest history. J Stud Alcohol Drugs.

[13] 
Teplin LA, Elkington KS, McClelland GM, Abram KM, Mericle AA, Washburn JJ (2005). Major mental disorders, substance use disorders, comorbidity, and HIV-AIDS risk behaviors in juvenile detainees. Psychiatr Serv.

[14] Naar-King S, Templin T, Wright K, Frey M, Parsons JT, Lam P (2006). Psychosocial factors and medication adherence in HIV-positive youth. AIDS Patient Care STDs.

[15] Hosek SG, Harper GW, Domanico R (2005). Predictors of medication adherence among HIV-infected youth. Psychol Health Med.

[16] Leserman J (2003). HIV disease progression: depression, stress, and possible mechanisms. Biol Psychiatry.

[17] Fielden S, Sheckter L, Chapman G, Alimenti A, Forbes J, Sheps S (2006). Growing up: perspectives of children, families and service providers regarding the needs of older children with perinatally-acquired HIV. AIDS Care.

[18] Mellins CA, Malee KM (2013). Understanding the mental health of youth living with perinatal HIV infection: lessons learned and current challenges. J Int AIDS Soc.

[19] Kessler RC, Berglund P, Demler O, Jin R, Merikangas KR, Walters EE (2005). Lifetime prevalence and age-of-onset distributions of dsm-iv disorders in the national comorbidity survey replication. Arch Gen Psychiatry.

[20] Merikangas KR, He JP, Burstein M, Swanson SA, Avenevoli S, Cui L (2010). Lifetime prevalence of mental disorders in US adolescents: results from the National Comorbidity Survey Replication Adolescent Supplement (NCS-A). J Am Acad Child Adolesc Psychiatry.

[21] Kessler RC, Angermeyer M, Anthony JC, de Graaf R, Demyttenaere K, Gasquet I (2007). Lifetime prevalence and age-of-onset distributions of mental disorders in the World Health Organization's World Mental Health Survey Initiative. World Psychiatry.

[22] Millstein SG, Petersen AC, Nightingale EO (1993). Promoting the health of adolescents: new directions for the twenty-first century.

[23] Rivara FP, Park MJ, Irwin CE, DiClemente R, Santelli J, Crosby R, Ralph J. DiClemente, John S. Santelli, Richard A. Crosby (2009). Trends in adolescent and young adult morbidity and mortality. Adolescent health: understanding and preventing risk behaviors.

[24] Hatzenbuehler ML (2011). The social environment and suicide attempts in lesbian, gay, and bisexual youth. Pediatrics.

[25] Mustanski BS, Garofalo R, Emerson EM (2010). Mental health disorders, psychological distress, and suicidality in a diverse sample of lesbian, gay, bisexual, and transgender youths. Am J Public Health.

[26] Leslie MB, Stein JA, Rotheram-Borus MJ (2002). Sex-specific predictors of suicidality among runaway youth. J Clin Child Adolesc Psychol.

[27] Almeida J, Johnson RM, Corliss HL, Molnar BE, Azrael D (2009). Emotional distress among LGBT youth: the influence of perceived discrimination based on sexual orientation. J Youth Adolesc.

[28] King M, Semlyen J, Tai SS, Killaspy H, Osborn D, Popelyuk D (2008). A systematic review of mental disorder, suicide, and deliberate self harm in lesbian, gay and bisexual people. BMC Psychiatry.

[29] Clements-Nolle K, Marx R, Katz M (2006). Attempted suicide among transgender persons: the influence of gender-based discrimination and victimization. J Homosex.

[30] Wallien MS, Swaab H, Cohen-Kettenis PT (2007). Psychiatric comorbidity among children with gender identity disorder. J Am Acad Child Adolesc Psychiatry.

[31] Conway KP, Compton W, Stinson FS, Grant BF (2006). Lifetime comorbidity of DSM-IV mood and anxiety disorders and specific drug use disorders: results from the National Epidemiologic Survey on Alcohol and Related Conditions. J Clin Psychiatry.

[32] Fazel S (2008). Mental disorders among adolescents in juvenile detention and correctional facilities: a systematic review and metaregression analysis of 25 surveys. J Am Acad Child Adolesc Psychiatry.

[33] Colins O, Vermeiren R, Vreugdenhil C, van den Brink W, Doreleijers T, Broekaert E (2010). Psychiatric disorders in detained male adolescents: a systematic literature review. Can J Psychiatry.

[34] Quinn MM, Osher DM, Poirier JM, Rutherford RB, Leone PE (2005). Youth with disabilities in juvenile corrections: a national survey. Except Child.

[35] Krezmien MP, Mulcahy CA, Leone PE (2008). Detained and committed youth: examining differences in achievement, mental health needs, and special education status. Educ Treat Children.

[36] Teplin LA, Abram KM, McClelland GM, Dulcan MK, Mericle AA (2002). Psychiatric disorders in youth in juvenile detention. Arch Gen Psychiatry.

[37] Kirst MJ, Erickson P, Strike C (2009). Poly-substance use among male and female street youth in Toronto, Canada. Int J Soc Inq.

[38] Cleverley K, Kidd SA (2011). Resilience and suicidality among homeless youth. J Adolesc.

[39] Johnson KD, Whitbeck LB, Hoyt DR (2005). Substance abuse disorders among homeless and runaway adolescents. J Drug Issues.

[40] Shahmanesh M, Wayal S, Cowan F, Mabey D, Copas A, Patel V (2009). Suicidal behavior among female sex workers in Goa, India: the silent epidemic. Am J Public Health.

[41] Burgos M, Richter DL, Reininger B, Coker AL, Saunders R, Alegria M (1999). Street based female adolescent Puerto Rican sex workers: contextual issues and health needs. Fam Community Health.

[42] Silverman JG (2011). Adolescent female sex workers: invisibility, violence and HIV. Arch Dis Child.

[43] Meyer IH (2003). Prejudice, social stress, and mental health in lesbian, gay, and bisexual populations: conceptual issues and research evidence. Psychol Bull.

[44] Institute of Medicine (2011). The health of lesbian, gay, bisexual, and transgender people: building a foundation for better understanding.

[45] Harper GW, Schneider M (2003). Oppression and discrimination among lesbian, gay, bisexual, and transgendered people and communities: a challenge for community psychology. Am J Community Psychol.

[46] Grossman AH, D'Augelli AR (2007). Transgender youth and life-threatening behaviors. Suicide Life Threat Behav.

[47] Nuttbrock L, Hwahng S, Bockting W, Rosenblum A, Mason M, Macri M (2010). Psychiatric impact of gender-related abuse across the life course of male-to-female transgender persons. J Sex Res.

[48] Adlaf EM, Zdanowicz YM (1999). A cluster-analytic study of substance problems and mental health among street youths. Am J Drug Alcohol Abuse.

[49] Merscham C, Van Leeuwen JM, McGuire M (2008). Mental health and substance abuse indicators among homeless youth in Denver, Colorado. Child Welfare.

[50] Slesnick N, Prestopnik J (2005). Dual and multiple diagnosis among substance using runaway youth. Am J Drug Alcohol Abuse.

[51] Whitbeck LB, Hoyt DR, Bao WN (2000). Depressive symptoms and co-occurring depressive symptoms, substance abuse, and conduct problems among runaway and homeless adolescents. Child Dev.

[52] Whitbeck LB, Johnson KD, Hoyt DR, Cauce AM (2004). Mental disorder and comorbidity among runaway and homeless adolescents. J Adolesc Health.

[53] Wasserman GA, McReynolds LS, Schwalbe CS, Keating JM, Jones SA (2010). Psychiatric disorder, comorbidity, and suicidal behavior in juvenile justice youth. Crim Justice Behav.

[54] Decker MR, McCauley HL, Phuengsamran D, Janyam S, Silverman JG (2010). Sex trafficking, sexual risk, sexually transmitted infection and reproductive health among female sex workers in Thailand. J Epidemiol Community Health.

[55] Beattie TS, Bhattacharjee P, Ramesh B, Gurnani V, Anthony J, Isac S (2010). Violence against female sex workers in Karnataka state, south India: impact on health, and reductions in violence following an intervention program. BMC Public Health.

[56] Haas AP, Eliason M, Mays VM, Mathy RM, Cochran SD, D'Augelli AR (2010). Suicide and suicide risk in lesbian, gay, bisexual, and transgender populations: review and recommendations. J Homosex.

[57] Frederick T, Dana Peterson, Vanessa R. Panfill (2014). Diversity at the margins: the interconnections between homelessness, sex work, mental health, and substance use in the lives of sexual minority homeless young people. Handbook of LGBT communities, crime, and justice.

[58] Garofalo R, Deleon J, Osmer E, Doll M, Harper GW (2006). Overlooked, misunderstood and at-risk: exploring the lives and HIV risk of ethnic minority male-to-female transgender youth. J Adolesc Health.

[59] Durso LE, Gates GJ (2012). Serving our youth: findings from a national survey of service providers working with lesbian, gay, bisexual, and transgender youth who are homeless or at risk of becoming homeless.

[60] 
Belknap J, Holsinger K, Little JS, Dana Peterson, Vanessa R. Panfill (2014). Lesbian, gay, and bisexual youth incarcerated in delinquent facilities. Handbook of LGBT communities, crime, and justice.

[61] Himmelstein KE, Brackner H (2011). Criminal-justice and school sanctions against nonheterosexual youth: a national longitudinal study. Pediatrics.

[62] Rosario M, Schrimshaw EW, Hunter J (2012). Homelessness among lesbian, gay, and bisexual youth: implications for subsequent internalizing and externalizing symptoms. J Youth Adolesc.

[63] Rew L (2008). Caring for and connecting with homeless adolescents. Fam Community Health.

[64] Friedman CK, Morgan EM (2009). Comparing sexual-minority and heterosexual young womens’ friends and parents as sources of support for sexual issues. J Youth Adolesc.

[65] Stall R, Friedman M, Catania JA, Wolitski RJ, Stall R, Valdiserri RO (2008). Interacting epidemics and gay men's health: a theory of syndemic production among urban gay men. Unequal opportunity: health disparities affecting gay and bisexual men in the United States.

[66] Bruce D, Harper GW (2011). Operating without a safety net: gay male adolescents and emerging adults’ experiences of marginalization and migration, and implications for theory of syndemic production of health disparities. Health Educ Behav.

[67] Bronfenbrenner U, Phyllis Moen, Glenn H. Elder, Kurt Luscher (1995). Developmental ecology through space and time: a future perspective. Examining lives in context: perspectives on the ecology of human development.

[68] Bronfenbrenner U (2005). Making human beings human: bioecological perspectives on human development.

[69] Horwitz AV, Carol S. Aneshensel, Jo C. Phelan, Alex Bierman (1999). The sociological study of mental illness. Handbook of the sociology of mental health.

[70] Miranda J, McGuire T, Williams D, Wang P (2008). Mental health in the context of health disparities. Am J Psychiatry.

[71] Schwartz S, Meyer IH (2010). Mental health disparities research: the impact of within and between group analyses on tests of social stress hypotheses. Soc Sci Med.

[72] McKeever VM, Huff ME (2003). A diathesis-stress model of posttraumatic stress disorder: ecological, biological, and residual stress pathways. Rev Gen Psychol.

[73] Rende R, Plomin R (1992). Diathesis-stress models of psychopathology: a quantitative genetic perspective. Appl Prev Psychol.

[74] Monroe SM, Simons AD (1991). Diathesis-stress theories in the context of life stress research: implications for the depressive disorders. Psychol Bull.

[75] Bronfenbrenner U (1977). Toward an experimental ecology of human development. Am Psychol.

[76] Reisner SL, Greytak EA, Parsons JT, Ybarra ML (2014). Gender minority social stress in adolescence: disparities in adolescent bullying and substance use by gender identity. J Sex Res.

[77] Noell JW, Ochs LM (2001). Relationship of sexual orientation to substance use, suicidal ideation, suicide attempts, and other factors in a population of homeless adolescents. J Adolesc Health.

[78] Moskowitz A, Stein JA, Lightfoot M (2013). The mediating roles of stress and maladaptive behaviors on self-harm and suicide attempts among runaway and homeless youth. J Youth Adolesc.

[79] Dodge KA, Pettit GS (2003). A biopsychosocial model of the development of chronic conduct problems in adolescence. Dev Psychol.

[80] Sowell ER, Peterson BS, Thompson PM, Welcome SE, Henkenius AL, Toga AW (2003). Mapping cortical change across the human life span. Nat Neurosci.

[81] Sharer LR (1992). Pathology of HIV-1 infection of the central nervous system. A review. J Neuropathol Exp Neurol.

[82] Arnett J (1992). Reckless behavior in adolescence: a developmental perspective. Dev Rev.

[83] Arnett JJ (1999). Adolescent storm and stress, reconsidered. Am Psychol.

[84] Greene K, Krcmar M, Walters LH, Rubin DL, Hale L (2000). Targeting adolescent risk-taking behaviors: the contributions of egocentrism and sensation-seeking. J Adolesc.

[85] Fenaughty J, Harré N (2003). Life on the seesaw: a qualitative study of suicide resiliency factors for young gay men. J Homosex.

[86] Herbert SE (2011). Female-to-male transgender adolescents. Child Adolesc Psychiatr Clin N Am.

[87] Mackelprang JL, Harpin SB, Grubenhoff JA, Rivara FP (2014). Adverse outcomes among homeless adolescents and young adults who report a history of traumatic brain injury. Am J Public Health.

[88] Huw Williams W, Cordan G, Mewse AJ, Tonks J, Burgess CN (2010). Self-reported traumatic brain injury in male young offenders: a risk factor for re-offending, poor mental health and violence?. Neuropsychol Rehabil.

[89] Lewinsohn PM, Joiner TE, Rohde P (2001). Evaluation of cognitive diathesis-stress models in predicting major depressive disorder in adolescents. J Abnorm Psychol.

[90] Grossman AH, D'augelli AR, Frank JA (2011). Aspects of psychological resilience among transgender youth. J LGBT Youth.

[91] Hershberger SL, D'Augelli AR (1995). The impact of victimization on the mental health and suicidality of lesbian, gay, and bisexual youths. Dev Psychol.

[92] Van Bergen DD, Bos HM, van Lisdonk J, Keuzenkamp S, Sandfort TG (2013). Victimization and suicidality among Dutch lesbian, gay, and bisexual youths. Am J Public Health.

[93] Ryan C, Huebner D, Diaz RM, Sanchez J (2009). Family rejection as a predictor of negative health outcomes in White and Latino Lesbian, Gay, and bisexual young adults. Pediatrics.

[94] Russell ST, Ryan C, Toomey RB, Diaz RM, Sanchez J (2011). Lesbian, gay, bisexual, and transgender adolescent school victimization: implications for young adult health and adjustment. J School Health.

[95] Kidd SA (2007). Youth homelessness and social stigma. J Youth Adolesc.

[96] Kidd SA, Carroll MR (2007). Coping and suicidality among homeless youth. J Adolesc.

[97] Keuroghlian AS, Shtasel D, Bassuk EL (2014). Out on the street: a public health and policy agenda for lesbian, gay, bisexual, and transgender youth who are homeless. Am J Orthopsychiatry.

[98] Mc Elroy S, Hevey D (2014). Relationship between adverse early experiences, stressors, psychosocial resources and wellbeing. Child Abuse Neglect.

[99] Wilson HW, Widom CS (2010). The role of youth problem behaviors in the path from child abuse and neglect to prostitution: a prospective examination. J Res Adolesc.

[100] Simons RL, Whitbeck LB (1991). Sexual abuse as a precursor to prostitution and victimization among adolescent and adult homeless women. J Family Issues.

[101] Rosario M, Schrimshaw EW, Hunter J (2012). Risk factors for homelessness among lesbian, gay, and bisexual youths: a developmental milestone approach. Child Youth Serv Rev.

[102] Tyler KA, Melander LA (2013). Child abuse, street victimization, and substance use among homeless young adults. Youth Society.

[103] Wilson HW, Widom CS (2008). An examination of risky sexual behavior and HIV in victims of child abuse and neglect: a 30-year follow-up. Health Psychol.

[104] Jones DJ, Runyan DK, Lewis T, Litrownik AJ, Black MM, Wiley T (2010). Trajectories of childhood sexual abuse and early adolescent HIV/AIDS risk behaviors: the role of other maltreatment, witnessed violence, and child gender. J Clin Child Adolesc Psychol.

[105] Lansford JE, Miller-Johnson S, Berlin LJ, Dodge KA, Bates JE, Pettit GS (2007). Early physical abuse and later violent delinquency: a prospective longitudinal study. Child Maltreat.

[106] Jespersen AF, Lalumière ML, Seto MC (2009). Sexual abuse history among adult sex offenders and non-sex offenders: a meta-analysis. Child Abuse Negl.

[107] Trickett PK, Noll JG, Putnam FW (2011). The impact of sexual abuse on female development: lessons from a multigenerational, longitudinal research study. Dev Psychopathol.

[108] Trickett PK, Negriff S, Ji J, Peckins M (2011). Child maltreatment and adolescent development. J Res Adolesc.

[109] Noll JG, Haralson KJ, Butler EM, Shenk CE (2011). Childhood maltreatment, psychological dysregulation, and risky sexual behaviors in female adolescents. J Pediatr Psychol.

[110] 
Engel CC, Engel AL, Campbell SJ, McFall ME, Russo J, Katon W (1993). Posttraumatic stress disorder symptoms and precombat sexual and physical abuse in Desert Storm veterans. J Nerv Ment Dis.

[111] Dodge KA, Pettit GS, Bates JE, Valente E (1995). Social information-processing patterns partially mediate the effect of early physical abuse on later conduct problems. J Abnorm Psychol.

[112] Thompson SJ, McManus H, Lantry J, Windsor L, Flynn P (2006). Insights from the street: perceptions of services and providers by homeless young adults. Eval Program Plann.

[113] Cummings JR, Wen H, Druss BG (2013). Improving access to mental health services for youth in the United States. JAMA.

[114] Kieling C, Baker-Henningham H, Belfer M, Conti G, Ertem I, Omigbodun O (2011). Child and adolescent mental health worldwide: evidence for action. Lancet.

[115] Solorio MR, Milburn NG, Andersen RM, Trifskin S, Rodríguez MA (2006). Emotional distress and mental health service use among urban homeless adolescents. J Behav Health Serv Res.

[116] Ensign J, Bell M (2004). Illness experiences of homeless youth. Qual Health Res.

[117] Ribeiro WS, Andreoli SB, Ferri CP, Prince M, Mari JJ (2009). Exposure to violence and mental health problems in low and middle-income countries: a literature review. Rev Bras Psiquiatr.

[118] Amone-P'Olak K, Jones PB, Abbott R, Meiser-Stedman R, Ovuga E, Croudace TJ (2013). Cohort profile: mental health following extreme trauma in a northern Ugandan cohort of War-Affected Youth Study (The WAYS Study). SpringerPlus.

[119] Corliss HL, Belzer M, Forbes C, Wilson EC (2008). An evaluation of service utilization among male to female transgender youth: qualitative study of a clinic-based sample. J LGBT Health Res.

[120] Castaneda H (2013). Structural vulnerability and access to medical care among migrant street-based male sex workers in Germany. Soc Sci Med.

[121] Shannon K, Rusch M, Shoveller J, Alexson D, Gibson K, Tyndall MW (2008). Mapping violence and policing as an environmental-structural barrier to health service and syringe availability among substance-using women in street-level sex work. Int J Drug Policy.

[122] Christiani A, Hudson AL, Nyamathi A, Mutere M, Sweat J (2008). Attitudes of homeless and drug-using youth regarding barriers and facilitators in delivery of quality and culturally sensitive health care. J Child Adolesc Psychiatr Nurs.

[123] Fergus S, Zimmerman MA (2005). Adolescent resilience: a framework for understanding healthy development in the face of risk. Annu Rev Public Health.

[124] Resnick MD, Bearman PS, Blum RW, Bauman KE, Harris KM, Jones J (1997). Protecting adolescents from harm: findings from the National Longitudinal Study on Adolescent Health. JAMA.

[125] Borowsky IW, Ireland M, Resnick MD (2002). Violence risk and protective factors among youth held back in school. Ambul Pediatr.

[126] Kidd S, Shahar G (2008). Resilience in homeless youth: the key role of self-esteem. Am J Orthopsychiatry.

[127] Tevendale HD, Lightfoot M, Slocum SL (2009). Individual and environmental protective factors for risky sexual behavior among homeless youth: an exploration of gender differences. AIDS Behav.

[128] Yap MB, Devilly GJ (2004). The role of perceived social support in crime victimization. Clin Psychol Rev.

[129] Eisenberg ME, Resnick MD (2006). Suicidality among gay, lesbian and bisexual youth: the role of protective factors. J Adolesc Health.

[130] Sheets RL, Mohr JJ (2009). Perceived social support from friends and family and psychosocial functioning in bisexual young adult college students. J Couns Psychol.

[131] Doty ND, Willoughby BL, Lindahl KM, Malik NM (2010). Sexuality related social support among lesbian, gay, and bisexual youth. J Youth Adolesc.

[132] Williams T, Connolly J, Pepler D, Craig W (2005). Peer victimization, social support, and psychosocial adjustment of sexual minority adolescents. J Youth Adolesc.

[133] Vincke J, van Heeringen K (2002). Confidant support and the mental wellbeing of lesbian and gay young adults: a longitudinal analysis. J Community Appl Soc Psychol.

[134] Szymanski DM (2009). Examining potential moderators of the link between heterosexist events and gay and bisexual men's psychological distress. J Couns Psychol.

[135] Stice E, Ragan J, Randall P (2004). Prospective relations between social support and depression: differential direction of effects for parent and peer support?. J Abnorm Psychol.

[136] Lewinsohn PM, Roberts RE, Seeley JR, Rohde P, Gotlib IH, Hops H (1994). Adolescent psychopathology: II. Psychosocial risk factors for depression. J Abnorm Psychol.

[137] Needham BL, Austin EL (2010). Sexual orientation, parental support, and health during the transition to young adulthood. J Youth Adolesc.

[138] Simons L, Schrager SM, Clark LF, Belzer M, Olson J (2013). Parental support and mental health among transgender adolescents. J Adolesc Health.

[139] Nesmith AA, Burton DL, Cosgrove TJ (1999). Gay, lesbian, and bisexual youth and young adults: social support in their own words. J Homosex.

[140] Rothman EF, Sullivan M, Keyes S, Boehmer U (2012). Parents’ supportive reactions to sexual orientation disclosure associated with better health: results from a population-based survey of LGB adults in Massachusetts. J Homosex.

[141] Wright ER, Perry BL (2006). Sexual identity distress, social support, and the health of gay, lesbian, and bisexual youth. J Homosex.

[142] McMorris BJ, Tyler KA, Whitbeck LB, Hoyt DR (2002). Familial and on-the-street risk factors associated with alcohol use among homeless and runaway adolescents. J Stud Alcohol Drugs.

[143] Ennett ST, Bailey SL, Federman EB (1999). Social network characteristics associated with risky behaviors among runaway and homeless youth. J Health Soc Behav.

[144] Darwich L, Hymel S, Waterhouse T (2012). School avoidance and substance use among lesbian, gay, bisexual, and questioning youths: the impact of peer victimization and adult support. J Educ Psychol.

[145] Chernoff M, Nachman S, Williams P, Brouwers P, Heston J, Hodge J (2009). Mental health treatment patterns in perinatally HIV-infected youth and controls. Pediatrics.

[146] World Health Organization (2014). mhGAP Intervention Guide for mental, neurological and substance use disorders in non-specialized health settings. http://www.paho.org/mhgap/en/depression.html.

[147] Costello JE, Erkanli A, Angold A (2006). Is there an epidemic of child or adolescent depression?. J Child Psychol Psychiatry.

[148] Goodyer IM, Croudace T, Dudbridge F, Ban M, Herbert J (2010). Polymorphisms in BDNF (Val66Met) and 5-HTTLPR, morning cortisol and subsequent depression in at-risk adolescents. Br J Psychiatry.

[149] Hetrick SE, Purcell R, Garner B, Parslow R (2010). Combined pharmacotherapy and psychological therapies for post traumatic stress disorder (PTSD). Cochrane Database Syst Rev.

[150] Coren E, Hossain R, Pardo Pardo J, Veras MM, Chakraborty K, Harris H (2013). Interventions for promoting reintegration and reducing harmful behaviour and lifestyles in street-connected children and young people. Cochrane Database Syst Rev.

[151] Johnson WD, Diaz RM, Flanders WD, Goodman M, Hill AN, Holtgrave D (2008). Behavioral interventions to reduce risk for sexual transmission of HIV among men who have sex with men. Cochrane Database Syst Rev.

[152] Bower P, Knowles S, Coventry PA, Rowland N (2011). Counselling for mental health and psychosocial problems in primary care. Cochrane Database Syst Rev.

[153] Cleary M, Hunt G, Matheson S, Siegfried N, Walter G (2008). Psychosocial interventions for people with both severe mental illness and substance misuse. Cochrane Database Syst Rev.

[154] McCambridge J, Gates S, Smith LA, Foxcroft DR (2004). Interventions for prevention of drug use by young people delivered in non-school settings. Cochrane Database Syst Rev.

[155] Goldberg E, Millson P, Rivers S, Manning SJ, Leslie K, Read S (2009). A human immunodeficiency virus risk reduction intervention for incarcerated youth: a randomized controlled trial. J Adolesc Health.

[156] St Lawrence JS, Crosby RA, Belcher L, Yazdani N, Brasfield TL (1999). Sexual risk reduction and anger management interventions for incarcerated male adolescents: a randomized controlled trial of two interventions. J Sex Educ Ther.

[157] Stanton BF, Li X, Ricardo I, Galbraith J, Feigelman S, Kaljee L (1996). A randomized, controlled effectiveness trial of an AIDS prevention program for low-income African-American youths. Arch Pediatr Adolesc Med.

[158] Cox GR, Callahan P, Churchill R, Hunot V, Merry SN, Parker AG (2012). Psychological therapies versus antidepressant medication, alone and in combination for depression in children and adolescents. Cochrane Database Syst Rev.

[159] Foxcroft DR, Tsertsvadze A (2012). Cochrane review: Universal school-based prevention programs for alcohol misuse in young people. Evid Based Child Health.

[160] Gates S, McCambridge J, Smith LA, Foxcroft D (2006). Interventions for prevention of drug use by young people delivered in non-school settings. Cochrane Database Syst Rev.

[161] McGovern MP, Lambert-Harris C, Gotham HJ, Claus RE, Xie H (2014). Dual diagnosis capability in mental health and addiction treatment services: an assessment of programs across multiple state systems. Adm Policy Ment Health.

[162] Foster S, LeFauve C, Kresky-Wolff M, Rickards LD (2010). Services and supports for individuals with co-occurring disorders and long-term homelessness. J Behav Health Serv Res.

[163] Drake RE, Essock SM, Shaner A, Carey KB, Minkoff K, Kola L (2001). Implementing dual diagnosis services for clients with severe mental illness. Psychiatr Serv.

[164] Mitchell AJ, Malone D, Doebbeling CC (2009). Quality of medical care for people with and without comorbid mental illness and substance misuse: systematic review of comparative studies. Br J Psychiatry.

[165] Mutumba M, Tomlinson M, Tsai AC (2014). Psychometric properties of instruments for assessing depression among African youth: a systematic review. J Child Adolesc Mental Health.

[166] Tomlinson M, Swartz L, Kruger L-M, Gureje O (2007). Manifestations of affective disturbance in sub-Saharan Africa: key themes. J Affect Disord.

[167] Ventevogel P, Jordans M, Reis R, de Jong J (2013). Madness or sadness? Local concepts of mental illness in four conflict-affected African communities. Confl Health.

[168] Bebbington P (1993). Transcultural aspects of affective disorders. Int Rev Psychiatry.

[169] Caldwell JC, Caldwell P, Caldwell BK, Pieris I (1998). The construction of adolescence in a changing world: implications for sexuality, reproduction, and marriage. Stud Fam Plann.

[170] Fatusi AO, Hindin MJ (2010). Adolescents and youth in developing countries: health and development issues in context. J Adolesc.

[171] United Nations Human Rights (UNHR) (1989). Convention on the rights of the child. http://www.ohchr.org/en/professionalinterest/pages/crc.aspx.

[172] Diemer MA, Li CH (2011). Critical consciousness development and political participation among marginalized youth. Child Dev.

# Sub article: Adolescent girls and young women: key populations for HIV epidemic control

**DOI:** 10.7448/IAS.18.2.19408

**Published:** 2015-02-26

**Authors:** Rachael C Dellar, Sarah Dlamini, Quarraisha Abdool Karim

**Affiliations:** 1 Centre for the AIDS Programme of Research in South Africa (CAPRISA), Nelson R Mandela School of Medicine, University of KwaZulu-Natal, Durban, South Africa; 2 Department of Epidemiology, Mailman School of Public Health, Columbia University, New York, NY, USA

**Keywords:** HIV prevention, adolescent girls, young women, prevention interventions

## Abstract

**Introduction:**

At the epicentre of the HIV epidemic in southern Africa, adolescent girls and young women aged 15–24 contribute a disproportionate ~30% of all new infections and seroconvert 5–7 years earlier than their male peers. This age–sex disparity in HIV acquisition continues to sustain unprecedentedly high incidence rates, and preventing HIV infection in this age group is a pre-requisite for achieving an AIDS-free generation and attaining epidemic control.

**Discussion:**

Adolescent girls and young women in southern Africa are uniquely vulnerable to HIV and have up to eight times more infection than their male peers. While the cause of this vulnerability has not been fully elucidated, it is compounded by structural, social and biological factors. These factors include but are not limited to: engagement in age-disparate and/or transactional relationships, few years of schooling, experience of food insecurity, experience of gender-based violence, increased genital inflammation, and amplification of effects of transmission co-factors. Despite the large and immediate HIV prevention need of adolescent girls and young women, there is a dearth of evidence-based interventions to reduce their risk. The exclusion of adolescents in biomedical research is a huge barrier. School and community-based education programmes are commonplace in many settings, yet few have been evaluated and none have demonstrated efficacy in preventing HIV infection. Promising data are emerging on prophylactic use of anti-retrovirals and conditional cash transfers for HIV prevention in these populations.

**Conclusions:**

There is an urgent need to meet the HIV prevention needs of adolescent girls and young women, particularly those who are unable to negotiate monogamy, condom use and/or male circumcision. Concerted efforts to expand the prevention options available to these young women in terms of the development of novel HIV-specific biomedical, structural and behavioural interventions are urgently needed for epidemic control. In the interim, a pragmatic approach of integrating existing HIV prevention efforts into broader sexual reproductive health services is a public health imperative.

## Introduction

Southern Africa is at the epicentre of the global HIV epidemic, bearing almost 40% of the global burden of infection despite being home to less than 2% of the global population [1]. In this endemic setting, the dominant mode of transmission is through heterosexual sex. UNAIDS has described the epidemic as a generalized and hyper-endemic to reflect the continued unprecedentedly high (>10%) population prevalence [1,2]. However, generalizability should not be equated to uniformity, as significant heterogeneity exists in terms of where and in whom HIV infections occur, with certain localities and populations being consistently more vulnerable to infection than others [1,3]. Focusing HIV prevention efforts on such high-incidence locations and populations is likely to enable the greatest gains to be made in altering current epidemiological trajectories toward control of the HIV epidemic [4].

An important key population in the southern African setting is young women aged 15–24 years, who contribute nearly 30% of all new HIV infections in the region [1,5,6]. In South Africa, this percentage translates to 113,000 new infections in young women per year, more than four-times the number contributed by their male peers (Figure 1) [5]. Such disproportionately high HIV incidence in young women compared to young men is explained by a striking and characteristic feature of the HIV epidemic in this region: the age–sex disparity in HIV acquisition, wherein young women acquire HIV around five to seven years earlier than young men, often synonymously with sexual debut (Figure 2) [5,7].

**Figure 1:** Disproportionate HIV incidence in young women in South Africa. Adapted from Shisana et al. [5].

**Figure 2:** Age–sex disparity in HIV acquisition. Adapted from Shisana et al. [5] and Abdool Karim et al. [8].

As a result of the age–sex disparity in HIV acquisition, HIV prevalence in young women is high, and represents a substantial treatment burden [5,8]; for example, between 2009 and 2013, 27% of women less than 20 years attending antenatal clinics in a rural sub-district of KwaZulu-Natal were found to be HIV positive (unpublished). On a population level, the high incidence in young women is sustaining intergenerational transmission of HIV and contributes to the overall disproportionate burden of HIV in women compared to men [9]. Indeed, approximately 60% of all people living with HIV in sub-Saharan Africa are women [1]. Clearly, achieving the goal of an “AIDS-free generation” depends on reducing the burden of new infection in this key population [10].

However, despite the imperative to prevent HIV acquisition in young women, there remains a paucity of evidence-based interventions available to this population. Indeed, current options are typically limited to promotion of abstinence (or delayed sexual debut), behaviour change, and condom use, all of which are somewhat challenging given the underlying gender-power dynamics of the southern African setting [10]. Further, whilst there has been great optimism following the recent demonstrations of the prevention potential of antiretrovirals (ARVs) – both prophylactically to prevent HIV acquisition (pre-exposure prophylaxis, or PrEP) and for treatment to minimise onward transmission (treatment as prevention, TasP) – to date none of the PrEP trials have included participants <18 years of age, and as such it seems unlikely that these advances will be of benefit to the full range of those considered young women (see Box 1) in the immediate future [10,11].

*Box 1.* Defining young women.

The standard definition of young women includes all those falling within the ages of 15–24 years. As such, most epidemiological data, and much of the discussion here, is presented in terms of this age stratification.

 It is, however, important to note that between these ages, young women undergo significant transitions in lifestyle, maturity, and legal rights which will place them at different vulnerabilities at different time points.

 It is likely that the significance of the <18 years vs. >18 years divide will increase in significance with the rollout of PrEP, as few safety studies for PrEP interventions have been conducted in adolescents <18 years. As such, we would like to encourage the use of this and other sub-strata by those reporting on HIV surveillance in young people.

Moreover, a crucial step in addressing the public health imperative to reduce HIV acquisition in young women is the validation of the safety of existing technologies and interventions for HIV prevention in young women <18 years [10,12]. Concurrently, a concerted effort is required to better understand both the biological and structural factors driving the heightened vulnerability to HIV infection in young women more broadly. Such efforts, in parallel with a consolidation of the evidence obtained from adolescent- and youth-focused HIV prevention interventions and programmes conducted to date, should serve to inform the development of more efficacious interventions.

The objective of this review is to provide an overview of the state-of-the-science of HIV prevention in young women and adolescent girls to inform policy and research direction. Specifically, we aim to (1) summarise the various behavioural and biological factors that predispose adolescent girls and young women to HIV infection, (2) briefly review the evidence from previous HIV prevention interventions targeted toward adolescent girls and young women, and (3) discuss future directions for HIV prevention in adolescent girls and young women.

## Discussion

### Why are adolescent girls and young women so vulnerable to HIV infection?

#### Socio-behavioural associations of HIV infection in adolescent girls and young women

Arguably the most convincing driver of the age–sex disparity in HIV acquisition observed in sub-Saharan Africa is the high prevalence of intergenerational relationships between young women and older men [13,14]. The aggregating prevalence of HIV with increasing age means that, *ceterius paribus*, a young girl engaging in a sexual relationship with an older man is at much higher risk of HIV acquisition compared to a young girl engaging with a male peer (Figure 2) [5]. Further, a young woman engaging in a relationship with an older man may be less likely to negotiate condom use given the gender-power dynamics in the southern African setting, further augmenting her risk [13,15]. Consistent with these data, a number of studies have demonstrated that engagement in an age-disparate or intergenerational relationship is strongly associated with increased HIV prevalence in young women [13,16–18]. Further work is needed to understand how this association may be changing over time with increasing ARV therapy (ART) coverage, and survival of both HIV infected men and women over 25 years of age.

Understanding the complex factors that drive adolescent girls and young women to engage in sexual relationships with older men is challenging, but may be critical in terms of adequately addressing the prevention needs of these key populations. In many cases, young women have reported feeling flattered by the attention of older men, and many relationships are likely to be built on genuine romantic connections [19,20]. In other instances, young women may be motivated primarily by the increased financial or social capital available through engaging in relationships with older men; indeed, many adolescent girls and young women report involvement in these “transactional relationships,” which have significant additional implications for HIV risk [21,22].

Beyond engagement in age-disparate relationships, other risk factors for HIV infection in young women include early sexual debut, few years of schooling, food insecurity, loss of a family member, and experience of gender-based violence [8,17,23–28]. Many of these factors may mediate their effects on HIV acquisition via increasing the relative value of financial capital available through engagement in transactional relationships with older men [21,29–32]. However, independent pathways of risk mediation are also likely to exist. Food insecurity, for example, may also make young women biologically more susceptible to HIV [33].

#### Possible biological mechanisms for heightened vulnerability to HIV infection in adolescent girls and young women

The per-coital act HIV incidence rate in adolescent girls and young women is so high that it seems unlikely that it can be explained by behavioural risk alone [34,35]. Indeed, many young women become infected after just a few coital encounters, and on a population level, acquisition seems almost synonymous with sexual debut [17,36]. As such, there has been significant investigation into potential biological factors that might augment behavioural risk, and a number of mechanisms have been hypothesised to result in heightened vulnerability to infection in young women, compared both to men and to older women.

For example, a number of studies focused on sero-discordant couples have highlighted a higher per-act risk of HIV acquisition in women compared to men [37–40]. A portion of this effect may be attributed to the higher viral load typically observed in men, but the phenomena may also be explained at least in part by physical factors that result in increased exposure to HIV in women, compounded both from the comparatively larger surface area of the cervico-vaginal mucosa and from the increased HIV mucosal exposure time (semen can remain in the female genital tract up to three days post-coitus) [41,42]. The higher per-act risk of HIV acquisition in women could also result from the relatively high levels of activation of the immune cells in the female genital tract, the increased expression of HIV co-receptors in cervical cells compared to foreskin cells, and/or a mucosal surface more likely to acquire micro-abrasions during sex: together, these factors result in more accessible portals for HIV entry in women [35,43–46].

Further, young women are more susceptible to HIV infection compared to older women, and there are a number of biological factors that have been promulgated to explain this age-variability in vulnerability. For example, the immature cervix has a greater proportion of genital mucosa exposed to HIV that is highly susceptible to infection, and young women have relatively high levels of genital inflammation which have consistently been reported to increase HIV acquisition risk [23,35,47–49].

When considering the apparently uniquely high per-act HIV acquisition risk in young women, it is also necessary to consider other relevant contextual factors that may mediate the infection environment, including other sexually transmitted infections (STIs) and contraceptive use. For example, many bacterial and viral STIs are associated with increased risk of HIV infection, and are much more prevalent in young women compared to young men [50,51]. A recent school-based survey conducted in rural KwaZulu-Natal, South Africa, found the trend in herpes simplex virus-2 (HSV-2) acquisition to mirror the age–sex disparity in HIV infection, with young female students acquiring HSV-2 soon after sexual debut, and a more than three-fold higher prevalence of HSV-2 compared to their male peers (Figure 3) [8]. Interestingly, recent HSV-2 infection may confer the greatest impact in terms of increasing vulnerability to HIV, such that the female genital tract in the immediate years following HSV-2 acquisition may be particularly susceptible to HIV infection [52,53].

**Figure 3:** Age–sex disparity in HSV-2 acquisition. Adapted from Abdool Karim et al. [8].

Beyond STIs, other biological risk factors may also be amplified in young women. For example, one study has shown that the use of the hormonal contraceptive depot medroxyprogesterone acetate (DMPA) increases the risk of HIV acquisition in young women (18–24 years), while decreasing HIV acquisition risk in older women (≥25 years) [54]. Further, although establishing causal relationships is challenging, intra-vaginal cleaning practices are more prevalent in younger women, suggesting these women are consequently more likely to have an altered vaginal flora, potentially heightening their HIV susceptibility [55,56].

Together these biological factors may create a “perfect storm” of conditions in recently sexually debuted adolescent girls and young women in southern Africa making them uniquely vulnerable to HIV infection when exposed to the virus via engaging in unprotected sex with an HIV-positive partner.

### Effectiveness of current HIV prevention interventions available to adolescent girls and young women

#### In-school interventions

Schools provide convenient venues for HIV prevention education, and not surprisingly a vast number of youth-targeted HIV, STI, and pregnancy prevention programmes operate in schools throughout sub-Saharan Africa [57]. The effectiveness of such programmes in young people in sub-Saharan Africa has been the subject of a considerable number of systematic reviews [58–71]. To summarise the evidence, several programmes have been demonstrated to be effective in improving knowledge and attitudes concerning HIV and the uptake of HIV testing. These data follow a general trend in sub-Saharan Africa of increasing comprehension and understanding about HIV in young people [1]. Those interventions demonstrating the most success are characterised by a number of factors, including but not limited to: iterative and context-specific session programmes, HIV prevention and sexual and reproductive health (SRH) curricula that include tasks focused toward more general skills and knowledge development, and delivery by trained facilitators [57]. In contrast, abstinence-only and peer-led in-school interventions tend to be ineffective [57,62].

Despite some apparent successes, few rigorously conducted trials have assessed the impact of interventions on biological outcomes, including HIV, STI and/or pregnancy incidence. Those trials that have demonstrated no significant effects of any school-based intervention on these biological outcomes, in spite of reporting positive impacts on self-reported behaviour change in adolescents [72–74]. These results may stem from the relatively strong prevention effect of being in school itself, which may dwarf the effect of any behavioural intervention. However, the burden of HIV in school-attending adolescents, while lower than out-of-school adolescents, remains significant, and thus there is also concern that the results might point to differential desirability bias by trial arm, which questions the validity of significant changes in self-reported markers of behaviour change reported by other studies. The data from school-based trials also underscore that while knowledge is a pre-requisite for HIV prevention, it is in itself insufficient to prevent HIV infection.

#### Attempts to make health services youth-friendly

Other interventions to prevent HIV infection in young people have focused on health systems strengthening in an effort to address barriers to healthcare access by increasing the provision of high-quality, youth-friendly HIV and SRH services. Such interventions are potentially critical, as there is significant demand for more comprehensive SRH services that recognise the inter-relationships between HIV and broader SRH and thus the importance of integrated service delivery [1,57].

Interventions to make health services more youth-friendly have typically focused on a different combinations of training of service providers, outreach activities, and provision of mobile services targeted toward specific high-risk adolescent populations [66,75–77]. Many of these interventions have been successful in terms of increasing uptake of services by young people. However, similarly to in-school interventions, there is a notable dearth of biological-outcome-based assessment.

#### Community-level interventions

HIV prevention interventions implemented at the community level are highly heterogeneous, including sporting events, mentoring and youth centres [78]. Evaluation of these interventions highlights their largely positive impact on knowledge and attitudes to HIV. However, these interventions often fail to reach the most HIV vulnerable populations, and evaluation designs are generally weak. Only one study to our knowledge has assessed HIV incidence, and this study reported no evidence of effectiveness [79].

#### Conditional cash transfers

Cash transfers to young people that incentivise safer behaviour have recently emerged as a new strategy to reduce young people's vulnerability to HIV [1,80]. The evidence in support of the efficacy of this strategy is limited but promising. Indeed, a recent randomized controlled trial in Lesotho demonstrated that a programme of financial incentives reduced the probability of acquiring HIV by 25% over two years [81]. Similarly, an independent randomized controlled trial in Malawi reported that those female high school students who received conditional cash transfers (CCTs) were 64% less likely to be HIV infected compared to those who were not [82]; however, baseline HIV infection was not measured. These data suggest a potential for CCT to prevent HIV in young people, and outcomes of current research in the field such as HPTN 068 are eagerly awaited.

### Gaps and future directions

Despite the large and immediate need for HIV prevention in adolescent girls and young women, there is a dearth of evidence-based interventions available to them to reduce their risk. Given the diversity of epidemics within and between countries, in order to develop more efficacious youth-focused prevention interventions, a sound understanding of the local epidemic is required as well as the bio-behavioural nexus that renders adolescent girls and young women more vulnerable to HIV infection. The significant SRH needs of young women should be central to the design of new interventions, as integration of services is the backbone of a pragmatic approach to address needs now, even as we refine, develop and test new and novel approaches [1,83]. A careful review of previous interventions and their evaluations is needed to ensure maximum gains. Most notably, it is critical that any future intervention should be rigorously assessed for effectiveness in controlled trials with biological outcomes prior to wide-scale implementation to maximise efficiency and effectiveness of resource allocation. Many researchers would benefit from engaging the young women themselves as partners in intervention design and implementation, and certainly encouraging male partner buy-in and female empowerment will also be important in those settings where gender-power dynamics augment HIV risk.

A further important direction for future research should be to develop interventions targeted to hard-to-reach young people who might be missed by school- or community-based interventions. The evidence for the best practice in reaching such populations is particularly limited, despite their often greater risk of HIV acquisition. However, our own experiences highlight that some important components of making service provision palatable and attractive to hard-to-reach adolescents include anonymized testing, flexible clinic hours and adaptions of respondent-driven sampling. Concurrently, efforts should be made to keep adolescents in school. The task of developing and evaluating new HIV prevention interventions – particularly those programmes that aim to address the underlying social vulnerabilities – is substantial, and will potentially require decades of concentrated action, during which time adolescent girls and young women will continue to become infected in their hundreds of thousands. As such, it is a moral imperative to effectively deliver what we know works now. The most pressing example of a technology that we know works but is not being delivered is PrEP, which was developed specifically with young women in southern Africa in mind: designed to allow them to exercise their rights over their health and take control over their own risk without dependence on their sexual partners. While the number of randomized controlled trials demonstrating the effectiveness of PrEP continues to grow, this success has yet to be translated into product availability in southern Africa. Undeniably, PrEP is not 100% effective, is limited by adherence and would benefit from improvements currently in development; however, one has to question where the threshold of evidence required for rollout of current forms of PrEP to young women in southern Africa lies. A simple calculation highlights that even with a 39% efficiency, rollout of Tenofovir gel to young women aged 15–24 years in South Africa alone might prevent more than 44,000 infections in one year. Implementation and policy science are urgently needed to translate research on PrEP effectiveness into averted infections. Further, there is also work to be done in ensuring that on rollout, the state-of-the-science of prevention is not lagging behind in adolescents <18 years because of restrictive ethico-legal guidelines that often prevent them from participating in biomedical research in spite of their substantial need [6,10].

This review was restricted to considering HIV prevention in adolescent girls and young women. However, the treatment needs resulting from the unprecedentedly high HIV incidence rates in these key populations should not be underestimated: in Lesotho for example, almost a quarter of all young people aged 15–24 years are infected with HIV [1]. Adolescent-focused HIV prevention interventions should also seek to meet the needs of HIV-positive young people who face significant barriers to care. Indeed, of note is that adolescents (10–19 years) are the only age group in which AIDS deaths have risen between 2001 and 2012 [1].

## Conclusions

Meeting the HIV prevention and SRH needs of adolescent girls and young women who are at uniquely high risk of HIV acquisition is a public health and moral imperative and a requirement to meet the laudable goals of achieving an AIDS-free generation and/or epidemic control. However, despite this imperative, evidence-based prevention options available to adolescent girls and young women remain limited, and even as efforts get underway to develop more efficacious interventions, they are likely to take many years to reach fruition. Immediate action is therefore needed to facilitate this key population to mediate their own risk, including as first steps rollout of PrEP, adolescent enrolment in biomedical HIV prevention trials, and provision of accessible and integrated SRH-HIV prevention services.

## Author information

RD is a research fellow at CAPRISA supported by the CAPRISA Training Programme.

## References

[1] UNAIDS Global report: UNAIDS report on the global AIDS epidemic 2013 [cited 2014 Aug 14]. http://www.unaids.org/en/media/unaids/contentassets/documents/epidemiology/2013/gr2013/UNAIDS_Global_Report_2013_en.pdf.

[2] UNAIDS 2008 Report on the global AIDS epidemic [cited 2014 Aug 14]. http://www.unaids.org/en/media/unaids/contentassets/dataimport/pub/globalreport/2008/jc1510_2008globalreport_en.pdf.

[3] Anderson SJ, Cherutich P, Kilonzo N, Cremin I, Fecht D, Kimanga D (2014). Maximising the effect of combination HIV prevention through prioritisation of the people and places in greatest need: a modelling study. Lancet.

[4] Barnabas RV, Celum C (2014). Bending the curve: maximising impact with focused HIV prevention. Lancet.

[5] Shisana O, Rehle T, Simbayi LC, Zuma K, Jooste S, Zungu N (2014). South African national HIV prevalence, incidence and behaviour survey, 2012.

[6] Cowan F, Pettifor A (2009). HIV in adolescents in sub-Saharan Africa. Curr Opin HIV AIDS.

[7] Abdool Karim Q, Abdool Karim SS, Singh B, Short R, Ngxongo S (1992). Seroprevalence of HIV infection in rural South Africa. AIDS.

[8] Abdool Karim Q, Kharsany AB, Leask K, Ntombela F, Humphries H, Frohlich JA (2014). Prevalence of HIV, HSV-2 and pregnancy among high school students in rural KwaZulu-Natal, South Africa: a bio-behavioural cross-sectional survey. Sex Transm Infect.

[9] Abdool Karim Q, Abdool Karim SS HIV/AIDS in South Africa.

[10] Abdool Karim Q, Dellar R (2014). Inclusion of adolescent girls in HIV prevention research – an imperative for an AIDS-free generation. J Int AIDS Soc.

[11] Fauci AS, Folkers GK, Dieffenbach CW (2013). HIV-AIDS: much accomplished, much to do. Nat Immunol.

[12] Pettifor A, Bekker LG (2012). Adolescent enrolment in HIV prevention trials. Lancet.

[13] Gregson S, Nyamukapa CA, Garnett GP, Mason PR, Zhuwau T, Carael M (2002). Sexual mixing patterns and sex-differentials in teenage exposure to HIV infection in rural Zimbabwe. Lancet.

[14] Pettifor A, Macphail C, Rees H, Cohen M (2008). HIV and sexual behavior among young people: the South African paradox. Sex Transm Dis.

[15] Pettifor AE, Measham DM, Rees HV, Padian NS (2004). Sexual power and HIV risk, South Africa. Emerg Infect Dis.

[16] Kelly RJ, Gray RH, Sewankambo NK, Serwadda D, Wabwire-Mangen F, Lutalo T (2003). Age differences in sexual partners and risk of HIV-1 infection in rural Uganda. J Acquir Immune Defic Syndr.

[17] Pettifor AE, Rees HV, Kleinschmidt I, Steffenson AE, MacPhail C, Hlongwa-Madikizela L (2005). Young people's sexual health in South Africa: HIV prevalence and sexual behaviors from a nationally representative household survey. AIDS.

[18] Kaiser R, Bunnell R, Hightower A, Kim AA, Cherutich P, Mwangi M (2011). Factors associated with HIV infection in married or cohabitating couples in Kenya: results from a nationally representative study. PLoS One.

[19] Wamoyi J, Wight D, Plummer M, Mshana GH, Ross D (2010). Transactional sex amongst young people in rural northern Tanzania: an ethnography of young women's motivations and negotiation. Reprod Health.

[20] Stoebenau K, Nixon SA, Rubincam C, Willan S, Zembe YZ, Tsikoane T (2011). More than just talk: the framing of transactional sex and its implications for vulnerability to HIV in Lesotho, Madagascar and South Africa. Global Health.

[21] Dunkle KL, Jewkes RK, Brown HC, Gray GE, McIntryre JA, Harlow SD (2004). Transactional sex among women in Soweto, South Africa: prevalence, risk factors and association with HIV infection. Soc Sci Med.

[22] MacPherson EE, Sadalaki J, Njoloma M, Nyongopa V, Nkhwazi L, Mwapasa V (2012). Transactional sex and HIV: understanding the gendered structural drivers of HIV in fishing communities in Southern Malawi. J Int AIDS Soc.

[23] Dixon-Mueller R (2008). How young is “too young”? Comparative perspectives on adolescent sexual, marital, and reproductive transitions. Stud Fam Plann.

[24] Hallman K (2005). Gendered socioeconomic conditions and HIV risk behaviours among young people in South Africa. Afr J AIDS Res.

[25] Hallman K (2006). HIV vulnerability of non-enrolled and urban poor girls in KwaZulu-Natal, South Africa.

[26] Joint United Nations Programme on HIV/AIDS (UNAIDS), Interagency Task Team on HIV and Young People (2008). Guidance brief: HIV interventions for most at-risk young people.

[27] Pettifor AE, van der Straten A, Dunbar MS, Shiboski SC, Padian NS (2004). Early age of first sex: a risk factor for HIV infection among women in Zimbabwe. AIDS.

[28] Pettifor AE, Levandowski BA, MacPhail C, Padian NS, Cohen MS, Rees HV (2008). Keep them in school: the importance of education as a protective factor against HIV infection among young South African women. Int J Epidemiol.

[29] Greif MJ (2012). Housing, medical, and food deprivation in poor urban contexts: implications for multiple sexual partnerships and transactional sex in Nairobi's slums. Health Place.

[30] Chatterji M, Murray N, London D, Anglewicz P (2005). The factors influencing transactional sex among young men and women in 12 sub-Saharan African countries. Soc Biol.

[31] Shefer T, Clowes L, Vergnani T (2012). Narratives of transactional sex on a university campus. Cult Health Sex.

[32] Weiser SD, Leiter K, Bangsberg DR, Butler LM, Percy-de Korte F, Hlanze Z (2007). Food insufficiency is associated with high-risk sexual behavior among women in Botswana and Swaziland. PLoS Med.

[33] Weiser SD, Young SL, Cohen CR, Kushel MB, Tsai AC, Tien PC (2011). Conceptual framework for understanding the bidirectional links between food insecurity and HIV/AIDS. Am J Clin Nutr.

[34] Pettifor AE, Hudgens MG, Levandowski BA, Rees HV, Cohen MS (2007). Highly efficient HIV transmission to young women in South Africa. AIDS.

[35] Yi TJ, Shannon B, Prodger J, McKinnon L, Kaul R (2013). Genital immunology and HIV susceptibility in young women. Am J Reprod Immunol.

[36] Glynn JR, Carael M, Auvert B, Kahindo M, Chege J, Musonda R (2001). Why do young women have a much higher prevalence of HIV than young men? A study in Kisumu, Kenya and Ndola, Zambia. AIDS.

[37] Carpenter LM, Kamali A, Ruberantwari A, Malamba SS, Whitworth JA (1999). Rates of HIV-1 transmission within marriage in rural Uganda in relation to the HIV sero-status of the partners. AIDS.

[38] Hira SK, Nkowane BM, Kamanga J, Wadhawan D, Kavindele D, Macuacua R (1990). Epidemiology of human immunodeficiency virus in families in Lusaka, Zambia. J Acquir Immune Defic Syndr.

[39] Padian NS, Shiboski SC, Glass SO, Vittinghoff E (1997). Heterosexual transmission of human immunodeficiency virus (HIV) in northern California: results from a ten-year study. Am J Epidemiol.

[40] Senkoro KP, Boerma JT, Klokke AH, Ng'weshemi JZ, Muro AS, Gabone R (2000). HIV incidence and HIV-associated mortality in a cohort of factory workers and their spouses in Tanzania, 1991 through 1996. J Acquir Immune Defic Syndr.

[41] Kigozi G, Wawer M, Ssettuba A, Kagaayi J, Nalugoda F, Watya S (2009). Foreskin surface area and HIV acquisition in Rakai, Uganda (size matters). AIDS.

[42] Jain R, Muralidhar S (2011). Contraceptive methods: needs, options and utilization. J Obstet Gynaecol India.

[43] Fish EN (2008). The X-files in immunity: sex-based differences predispose immune responses. Nat Rev Immunol.

[44] Prodger JL, Gray R, Kigozi G, Nalugoda F, Galiwango R, Hirbod T (2012). Foreskin T-cell subsets differ substantially from blood with respect to HIV co-receptor expression, inflammatory profile, and memory status. Mucosal Immunol.

[45] McKinnon LR, Nyanga B, Chege D, Izulla P, Kimani M, Huibner S (2011). Characterization of a human cervical CD4+ T cell subset coexpressing multiple markers of HIV susceptibility. J Immunol.

[46] Stanley M (2009). Early age of sexual debut: a risky experience. J Fam Plann Reprod Health Care.

[47] Hwang LY, Scott ME, Ma Y, Moscicki AB (2011). Higher levels of cervicovaginal inflammatory and regulatory cytokines and chemokines in healthy young women with immature cervical epithelium. J Reprod Immunol.

[48] Ghanem KG, Shah N, Klein RS, Mayer KH, Sobel JD, Warren DL (2005). Influence of sex hormones, HIV status, and concomitant sexually transmitted infection on cervicovaginal inflammation. J Infect Dis.

[49] Levinson P, Kaul R, Kimani J, Ngugi E, Moses S, MacDonald KS (2009). Levels of innate immune factors in genital fluids: association of alpha defensins and LL-37 with genital infections and increased HIV acquisition. AIDS.

[50] Rottingen JA, Cameron DW, Garnett GP (2001). A systematic review of the epidemiologic interactions between classic sexually transmitted diseases and HIV: how much really is known?. Sex Transm Dis.

[51] Cohen MS (2004). HIV and sexually transmitted diseases: lethal synergy. Top HIV Med.

[52] Nagot N, Ouedraogo A, Foulongne V, Konate I, Weiss HA, Vergne L (2007). Reduction of HIV-1 RNA levels with therapy to suppress herpes simplex virus. N Engl J Med.

[53] Wald A, Link K (2002). Risk of human immunodeficiency virus infection in herpes simplex virus type 2-seropositive persons: a meta-analysis. J Infect Dis.

[54] Morrison CS, Turner AN, Jones LB (2009). Highly effective contraception and acquisition of HIV and other sexually transmitted infections. Best Pract Res Clin Obstet Gynaecol.

[55] Low N, Chersich MF, Schmidlin K, Egger M, Francis SC, van de Wijgert JH (2011). Intravaginal practices, bacterial vaginosis, and HIV infection in women: individual participant data meta-analysis. PLoS Med.

[56] McClelland RS, Lavreys L, Hassan WM, Mandaliya K, Ndinya-Achola JO, Baeten JM (2006). Vaginal washing and increased risk of HIV-1 acquisition among African women: a 10-year prospective study. AIDS.

[57] Mavedzenge SN, Luecke E, Ross DA (2013). Effectiveness of HIV prevention, treatment and care interventions among adolescents: a systematic review of systematic reviews. UNICEF technical brief.

[58] Yankah E, Aggleton P (2008). Effects and effectiveness of life skills education for HIV prevention in young people. AIDS Educ Prev.

[59] Michielsen K, Chersich MF, Luchters S, De Koker P, Van Rossem R, Temmerman M (2010). Effectiveness of HIV prevention for youth in sub-Saharan Africa: systematic review and meta-analysis of randomized and nonrandomized trials. AIDS.

[60] Harrison A, Newell ML, Imrie J, Hoddinott G (2010). HIV prevention for South African youth: which interventions work? A systematic review of current evidence. BMC Public Health.

[61] Agbemenu K, Schlenk EA (2011). An integrative review of comprehensive sex education for adolescent girls in Kenya. J Nurs Scholarsh.

[62] Napierala Mavedzenge SM, Doyle AM, Ross DA (2011). HIV prevention in young people in sub-Saharan Africa: a systematic review. J Adolesc Health.

[63] Chin HB, Sipe TA, Elder R, Mercer SL, Chattopadhyay SK, Jacob V (2012). The effectiveness of group-based comprehensive risk-reduction and abstinence education interventions to prevent or reduce the risk of adolescent pregnancy, human immunodeficiency virus, and sexually transmitted infections: two systematic reviews for the Guide to Community Preventive Services. Am J Prev Med.

[64] Paul-Ebhohimhen VA, Poobalan A, van Teijlingen ER (2008). A systematic review of school-based sexual health interventions to prevent STI/HIV in sub-Saharan Africa. BMC Public Health.

[65] Robin L, Dittus P, Whitaker D, Crosby R, Ethier K, Mezoff J (2004). Behavioral interventions to reduce incidence of HIV, STD, and pregnancy among adolescents: a decade in review. J Adolesc Health.

[66] Sales JM, Milhausen RR, Diclemente RJ (2006). A decade in review: building on the experiences of past adolescent STI/HIV interventions to optimise future prevention efforts. Sex Transm Infect.

[67] Gallant M, Maticka-Tyndale E (2004). School-based HIV prevention programmes for African youth. Soc Sci Med.

[68] Morrison-Beedy D, Nelson LE (2004). HIV prevention interventions in adolescent girls: what is the state of the science?. Worldviews Evid Based Nurs.

[69] Kirby DB, Laris BA, Rolleri LA (2007). Sex and HIV education programs: their impact on sexual behaviors of young people throughout the world. J Adolesc Health.

[70] Speizer IS, Magnani RJ, Colvin CE (2003). The effectiveness of adolescent reproductive health interventions in developing countries: a review of the evidence. J Adolesc Health.

[71] DiCenso A, Guyatt G, Willan A, Griffith L (2002). Interventions to reduce unintended pregnancies among adolescents: systematic review of randomised controlled trials. BMJ.

[72] Doyle AM, Ross DA, Maganja K, Baisley K, Masesa C, Andreasen A (2010). Long-term biological and behavioural impact of an adolescent sexual health intervention in Tanzania: follow-up survey of the community-based MEMA kwa Vijana Trial. PLoS Med.

[73] Ross DA, Changalucha J, Obasi AI, Todd J, Plummer ML, Cleophas-Mazige B (2007). Biological and behavioural impact of an adolescent sexual health intervention in Tanzania: a community-randomized trial. AIDS.

[74] Cowan FM, Pascoe SJ, Langhaug LF, Mavhu W, Chidiya S, Jaffar S (2010). The Regai Dzive Shiri project: results of a randomized trial of an HIV prevention intervention for youth. AIDS.

[75] Dick B, Ferguson J, Chandra-Mouli V, Brabin L, Chatterjee S, Ross DA (2006). Review of the evidence for interventions to increase young people's use of health services in developing countries. World Health Organ Tech Rep Ser.

[76] Denno DM, Chandra-Mouli V, Osman M (2012). Reaching youth with out-of-facility HIV and reproductive health services: a systematic review. J Adolesc Health.

[77] Zuurmond MA, Geary RS, Ross DA (2012). The effectiveness of youth centers in increasing use of sexual and reproductive health services: a systematic review. Stud Fam Plann.

[78] Maticka-Tyndale E, Brouillard-Coylea C (2006). The effectiveness of community interventions targeting HIV and AIDS prevention at young people in developing countries. World Health Organ Tech Rep Ser.

[79] Jewkes R, Nduna M, Levin J, Jama N, Dunkle K, Puren A (2008). Impact of stepping stones on incidence of HIV and HSV-2 and sexual behaviour in rural South Africa: cluster randomised controlled trial. BMJ.

[80] Pettifor A, MacPhail C, Nguyen N, Rosenberg M (2012). Can money prevent the spread of HIV? A review of cash payments for HIV prevention. AIDS Behav.

[81] Björkman-Nyqvist M, Corno L, de Walque D, Svensson J (2013). Evaluating the impact of short term financial incentives on HIV and STI incidence among youth in Lesotho: a randomized trial.

[82] Baird SJ, Garfein RS, McIntosh CT, Ozler B (2012). Effect of a cash transfer programme for schooling on prevalence of HIV and herpes simplex type 2 in Malawi: a cluster randomised trial. Lancet.

[83] Pettifor A, Bekker LG, Hosek S, DiClemente R, Rosenberg M, Bull SS (2013). Preventing HIV among young people: research priorities for the future. J Acquir Immune Defic Syndr.

# Sub article: “We don't need services. We have no problems”: exploring the experiences of young people who inject drugs in accessing harm reduction services

**DOI:** 10.7448/IAS.18.2.19442

**Published:** 2015-02-26

**Authors:** Anita Krug, Mikaela Hildebrand, Nina Sun

**Affiliations:** 1 Youth RISE, London, UK; 2 UNAIDS, Geneva, Switzerland

**Keywords:** young people, adolescents, drugs, injecting drug use, harm reduction, HIV

## Abstract

**Introduction:**

Evidence suggests that people who inject drugs often begin their drug use and injecting practices in adolescence, yet there are limited data available on the HIV epidemic and the responses for this population. The comprehensive package of interventions for the prevention, treatment and care of HIV infection among people who inject drugs first laid out in 2009 (revised in 2012) by World Health Organization, United Nations Office of Drugs and Crime and Joint United Nations Programme on HIV/AIDS, does not consider the unique needs of adolescent and young people. In order to better understand the values and preferences of young people who inject drugs in accessing harm reduction services and support, we undertook a series of community consultations with young people with experience of injecting drugs during adolescence.

**Methods:**

Community consultations (4–14 persons) were held in 14 countries. Participants were recruited using a combined criterion and maximum variation sampling strategy. Data were analyzed using collaborative qualitative data analysis. Frequency analysis of themes was conducted.

**Results:**

Nineteen community consultations were organized with a total of 132 participants. All participants had experienced injecting drugs before the age of 18. They had the following age distribution: 18–20 (37%), 21–25 (48%) and 26–30 (15%). Of the participants, 73.5% were male while 25.7% were female, with one transgender participant. Barriers to accessing the comprehensive package included: lack of information and knowledge of services, age restrictions on services, belief that services were not needed, fear of law enforcement, fear of stigma, lack of concern, high cost, lack of outreach, lack of knowledge of HCV/TB and lack of youth friendly services.

**Conclusions:**

The consultations provide a rare insight into the lived experiences of adolescents who inject drugs and highlight the dissonance between their reality and current policy and programmatic approaches. Findings suggest that harm reduction and HIV policies and programmes should adapt the comprehensive package to reach young people and explore linkages to other sectors such as education and employment to ensure they are fully supported and protected. Continued participation of the community of young people who inject drugs can help ensure policy and programmes respond to the social exclusion and denial of rights and prevent HIV infection among adolescents who inject drugs.

## Introduction

While the age distribution of the 12.7 million people who inject drugs globally is unknown [1], evidence suggests that people who inject drugs begin their injecting practices at a young age, often in adolescence [2]. Of the total population of people who inject drugs, 13.1%, or 1.7 million, were living with HIV in 2013 [1]. Globally, young people aged 15–24 years account for an estimated 35% of all new infections in people over 15 years of age [3]; yet data on the epidemic and response among young people who inject drugs (YPWID) are limited.

However, the data that do exist paints a stark picture. A number of countries have reported increases in prevalence of injecting drug use among young people [4] and high rates of HIV amongst adolescents who inject drugs [5,6]. YPWID are especially vulnerable to HIV [7,8]. Young people are more likely to share non-sterile injecting equipment [9]. As young people are new to the injecting community, they are less likely to know safer injecting practices [10]. In addition, sexual risk-taking takes place amongst YPWID [11] further increasing HIV risk. Legal age restrictions on harm reduction services prevents young people from accessing these services [12] and punitive measures that criminalize drug use further discourage service use thereby increasing HIV risk [13,14].

The comprehensive package of harm reduction services [15] has been endorsed by the World Health Organization (WHO), United Nations Office of Drugs and Crime (UNODC) and the Joint United Nations Programme on HIV/AIDS (UNAIDS) and are critical for reducing drug-related harms amongst people who inject drugs. The comprehensive package includes: 1) needle and syringe programmes (NSPs); 2) opiate substitution therapy (OSTs); 3) HIV testing and counselling; 4) antiretroviral therapy; 5) prevention of sexually transmitted infections; 6) condom programmes for people who inject drugs and their sexual partners; 7) targeted information, education and communication for people who inject drugs and their sexual partners; 8) vaccination, diagnosis and treatment of viral hepatitis; and 9) prevention, diagnosis and treatment of tuberculosis. However, current guidelines do not consider the unique needs of adolescent and young people or expand on how they could be adapted to ensure this age group is reached with services.

Formative research and/or meaningful community engagement that explore experiences of the “target population” can help develop programmes and policies that are effective [16]. Yet the participation of YPWID in policy and programme development cycles is largely absent [13]. To inform the current effort of the UNAIDS Inter-Agency Working Group on Key Populations (IAWGKP) to develop technical briefs on YPWID, Youth RISE with support from UNAIDS, undertook community consultations with young people who have experience injecting drugs during adolescence (10–19). This report presents the findings and discusses the implications for the comprehensive package of harm reduction services.

## Methods

### Community consultations

Given the dearth of data related to adolescents and YPWID, community consultations were used to generate in-depth information for the technical brief. A community consultation “is designed to recognize and accommodate the relevant particularities of a given community for a specific project” [17].

Consultations were organized in 14 countries, selected for convenience while ensuring geographical/regional diversity. Consultations were organized in different settings. In some countries, two smaller groups were held on the street. In Nepal and Nigeria, separate male and female consultations were held. As a result, a total of 19 consultations were organized: Indonesia (2), Kenya, Kyrgyzstan (2), Lebanon, Mauritius, Mexico, Nepal (2), Nigeria (2), Portugal, Romania, Slovenia, Ukraine, United States (2) and Vietnam. All consultations were conducted between August 2013 and January 2014.

The policy environment for OST and NSPs in the 14 countries was mapped and crosschecked with the 2012 Global State of Harm Reduction report [18]. Age of consent laws were mapped through review of literature [19–21].

A standardized consultation toolkit (a semi-structured discussion guide, a facilitator's guide, ethics protocol, informed consent and demographic information form) was developed by Youth RISE and UNAIDS, together with the community consultation facilitators. The kit was sent out for wider review by experts within the harm reduction field. Questions focused on experiences in accessing the comprehensive package of harm reduction services and how to improve access.

The consultations were facilitated by local young Youth RISE members. Facilitators were selected based on their experience with YPWID. All took part in a project and methodology workshop.

### Participants

Participants were recruited using a combined criterion and maximum variation sampling strategy [22]; that is, the facilitators purposefully recruited diverse participants that met a set of inclusion criteria. The initial criteria were: 1) have experienced injecting under the age of 18, and 2) aged between 18 and 25 years. Participants under the age of 18 were excluded due to ethical considerations. Consultation facilitators identified youth who met the inclusion criteria through services and/or street recruitment. Younger participants were hesitant to take part; consequently the age range was extended to 30 years of age to enable recruitment. While participants included both current and former injectors (regular and less regular), all participants met the inclusion criteria “having experienced injecting under the age of 18.” The discussions explored the experiences of adolescents, but as all participants were over 18 this report refers to young people.

### Data collection and analysis

The consultations were audio recorded, transcribed and translated into English where necessary. Data were analyzed using collaborative qualitative analysis [23]. Facilitators completed a standardized reporting template and the project coordinator independently coded the data [23]. The project coordinator and facilitator analysis were compared, and a preliminary report reviewed by all facilitators to verify data interpretation and findings. Once validated, frequency analysis of themes was conducted [23]. There were a total of 19 complete transcripts. If a theme was present in a majority of the transcripts (10+), it was considered a “strong” theme. If a theme was identified in a third (6–9), it was considered moderate. Unique themes were identified three or less of the transcripts, but offered a unique perspective in relation to the comprehensive package of interventions.

### Ethical protocol

IRB approval for the consultations was not obtained; however, an ethical protocol was followed, including: informed consent and confidentiality to protect identity. UNAIDS in-country offices advised on steps needed to ensure safety of participants, including engagement with Governments where necessary.

## Results

The results of the policy mapping on age restrictions are presented in Table 1 and indicate at what age adolescents can access these services without parental consent in the 14 countries. At the time of consultations, NSPs and OST are available in all countries except for Nigeria and Kenya [18]. Whilst the remaining interventions in the comprehensive package are available in all countries, the coverage of these programmes varies significantly and may not be specifically targeted towards people who inject drugs.

**Table 1:** Age restrictions on harm reduction services and HIV testing in countries where consultations took place

Country	City/location	Age restrictions (NSP)	Age restrictions (OST)	Age restrictions (HIV testing)
Indonesia	Bandung; Medan	None	Yes (18)	None
Kenya	Nairobi	None	No services	None
Kyrgyzstan	Bishkek	None	None	Yes (16)
Lebanon	Beirut	None	Yes (18)	No information identified
Mauritius	Port Louis	None	Yes (18)	None
Mexico	Hermosillo	Yes (18)	Yes (18)	Yes (18)
Nepal	Kathmandu; Pokhara	None	Yes (18)	Yes (14)
Nigeria	Abuja	No services	No services	Yes (18)
Portugal	Porto	None	Yes (18)	Yes (16)
Romania	Bucharest	Yes (18)	Yes (18)	No information identified
Slovenia	Ljubljana	None	16 (methadone); 15 (Subutex)	Yes (15)
Ukraine	Rivne	Yes (14)	Yes (14)	Yes (14)
United States	San Francisco	Varies by state (none in California)	Yes (18)	No information identified
Vietnam	Hanoi	Yes (16)	Yes (18)	Yes (16)

### Consultations

There were 132 participants in the consultations: age: 18–20 (*n*=49 (37%)), 21–25 (*n*=63 (48%)), 26–30 (*n*=20 (15%)); male: (*n*=97 (73.5%)); female: (*n*=34 (25.7%)), and genderqueer: (*n*=1 (0.8%)).

### Age of initiation into injecting drugs and reasons for initiation of injecting

Participants reported initiation into injecting as commonly starting between ages 15 and 18, although age of initiation was reported as young as nine. A process of progressing from cannabis, snorting drugs to finally injecting was commonly described. In all consultations, the need to get a more intense high was reported as the primary reason why injecting was initiated.

Curiosity was reported in a majority of the consultations as influencing adolescents’ decision to inject. Reduced quality and potency of drugs, as well as economic efficiency, were also reported as important reasons. Another was peer-influence:

For me at the beginning I rejected injecting drugs because I saw the blood inside the syringes and I know that it is dangerous and it's also dirty, but after several times saying no, I started to use the needle because I am just curious. I start to inject after about three times my friend offered for me to inject. (young man, Indonesia)

In the US and Indonesia, participants cited rejection from society or family as a point where they turned to harder drugs and/or injecting. “The reason I started injecting was because I was angry. I was expelled from school and abandoned by my own family when they found out I was taking some drugs. So I thought why not go all the way” (young man, Indonesia).

The gratification experienced from injecting drugs played an important role in why a young person continues to inject after their initial experience.

### Experiences accessing the comprehensive package of harm reduction

Participants identified barriers to accessing the interventions contained in the comprehensive package of harm reduction. Table 2 summarizes the frequency analysis of themes in relation to barriers identified.

**Table 2:** Adolescent and young people's identified barriers to accessing the comprehensive package of harm reduction services

Intervention and identified barriers	Theme strength
**Needle and syringe programmes**	
Lack of knowledge of services	Strong
Belief that services are not needed	Strong
Fear of police	Moderate
Fear of exposure of drug use	Moderate
Limited hours of operation	Moderate
Lack of youth-friendly services	Moderate
Age restrictions/parental consent requirements	Unique
Requirement of identity card	Unique
One-for-one exchange policies	Unique
**Opiate substitution therapy**	
Age restrictions/parental consent requirements	Strong
Belief that services are not needed	Strong
Lack of knowledge of service	Moderate
Cost	Moderate
Negative perception of OST and its side effects	Moderate
Registration of people who use drugs and lack of confidentiality	Unique
**HIV testing and counseling**	
Lack of concern	Strong
Cost	Strong
Lack of knowledge of services	Moderate
Stigma and fear of result	Moderate
Un-friendly staff	Moderate
Age of consent and/or parental consent requirements	Unique
**Antiretroviral therapy**	
Cost	Moderate
Age restrictions/parental consent requirements	Unique
Low testing and knowledge of status	Unique
Retention in ART services	Unique
**Prevention of sexually transmitted infections and condom programmes for IDUs and their sexual partners**	
Lack of concern	Strong
Effect of drugs on decisions around safe sex	Strong
Distribution of low quality condoms	Unique
Conservative social climate	Unique
**Targeted information, education and communication for IDUs and their sexual partners**	
No information received in adolescence	Strong
Lack of outreach	Strong
**Prevention, diagnosis and treatment of viral hepatitis**	
Lack of knowledge of HCV	Strong
Lack of concern	Strong
Cost	Moderate
**Prevention, diagnosis and treatment of tuberculosis**	
Lack of knowledge of tuberculosis	Strong
Lack of concern	Strong

#### Structural barriers

One commonly cited reason why participants did not access NSPs, OST and HIV services were age restrictions and/or parental consent requirements. While Mauritius has no age restrictions on NSPs, lack of clarity in the law has led to rejection of adolescents from NSPs and a lack of awareness among adolescents that services should be available to them. In all consultations, age restrictions were raised as a barrier to adolescents accessing OST. In Kyrgyzstan and Mexico, age of consent to HIV testing was also cited as a barrier where positive results are only released in the presence of parents and/or guardian.

A third of consultations described fear of police harassment and arrest as a reason why young people prefer not to access NSPs. Fear of law enforcement was also an important barrier for purchasing syringes from pharmacies. Cost and distance to services was identified as a barrier to accessing OST, HIV testing, viral hepatitis testing, and ART, as was the need to travel to centralized locations.

#### Social barriers

Fear of being exposed as a person who uses drugs led to hesitance amongst young people to access NSPs, and a preference for obtaining injecting equipment from pharmacies, which were perceived as more discrete.

In Kyrgyzstan, concern was raised about the registration of methadone clients and the impact that has on a young person's life, “Nobody wants to start on methadone at 18 because they will register you at once [...], You will have no normal life after that, no driving license, and they will give data on you everywhere, at school, local police and to doctors” (young female, Kyrgyzstan).

The two female-only consultations held in Nepal and Nigeria, as well as the mixed-group consultations, provided insights into how the needs of females differ. Participants indicated that females start injecting at a similar age as males; however, young women are less likely to be in contact with services and are more concerned about their drug use being exposed. A number of participants explained how their male partners initiated them into injecting, while heavy reliance on their partners for injecting equipment meant that accessing services was not necessary.

#### Lack of youth-friendly services

The presence of older users at harm reduction services and their attitude towards younger users made young people uncomfortable. “I think that we should have other types of services to young people under 18 years old. Because the CAT [Government run drug service in Portugal] is a bit aggressive. Can you imagine going into a CAT, in the middle of all that junkies on methadone and shit, we think, ‘What is this??’ [laughs]. Like this, young people don't feel like going into a CAT if they are having problems!” (Male, Portugal).

Judgemental attitudes of staff members towards young people who access HIV testing and counselling was repeatedly raised as a deterrent. Whilst young people largely preferred accessing injecting equipment from pharmacies, participants also reported that pharmacists’ attitudes were negative and that they sometimes refused to sell syringes.

#### Lack of information and risk-perception

Lack of knowledge of services was an important barrier to accessing all services. An adolescent who is new to the injecting community was unlikely to know of NSPs. A lack of knowledge of OST was also reported, particularly in adolescence; some apprehension about OST also existed with participants hearing about issues such as loss of teeth and a more intense withdrawal from methadone than heroin.

In a majority of the consultations, the participants said they had no knowledge of HIV testing in their adolescence. It was also suggested that YPWID only become aware of HIV testing sites when they begin drug treatment. Low knowledge of viral hepatitis and tuberculosis testing sites was reported in a majority of the consultations.

Information received about HIV prevention and treatment, safe sex and safe drug use varied considerably across consultations from very good information (e.g. US and Portugal) to none at all (e.g. Nigeria). However, in the majority of consultations, it was reiterated that information was only received after risky behaviours had already taken place.

Concern about STIs, viral hepatitis and tuberculosis was low across consultations. Whilst condoms were generally accessible according to participants, in a majority of the consultations it was reported that concern for safe sex disappeared after using drugs. “If you are high you do not care” (young man, Romania). In a majority of consultations, participants said that they were not concerned enough to get tested for HIV, believing it was a problem for “older users.”

The belief that services are not needed emerged strongly from the consultations. Participants described enjoying their drug use and not experiencing many negative consequences yet, and thus did not feel it necessary to seek out services. In the Ukraine, a participant put it simply: “We don't need services. We have no problems.” Similarly, a young woman in Slovenia said, “But when you are under age you mostly don't want OST. Cos those are the years when things are still good even if you are already addicted.”

#### Beyond the comprehensive package: additional support needs

Participants expressed that they require support beyond the comprehensive package of harm reduction. The following interventions were suggested in a third or more of the consultations: safe injecting advice; vocational training, removal of stigma and policy barriers to employment; parental engagement and education; support for street-involved youth; improved school drug education with a greater focus on risk reduction; legalization of softer drug; and legal education.

Young users need to be taught a) how to use, b) how to use correctly, and c) how not to die. (young man, US)

## Discussion

The consultations show that a person who injects drugs in their adolescence differs from older persons who inject drugs, which put them at greater risk of harm. The implications of the findings from the community consultations for the comprehensive package of harm reduction services recommended by WHO [15] is now discussed.

Participants agreed that young people begin using other drugs before using and injecting opiates. In a number of the consultations, perceived changes in drug trends amongst young people were discussed, generally from heroin to legal highs and synthetic drugs including amphetamine-type-stimulants (ATS). This trend has been reported elsewhere [1,24]. Whilst these drugs are more often not injected, injecting of synthetic and stimulant drugs also occurs [25]. Use of stimulant drugs in the party scene is commonplace amongst many young people [26], a population often not in contact with traditional harm reduction services. ATS users rarely use harm reduction services as they do not see these services as relevant [27]. In developing services to address adolescents and young people, different outreach strategies will be needed to reach both non-injecting and non-opiate substance use.

Reaching young people *before* they start injecting is an opportunity to prevent initiation into injecting, and/or to provide support and education to inject safely if injection drug use is initiated. Where young people experienced rejection by family, school and society over using “softer” drugs and started injecting as a coping-strategy also shows the importance of a more supportive response to non-injecting substance use.

While teaching an adolescent how to inject drugs may raise serious ethical and legal considerations for many policy makers and service providers, the fact that safe injecting education was identified as a need by participants within the majority of the consultations deserves further exploration.

Given the social context of initiation, working through social networks to prevent initiation into injecting may be an effective approach [28]. Initiatives and research on effective models is limited however, and further work is needed.

Consistent with other research [29], consultations found that even when NSPs are available for minors without parental consent, young people in the consultations consistently preferred to access their injecting equipment from pharmacies or friends, making the risk of sharing syringes greater. If adolescents prefer to obtain their syringes from pharmacies, it may be important to ensure pharmacies are able to provide information and education, engage in behavior change and link to drug treatment programmes and HIV testing and treatment programmes.

The consultations suggested that young people may be uncomfortable accessing services that older injectors frequent. Services that include a range of services that are not drug-specific may be more effective in engaging and serving adolescents. Integration of harm reduction interventions into other services already in contact with at-risk youth may also be a good approach. Lack of knowledge of services was another recurring theme, suggesting that a need for specific outreach strategies in order to reach younger people is necessary.

Adolescence is a period of experimentation and for many this includes experimentation with drug use [2]. During the initial period of drug use, consultations showed that young people may not necessarily identify themselves as “drug users at risk and/or in need of services.” The participants in the consultations often stressed pleasure as a key motivator for using drugs, and during this period where they may not be experiencing too many adverse effects, they are unlikely to reach out for support.

This has implications for how to successfully establish initial contact and engagement into services. More creative methods are needed to engage a young person who does not see him/herself as a “drug user,” whilst re-orienting services to be responsive to people who engage in experimental and enjoyable drug use as opposed to being services only relevant for those who are experiencing difficulties and/or want to stop using drugs may also lead to more successful engagement of younger people.

Under current WHO guidelines, there are no recommended age restrictions on NSP or OST programmes, yet these pose clear barriers to accessing NSPs and OST for adolescents [30]. The comparative review of age restrictions and parental consent requirements in the countries where consultations were organized indicate arbitrary restrictions, and such restrictions were repeatedly mentioned as a reason why young people who participated in the consultations were unable to access services. Countries should consider harmonizing their age-restrictions to international guidelines.

Another important yet often overlooked issue that arose consistently across the consultations was the impact of drug use on unsafe sex practices amongst adolescents, which supports previous research [31]. Programmes that better address the connection between sexual health and drugs are needed, particularly for adolescents.

Young women who use drugs are more vulnerable to HIV due to a number of age- and gender-specific vulnerabilities to both injection and sexual transmission routes [32]. Consultations indicated that young women are less willing to access services, have less knowledge about services, HIV, Hepatitis and TB, and frequently share injecting equipment with their male partners [33]. Outreach strategies are especially important for young women as they may be more dependent on their partners for injecting equipment and are fearful of the greater stigma placed on them, thereby resisting accessing services. Comprehensive health services that also address their sexual and reproductive health needs are needed [34].

### Study limitations

While working through a network of community activists proved an invaluable asset in building trust, some facilitators noted difficulties in recruiting participants due to fear of exposure as a person who uses drugs. In addition, all participants were over 18 and were questioned about their experiences of injecting drugs under 18. Recall bias inherent in this approach and self-reporting introduces further respondent biases. Whilst all participants reported that they currently inject drugs or have done so in the past, there was no process to verify whether participants did inject under the age of 18. Given the highly sensitive topic of drug use, the group rather than individual consultation approach may have led to minority perspectives not being raised. To mitigate this, the facilitators were selected based on their closeness to the community and were trained.

## Conclusions

The findings presented in this paper provide a rare insight into the lived experiences of YPWID and the challenges they face in accessing harm reduction services. Interestingly, experiences were fairly consistent across the 14 different countries. For example, the fact that YPWID do not identify as a “drug user in need of services” may provide insights into why current approaches to outreach and service delivery may be failing. The consultations indicated that adolescents and young people require significant support beyond the comprehensive package of harm reduction, with clear linkages to other sectors such as social security, education and employment.

While the findings are not representative, they speak of the importance of conducting formative and action research together with young people who use drugs to understand context specific barriers, social norms within the community, and the dissonance between legal and policy environment and practice. In addition, the empowerment process from participatory approaches should be valued in its own right. While drug use among adolescents and young people is a sensitive topic, it is hoped that the lived experiences of young people themselves can engender more honest conversations on how to best address the reality of injecting drug use among young people to reduce risk and harm.

## References

[1] United Nations Office of Drugs and Crime (2014). Word drug report 2014.

[2] Barrett D, Hunt N, Stoicescu C Harm Reduction International. Injecting drug use among under 18s: a snapshot of available data [Internet]. http://www.ihra.net/files/2014/08/06/injecting_among_under_18s_snapshot_WEB.pdf.

[3] Joint United Nations Programme on HIV/AIDS (2013). Unpublished estimates.

[4] Cook C, Fletcher A, Barret D (2011). Youth drug-use research and the missing pieces in the puzzle: how can researchers support the next generation of harm reduction approaches. Children of the drug war: perspectives on the impact of drug policies on young people.

[5] Kissin DM, Zapata L, Yorick R, Vinogradova EN, Volkova GV, Cherkassova E (2007). HIV seroprevalence in street youth, St Petersburg, Russia. AIDS.

[6] Atkinson J, McCurdy S, Williams M, Mwambo J, Kilonzo J (2011). HIV risk behaviours, perceived severity of drug use problems and prior treatment experiences in a sample of young heroin injectors in Dar es Salaam, Tanzania. Afr J Drug Alcohol Stud.

[7] Inter-Agency Working Group on Key Populations (2014). HIV and young people who inject drugs [Internet]. http://www.who.int/hiv/pub/guidelines/briefs_pwid_2014.pdf.

[8] Fennema JSA, Ameijden EJCV, Hoek AVD, Coutinho RA (1997). Young and recent-onset injecting drug users are at higher risk for HIV. Addiction.

[9] (2010). FHI. Young People Most at Risk of HIV: A Meting Report and Discussion Paper form the Interagency Youth Working Group, US Agency for International Development, the Joint United Nations Programme on HIV/AIDS (UNAIDS) Inter-Agency Task Team on HIV and Young People, and FHI.

[10] Merkinaite S, Grund JP (2009). Young people and injecting drug use in selected countries of Central and Eastern Europe.

[11] Kral A, Lorvick J, Edlin B (2000). Sex-and drug-related risk among populations of younger and older injection drug users in adjacent neighborhoods in San Francisco. J Acquir Immune Defic Syndr.

[12] Curth N, Hansson L, Storm F, Lazarus J (2009). Select barriers to harm reduction services for IDUs in Eastern Europe. Cent Eur J Public Health.

[13] Merkinaite S, Grund JP, Frimpong A (2010). Young people and drugs: next generation of harm reduction. Int J Drug Policy.

[14] Debeck K, Wood E, Zhang R, Buxton J, Montaner J, Kerr T (2011). A dose-dependent relationship between exposure to a street-based drug scene and health-related harms among people who use injection drugs. J Urban Health.

[15] WHO, UNODC, UNAIDS (2012). Technical guide for countries to set targets for universal access to HIV prevention, treatment and care for injecting drug users: 2012 revision.

[16] UNAIDS (2011). Securing the future today: synthesis of strategic information on HIV and young people.

[17] Dickert N, Sugarman J (2005). Ethical goals of community consultation in research. Am J Public Health.

[18] Harm Reduction International The global state of harm reduction 2012. Towards an integrated response [Internet]. http://www.ihra.net/files/2012/07/24/GlobalState2012_Web.pdf.

[19] World Health Organization HIV testing and counselling: age of consent to test [Internet]. http://apps.who.int/adolescent/second-decade/section8/page6/policies-can-block-access.html.

[20] UNESCO, UNFPA, UNAIDS, UNDP, Youth LEAD (2013). Young people and the law in Asia and the Pacific. A review of laws and policies affecting young people's access to sexual and reproductive health and HIV services [Internet]. http://unesdoc.unesco.org/images/0022/002247/224782E.pdf.

[21] Fox K, Ferguson J, Ajose W, Singh J, Marum E, Baggaley R (2013). World Health Organization. HIV and adolescents: guidance for HIV testing and counselling and care for adolescents living with HIV. Annex15: Adolescent consent to testing: a review of current policies and issues in sub-Saharan Africa [Internet].

[22] Patton MQ (2001). Qualitative research and evaluation methods.

[23] Guest G, Macqueen K, Namey E (2012). Applied thematic analysis.

[24] United Nations Office on Drugs and Crime (2013). Patterns and trends of amphetamine-type stimulants and other drugs; challenges for Asia and the Pacific. A report from the Global SMART Programme. https://www.unodc.org/documents/southeastasiaandpacific//Publications/2013/ats-2013/2013_Regional_ATS_Report_web.pdf.

[25] Colfax G, Santos GM, Chu P, Vittinghoff E, Pluddermann A, Kumar S (2010). Amphetamine-group substances and HIV. Lancet.

[26] United Nations Office of Drugs and Crime (2007). Preventing amphetamine-type stimulant use among young people: a policy and programming guide.

[27] World Health Organisation (2011). Technical brief 2: harm reduction and brief intervention for ATS users, Technical Briefs on amphetamine-type stimulants (ATS).

[28] Werb D, Buxton J, Shoveller J, Richardson C, Rowell G, Wood E (2013). Interventions to prevent initiation of injection drug use: a systematic review. Drug Alcohol Depend.

[29] Busza J, Douthwaite M, Bani R, Scutelniciuc O, Preda M, Simic D (2013). Injecting behavior and service use among young injectors in Albania, Moldova, Romania and Serbia. Int J Drug Policy.

[30] Fletcher A, Krug A (2012). ‘Excluding youth? A global review of harm reduction services for young people who use drugs’, in the 2012 Global State of Harm Reduction.

[31] Loxley W (2000). Double risk: young injectors and sexual relationships. Sex Relationsh The.

[32] Evans J, Hahn J, Page-Shafer K, Lum P, Davidson P, Moss A (2003). Gender differences in sexual and injection risk behavior among active young injection drug users in San Francisco. J Urban Health.

[33] Gyarmathy V, Li N, Tobin K, Hoffman I, Sokolov N, Levchenko J (2010). Injecting equipment sharing in Russian drug injecting dyads. AIDS Behav.

[34] Eurasian Harm Reduction Network (2010). Women and drug policy in Eurasia.

# Sub article: “First, do no harm”: legal guidelines for health programmes affecting adolescents aged 10–17 who sell sex or inject drugs

**DOI:** 10.7448/IAS.18.2.19437

**Published:** 2015-02-26

**Authors:** Brendan Conner

**Affiliations:** Technical Advisor, HIV Young Leaders Fund

**Keywords:** young key populations, adolescent key populations, young drug users, harm reduction, commercial sexual exploitation of children, sexually exploited children, juvenile prostitution, young sex workers, young people, minimum intervention

## Abstract

**Introduction:**

There is a strong evidence base that the stigma, discrimination and criminalization affecting adolescent key populations (KPs) aged 10–17 is intensified due to domestic and international legal constructs that rely on law-enforcement-based interventions dependent upon arrest, pre-trial detention, incarceration and compulsory “rehabilitation” in institutional placement. While there exists evidence and rights-based technical guidelines for interventions among older cohorts, these guidelines have not yet been embraced by international public health actors for fear that international law applies different standards to adolescents aged 10–17 who engage in behaviours such as selling sex or injecting drugs.

**Discussion:**

As a matter of international human rights, health, juvenile justice and child protection law, interventions among adolescent KPs aged 10–17 must not involve arrest, prosecution or detention of any kind. It is imperative that interventions not rely on law enforcement, but instead low-threshold, voluntary services, shelter and support, utilizing peer-based outreach as much as possible. These services must be mobile and accessible, and permit alternatives to parental consent for the provision of life-saving support, including HIV testing, treatment and care, needle and syringe programmes, opioid substitution therapy, safe abortions, antiretroviral therapy and gender-affirming care and hormone treatment for transgender adolescents. To ensure enrolment in services, international guidance indicates that informed consent and confidentiality must be ensured, including by waiver of parental consent requirements. To remove the disincentive to health practitioners and researchers to engaging with adolescent KPs aged 10–17 government agencies and ethical review boards are advised to exempt or grant waivers for mandatory reporting. In the event that, in violation of international law and guidance, authorities seek to involuntarily place adolescent KPs in institutions, they are entitled to judicial process. Legal guidelines also provide that these adolescents have influence over their placement, access to legal counsel to challenge the conditions of their detention and regular visitation from peers, friends and family, and that all facilities be subject to frequent and periodic review by independent agencies, including community-based groups led by KPs.

**Conclusions:**

Controlling international law specifies that protective interventions among KPs aged 10–17 must not only include low-threshold, voluntary services but also “protect” adolescent KPs from the harms attendant to law-enforcement-based interventions. Going forward, health practitioners must honour the right to health by adjusting programmes according to principles of minimum intervention, due process and proportionality, and duly limit juvenile justice and child protection involvement as a measure of last resort, if any.

## Introduction

It is well settled that adolescent key populations (KPs) face heightened health risks as a result of law and policy barriers to accessing HIV treatment, diagnostic and prevention services and that the stigma, discrimination and criminalization experienced by adolescent KPs aged 10–17 is intensified, as compared to their older cohorts. Those adolescents who sell sex or inject drugs more often face aggravating circumstances such as family rejection and street-involvement, combined with legal constructs concerning consent and age of majority. These constructs may condition access to life-saving treatment on parental consent or proof of “emancipation,” or lower the age of consent to sell sex or use drugs as compared to general consent-to-sex law, resulting in increased police encounters and commitment to state custody [1,2].

This commentary addresses only adolescent KPs aged 10–17 and not adolescents aged 18–19 in order to spotlight the specific, legal guidelines for health and protective interventions that apply to persons under the legal age of majority. It must be cautioned that developmentally adolescence is a dynamic state and that major differences may exist in identity, understanding and behaviour between adolescents within this range. While it is true that adolescents aged 10–17 who are transgender (TG) or young men who have sex with men (YMSM) face related challenges [3,4], in order to tailor recommendations to the special legal framework applicable to these populations, this paper narrows its scope to adolescents who sell sex or use drugs, including the disproportionate number of adolescent TG and YMSM who do so.

There is a strong and emergent evidence base that law enforcement-based interventions targeting adolescents aged 10–17 who sell sex or use drugs result in affirmative harm to the very same adolescents they are intended to protect [1,2,5–7]. The daily reality reported by many adolescents who sell sex or use drugs is dominated by harassment, theft, detention, deportation, and physical and sexual violence by law enforcement and military personnel, including rape and extortion in exchange for release [1,2,8–10]. This lived experience of violence and corruption instills fear in adolescents and prevents adolescent victims from reporting the crimes committed against them [1,2,8]. It similarly poisons relationships with service providers who may be compelled to report adolescents to law enforcement as a result of mandatory reporting laws [1,2]. What is more, adolescent KPs report abuse by health practitioners themselves, including discrimination and service denial, and even physical and sexual violence, forced abortions, breach of confidentiality and mandatory HIV testing [1,2,8].

Despite this frightening reality, a child protection framework is frequently applied at the country level to justify arrest-based interventions without reference to international human rights law governing the administration of juvenile justice, child protection and the right to health. The overwhelming majority of low- and middle-income countries possess little to no specialized child protection services. Thus “child protection” falls to precisely those persons identified by young KPs as perpetrators of violence: uniformed services, primarily police and military personnel. Even in high-income countries, arrest-based interventions are relied upon, with diversion to “services,” if at all, only *after* arrest in violation of international law and without clear guidelines for appeal or periodic review of the conditions of confinement [1,8,11]. Ironically, the very same interventions championed in the name of “child protection” are therefore increasing the numbers of one such KP – adolescents in prisons and other closed settings.

The confusing policy and programme environment has caused many healthcare providers to limit or cease services to adolescents aged 10–17 who sell sex or use drugs. In effect, implementation of international legal principles has drawn a red line at the age of majority that health practitioners dare not cross. The strong, evidence-based technical guidelines that exist for rights-based health interventions among sex workers and injecting drug users *over* the age of majority decline to extend rights-based recommendations to KPs aged 10–17 even when medical science invites an equivalent approach [12,13]. This commentary calls for a reexamination of the treaty framework, situating child-protective interventions firmly within international law and guidance governing the right to health, and regulating juvenile justice and child welfare interventions according to principles of minimum intervention, last resort, due process and proportionality. These important principles clarify that state obligations to respect, protect and fulfil the rights of KPs aged 10–17 are not simply directed at the prohibition of behaviours, but also to protect vulnerable young people from the inherent harms of certain law enforcement-based interventions undertaken for the purposes of “protection,” which are a direct result of the current misreading of the international legal framework.

The commentary also advances concrete guidelines for framing health programme interventions, specifying that:

1. The principle of non-criminalization mandates non-compliance of healthcare providers with arrest-based interventions, an immediate end to arrest and prosecution of adolescent KPs aged 10–17, and the abolition of involuntary custodial placement in the name of “rehabilitation”;
2. Voluntary, confidential and adolescent-friendly primary, sexual and reproductive health services;
3. The right of adolescents aged 10–17 who sell sex or use drugs to be heard includes meaningful participation in policy and decision-making in health services and other programmes that concern them, as well as reliable complaint procedures and remedies for rights violations;
4. Parental consent waiver for life-saving sexual and reproductive health services, HIV and harm-reductionist treatment;
5. Client-centred informed consent and right to refuse or consent to participation in medical treatment and research trials.

## Discussion

The Convention on the Rights of the Child is organized around the principle that “[i]n all actions concerning children … the best interests of the child shall be a primary consideration” [14, art. 3(1)]. This principle expressly includes non-state actors such as civil society groups and medical practitioners whose actions concern children [14]. This determination depends on a variety of individual circumstances, such as the nature of the decision being made, the age and the level of maturity of the child, the views of the child, the capacity and circumstances of caregivers to provide adequate food, clothing and medical care, and the safety and health risks of the alternative circumstances proposed. As such the proposed programmatic guidelines must be adjusted to the individual circumstances of the adolescent. Nonetheless, this commentary advances several preliminary guidelines based on international law, with the expectation that international agencies and civil society groups led by those adolescents affected will revisit them.

### 

#### The principle of non-criminalization

The categories “respect, protect and fulfil” are often used to summarize the obligations of States parties as signatories to human rights treaties. States parties are obliged to “respect” by not interfering with the enjoyment of human rights, to “protect” individuals and groups against human rights abuses and to “fulfil” by taking positive action to facilitate the enjoyment of basic human rights [15]. In reference to adolescents aged 10–17 who sell sex or use drugs, the obligation to “protect” has suffered from a perverse misapplication at the country level. The child protection framework has been used to justify law enforcement-based interventions without reference to international human rights law governing the administration of juvenile justice, child protection and the right to health.

The four guiding principles identified by the CRC Committee include non-discrimination (article 2); devotion to the best interests of the child (article 3); the right to life, survival and development (article 6); and respect for the views of the child (article 12) [16]. With the exception of the non-discrimination principle, these guidelines largely rely on an understanding of adolescents’ “positive” rights, as in an adolescent's right *to* receive life-saving medical treatment or right *to* have her view respected. Yet in prioritizing these principles, the Committee neglects the Convention's protection of “negative” liberties, primarily those international standards governing the application of judicial measures, institutional placements and protective interventions targeting persons under the age of majority. The CRC Committee has repeatedly ruled that no provision of the Convention may be read in isolation from other provisions more conducive to the rights of the child [17,18].

This neglect of the Convention's principles of negative liberty likely stems from the Convention's article 33 and 34 stipulations that States parties must “take all appropriate measures … to protect children from the use of narcotic drugs and psychotropic substances” and to “protect the child from all forms of sexual exploitation …” [14, arts. 33–34]. The Optional Protocol to the Convention on the Rights of the Child on the Sale of Children, Child Prostitution and Child Pornography (OPSC) also requires States parties to adopt criminal, civil and administrative penalties for the sale of children, child prostitution and child pornography [19]. The Protocol also expands the Convention's guarantee of protection of the rights and interests of child victims in criminal proceedings against their perpetrators [19]. The initial difficulty is that these treaties remain silent on the definition of “appropriate measures” for protective interventions. The qualifier “appropriate” is said to have been intended to act as a prophylactic against “arbitrariness, disproportionate measures and abuses of human rights in pursuit of protecting children ….” [7, p. 37, ¶ 73]. Commentators have interpreted the term to indicate that protective interventions must be based on adequate data, targeted and effective and proportionate such that they are in pursuit of a legitimate aim and tailored such that they are no more than necessary for the achievement of that aim [7, pp. 38–39, ¶ 76].

While a comprehensive framework regulating protective measures has eluded the CRC Committee, it has articulated certain limits. The CRC Committee has stated in the context of adolescents aged 10–17 who sell sex that the obligation to protect extends to ending their arrest and prosecution under national criminal or other laws [20]. The CRC Committee has consistently noted in its dialogue with States parties to the Optional Protocol that those adolescents who sell sex should “be neither criminalized nor penalized, and that all possible measures should be taken to avoid their stigmatization and social marginalization” [21, p. 8, ¶ 25]. The Committee has specifically criticized States parties with inadequate legislation and contradictory provisions on this issue [20,21]. The jurisprudence on arrest-based interventions for adolescents aged 10–17 who use drugs is slightly less certain, although the Committee's Concluding Observations in reference to article 33 protection from drug use and dependence use similar phrasing, namely that a child who uses drugs is to be “seen as a victim, not a criminal” [7, p. 41, ¶ 81]. It therefore is beyond argument that an arrest or prosecution brought for the purpose of bringing an adolescent aged 10–17 who sells sex or uses drugs before a juvenile or criminal court is violative of international law.

The question then becomes whether the non-criminalization principle bars the custodial arrest of adolescents aged 10–17 who use drugs or sell sex for purposes of bringing state custody proceedings against the adolescent for commission of a status offence or an abuse or neglect proceeding. While this question is beyond the scope of this article and will be addressed in a future writing, it is the author's position that custodial arrests are, as a form of temporary detention and by virtue of the involvement of uniformed services, a *per se* violation of the non-criminalization principle contained in international guidance such as the Riyadh Guidelines and the 2010 UN Guidelines on the Alternative Care of Children.

The CRC Committee's jurisprudence also specifically contemplates the health-related dangers of law enforcement-based interventions on adolescent KPs aged 10–17. The Committee's General Comment on HIV/AIDS acknowledges that rape and other sexual abuse by child protection officers, law enforcement and detention personnel expose adolescents to increased risk of sero-conversion [18]. The Committee's General Comment on Adolescent Health and Development recommends comprehensive health services specific to adolescents who sell sex and notes that it is the obligation of States parties to treat such youth “as victims and not as offenders” [22, p. 10, ¶ 37] and that States parties should also “ensure adolescents affected by poverty who are socially marginalised are not criminalised” [22, p. 4, ¶ 12].

Despite the clarity of the Committee's decisions concerning the principle of non-criminalization, its periodic reporting guidelines fail to adequately apprise or require reporting on States parties’ attendance to the health consequences specific to adolescents in conflict with the law. The CRC Committee's periodic reporting mechanism would be improved were it to more strictly account for health consequences affecting adolescents in conflict with the law. While the protection of adolescents from drug use and dependence is now appropriately dealt with under the “disability, basic health and welfare” cluster, the protection of adolescents from sexual exploitation and sexual abuse, children in street situations, and children in conflict with the law improperly remain under the “special protection measures” cluster [23,24]. Under the “disability, basic health and welfare” cluster, States parties are required to take into account the General Comment on HIV/AIDS [23,24]. While the CRC Committee requires that States parties take into account the Committee's jurisprudence on children's rights in juvenile justice under the “special protection measures” cluster, it is rarely the case that States parties do so in the context of adolescents who sell sex or use drugs [23,24].

#### The right to voluntary, confidential and adolescent-friendly health services

It is common for legal analyses of health interventions relevant to KPs aged 10–17 to rely on a “right to health” lens. The reliance on the right to health provisions is fitting for a movement organized around “universal access,” as the Convention clarifies that no adolescent aged 10–17 must be “deprived of his or her right of access to such health care services” [14, art. 24(1)]. Article 24 of the Convention requires that signatories “recognise the right of the child to the enjoyment of the highest attainable standard of health and to facilities for the treatment of illness and rehabilitation of health” [14]. States parties are also obligated “to promote physical and psychological recovery and social reintegration of a child victim of: any form of neglect, exploitation, or abuse …” [14, art. 39]. It is important to note that this principle is to be read in tandem with the Committee's jurisprudence on the principle of non-criminalization such that *involuntary* “rehabilitation” is violative of international law.

The right to health must not be restricted to health and mental health services, but include the article 26 right to social security and article 27 right to a standard of living adequate for adolescent development, particularly clothing, nutrition and, if desired, immediate shelter and long-term housing [1,2,14,25,26]. It is therefore advisable that health practitioners working with adolescents aged 10–17 who use drugs or sell sex integrate comprehensive social services into health programmes, and establish a reliable and safe referral network.

The “health and human rights” framework is also appealed to in support of adolescents’ sexual and reproductive health and rights. The CRC Committee's General Comment No. 15 in particular elaborates on these principles [25]. The Committee stipulates that freedoms inherent in children's right to health “include the right to control one's health and body, including sexual and reproductive freedom to make responsible choices” [25, p. 8, ¶ 24]. The Committee has interpreted this right rather broadly in reference to services, to include a right to access a range of facilities and goods as well as prevention, treatment, rehabilitation and palliative care services [25].

On the other hand, the CRC Committee has been less clear on the parameters of parental consent. The Committee suggests waiver is necessary for life-saving treatment but at the same time attenuates the right to sexual and reproductive health according to the Convention's principle of “evolving capacities,” contained in article 5 of the Convention [14,22]. This principle is also leveraged to support the view that States parties bear responsibility to build adolescents’ capacity for informed decision-making in these matters through comprehensive sexuality education and access to confidential and adolescent-friendly sexual and reproductive health services [15,16]. Those who provide healthcare may also rely on the fact that many forms of sexual and reproductive health services are life-saving in nature, including but not limited to prevention, care and treatment of HIV, access to safe abortion and access to gender-affirming treatment for TG adolescents.

The Convention also firmly guarantees the right to privacy (article 16), particularly in the context of HIV prevention, treatment and care of adolescents [14,18]. The CRC Committee's General Comment on HIV/AIDS states that State parties “must protect the confidentiality of HIV test results … including *within* health and social welfare settings, and information on the HIV status of children may not be disclosed to third parties, including parents, without the child's consent” [18, p. 8, ¶ 24]. Nonetheless, health professionals and other service providers report a conflict between their reporting obligations and the young person's expectation of confidential care [1,2,8]. The CRC Committee has yet to rule definitively on the right to privacy in relation to mandatory reporting, and contrary domestic laws may be in force.

While there already exists powerful guidance on consent and confidentiality for young adult and adult KPs [12,13,27], international actors are cautious in advancing similar recommendations for KPs aged 10–17. The WHO has recommended HIV testing and counselling, with linkages to prevention, treatment and care, for adolescents from KPs in all epidemic scenarios, and specified that consent and confidentiality must be ensured so that services are not used in punitive or coercive ways for adolescent KPs [28]. Yet in still other cases bold and necessary recommendations for rights-based health programming are lacking. For instance, while the UN system endorsed a core package of nine essential harm-reduction services for people who inject drugs which have been shown to reduce HIV infections [13], they are not youth-focused, and key issues regarding young people, IDU and HIV may be falling between the priority areas of different international organizations [29].

It is clear from the Committee's decisions that it not only rejects this trepidation but also argues for the opposite view, namely that the more vulnerable the adolescent, the more critical the right to informed consent and confidentiality. The CRC Committee has specifically held in its General Comment on HIV/AIDS that the Convention requires States parties to ensure access to voluntary, confidential HIV counseling and testing and that prevention programmes must “acknowledge the realities of the lives of adolescents” [18, p. 4, ¶ 11]. The CRC Committee has repeatedly emphasized the importance of adolescent-friendly health services that are “friendly and supportive, provide a wide range of services and information, are geared to their needs, give them the opportunity to participate in decisions affecting their health, are accessible, affordable, confidential and non-judgemental, do not require parental consent and are not discriminatory” [18, p. 7, ¶ 20].

The World Health Organization's quality of care framework provides a useful metric for “adolescent-friendly services” in practice for adolescent KPs: available, accessible, appropriate, equitable and effective [30–32]. In certain locales, health services such as first- or second-line antiretroviral drugs (ARV) or safe abortions are simply not *available* to anyone regardless of age [30,33]. Where health services are available, however, adolescents may yet find them not *accessible* due to unaffordability, remoteness of location or incongruity of hours, or restrictive laws and policies, such as denial of services to non-citizens or migrants, bans on provision of contraception to unmarried adolescents who sell sex, prohibitions on sterile injecting equipment and hormone treatment for adolescents who inject drugs and TG adolescents, or stringent identification requirements [30,33]. Still other services that are available and accessible may yet be delivered in such a way that adolescents are not willing to use them because they are not *acceptable* or safe for young people, for instance, by a doctor known to criticize YMSM who sell sex about the origin of STIs or their feminine appearance, or engage in regular breaches of confidentiality as to the young person's behaviour or HIV status [30,33]. Further, health services must be *appropriate* such that the health services an adolescent actually needs are provided, such as an adolescent who sells sex seeking PrEP and not simply condom distribution or counseling, and *effective* in that the right health services are provided in the right way, and make a positive contribution to the adolescent's health [32]. Finally, health services must be *equitable* to the extent that they do not cater to some adolescent groups and not others, such as a clinic that provides confidential HIV prevention, care and treatment to young people from high-income backgrounds but does not reach street-based young people [30,31]. In other words, all adolescents and not just selected groups are able to obtain the health services that are available [32].

#### The right of meaningful participation in decision-making regarding policy and health programmes

The CRC Committee has identified the article 12 right to be heard as one of the four general principles of the Convention, and repeatedly emphasized it is also to be considered in the interpretation and implementation of all other rights [14,34]. The Committee has specified that this right includes the meaningful participation of adolescents in decision-making, policymaking and preparation of laws, as well as the adoption of complaint procedures and remedies [34]. The CRC Committee's General Comment on HIV/AIDS notes that States parties must provide adolescents with the means “to fully participate at both community and national levels in HIV policy and programme conceptualization, design, implementation, coordination, monitoring and review” [18, p. 5, ¶ 12].

In the context of adolescents aged 10–17 who sell sex or use drugs, in crediting this right it is particularly important that health practitioners consider the greater capacity of adolescents 10–17 who are living independently, have no parents/guardians or no contact with them, have abusive parents/guardians, or who are pregnant [1,2,34].

Article 12 of the International Covenant on Economic, Social and Cultural Rights (ICESCR) further reinforces the right of all people to the highest attainable standard of physical and mental health [35]. The ESCR Committee's General Comment 14 recommends States parties ensure adolescent-friendly healthcare, and adolescents’ “opportunity to participate in decisions affecting their health, to build life-skills, to acquire appropriate information, to receive counselling and to negotiate the health-behaviour choices they make” [36, p. 9, ¶ 23].

#### The right to informed consent and to refuse treatment and research trials

The mixed policy and programme environment has resulted in longstanding limitations on data collection, service provision and medical treatment to adolescent KPs aged 10–17 [1,2,37,38]. The reluctance by international actors to take a position is reflected in the dearth of medical trials, monitoring or evaluation of adolescent KPs aged 10–17 who sell sex or use drugs. Even where surveys do monitor prevalence and trends of drug use among young people, they are almost always still based on school samples that neglect street-based and out-of-school youth, and people who inject drugs remain largely invisible in the official statistics on youth drug use [29].

The “solutions” to the research gap proposed by international health actors remain incomplete as a result of unclear international legal guidance. For instance, in a series of joint UNAIDS and WHO meetings regarding ethical guidelines for engaging PWID in HIV prevention trials, it was recommended that researchers seek the adolescents’ permission to disclose use of injecting drugs before making contact with parents, and if they are not willing to do so, they should not be included in the study [37,38]. While the approach appropriately honours the right to privacy and right to refuse or consent to participation in medical treatment or research trials by preventing disclosure of drug use or the sale of sex to guardians, it fails to confirm the right of adolescents to confidentiality *and* at the same time the positive right to go forward with said life-saving treatment [18]. As a result, service providers may resort to not asking clients their age in order to provide them with assistance, and to avoid enforcement of age restrictions on accessing harm-reduction services, preventing the disaggregation of data by age [7].

#### The right to treatment and waiver of parental consent

Current treatment guidelines fail to honour the right of adolescents to life-saving treatment. In the joint UNAIDS and WHO Eastern Europe & Central Asia experts meetings regarding ethical guidelines for engaging adolescents aged 10–17 who inject drugs in HIV prevention trials, researchers concluded that absent parental consent for medical treatment for HIV or STIs, NSP or OST, researchers may not provide this life-saving treatment to adolescents where parental consent is required by domestic legislation [38]. In the words of the consultation report, “[r]esearchers should not conduct trials with proven interventions with the aim of bringing about change in law and policy” [37, p. 27].

The Committee recently explained that children, in accordance with their evolving capacities, should have access to confidential counselling without consent of a guardian or a parent [14,34]. In addition, states should consider “allowing children to consent to certain medical treatments and interventions without the permission of a parent, caregiver, or guardian, such as HIV testing and sexual and reproductive health services, including education and guidance on sexual health, contraception and safe abortion” [25, p. 9, ¶ 31]. It should go without saying that life-saving medical treatment for HIV or STIs, and critical harm-reduction resources such as NSP and OST, must be made available to all adolescents – whose right to life and health trumps a guardian's right to care and custody. It is incumbent on the Committee on the Rights of the Child to elaborate on these opinions, and make clear that even the most marginalized adolescents are provided life-saving medical treatment.

## Conclusions

The “cure,” as the idiom goes, may be worse than the problem it intends to remedy. This is precisely the case for health interventions among adolescents aged 10–17 who use drugs or sell sex. Without full implementation of the above principles, funders, researchers, health practitioners and community-based KP groups may ignore the urgent needs of KPs aged 10–17 in order to protect themselves from prosecution. Without appropriate guidance, the medical analogue to the principle of minimal intervention – “first, do no harm” – may persuade peer and other health practitioners to practice life-saving interventions in the shadow of threatening and outdated interpretations of the law.

## Author information

Brendan Michael Conner, Esq., is a Staff Attorney at Streetwise and Safe, where he provides civil legal representation and policy support to LGBTQQ youth of color who experience police encounters and government benefits discrimination in New York City, including youth with experience in trading sex for survival needs. He has served as an expert on international law and young key populations, including as Technical Advisor to the HIV Young Leaders Fund. He has worked both independently and in partnership with the Fremont Center on projects for clients such as Safe Horizon's Streetwork Project, the UN Development Programme (UNDP), the United States Agency for International Development (USAID) and the Open Society Institute. The opinions expressed in this article are the author's own and do not necessarily reflect the views of Streetwise and Safe.

## References

[1] WHO, UNAIDS, IAWG (2014). HIV and young people who sell sex: a technical brief.

[2] WHO, UNAIDS, IAWG (2014). HIV and young people who sell drugs: a technical brief.

[3] WHO, UNAIDS, Inter-Agency Working Group on Key Populations (“IAWG”) (2014). HIV and young men who have sex with men: a technical brief.

[4] WHO, UNAIDS, IAWG (2014). HIV and young transgender people: a technical brief. Draft report.

[5] Global Commission on HIV and the Law (2012). HIV and the law: risks, rights and health.

[6] Rahman F Legal and policy considerations in reaching young key populations.

[7] Barrett D, Veerman P (2012). Article 33: protection from narcotic drugs and psychotropic substances.

[8] Conner B, Mago A, Middleton-Lee S (2014). Sexual and reproductive health needs and access to health services for adolescents under 18 engaged in selling sex in Asia Pacific.

[9] Youth Voices Count (2013). Policy brief on self-stigma among young men who have sex with men and young transgender women and the linkages with HIV in Asia.

[10] UNESCO, UNFPA, UNAIDS, UNDP, Youth Lead (2013). Young people and the law in Asia and the Pacific: a review of laws and policies affecting young people's access to sexual and reproductive health and HIV services.

[11] McClure C, Chandler C, Bissell S (2014). Responses to HIV in sexually exploited children or adolescents who sell sex. Lancet.

[12] WHO, UNFPA, UNAIDS, NSWP (2012). Prevention and treatment of HIV and other sexually transmitted infections for sex workers in low-and-middle-income countries: recommendations for a public health approach.

[13] WHO, UNODC, UNAIDS (2009). Technical guide for countries to set targets for universal access to HIV prevention, treatment and care for injecting drug users.

[14] UN Convention on the Rights of the Child, G.A. res. 44/25, annex, 44 UN GAOR Supp. (No. 49) at 167, UN Doc. A/44/49 (1989), entered into force Sept. 2 (1990).

[15] Schabas W, Sax H (2006). Article 37: prohibition of torture, death penalty, life imprisonment and deprivation of liberty.

[16] CRC Committee

[17] CRC Committee

[18] CRC Committee

[19] Optional Protocol to the Convention on the Rights of the Child on the Sale of Children, Child Prostitution and Child Pornography, adopted by G.A. Res. 54/263, opened for signature 25 May 2000 (entered into force 18 January 2002)

[20] Muntarbhorn V (2007). Article 34: sexual exploitation and sexual abuse of children.

[21] Report of the Committee on the Rights of the Child General Assembly, Sixty-third Session.

[22] CRC Committee (2003). General Comment No. 4: adolescent health and development in the context of the Convention on the Rights of the Child CRC/GC/2003/4.

[23] CRC Committee

[24] CRC Committee

[25] CRC Committee

[26] Eide A, Eide B (2006). Article 24: the right to health.

[27] WHO, UNAIDS, MSMGF, UNDP (2011). Prevention and treatment of HIV and other sexually transmitted infections among men who have sex with men and transgender people: recommendations for a public health approach.

[28] WHO Consolidated guidelines on the use of antiretroviral drugs for treating and preventing HIV infection: recommendations for a public health approach.

[29] Fletcher A, Krug A, Stoicescu C (2012). Excluding youth? A global review of harm reduction services for young people. The global state of harm reduction 2012: towards an integrated response.

[30] WHO (2003). Adolescent-friendly health services: an agenda for change.

[31] WHO (2012). Making health services adolescent friendly: developing national quality standards for adolescent friendly health services.

[32] WHO (2009). Quality assessment guidebook. A guide to assessing health services for adolescent clients.

[33] Conner B (Forthcoming). “First, do no harm”: an advocacy brief on sexual and reproductive health needs and access to health services for adolescents 10–17 engaged in selling sex in the Asia Pacific.

[34] General Comment No. 12: right of the child to be heard, UN Doc

[35] International Covenant on Economic

[36] Committee on Economic, Social and Cultural Rights (“CESCR”)

[37] WHO, UNAIDS (2010). Ethical engagement of people who inject drugs in HIV prevention trials.

[38] WHO, UNAIDS (2011). Ethical engagement of people who inject drugs in HIV prevention trials.

# Sub article: Young key populations and HIV: a special emphasis and consideration in the new WHO Consolidated Guidelines on HIV Prevention, Diagnosis, Treatment and Care for Key Populations

**DOI:** 10.7448/IAS.18.2.19438

**Published:** 2015-02-26

**Authors:** Rachel Baggaley, Alice Armstrong, Zoe Dodd, Ed Ngoksin, Anita Krug

**Affiliations:** 1 HIV Department, World Health Organization, Geneva, Switzerland; 2 International Network of People Who Use Drugs, Toronto, Canada; 3 Global Network of People Living with HIV, Cape Town, South Africa; 4 Youth Research Information Support Education (Youth RISE), Melbourne, Australia

WHO released its new *Consolidated Guidelines on HIV Prevention, Diagnosis, Treatment and Care for Key Populations* [1] at the International AIDS Conference in Melbourne in July 2014. This guidance addresses five key populations: men who have sex with men, people who inject drugs, people in prisons and other closed settings, sex workers and transgender people. For the first time in its work on key populations, WHO chose to specifically address adolescent and young key populations, considered specific adolescent issues relating to all recommendations and implementation considerations, highlighted case examples and discussed challenges and barriers to acceptable and effective service delivery. In addition, four technical briefs, developed by the Interagency Working Group of Key populations, on HIV and young men who have sex with men, young people who sell sex, young people who inject drugs and young transgender people have been included as annexes to the guidelines.

## High HIV risk: limited data

In all epidemic contexts, HIV incidence remains high or is increasing among key populations (Figure 1). Currently, there is a lack of global data pertaining to estimates of adolescent and young key populations, as well as their risks and needs. Where accurate surveillance data for young key populations are available, the HIV prevalence among these groups is often found to be significantly higher than that of the general youth population [3]. Available data are often not disaggregated by age, and those under 18 years are underrepresented in research. However, what we do know paints a stark picture.

**Figure 1:** New infections attributable to key populations. From Ref. [3].

According to the report of the Commission on AIDS in Asia, nearly all (95%) new HIV infections among young people in Asia occur in young key populations. In this region, however, over 90% of HIV resources for young people are focused on programming for “low-risk youth” [4]. Furthermore, studies consistently demonstrate that young key populations are even more vulnerable than older cohorts to sexually transmitted infections, including HIV and other sexual and reproductive health concerns [5–9].

Available data also suggest adolescent key populations are disproportionately affected by HIV in almost all settings [10]. For example, pooled data show significantly higher HIV prevalence and increasing rates of new HIV infections among adolescent men who have sex with men than among men of the same age in the general population [11,12]. Among adolescent males aged 13–19, in the United States, 92.8% of all diagnosed HIV infections were attributed to male-to-male sexual contact [13,14]. HIV infection rates ranging from 9 to 22% have also been reported in a variety of small, non-representative samples of adolescent transgender females [15,16]. Such reports are notable and significantly higher than the HIV prevalence reported in other adolescent study samples [9,17]. Adolescent transgender females with a history of selling sex may be more than four times as likely to be HIV-infected than their peers [18].

The age at which young people start to engage in behaviours that place them at higher risk of HIV is diverse and varies by country and context; however, evidence shows some begin high-risk behaviours during adolescence. In community consultations, most young people reported starting to inject drugs between 15 and 18 years [19]. In a study among 10–19 year olds living or working on the streets in four cities of Ukraine, 45% of those who reported injecting drugs said that they began doing so before they were 15 years old [20]. Behavioural surveillance indicates that in India 17% of female sex workers initiated selling sex before the age of 15 years, while those in Papua New Guinea reported a mean age of initiation of 17–19 years [21,22].

Although there are unique and diverse issues which contribute to the particular vulnerabilities of adolescent and young key populations, it is also important to recognize their strengths, capacities and resilience, and to recognize these in developing and supporting services and responses to their needs.

## Barriers to services: poor service provision

Young key populations are not adequately reached with appropriate and acceptable HIV prevention, treatment and care interventions and services. Many barriers limit their access to these essential services, or exclude them from using formal health services altogether. Notably, policy and legal barriers related to age of consent to accessing a range of health services including HIV testing and counselling, sexual and reproductive health, harm reduction, and other services provided specifically for key populations limit the ability of young individuals to exercise their right to independent decision-making and prevent them from accessing essential services. For example, in sub-Saharan Africa at least 33 countries have age-based criteria for consenting to HIV testing; 14 of which assert that only a person 18 years of age and above can consent to an HIV test [23].

Adolescents from key population groups are also often subject to significant levels of stigma, discrimination and violence. In many settings, laws that criminalize behaviours such as drug use, sex work and same-sex relationships further marginalize young people and perpetuate their social exclusion from their communities and essential support services. Fearing discrimination and possible legal consequences, many adolescents from key population groups are reluctant to attend HIV testing and treatment services. As such, they remain hidden from services and support networks and are often reluctant to disclose their HIV status to parents and family members in fear of revealing their identity or risk behaviour.

Additionally, most health services are not designed to care for, and address the needs of, adolescents and young people from key populations. Often services are delivered by staff who do not have experience or training in providing care and services for adolescents, and therefore may lack the sensitivity required to work with adolescent key populations. In other settings, services are simply not available, for example, for young transgenders. Available data indicate that young key populations may find services delivered through community and outreach-based programmes more acceptable than those provided in government facilities. This may be in part due to the impact of discriminatory policies including age restrictions, lack of confidentiality, mandatory registration and attitudes towards adolescent and young key populations within facility-based services [24].

## The new WHO guidelines

The new *Consolidated Guidelines on HIV Prevention, Diagnosis, Treatment and Care for Key Populations* have been developed in collaboration with key partners including community-based networks led by and/or for young key populations. They were based on reviews of available peer-reviewed published and grey literature (literature not available through the usual bibliographic databases, for example, programme and project reports), community consultations with young key populations and an extensive effort to collect case examples of good practices from programmes and organizations providing services to key populations. The case studies provide concrete practical examples of services or young key populations and highlight examples of their critical roles in developing and delivering these, including in youth-led advocacy, leadership and empowerment. They summarize the key issues facing key populations and underscore the importance of implementing a comprehensive package of evidence-based services and developing a national strategy to address their unique and diverse needs (Table 1).

**Table 1:** The comprehensive package of HIV prevention, treatment and care interventions and strategies for adults and adolescents as cited in the WHO key population guidelines

Essential health sector interventions	
1.	Comprehensive condom and lubricant programming.
2.	Harm reduction interventions^a^ for substance use (in particular needle and syringe programmes^b^ and opioid substitution therapy).
3.	Behavioural interventions.
4.	HIV testing and counselling.
5.	HIV treatment and care.
6.	Sexual and reproductive health interventions.^c^
7.	Prevention and management of co-infections and other co-morbidities, including viral hepatitis, tuberculosis and mental health conditions.
Essential strategies for an enabling environment	
1.	Supportive legislation, policy and financial commitment, including decriminalization of behaviours of key populations.
2.	Addressing stigma and discrimination.
3.	Community empowerment.
4.	Addressing violence against people from key populations.

a This package is essentially the same as the comprehensive package for HIV prevention, treatment and care for people who inject drugs that has been widely endorsed at the highest level [25,26]. For people who inject drugs, the harm reduction component of the package, and in particular the implementation of needle and syringe programmes and opioid substitution therapy, remains the first priority

b needle and syringe programmes are important for those people who inject drugs and also for transgender people who require sterile injecting equipment to safely inject hormones for gender affirmation. Other important areas include for tattooing, piercing and other forms of skin penetration, which are particularly relevant in prisons and other closed settings

c including contraception, diagnosis and treatment of sexually transmitted infections, cervical screening, etc.

From Ref. [1].

This comprehensive package recommends interventions and strategies relevant for adolescents and adults. The guidelines bring together relevant existing adolescent recommendations such as on HIV testing and counselling as well as provide additional specific adolescent considerations for overall recommendations. For example, in addressing legislative and policy barriers, additional adolescent considerations regarding age of consent barriers are specified.

Furthermore, the guidelines highlight that it is urgent for countries to review their legal policies, initiate the provision of services as well as improve services, include adolescent and young key populations in developing acceptable services and offer opportunities for their meaningful inclusion in defining their HIV and health service needs, developing effective services and participating in research. The resourcefulness and expertise of adolescents and young people is widely recognized, and their empowerment and inclusion in the design and delivery of research, services and interventions is promoted in many settings. In relation to HIV, much can be learned from listening to and involving young people regarding the strategies they already use in keeping themselves and their peers and partners safe, and in finding ways to more easily, safely and sustainably engage with health and other forms of care and support, despite the often considerable barriers and constraints.

Urgent attention must however be given – and practical ways of working within legally constrained settings sought – in order to provide services for young key populations and to prevent their continuing vulnerability to and risk of HIV infection, and to ensure equitable access to HIV testing, treatment and care. We hope that the new guidelines will catalyze better programming for adolescent and young key populations and legitimize their role in designing, developing and delivering them.

## References

[1] WHO (2014). Consolidated guidelines on HIV prevention, diagnosis, treatment and care for key populations.

[2] Hirnschall G, Baggaley R, Verster A, WHO (2014). Consolidated guidelines on HIV prevention, diagnosis, treatment and care for key populations.

[3] UNESCO (2014). In or out: Asia-Pacific review of young key populations in national AIDS strategic plans.

[4] Commission on AIDS in Asia (2008). Redefining AIDS in Asia.

[5] Koblin B, Chesney M, Coates T (2004). EXPLORE Study Team. Effects of a behavioural intervention to reduce acquisition of HIV infection among men who have sex with men: the EXPLORE randomised controlled study. Lancet.

[6] van Griensven F, de Lind van Wijngaarden JW (2010). A review of the epidemiology of HIV infection and prevention responses among MSM in Asia. AIDS.

[7] Clatts MC, Colón-López V, Giang le M, Goldsamt LA (2009). Prevalence and incidence of HCV infection among Vietnam heroin users with recent onset of injection. J Urban Health.

[8] Shannon K, Bright V, Gibson K, Tyndall MW (2007). Sexual and drug-related vulnerabilities for HIV infection among women engaged in survival sex work in Vancouver, Canada. Can J Public Health.

[9] Wilson EC, Garofalo R, Harris RD, Herrick A, Martinez M, Martinez J (2009). Transgender female youth and sex work: HIV risk and a comparison of life factors related to engagement in sex work. AIDS Behav.

[10] UNAIDS (2013). Global report.

[11] Baral S, Sifakis F, Cleghorn F, Beyrer C (2007). Elevated risk for HIV infection among men who have sex with men in low- and middle-income countries 2000–2006: a systematic review. PLoS Med.

[12] UNICEF (2011). Opportunity in crisis: preventing HIV from early adolescence to early adulthood.

[13] US Centers for Disease Control and Prevention (2012). HIV among youth in the US: protecting a generation, in CDC vital signs.

[14] US Centers for Disease Control and Prevention (2012). HIV surveillance in adolescents and young adults.

[15] Schulden JD, Song B, Barros A, Mares-DelGrasso A, Martin CW, Ramirez R (2008). Rapid HIV testing in transgender communities by community-based organizations in three cities. Public Health Rep.

[16] Nemoto T, Operario D, Keatley J, Han L, Soma T (2004). HIV risk behaviors among male-to-female transgender persons of color in San Francisco. Am J Public Health.

[17] Garofalo R, Deleon J, Osmer E, Doll M, Harper GW (2006). Overlooked, misunderstood and at-risk: exploring the lives and HIV risk of ethnic minority male-to-female transgender adolescents. J Adolesc Health.

[18] Operario D, Soma T, Underhill K (2008). Sex work and HIV status among transgender women: systematic review and meta-analysis. J Acquir Immune Defic Syndr.

[19] Youth Research Information Support Education (Youth RISE) and Joint United Nations Programme on HIV/AIDS Experiences of young people who inject drugs and their challenges in accessing harm reduction services. http://www.who.int/hiv/pub/guidelines/keypopulations/en/.

[20] Busza JR, Balakireva OM, Teltschik A, Bondar TV, Sereda YV, Meynell C (2011). Street-based adolescents at high risk of HIV in Ukraine. J Epidemiol Community Health.

[21] UNAIDS (2013). Figures from www.aidsdatahub.org based on national behavioural surveillance reports; Global aids response progress reports: country reports 2012. http://www.aidsdatahub.org.

[22] Asian Human Rights Commission (2002). Bangladesh's child sex workers: no place to go.

[23] WHO (2013). HIV and adolescents: guidance for HIV testing and counselling and care for adolescents living with HIV ANNEX 15: adolescent consent to testing: a review of current policies and issues in sub-Saharan Africa.

[24] Interagency Youth Working Group (2010). U.S. Agency for International Development, the Joint United Nations Programme on HIV/AIDS (UNAIDS) Inter-Agency Task Team on HIV and Young People, and FHI.

[25] WHO (2009). Technical guide for countries to set targets for universal access to HIV prevention, treatment and care for injecting drug users. http://www.who.int/hiv/pub/idu/targetsetting/en/.

[26] WHO (2012). Technical guide for countries to set targets for universal access to HIV prevention, treatment and care for injecting drug users 2012 revision. http://www.who.int/hiv/pub/idu/targets_universal_access/en/.
